# Supplementary material for: Divergent access to 5,6,7-perifused cycles
Source: Nat Commun. 2023 Aug 24;14:5148. doi: 10.1038/s41467-023-40801-0 (PMC10449863; doi:10.1038/s41467-023-40801-0)
Supplement: Supplementary file 1 — Supplementary Information [file 41467_2023_40801_MOESM1_ESM.pdf]

Supplementary Information

Divergent Access to 5,6,7-perifused Cycles

Jingpeng Han[1], Yongjian Yang[1], Yingjian Gong[1], Xuan Tang[1], Yi Tian[1] and Baosheng Li[1,*]

[1]School of Chemistry and Chemical Engineering, Chongqing University. 174 Shazheng Street, Chongqing 400044, (P. R. China).
E-mail: libs@cqu.edu.cn

Table of Content

1 Supplementary Methods ... S1

1.1 General experimental ... S1

1.2 The preparation of substrates **1** and **3** ... S2

1.3 General procedure for the synthesis of substrates **1** ... S3

1.4 The synthesis of deuterated substrate **1a'** ... S4

1.5 General procedure for the synthesis of substrates **3** ... S5

1.6 Optimization conditions for the synthesis of **4a** ... S6

1.7 Procedure A: The synthesis of products **2a** − **2ac** ... S7

1.8 Procedure B: The synthesis of products **4a** − **4x** ... S7

1.9 The gram-scale synthesis of **2c** and **4a** ... S8

1.10 Procedure for the synthetic transformations of **4a** ... S8

1.11 Mechanistic studies ... S9

1.12 Characterization data for key compounds ... S10

1.13 The X-ray structure of **2a**, **2z**, **3a**, **4m** ... S38

1.14 Copies of NMR spectra ... S42

2 Supplementary References ... S147

1 Supplementary Methods

1.1 General experimental

All reactions were carried out under an argon atmosphere with magnetic stirring unless otherwise noted. Tetrahydrofuran (THF) was dried by sodium sand solvent purification system before used, the water content of toluene ($H_2O \approx 0.30\%$) and chloroform ($H_2O \approx 0.35\%$) were determined by Karl Fischer Coulomb titration method[1]. All other reagents were purchased from suppliers and used as received unless noted otherwise. All new compounds were characterized by NMR spectroscopy, IR spectroscopy, high-resolution mass spectroscopy (HRMS).

Proton nuclear magnetic resonance (^1H NMR) spectra were recorded using Bruker 400 MHz or Bruker 500 MHz. Chemical shift (δ) was reported in parts per million (ppm) downfield relative to tetramethylsilane (TMS, 0.0 ppm), $CDCl_3$ (7.26 ppm) or DMSO-d_6 (2.50 ppm). Coupling constants (*J*) were reported in Hz. Multiplicities were reported using the following abbreviations: s (singlet); d (doublet); t (triplet); dd (doublet of doublets); ddd (doublet of doublet of doublets); td (triplet of doublets); dt (doublet of triplets); m (multiplet); Carbon nuclear magnetic resonance (^{13}C NMR) spectra were recorded using Bruker 400 MHz spectrometers at 101 MHz or Bruker 500 MHz spectrometers at 126 MHz. Chemical shift was reported in ppm relative to the carbon resonance of $CDCl_3$ (77.00 ppm) or DMSO-d_6 (39.50 ppm). Fluorine-19 (^{19}F NMR) nuclear magnetic resonance spectra were recorded using Bruker 400 MHz at 377 MHz or Bruker 600 MHz at 564 MHz, respectively.

Infrared (IR) spectra were measured on Thermofisher Nicolet iN10 FM-IR spectrometer using KBr plates.

High resolution mass spectral analysis (HRMS) were recorded on a FT-ICR (Fourier Transform-Ion Cyclotron Resonance) mass spectrometer by using electrospray ionization (ESI) techniques.

Single crystal X-ray diffraction measurements were performed on an Agilent SuperNova-CCD X-Ray diffractometer.

Column chromatographic was performed on 200−300 mesh silica gel or active aluminum oxide, reactions were monitored by thin layer chromatography (TLC) using silica gel GF 254 plates. Visualisation was by ultraviolet fluorescene (λ = 254 nm) and staining with phosphomolybdic acid.

1.2 The preparation of substrates 1 and 3

1a – 1a'[2–4], **3a – 3j**[5] were synthesized according to literatures.

substrates 1

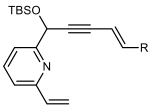

1a, R = 4-OMe-Ph
1b, R = Ph
1d, R = 4-Me-Ph
1f, R = 4-F-Ph
1g, R = 3-thienyl
1h, R = 2-pyridyl
1j, R = cyclohexyl
1k, R = *n*-hexyl

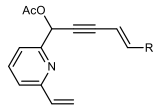

1c, R = Ph
1e, R = 4-Br-Ph
1i, R = styrenyl

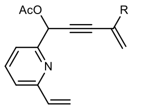

1l, R = 4-OMe-Ph
1m, R = 4-Cl-Ph
1n, R = Me

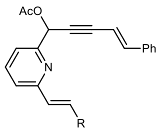

1p, R = Ph
1q, R = 4-OMe-Ph
1r, R = 4-Me-Ph
1s, R = 4-Br-Ph
1u, R = styrenyl
1v, R = cyclohexyl
1w, R = Me

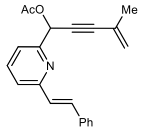

1o

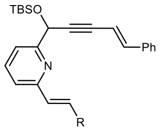

1t, R = 2-pyridyl

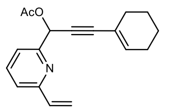

1x, R = H

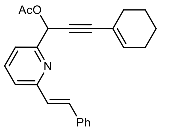

1y

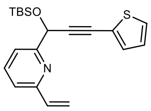

1z

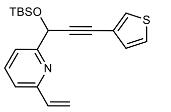

1aa

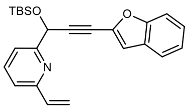

1ab

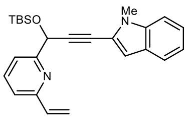

1ac

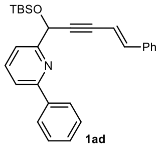

1ad

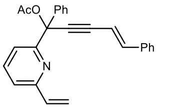

1ae, R = Ph

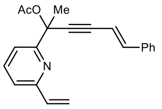

1af, R = Me

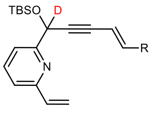

1a', R = 4-OMe-Ph

substrates 3

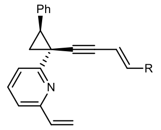

3a, R = Ph
3b, R = 3-thienyl
3c, R = styrenyl
3d, R = *n*-hexyl
3e, R = cyclohexyl

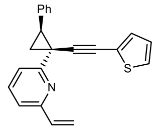

3f

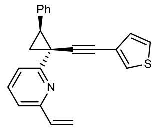

3g

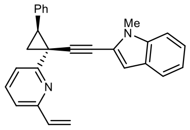

3h

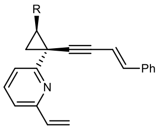

3i, R = 4-Me-Ph

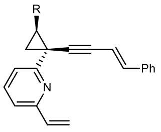

3j, R = 4-F-Ph

1.3 General procedure for the synthesis of substrates 1

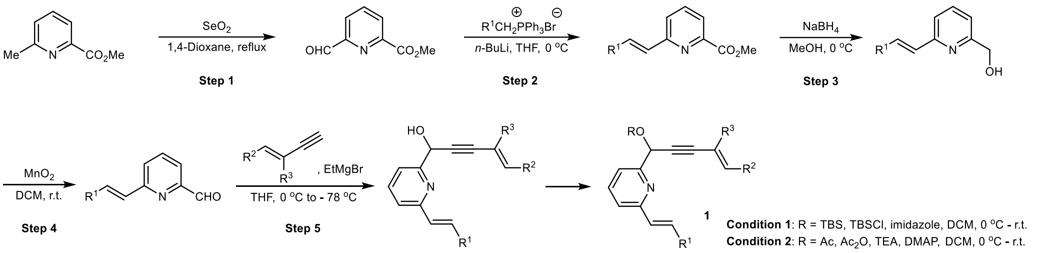

Step 1: The methyl 6-methylpicolinate (100.0 mmol, 1.0 equiv.) was dissolved in 1,4-Dioxane (80.0 mL) for a 200.0 mL round bottom flask, followed SeO_2 (200.0 mmol, 2.0 equiv.) was added, the reaction mixture was refluxed until the material was completely consumed. After completion, the reaction mixture was filtered, and the organic solution was concentrated under *vacuo*, residue was purified by column chromatography on silica gel to afford the methyl 6-formylpicolinate as a colorless solid in 71% yield.

Step 2: The wittig reagent (11.0 mmol, 1.1 equiv.) was dissolved in dried THF (20.0 mL) for a 50.0 mL round bottom flask, followed *n*-BuLi (2.5 M in Hexane, 11.0 mmol, 1.1 equiv.) was added dropwise at 0 °C for 0.5 h, then the methyl 6-formylpicolinate obtained from **step 1** (10.0 mmol, 1.0 equiv.) was added to the mixture at 0 °C and stirred until methyl 6-formylpicolinate was completely consumed. After completion, the reaction mixture was quenched with saturated aq. NH_4Cl solution, the aqueous phase was extracted by DCM (3 × 20 mL), combined the organic solution and dried over anhydrous Na_2SO_4, then the organic solution was concentrated under *vacuo*, the residue was purified by column chromatography on silica gel to afford the methyl 6-vinylpicolinate.

Step 3: The methyl 6-vinylpicolinate (1.0 equiv.) obtained from **step 2** was dissolved in MeOH (15.0 mL) for a 50.0 mL round bottom flask, followed $NaBH_4$ (2.1 equiv.) was added slowly at 0 °C and stirred until the methyl 6-vinylpicolinate was completely consumed. After completion, the reaction mixture was quenched with saturated aq. NH_4Cl solution, the aqueous phase was extracted by DCM (3 × 20 mL), combined the organic solution and dried over anhydrous Na_2SO_4, then the organic solution was concentrated under *vacuo*, the residue was purified by column chromatography on silica gel to afford the alcohol.

Step 4: The alcohol (1.0 equiv.) obtained from **step 3** was dissolved in DCM (15.0 mL) for a 50.0 mL round bottom flask, followed MnO_2 (5.0 equiv.) was added at room temperature and stirred until the alcohol was completely consumed. After completion, the reaction mixture was filtered, and the organic solution was concentrated under *vacuo*, residue was purified by column chromatography on silica gel to afford the aldehyde.

Step 5: The alkyne (5.0 mmol, 1.2 equiv.) was dissolved in dried THF (10.0 mL) for a 50.0 mL round bottom flask, followed EtMgBr (2.0 M in THF, 5.0 mmol, 1.2 equiv.) was added dropwise at 0 °C, the reaction mixture was stirred at 0 °C for 0.5 h and then cooled to – 78 ℃, a solution of aldehyde (4.2 mmol, 1.0 equiv.) obtained from **step 4** in THF (3.0 mL) was added dropwise, the reaction mixture was stirred at – 78 ℃ until the aldehyde was completely consumed. After completion, the reaction mixture was quenched with saturated aq. NH_4Cl solution, the aqueous phase was extracted by DCM (3 × 20 mL), combined organic solution and dried over anhydrous Na_2SO_4, then the organic solution was concentrated under *vacuo*, residue was purified by column chromatography on silica gel to afford the alcohol.

Condition 1: The alcohol (1.0 equiv.) obtained from **step 5** was dissolved in DCM (15.0 mL) for a 50.0 mL round bottom flask, followed imidazole (2.2 equiv.) and TBSCl (2.2 equiv.) were added at 0 °C, and the resulting mixture was warmed to room temperature and stirred until the alcohol was completely consumed. After completion, the organic solution was concentrated under *vacuo*, the residue was purified by column chromatography on silica gel to afford the substrates **1**.

Condition 2: The TEA (2.0 equiv.), DMAP (10.0 mol%), and alcohol (1.0 equiv.) obtained from **step 5** were dissolved in DCM (15.0 mL) for a 50.0 mL round bottom flask, followed acetic anhydride (2.0 equiv.) was added dropwise at 0 °C, and the resulting mixture was warmed to room temperature and stirred until alcohol completely consumed. After completion, the reaction mixture was quenched with saturated aq. NH_4Cl solution, the aqueous phase was extracted by DCM (3 × 20 mL), combined organic solution and dried over anhydrous Na_2SO_4, then the organic solution concentrated was under *vacuo*, residue was purified by column chromatography on silica gel to afford the substrates **1**.

1.4 The synthesis of deuterated substrate 1a'

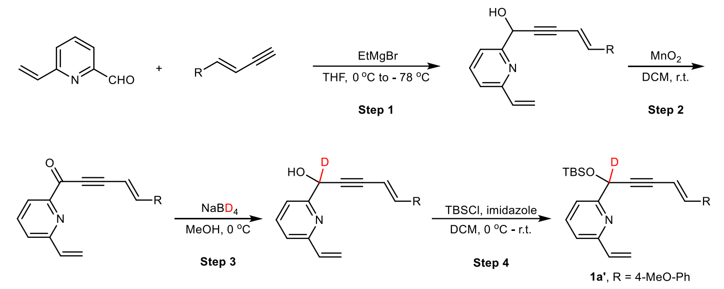

Step 1: The alkyne (5.0 mmol, 1.2 equiv.) was dissolved in dried THF (10.0 mL) for a 50.0 mL round bottom flask, followed EtMgBr (2.0 M in THF, 5.0 mmol, 1.2 equiv.) was added dropwise at 0 °C, the reaction mixture was stirred at 0 °C for 0.5 h and then cooled to – 78 °C, a solution of aldehyde (4.2 mmol, 1.0 equiv.) in THF (3.0 mL) was added dropwise, the reaction mixture was stirred at –78 °C until the aldehyde was completely consumed. After completion, the reaction mixture was quenched with saturated aq. NH_4Cl solution, the aqueous phase was extracted by DCM (3 × 20 mL), combined organic solution and dried over anhydrous Na_2SO_4, then the organic solution was concentrated under *vacuo*, residue was purified by column chromatography on silica gel to afford the alcohol.

Step 2: The alcohol (2.8 mmol, 1.0 equiv.) obtained from **step 1** was dissolved in DCM (10.0 mL) for a 50.0 mL round bottom flask, followed MnO_2 (14.0 mmol, 5.0 equiv.) was added at room temperature, stirred at room temperature until the alcohol was completely consumed. After completion, the reaction mixture was filtered, and the organic solution was concentrated under *vacuo*, residue was purified by column chromatography on silica gel to afford ketone.

Step 3: The ketone (2.7 mmol, 1.0 equiv.) obtained from **step 2** was dissolved in MeOH (10.0 mL) for a 50.0 mL round bottom flask, followed $NaBD_4$ (3.0 mmol, 1.1 equiv.) was added slowly at 0 °C, the reaction mixture was stirred at 0 °C until the ketone was completely consumed. After completion, the reaction mixture was quenched with saturated aq. NH_4Cl solution, the aqueous phase was extracted by DCM (3 × 20 mL), combined organic solution and dried over anhydrous Na_2SO_4, then the organic solution was concentrated under vacuo, residue was purified by column chromatography on silica gel to afford the alcohol.

Step 4: The alcohol (2.5 mmol, 1.0 equiv.) obtained from **step 3** was dissolved in DCM (10.0 mL)

for a 50.0 mL round bottom flask, followed imidazole (5.5 mmol, 2.2 equiv.) and TBSCl (5.5 mmol, 2.2 equiv.) were added at 0 °C, and the resulting mixture was warmed to room temperature and stirred until the alcohol was completely consumed. After completion, the organic solution was concentrated under *vacuo*, the residue was purified by column chromatography on silica gel to afford the **1a'**.

1.5 General procedure for the synthesis of substrates 3

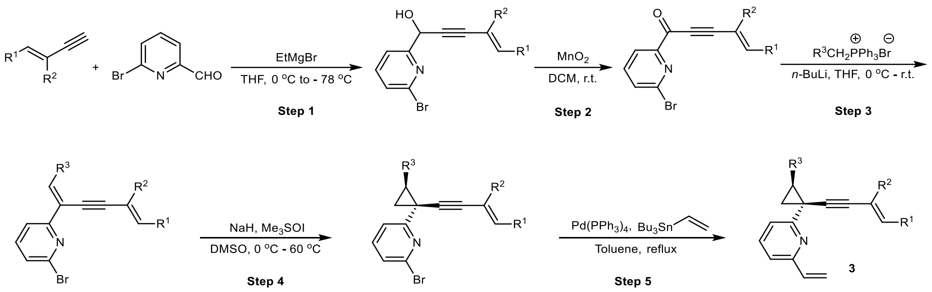

Step 1: The alkyne (12.0 mmol, 1.2 equiv.) was dissolved in dried THF (20.0 mL) for a 100.0 mL round bottom flask, followed EtMgBr (2.0 M in THF, 12.0 mmol, 1.2 equiv.) was added dropwise at 0 °C, the reaction mixture was stirred at 0 °C for 1.0 h and then cooled to – 78 °C, a solution of aldehyde (10.0 mmol, 1.0 equiv.) in THF (5.0 mL) was added dropwise, the reaction mixture was stirred at – 78 °C until the aldehyde was completely consumed. After completion, the reaction mixture was quenched with saturated aq. NH_4Cl solution, the aqueous phase was extracted by DCM (3 × 20 mL), combined organic solution and dried over anhydrous Na_2SO_4, then the organic solution was concentrated under *vacuo*, residue was purified by column chromatography on silica gel to afford the alcohol.

Step 2: The alcohol (1.0 equiv.) obtained from **step 1** was dissolved in DCM (20.0 mL) for a 50.0 mL round bottom flask, followed MnO_2 (5.0 equiv.) was added at room temperature, stirred at room temperature until the alcohol was completely consumed. After completion, the reaction mixture was filtered, and the organic solution was concentrated under *vacuo*, residue was purified by column chromatography on silica gel to afford ketone.

Step 3: The wittig reagent (1.1 equiv.) was dissolved in dried THF (20.0 mL) for a 50.0 mL round bottom flask, followed *n*-BuLi (2.5 M in Hexane, 1.1 equiv.) was added dropwise at 0 °C, the reaction was stirred at 0 °C for 0.5 h, then the ketone (1.0 equiv.) obtained from **step 2** was added to the mixture at 0 °C, and the resulting mixture was warmed to room temperature and stirred until the ketone was completely consumed. After completion, the reaction mixture was quenched with saturated aq. NH_4Cl solution, the aqueous phase was extracted by DCM (3 × 20 mL), combined organic solution and dried over anhydrous Na_2SO_4, then the organic solution was concentrated under *vacuo*, residue was purified by column chromatography on silica gel to afford diene.

Step 4: The trimethylsulfoxonium iodide (1.5 equiv.) was dissolved in DMSO (15.0 mL) for a 50.0 mL round bottom flask, followed NaH (1.5 equiv.) was added slowly at 0 °C, the reaction mixture was stirred at 0 °C for 0.5 h, a solution of diene (1.0 equiv.) obtained from **step 3** in DMSO (5.0 mL) was added dropwise, then the mixture was heat to 60 °C and stirred until the diene was completely consumed. After completion, the reaction mixture was quenched with saturated aq. NH_4Cl solution, the aqueous phase was extracted by DCM (3 × 20 mL), combined organic solution and dried over anhydrous Na_2SO_4, then the organic solution was concentrated under *vacuo*, residue

was purified by column chromatography on silica gel to afford the bromocyclopropane.

Step 5: The $Pd(PPh_3)_4$ (1.0 mol%) and bromocyclopropane (1.0 equiv.) obtained from **step 4** were dissolved in toluene (15.0 mL) for a 50.0 mL round bottom flask, followed tributyl(vinyl)tin (1.1 equiv.) was added at room temperature, the reaction mixture was refluxed until the material were completely consumed, then reaction mixture cooled to room temperature, KF (2.0 equiv.) was added and stirred for 2.0 h. After completion, the reaction mixture was filtered, and the organic solution was concentrated under *vacuo*, residue was purified by column chromatography on silica gel to afford the **3a** – **3j** as single diastereoisomers.

1.6 Optimization conditions for the synthesis of 4a

Supplementary Table 1. Optimization conditions for the synthesis of 4a[a,b]

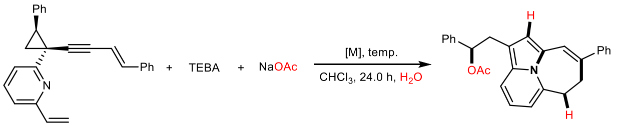

3a 4a

entry	[M]	NaOAc/(equiv.)	Solvents	Temp. (°C)	yield (%)
1	CuTc	2	$CHCl_3$	90	11
2	$Cu(OAc)_2$	2	$CHCl_3$	90	15
3	$CuSO_4$	2	$CHCl_3$	90	27
4	$Cu(OTf)_2$	2	$CHCl_3$	90	38
5	AgOAc	2	$CHCl_3$	90	41
6	CF_3CO_2Ag	2	$CHCl_3$	90	44
7	Ag_3PO_4	2	$CHCl_3$	90	65
8	$AgSbF_6$	2	$CHCl_3$	90	36
9	AgCl	2	$CHCl_3$	90	46
10	Ag_3PO_4	2	THF	90	22
11	Ag_3PO_4	2	toluene	90	19
12	Ag_3PO_4	2	DMF	90	41
13	Ag_3PO_4	3	$CHCl_3$	90	74
**14**	**Ag_3PO_4**	**3**	**$CHCl_3$**	**100**	**80**
15	Ag_3PO_4	3	$CHCl_3$	110	77

[a]All reactions were performed with **3a** (0.1 mmol, 1.0 equiv.), catalyst (20.0 mol%), TEBA (0.2 mmol, 2.0 equiv.) and NaOAc in the solvent (2.0 mL) were stirred for 24.0 h. [b]Isolated yield. TEBA: benzyltriethylammonium chloride.

1.7 Procedure A: The synthesis of products 2a – 2ac

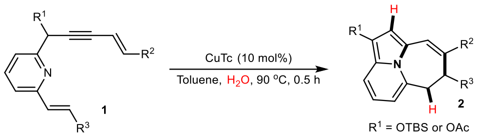

The freshly prepared **1** (0.05 mmol, 1.0 equiv.), CuTc (10.0 mol%), and toluene (1.0 mL) were added to a 5.0 mL round bottom flask at room temperature, the reaction mixture was stirred at 90 °C for 0.5 h and then cooled to room temperature. After completion, the reaction mixture was filtered, and the organic solution was concentrated under *vacuo*, residue was purified by column chromatography on alkaline alumina to afford the **2a** – **2ac**.

1.8 Procedure B: The synthesis of products 4a – 4x

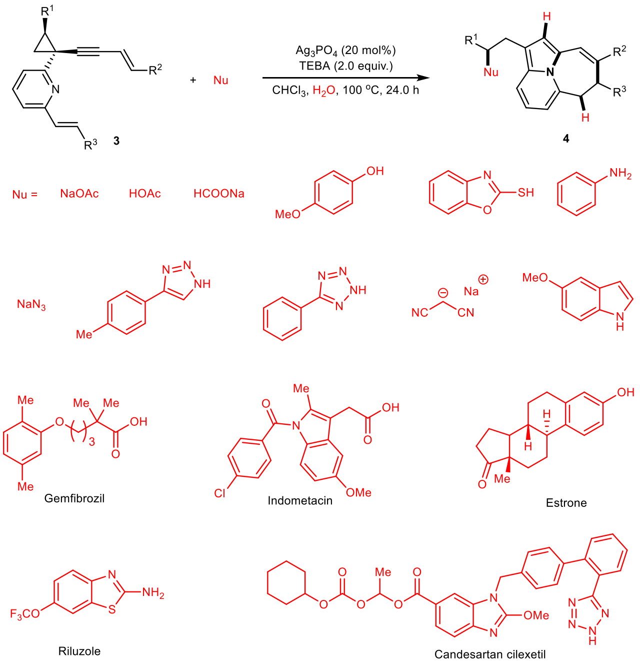

Ag_3PO_4 (20.0 mol%), TEBA (0.2 mmol, 2.0 equiv.) and nucleophilic reagent (0.3 mmol, 3.0 equiv.) were added to a solution of freshly prepared **3** (0.1 mmol, 1.0 equiv.) (**1ae** and **1af** did not react with the recovery of materials) in $CHCl_3$ (2.0 mL) for a Schlenk tube at room temperature, the reaction mixture was stirred at 100 °C for 24.0 h and then cooled to room temperature. After completion, the reaction mixture was filtered, and the organic solution was concentrated under *vacuo*, residue was purified by column chromatography on silica gel to afford the **4a** – **4x**.

1.9 The gram-scale synthesis of 2c and 4a

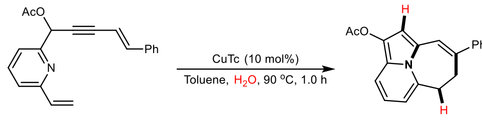

1c, 0.80 g   **2c**, 0.65 g, 81%

The freshly prepared **1c** (2.64 mmol, 0.80 g, 1.0 equiv.), CuTc (10.0 mol%), and toluene (20.0 mL) were added to a 50.0 mL round bottom flask at room temperature, the reaction mixture was stirred at 90 °C for 1.0 h and then cooled to room temperature. After completion, the reaction mixture was filtered, and the organic solution was concentrated under *vacuo*, residue was purified by column chromatography on alkaline alumina to afford the **2c** as yellow thick liquid (0.65 g, 81%).

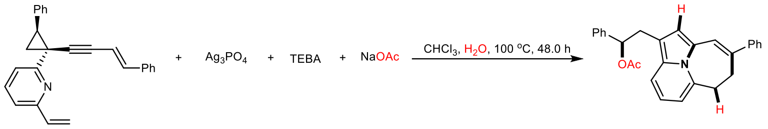

3a, 1.00 g   **4a**, 0.83 g, 71%

Ag_3PO_4 (20.0 mol%), TEBA (5.76 mmol, 2.0 equiv.) and Nucleophilic reagent (8.64 mmol, 3.0 equiv.) were added to a solution of freshly prepared **3a** (2.88 mmol, 1.00 g, 1.0 equiv.) in $CHCl_3$ (20.0 mL) for a 50.0 mL round bottom flask at room temperature, the reaction mixture was stirred at 100 °C for 48.0 h and then cooled to room temperature. After completion, the reaction mixture was filtered, and the organic solution was concentrated under *vacuo*, residue was purified by column chromatography on silica gel to afford **4a** as yellow thick liquid (0.83 g, 71%).

1.10 Procedure for the synthetic transformations of 4a

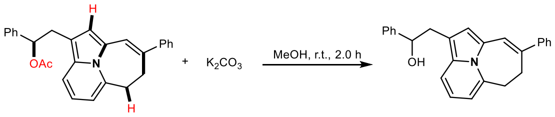

4a   **4a-1**

The **4a** (0.1 mmol, 1.0 equiv.), K_2CO_3 (0.4 mmol, 4.0 equiv.) and MeOH (1.0 mL) were added to a 5.0 mL round bottom flask at room temperature, then the reaction mixture was stirred at room temperature for 2.0 h. After completion, the organic solution was concentrated under *vacuo* and purified by column chromatography on silica gel afford **4a-1** in 96% yield as yellow amorphous solid.

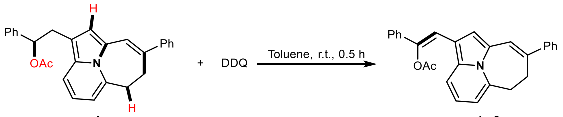

4a   **4a-2**

The **4a** (0.1 mmol, 1.0 equiv.), DDQ (0.15 mmol, 1.5 equiv.) and toluene (2.0 mL) were added to a 5.0 mL round bottom flask at room temperature, then the reaction mixture was stirred at room temperature for 0.5 h. After completion, the organic solution was concentrated under *vacuo*, the residue was purified by column chromatography on silica gel to afford **4a-2** in 86% yield as yellow amorphous solid.

1.11 Mechanistic studies

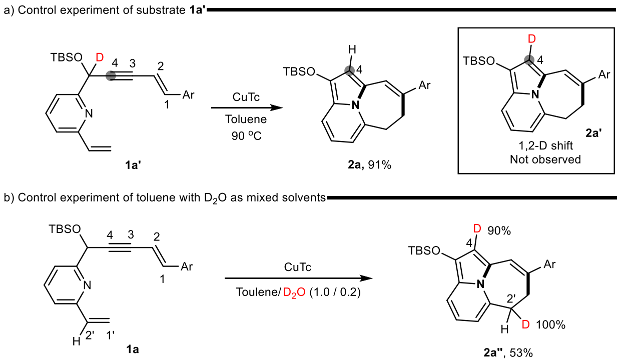

Control experiment 1: The freshly prepared **1a'** (0.05 mmol, 1.0 equiv.), CuTc (10.0 mol%), and toluene (1.0 mL) were added to a 5.0 mL round bottom flask at room temperature, the reaction mixture was stirred at 90 °C for 0.5 h and then cooled to room temperature. After completion, the reaction mixture was filtered, and the organic solution was concentrated under *vacuo,* residue was purified by column chromatography on alkaline alumina to afford the **2a** in 91% yield as yellow amorphous solid.

Control experiment 2: The freshly prepared **1a** (0.05 mmol, 1.0 equiv.), CuTc (10.0 mol%), dried toluene (1.0 mL) and D_2O (0.2 mL) were added to a 5.0 mL round bottom flask at room temperature, the mixture was stirred at 90 °C for 0.5 h and then cooled to room temperature. After completion, the reaction mixture was filtered, and the organic solution was concentrated under *vacuo*, residue was purified by column chromatography on alkaline alumina to afford the **2a''** in 53% yield as yellow amorphous solid.

1.12 Characterization data for key compounds

(E)-2-(1-((tert-butyldimethylsilyl)oxy)-5-(4-methoxyphenyl) pent-4-en-2-yn-1-yl)-6-vinylpyridine

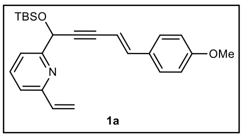

Compound **1a** was obtained as a yellow liquid; **^{1}H NMR (500 MHz, CDCl$_3$)** δ 7.68 (t, J = 7.5 Hz, 1H), 7.55 (d, J = 7.5 Hz, 1H), 7.29 – 7.26 (m, 3H), 6.89 – 6.82 (m, 4H), 6.21 (d, J = 16.0 Hz, 1H), 6.04 (d, J = 16.0 Hz, 1H), 5.72 (s, 1H), 5.48 (d, J = 11.0 Hz, 1H), 3.78 (s, 3H), 0.97 (s, 9H), 0.25 (s, 3H), 0.18 (s, 3H); **^{13}C NMR (126 MHz, CDCl$_3$)** δ 160.6, 160.0, 154.8, 140.9, 137.3, 136.9, 129.0, 127.5, 119.8, 119.0, 118.3, 114.0, 105.4, 91.0, 85.1, 67.3, 55.2, 25.8, 18.3, -4.5, -4.9; **IR (cm^{-1})**: v 3028, 2924, 2850, 2210, 1631, 1449, 1395, 1083, 957, 854; **HRMS** (ESI) m/z calculated for $C_{25}H_{32}NO_2Si$ $[M + H]^+$: 406.2197, found: 406.2199.

(E)-2-(1-((tert-butyldimethylsilyl)oxy)-5-(p-tolyl) pent-4-en-2-yn-1-yl)-6-vinylpyridine

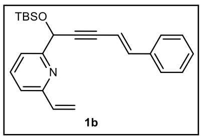

Compound **1b** was obtained as a yellow liquid; **^{1}H NMR (500 MHz, CDCl$_3$)** δ 7.72 (t, J = 7.5 Hz, 1H), 7.57 (d, J = 7.5 Hz, 1H), 7.38 – 7.27 (m, 6H), 6.94 (d, J = 17.0 Hz, 1H), 6.86 (dd, J_1 = 17.0 Hz, J_2 = 10.5 Hz, 1H), 6.25 – 6.18 (m, 2H), 5.74 (s, 1H), 5.50 (d, J = 10.5 Hz, 1H), 0.98 (s, 9H), 0.26 (s, 3H), 0.19 (s, 3H); **^{13}C NMR (126 MHz, CDCl$_3$)** δ 160.5, 154.9, 141.4, 137.4, 136.9, 136.2, 128.6, 128.5, 126.2, 119.9, 119.0, 118.3, 107.9, 91.8, 84.8, 67.3, 25.8, 18.4, -4.5, -4.9; **IR (cm^{-1})**: v 3029, 2926, 2824, 2206, 1601, 1491, 1361, 1082, 748; **HRMS** (ESI) m/z calculated for $C_{24}H_{30}NOSi$ $[M + H]^+$: 376.2091, found: 376.2089.

(E)-5-phenyl-1-(6-vinylpyridin-2-yl)pent-4-en-2-yn-1-yl acetate

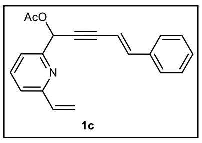

Compound **1c** was obtained as a yellow liquid; **^{1}H NMR (500 MHz, CDCl$_3$)** δ 7.67 (t , J = 7.5 Hz, 1H), 7.47 (d, J = 7.5 Hz, 1H), 7.34 – 7.24 (m, 6H), 6.99 (d, J = 17.5 Hz, 1H), 6.82 (dd, J_1 = 17.5 Hz, J_2 = 11.0 Hz, 1H), 6.66 (s, 1H), 6.23 (d, J = 17.5 Hz, 1H), 6.17 (d, J = 17.5 Hz, 1H), 5.48 (d, J = 11.0 Hz, 1H), 2.14 (s, 3H); **^{13}C NMR (126 MHz, CDCl$_3$)** δ 169.3, 155.5, 155.4, 142.6, 137.3, 136.4, 135.6, 128.7, 128.5, 126.2, 120.6, 120.2, 118.8, 106.8, 86.9, 86.4, 67.3, 20.8; **IR (cm^{-1})**: v 3028, 2928, 2815, 2214, 1746, 1620, 1021, 928, 749; **HRMS** (ESI) m/z calculated for $C_{20}H_{18}NO_2$ $[M + H]^+$: 304.1332, found: 304.1326.

(E)-2-(1-((tert-butyldimethylsilyl)oxy)-5-(p-tolyl)pent-4-en-2-yn-1-yl)-6-vinylpyridine

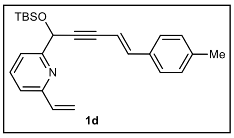

Compound **1d** was obtained as a yellow liquid; **^{1}H NMR (500 MHz, CDCl$_3$)** δ 7.66 (t, J = 7.5 Hz, 1H), 7.52 (d, J = 7.5 Hz, 1H), 7.26 – 7.22 (m, 3H), 7.09 – 7.07 (m, 2H), 6.88 – 6.79 (m, 2H), 6.18 (d, J = 17.0 Hz, 1H), 6.10 (d, J = 17.0 Hz, 1H), 5.69 (s, 1H), 5.45 (d, J = 11.0 Hz, 1H), 2.30 (s, 3H), 0.93 (s, 9H), 0.21 (s, 3H), 0.14 (s, 3H); **^{13}C NMR (126 MHz, CDCl$_3$)** δ 160.5, 154.8, 141.4, 138.6, 137.4, 136.9, 133.4, 129.3, 126.1, 119.8, 119.0, 118.3, 106.7, 91.4, 85.0, 67.3, 25.8, 21.3, 18.3, -4.5, -4.9; **IR (cm^{-1})**: v 3026, 2954, 2856, 2210, 1628, 1454, 1389, 1082, 918, 837; **HRMS** (ESI) m/z calculated for $C_{25}H_{32}NOSi$ $[M + H]^+$: 390.2248, found: 390.2247.

(*E*)-5-(4-bromophenyl)-1-(6-vinylpyridin-2-yl)pent-4-en-2-yn-1-yl acetate

Compound **1e** was obtained as a yellow liquid; **^{1}H NMR (400 MHz, $CDCl_3$)** δ 7.69 (t, J = 7.6 Hz, 1H), 7.46 (d, J = 7.6 Hz, 1H), 7.43 (d, J = 1.2 Hz, 1H), 7.41(d, J = 1.2 Hz, 1H), 7.31 (d, J = 7.6 Hz, 1H), 7.21 (d, J = 1.2 Hz, 1H), 7.18 (d, J = 1.2 Hz, 1H), 6.91 (d, J = 16.0 Hz, 1H), 6.83 (dd, J_1 = 17.6 Hz, J_2 = 10.8 Hz, 1H), 6.63 (d, J = 1.6 Hz, 1H), 6.23 (dd, J_1 = 17.6 Hz, J_2 = 1.6 Hz, 1H), 6.18 – 6.13 (m, 1H), 5.50 (d, J = 10.8 Hz, 1H), 2.16 (s, 3H); **^{13}C NMR (101 MHz, $CDCl_3$)** δ 169.4, 155.5, 141.3, 137.4, 134.6, 131.7, 127.7, 122.7, 120.7, 120.2, 118.9, 107.7, 87.6, 86.1, 67.3, 20.9; **IR (cm^{-1})**: *v* 3036, 2927, 2833, 2100, 1745, 1627, 1071, 959, 804; **HRMS** (ESI) m/z calculated for $C_{20}H_{16}BrNNaO_2$ $[M + Na]^+$: 404.0257, found: 404.0251.

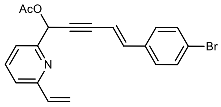

1e

(*E*)-2-(1-((tert-butyldimethylsilyl)oxy)-5-(4-fluorophenyl)pent-4-en-2-yn-1-yl)-6-vinylpyridine

Compound **1f** was obtained as a yellow liquid; **^{1}H NMR (500 MHz, $CDCl_3$)** δ 7.60 (t, J = 7.5 Hz, 1H), 7.47 (d, J = 7.5 Hz, 1H), 7.23 – 7.18 (m, 3H), 6.91 – 6.88 (m, 2H), 6.80 – 6.73 (m, 2H), 6.13 (d, J = 17.5 Hz, 1H), 6.00 (d, J = 17.5 Hz, 1H), 5.65 (s, 1H), 5.39 (d, J = 11.0 Hz, 1H), 0.89 (s, 9H), 0.17 (s, 3H), 0.10 (s, 3H); **^{13}C NMR (126 MHz, $CDCl_3$)** δ 163.8, 161.8, 160.4, 154.8, 140.1, 137.3, 136.9, 132.3 (d, J = 3.4 Hz), 127.8 (d, J = 8.1 Hz), 119.9, 118.6 (d, J = 78.5 Hz), 115.6 (d, J = 21.8 Hz), 107.6 (d, J = 2.4 Hz), 91.8, 84.5, 67.3, 25.8, 18.3, -4.5, -4.9; **^{19}F NMR (564 MHz, $CDCl_3$)** δ -112.47 (t, J = 7.3 Hz, 1F); **IR (cm^{-1})**: *v* 3022, 2929, 2857, 2205, 1610, 1455, 1361, 1096, 934, 837; **HRMS** (ESI) m/z calculated for $C_{24}H_{29}FNOSi$ $[M + H]^+$: 394.1997, found: 394.2000.

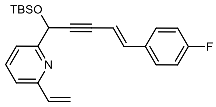

1f

(*E*)-2-(1-((tert-butyldimethylsilyl)oxy)-5-(thiophen-3-yl)pent-4-en-2-yn-1-yl)-6-vinylpyridine

Compound **1g** was obtained as a yellow liquid; **^{1}H NMR (500 MHz, $CDCl_3$)** δ 7.68 (t, J = 7.5 Hz, 1H), 7.53 (d, J = 7.5 Hz, 1H), 7.28 – 7.24 (m, 2H), 7.19 (s, 1H), 7.18 (d, J = 4.0 Hz, 1H), 6.90 (d, J = 17.5 Hz, 1H), 6.83 (dd, J_1 = 17.5 Hz, J_2 = 11.0 Hz, 1H), 6.19 (d, J = 17.5 Hz, 1H), 6.00 (d, J = 17.5 Hz, 1H), 5.69 (s, 1H), 5.47 (d, J = 11.0 Hz, 1H), 0.95 (s, 9H), 0.22 (s, 3H), 0.16 (s, 3H); **^{13}C NMR (126 MHz, $CDCl_3$)** δ 160.5, 154.8, 139.1, 137.3, 136.9, 135.4, 126.4, 124.3, 123.5, 119.8, 119.0, 118.3, 107.6, 91.7, 84.7, 67.3, 25.8, 18.3, -4.5, -4.9; **IR (cm^{-1})**: *v* 3033, 2955, 2856, 2206, 1664, 1454, 1361, 1085, 949, 749; **HRMS** (ESI) m/z calculated for $C_{22}H_{28}NOSSi$ $[M + H]^+$: 382.1655, found: 382.1658.

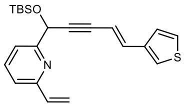

1g

(*E*)-2-(1-((tert-butyldimethylsilyl)oxy)-5-(pyridin-2-yl)pent-4-en-2-yn-1-yl)-6-vinylpyridine

Compound **1h** was obtained as a yellow liquid; **^{1}H NMR (500 MHz, $CDCl_3$)** δ 8.52 (s, 1H), 7.66 (t, J = 7.5 Hz, 1H), 7.58 (t, J = 7.5 Hz, 1H), 7.52 (d, J = 7.5 Hz, 1H), 7.24 (s, 1H), 7.18 (d, J = 7.5 Hz, 1H), 7.12 – 7.10 (m, 1H), 6.92 (d, J = 17.5 Hz, 1H), 6.81 (dd, J_1 = 17.5 Hz, J_2 = 10.5 Hz, 1H), 6.73 (d, J = 17.5 Hz, 1H), 6.18 (d, J = 17.5 Hz, 1H), 5.70 (s, 1H), 5.45 (d, J = 10.5 Hz, 1H), 0.94 (s, 9H), 0.21 (s, 3H), 0.15 (s, 3H); **^{13}C NMR (126 MHz, $CDCl_3$)** δ 160.3, 154.9, 154.1, 149.7, 140.3, 137.3, 136.9, 136.5, 122.9, 122.0, 119.9, 119.0, 118.3, 112.4, 93.9, 84.5, 67.4, 25.8, 18.3, -4.5, -4.9; **IR (cm^{-1})**: *v* 3022, 2928, 2856, 2110, 1584, 1455, 1092, 991, 779; **HRMS** (ESI) m/z calculated for $C_{23}H_{29}N_2OSi$ $[M + H]^+$: 377.2044, found: 377.2047.

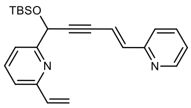

1h

(4*E*,6*E*)-7-phenyl-1-(6-vinylpyridin-2-yl)hepta-4,6-dien-2-yn-1-yl acetate

Compound **1i** was obtained as a yellow liquid; **^{1}H NMR (400 MHz, CDCl$_3$)** δ 7.71 (d, *J* = 7.6 Hz, 1H), 7.50 (d, *J* = 7.6 Hz, 1H), 7.43 (d, *J* = 1.2 Hz, 1H), 7.41 (s, 1H), 7.36 – 7.32 (m, 3H), 7.28 – 7.25 (m, 1H), 6.91 – 6.86 (m, 1H), 6.84 (d, *J* = 3.2 Hz, 1H), 6.81 (d, *J* = 4.4 Hz, 1H), 6.69 (d, *J* = 2.0 Hz, 1H), 6.66 – 6.62 (m, 1H), 6.27 (dd, J_1 = 17.2 Hz, J_2 = 1.2 Hz, 1H), 5.82 – 5.74 (m, 1H), 5.54 (dd, J_1 = 10.8 Hz, J_2 = 1.2 Hz, 1H), 2.19 (s, 3H); **^{13}C NMR (101 MHz, CDCl$_3$)** δ 169.6, 155.7, 155.6, 143.3, 137.4, 136.6, 136.5, 135.4, 128.7, 128.2, 127.7, 126.7, 120.7, 120.3, 118.9, 110.1, 88.0, 86.8, 67.5. 21.0; **IR (cm^{-1})**: *v* 3025, 2941, 2855, 2210, 1717, 1615, 1020, 984, 749; **HRMS** (ESI) m/z calculated for $C_{22}H_{19}NNaO_2$ [M + Na]$^+$: 352.1308, found: 352.1305.

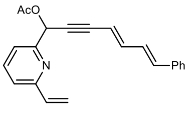

1i

(*E*)-2-(1-((tert-butyldimethylsilyl)oxy)-5-cyclohexylpent-4-en-2-yn-1-yl)-6-vinylpyridine

Compound **1j** was obtained as a yellow liquid; **^{1}H NMR (400 MHz, CDCl$_3$)** δ 7.62 (t, *J* = 8.0 Hz, 1H), 7.51 (d, *J* = 8.0 Hz, 1H), 7.25 (d, *J* = 8.0 Hz, 1H), 6.82 (dd, J_1 = 17.6 Hz, J_2 = 10.8 Hz, 1H), 6.18 (dd, J_1 = 17.6 Hz, J_2 = 1.2 Hz, 1H), 6.07 (dd, J_1 = 16.0 Hz, J_2 = 6.8 Hz, 1H), 5.63 (d, *J* = 1.2 Hz, 1H), 5.47 – 5.41 (m, 2H), 2.03 – 1.96 (m, 1H), 1.73 – 1.61 (m, 5H), 1.29 – 1.00 (m, 5H), 0.93 (s, 9H), 0.20 (s, 3H), 0.13 (s, 3H); **^{13}C NMR (101 MHz, CDCl$_3$)** δ 160.7, 154.7, 150.4, 137.3, 137.0, 119.7, 119.0, 118.2, 106.9, 88.1, 84.7, 77.3, 77.0, 76.7, 67.3, 41.1, 32.1, 26.0, 25.8, 25.8, 18.3, -4.5, -5.0; **IR (cm^{-1}):** *v* 3058, 2928, 2855, 2213, 1626, 1453, 1361, 1082, 957, 749; **HRMS** (ESI) m/z calculated for $C_{24}H_{36}NOSi$ [M + H]$^+$: 382.2561, found: 382.2554.

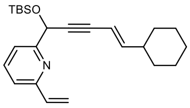

1j

(*E*)-2-(1-((tert-butyldimethylsilyl)oxy)dodec-4-en-2-yn-1-yl)-6-vinylpyridine

Compound **1k** was obtained as a yellow liquid; **^{1}H NMR (500 MHz, CDCl$_3$)** δ 7.66 (t, *J* = 7.5 Hz, 1H), 7.51 (d, *J* = 7.5 Hz, 1H), 7.25 (d, *J* = 7.5 Hz, 1H), 6.81 (dd, J_1 = 17.5 Hz, J_2 = 11.0 Hz, 1H), 6.18 (d, *J* = 17.5 Hz, 1H), 6.15 – 6.09 (m, 1H), 5.64 (s, 1H), 5.49 (m, 2H), 2.07 (dd, J_1 = 14.0 Hz, J_2 = 7.0 Hz, 2H), 1.37 – 1.33 (m, 2H), 1.30 – 1.25 (m, 8H), 0.94 (s, 9H), 0.87 (t, *J* = 6.5 Hz, 3H), 0.21 (s, 3H), 0.14 (s, 3H); **^{13}C NMR (126 MHz, CDCl$_3$)** δ 160.7, 154.8, 145.1, 137.2, 137.0, 119.7, 118.9, 118.1, 109.1, 87.9, 84.5, 67.3, 33.0, 31.7, 29.0, 29.0, 28.6, 22.6, 18.3, 14.0, -4.5, -4.9; **IR (cm^{-1})**: *v* 3015, 2926, 2851, 2207, 1621, 1455, 1252, 1082, 956, 749; **HRMS** (ESI) m/z calculated for $C_{25}H_{40}NOSi$ [M + H]$^+$: 398.2874, found: 398.2873.

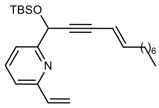

1k

4-(4-methoxyphenyl)-1-(6-vinylpyridin-2-yl)pent-4-en-2-yn-1-yl acetate

Compound **1l** was obtained as a yellow liquid; **^{1}H NMR (400 MHz, DMSO-*d$_6$*)** δ 7.88 (t, *J* = 7.6 Hz, 1H), 7.64 – 7.60 (m, 2H), 7.53 (d, *J* = 7.6 Hz, 2H), 6.94 – 6.89 (m, 2H), 6.86 (dd, J_1 = 17.6 Hz, J_2 = 10.8 Hz, 1H), 6.63 (s, 1H), 6.29 (dd, J_1 = 17.6 Hz, J_2 = 1.2 Hz, 1H), 6.05 (s, 1H), 5.60 (s, 1H), 5.54 (dd, J_1 = 10.8 Hz, J_2 = 1.2 Hz, 1H), 3.76 (s, 3H), 2.16 (s, 3H); **^{13}C NMR (101 MHz, DMSO-*d$_6$*)** δ 169.3, 159.7, 155.2, 154.9, 138.3, 136.4, 128.4, 128.0, 127.1, 121.4, 120.4, 120.4, 119.2, 113.9, 86.9, 86.0, 66.7, 55.2, 20.6; **IR (cm^{-1})**: *v* 3017, 2933, 2856, 2210, 1745, 1607, 1027, 993; **HRMS** (ESI) m/z calculated for $C_{21}H_{19}NNaO_3$ [M + Na]$^+$: 356.1257, found: 356.1267.

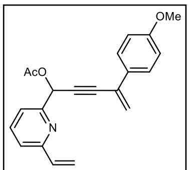

1l

4-(4-chlorophenyl)-1-(6-vinylpyridin-2-yl)pent-4-en-2-yn-1-yl acetate

Compound **1m** was obtained as a yellow liquid; **^{1}H NMR (400 MHz, DMSO-*d$_6$*)** δ 7.87 (t, *J* = 7.6 Hz, 1H), 7.71 – 7.67 (m, 2H), 7.52 (d, *J* = 7.6 Hz, 2H), 7.43 – 7.40 (m, 2H), 6.85 (dd, J_1 = 17.6 Hz, J_2 = 10.8 Hz, 1H), 6.64 (s, 1H), 6.28 (dd, J_1 = 17.6 Hz, J_2 = 1.2 Hz, 1H), 6.21 (s, 1H), 5.77 (s, 1H), 5.53 (dd, J_1 = 10.8 Hz, J_2 = 1.2 Hz, 1H), 2.16 (s, 3H); **^{13}C NMR (101 MHz, DMSO-*d$_6$*)** δ 169.8, 155.5, 155.4, 138.8, 136.9, 135.3, 133.9, 129.1, 128.0, 127.9, 124.0, 122.0, 121.0, 119.7, 88.0, 85.7, 67.1, 21.1; **IR (cm^{-1})**: *v* 3019, 2928, 2208, 1747, 1631, 1013, 994; **HRMS** (ESI) m/z calculated for $C_{20}H_{16}ClNNaO_2$ [M + Na]$^+$: 360.0762, found: 360.0767.

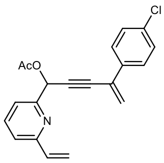

1m

2-(1-((tert-butyldimethylsilyl)oxy)-4-methylpent-4-en-2-yn-1-yl)-6-vinylpyridine

Compound **1n** was obtained as a yellow liquid; **^{1}H NMR (500 MHz, CDCl$_3$)** δ 7.59 (t, *J* = 7.5 Hz, 1H), 7.45 (d, *J* = 7.5 Hz, 1H), 7.17 (d, *J* = 7.5 Hz, 1H), 6.75 (dd, J_1 = 17.0 Hz, J_2 = 11.0 Hz, 1H), 6.13 (d, *J* = 17.0 Hz, 1H), 5.59 (s, 1H), 5.39 (d, *J* = 11.0 Hz, 1H), 5.21 (s, 1H), 5.13 (s, 1H), 1.80 (s, 3H), 0.88 (s, 9H), 0.16 (s, 3H), 0.09 (s, 3H); **^{13}C NMR (126 MHz, CDCl$_3$)** δ 160.4, 154.7, 137.2, 136.9, 126.4, 121.9, 119.8, 118.9, 118.2, 88.6, 86.7, 67.1, 25.8, 23.2, 18.3, -4.6, -4.9; **IR (cm^{-1})**: *v* 3097, 2956, 2857, 2225, 1615, 1454, 1361, 1088, 990, 778; **HRMS** (ESI) m/z calculated for $C_{19}H_{28}NOSi$ [M + H]$^+$: 314.1935, found: 314.1926.

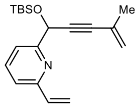

1n

(*E*)-4-methyl-1-(6-styrylpyridin-2-yl)pent-4-en-2-yn-1-yl acetate

Compound **1o** was obtained as a yellow liquid; **^{1}H NMR (400 MHz, CDCl$_3$)** δ 7.49 (t, *J* = 7.6 Hz, 1H), 7.38 (d, *J* = 7.6 Hz, 1H), 7.30 – 7.21 (m, 5H), 7.13 (d, *J* = 7.6 Hz, 1H), 6.84 (d, *J* = 12.4 Hz, 1H), 6.69 (d, *J* = 12.4 Hz, 1H), 6.54 (s, 1H), 5.37 (s, 1H), 5.28 (s, 1H), 2.14 (s, 3H), 1.89 (s, 3H); **^{13}C NMR (101 MHz, CDCl$_3$)** δ 169.5, 156.1, 155.7, 136.6, 136.5, 133.7, 130.1, 128.9, 128.2, 127.6, 125.9, 123.6, 123.3, 119.7, 88.4, 83.9, 67.2, 23.1, 21.0; **IR (cm^{-1})**: *v* 3022, 2922, 2855, 2200, 1748, 1613, 1019, 971, 751; **HRMS** (ESI) m/z calculated for $C_{21}H_{20}NO_2$ [M + H]$^+$: 318.1489, found: 318.1482.

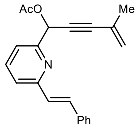

1o

(*E*)-5-phenyl-1-(6-((*E*)-styryl)pyridin-2-yl)pent-4-en-2-yn-1-yl acetate

Compound **1p** was obtained as a yellow liquid; **^{1}H NMR (400 MHz, CDCl$_3$)** δ 7.51 (t, *J* = 8.0 Hz, 1H), 7.42 (d, *J* = 8.0 Hz, 1H), 7.38 – 7.23 (m, 10H), 7.15 (d, *J* = 8.0 Hz, 1H), 7.02 (d, *J* = 16.0 Hz, 1H), 6.86 (d, *J* = 12.4 Hz, 1H), 6.71 (d, *J* = 12.4 Hz, 1H), 6.60 (d, *J* = 2.0 Hz, 1H), 6.19 (dd, J_1 = 16.0 Hz, J_2 = 2.0 Hz, 1H), 2.16 (m, 3H); **^{13}C NMR (101 MHz, CDCl$_3$)** δ 169.5, 156.2, 155.7, 142.8, 136.6, 136.5, 135.9, 133.8, 130.1, 128.9, 128.9, 128.7, 128.2, 127.6, 126.3, 123.7, 119.7, 107.0, 87.0, 86.6, 67.4, 21.0; **IR (cm^{-1})**: *v* 3025, 2929, 2841, 2214, 1744, 1637, 1026, 957, 749; **HRMS** (ESI) m/z calculated for $C_{26}H_{22}NO_2$ [M + H]$^+$: 380.1645, found: 380.1637.

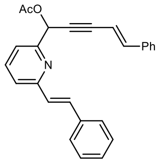

1p

(E)-1-(6-((E)-4-methoxystyryl)pyridin-2-yl)-5-phenylpent-4-en-2-yn-1-yl acetate

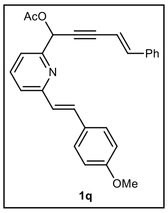

Compound **1q** was obtained as a yellow liquid; **^{1}H NMR (400 MHz, CDCl$_3$)** δ 7.54 (t, J = 8.0 Hz, 1H), 7.42 (d, J = 8.0 Hz, 1H), 7.38 – 7.28 (m, 7H), 7.21 (d, J = 8.0 Hz, 1H), 7.02 (d, J = 16.4 Hz, 1H), 6.80 – 6.76 (m, 3H), 6.62 – 6.51 (m, 2H), 6.20 (dd, J_1 = 17.6 Hz, J_2 = 2.0 Hz, 1H), 3.78 (s, 3H), 2.17 (s, 3H); **^{13}C NMR (101 MHz, CDCl$_3$)** δ 169.6, 159.1, 156.6, 155.6, 142.8, 136.7, 135.8, 133.4, 130.5, 128.9, 128.7, 128.4, 126.3, 123.7, 119.5, 113.6, 107.0, 87.0, 86.5, 67.5, 55.1, 21.1; **IR (cm^{-1})**: v 3027, 2933, 2856, 2210, 1744, 1605, 1030, 958, 833; **HRMS** (ESI) m/z calculated for $C_{25}H_{22}NO_3$ [M + H]$^+$: 384.1594, found: 384.1593.

(E)-1-(6-((E)-4-methylstyryl)pyridin-2-yl)-5-phenylpent-4-en-2-yn-1-yl acetate

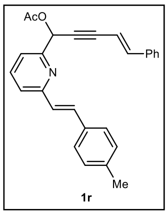

Compound **1r** was obtained as a yellow liquid; **^{1}H NMR (400 MHz, CDCl$_3$)** δ 7.78 (t, J = 8.0 Hz, 1H), 7.76 (d, J = 8.0 Hz, 1H), 7.66 – 7.52 (m, 5H), 7.49 – 7.45 (m, 3H), 7.35 – 7.30 (m, 3H), 7.08 (d, J = 12.4 Hz, 1H), 6.92 (d, J = 12.4 Hz, 1H), 6.87 (d, J = 1.6 Hz, 1H), 6.46 (dd, J_1 = 16.0 Hz, J_2 = 1.6 Hz, 1H), 2.58 (s, 3H), 2.43 (s, 3H); **^{13}C NMR (101 MHz, CDCl$_3$)** δ 169.6, 156.5, 155.7, 142.8, 137.6, 136.6, 135.9, 133.8, 133.6, 129.4, 129.0, 128.9, 128.9, 128.7, 126.4, 123.7, 119.6, 107.1, 87.0, 86.6, 67.5, 21.2, 21.1; **IR (cm^{-1})**: v 3024, 2922, 2851, 2218, 1745, 1616, 1013, 980, 749; **HRMS** (ESI) m/z calculated for $C_{27}H_{24}NO_2$ [M + H]$^+$: 394.1802, found: 394.1801.

(E)-1-(6-((E)-4-bromostyryl)pyridin-2-yl)-5-phenylpent-4-en-2-yn-1-yl acetate

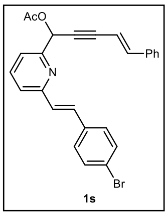

Compound **1s** was obtained as a yellow liquid; **^{1}H NMR (400 MHz, CDCl$_3$)** δ 7.57 (t, J = 8.0 Hz, 1H), 7.43 (d, J = 8.0 Hz, 1H), 7.39 – 7.29 (m, 7H), 7.23 (s, 1H), 7.21 (s, 1H), 7.14 (d, J = 8.0 Hz, 1H), 7.02 (d, J = 16.0 Hz, 1H), 6.75 (d, J = 12.8 Hz, 1H), 6.71 (d, J = 12.8 Hz, 1H), 6.59 (d, J = 2.0 Hz, 1H), 6.19 (dd, J_1 = 16.0 Hz, J_2 = 2.0 Hz, 1H), 2.16 (s, 3H); **^{13}C NMR (101 MHz, CDCl$_3$)** δ 169.6, 155.9, 155.8, 142.9, 136.9, 135.9, 135.4, 132.5, 131.4, 130.8, 130.6, 128.9, 128.7, 126.4, 123.7, 121.7, 119.9, 107.0, 86.9, 86.6, 67.4, 21.0; **IR (cm^{-1})**: v 3017, 2924, 2856, 2215, 1744, 1623, 1010, 956, 748; **HRMS** (ESI) m/z calculated for $C_{26}H_{21}BrNO_2$ [M + H]$^+$: 458.0750, found: 458.0755.

2-((E)-1-((tert-butyldimethylsilyl)oxy)-5-phenylpent-4-en-2-yn-1-yl)-6-((E)-2-(pyridin-2-yl)vinyl)pyridine

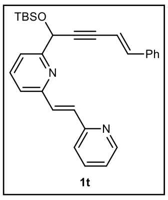

Compound **1t** was obtained as a yellow liquid; **^{1}H NMR (500 MHz, CDCl$_3$)** δ 8.52 (d, J = 3.0 Hz, 1H), 7.66 (m, 3H), 7.55 (dd, J_1 = 15.0 Hz, J_2 = 7.5 Hz, 1H), 7.48 (d, J = 7.5 Hz, 1H), 7.34 (d, J = 7.5 Hz, 1H), 7.27 – 7.24 (m, 3H), 7.20 – 7.12 (m, 3H), 7.04 (t, J = 6.0 Hz, 1H), 6.84 (d, J = 16.0 Hz, 1H), 6.09 (d, J = 16.0 Hz, 1H), 5.67 (s, 1H), 0.88 (s, 9H), 0.17 (s, 3H), 0.10 (s, 3H); **^{13}C NMR (126 MHz, CDCl$_3$)** δ 160.7, 155.0, 153.9, 149.6, 141.4, 137.4, 136.5, 136.0, 132.0, 131.7, 128.6, 128.5, 126.1, 122.8, 122.4, 121.9, 119.2, 107.7, 91.8, 84.8, 67.4, 25.8, 18.3, -4.5, -4.9; **IR (cm^{-1})**: v 3016, 2928, 2856, 2217, 1630, 1450, 1328, 1077, 954, 746; **HRMS** (ESI) m/z calculated for $C_{29}H_{33}N_2OSi$ [M + H]$^+$: 453.2357, found: 453.2352.

(*E*)-5-phenyl-1-(6-((1*E*,3*E*)-4-phenylbuta-1,3-dien-1-yl)pyridin-2-yl)pent-4-en-2-yn-1-yl acetate

Compound **1u** was obtained as a yellow liquid; **^{1}H NMR (400 MHz, CDCl$_3$)** δ 7.51 (t, *J* = 7.6 Hz, 1H), 7.35 – 7.26 (m, 4H), 7.22 – 7.07 (m, 9H), 6.89 – 6.79 (m, 2H), 6.63 (d, *J* = 15.6 Hz, 1H), 6.58 (d, *J* = 16.4 Hz, 1H), 6.50 (s, 1H), 6.04 (dd, J_1 = 16.4 Hz, J_2 = 2.0 Hz, 1H), 2.02 (s, 3H); **^{13}C NMR (101 MHz, CDCl$_3$)** δ 169.6, 155.8, 155.5, 142.8, 137.3, 136.9, 135.8, 135.5, 133.9, 131.5, 128.8, 128.7, 128.6, 128.3, 127.9, 126.6, 126.3, 121.4, 119.7, 107.0, 87.0, 86.5, 67.6, 21.0; **IR (cm^{-1})**: *v* 3020, 2924, 2849, 2214, 1743, 1618, 1025, 957, 749; **HRMS** (ESI) m/z calculated for $C_{28}H_{24}NO_2$ [M + H]$^+$: 406.1802, found: 406.1795.

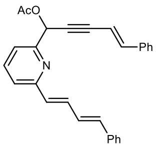

1u

(*E*)-1-(6-((*E*)-2-cyclohexylvinyl)pyridin-2-yl)-5-phenylpent-4-en-2-yn-1-yl acetate

Compound **1v** was obtained as a yellow liquid; **^{1}H NMR (400 MHz, CDCl$_3$)** δ 7.55 (t, *J* = 8.0 Hz, 1H), 7.31 (d, *J* = 8.0 Hz, 1H), 7.25 – 7.13 (m, 5H), 7.03 (d, *J* = 8.0 Hz, 1H), 6.90 (d, *J* = 16.4 Hz, 1H), 6.55 (s, 1H), 6.22 (d, *J* = 12.0 Hz, 1H), 6.08 (dd, J_1 = 16.4 Hz, J_2 = 2.0 Hz, 1H), 5.63 (dd, J_1 = 12.0 Hz, J_2 = 9.6 Hz, 1H), 3.22 – 3.14 (m, 1H), 2.05 (s, 3H), 1.75 – 1.72 (m, 2H), 1.64 – 1.55 (m, 3H), 1.31 – 1.22 (m, 2H), 1.16 – 0.99 (m, 3H); **^{13}C NMR (101 MHz, CDCl$_3$)** δ 169.4, 156.6, 155.2, 144.2, 142.6, 136.9, 135.7, 128.7, 128.6, 126.2, 125.6, 123.4, 118.6, 107.0, 87.2, 86.1, 67.4, 37.0, 32.6, 26.0, 25.6, 20.9; **IR (cm^{-1})**: *v* 3009, 2923, 2848, 2215, 1745, 1640, 1026, 957, 748; **HRMS** (ESI) m/z calculated for $C_{26}H_{28}NO_2$ [M + H]$^+$: 386.2115, found: 386.2109.

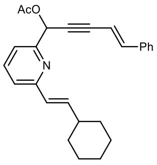

1v

(*E*)-5-phenyl-1-(6-((*E*)-prop-1-en-1-yl)pyridin-2-yl)pent-4-en-2-yn-1-yl acetate

Compound **1w** was obtained as a yellow liquid; **^{1}H NMR (400 MHz, CDCl$_3$)** δ 7.66 (t, *J* = 7.6 Hz, 1H), 7.41 (d, *J* = 7.6 Hz, 1H), 7.38 – 7.28 (m, 5H), 7.22 (d, *J* = 7.6 Hz, 1H), 7.01 (d, *J* = 16.0 Hz, 1H), 6.83 – 6.74 (m, 1H), 6.55 (d, *J* = 2.0 Hz, 1H), 6.53 (dd, J_1 = 16.0 Hz, J_2 = 2.0 Hz, 1H), 6.19 (dd, J_1 = 16.0 Hz, J_2 = 2.0 Hz, 1H), 2.17 (s, 3H), 1.93 (dd, J_1 = 6.8 Hz, J_2 = 2.0 Hz, 1H); **^{13}C NMR (101 MHz, CDCl$_3$)** δ 169.6, 156.0, 155.5, 142.7, 137.3, 135.9, 131.6, 130.9, 128.8, 128.7, 126.3, 120.4, 119.4, 107.0, 87.1, 86.4, 67.6, 21.0, 18.3; **IR (cm^{-1})**: *v* 3027, 2931, 2853, 2215, 1743, 1644, 1025, 957, 749; **HRMS** (ESI) m/z calculated for $C_{21}H_{20}NO_2$ [M + H]$^+$: 318.1489, found: 318.1481.

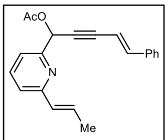

1w

2-(1-((tert-butyldimethylsilyl)oxy)-3-(cyclohex-1-en-1-yl)prop-2-yn-1-yl)-6-vinylpyridine

Compound **1x** was obtained as a yellow liquid; **^{1}H NMR (500 MHz, CDCl$_3$)** δ 7.55 (t, *J* = 7.5 Hz, 1H), 7.41 (d, *J* = 7.5 Hz, 1H), 7.13 (d, *J* = 7.5 Hz, 1H), 6.71 (dd, J_1 = 17.5 Hz, J_2 = 11.0 Hz, 1H), 6.08 (d, *J* = 17.5 Hz, 1H), 5.97 (s, 1H), 5.54 (s, 1H), 5.35 (d, *J* = 11.0 Hz, 1H), 1.99 – 1.94 (m, 4H), 1.49 – 1.43 (m, 4H), 0.84 (s, 9H), 0.11 (s, 3H), 0.04 (s, 3H); **^{13}C NMR (126 MHz, CDCl$_3$)** δ 160.8, 154.7, 137.2, 136.9, 134.8, 120.3, 119.7, 119.0, 118.1, 87.4, 86.9, 67.3, 28.9, 25.8, 25.5, 22.2, 21.4, 18.3, -4.5, -4.9; **IR (cm^{-1})**: *v* 3016, 2928, 2851, 2217, 1630, 1454, 1332, 1082, 991; **HRMS** (ESI) m/z calculated for $C_{22}H_{32}NOSi$ [M + H]$^+$: 354.2248, found: 354.2242.

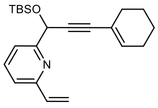

1x

(*E*)-3-(cyclohex-1-en-1-yl)-1-(6-styrylpyridin-2-yl)prop-2-yn-1-yl acetate

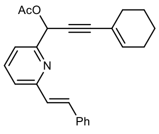

1y

Compound **1y** was obtained as a yellow liquid; **^{1}H NMR (400 MHz, $CDCl_3$)** δ 7.48 (t, *J* = 8.0 Hz, 1H), 7.39 (d, *J* = 8.0 Hz, 1H), 7.31 – 7.22 (m, 5H), 7.12 (d, *J* = 8.0 Hz, 1H), 6.83 (d, *J* = 12.4 Hz, 1H), 6.68 (d, *J* = 12.4 Hz, 1H), 6.54 (s, 1H), 6.20 – 6.17 (m, 1H), 2.15 – 2.06 (m, 7H), 1.64 – 1.53 (m, 4H); **^{13}C NMR (101 MHz, $CDCl_3$)** δ 169.5, 156.1, 156.0, 136.6, 136.5, 136.5, 133.6, 130.2, 128.9, 128.2, 127.6, 123.5, 119.8, 119.7, 89.2, 82.3, 67.4, 28.8, 25.6, 22.1, 21.3, 21.1; **IR (cm^{-1})**: *v* 3021, 2928, 2851, 2216, 1768, 1635, 1022, 912, 699; **HRMS** (ESI) m/z calculated for $C_{24}H_{24}NO_2$ $[M + H]^+$: 358.1802, found: 358.1793.

2-(1-((tert-butyldimethylsilyl)oxy)-3-(thiophen-2-yl)prop-2-yn-1-yl)-6-vinylpyridine

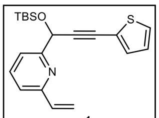

1z

Compound **1z** was obtained as a yellow liquid; **^{1}H NMR (500 MHz, $CDCl_3$)** δ 7.72 (t, *J* = 7.5 Hz, 1H), 7.59 (d, *J* = 7.5 Hz, 1H), 7.30 (d, *J* = 7.5 Hz, 1H), 7.24 (dd, J_1 = 5.0 Hz, J_2 = 1.0 Hz, 1H), 7.21 – 7.20 (m, 1H), 6.96 (dd, J_1 = 5.0 Hz, J_2 = 3.5 Hz, 1H), 6.86 (dd, J_1 = 17.5 Hz, J_2 = 11.0 Hz, 1H), 6.24 (d, *J* = 17.5 Hz, 1H), 5.80 (s, 1H), 5.51 (d, *J* = 11.0 Hz, 1H), 1.00 (s, 9H), 0.28 (s, 3H), 0.21 (s, 3H); **^{13}C NMR (126 MHz, $CDCl_3$),** δ 160.1, 154.3, 137.3, 136.8, 132.0, 127.0, 126.8, 122.8, 120.0, 119.0, 118.4, 93.4, 78.9, 67.3, 25.8, 18.3, -4.5, -4.9; **IR (cm^{-1})**: *v* 3016, 2929, 2856, 2221, 1613, 1454, 1360, 1084, 991; **HRMS** (ESI) m/z calculated for $C_{20}H_{26}NOSSi$ $[M + H]^+$: 356.1499, found: 356.1498.

2-(1-((tert-butyldimethylsilyl)oxy)-3-(thiophen-3-yl)prop-2-yn-1-yl)-6-vinylpyridine

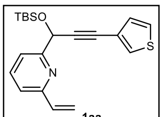

1aa

Compound **1aa** was obtained as a yellow liquid; **^{1}H NMR (500 MHz, $CDCl_3$)** δ 7.73 (t, *J* = 8.0 Hz, 1H), 7.63 (d, *J* = 8.0 Hz, 1H), 7.47(s, 1H), 7.31 (d, *J* = 8.0 Hz, 1H), 7.26 – 7.25 (m, 1H), 7.14 (d, *J* = 4.5 Hz, 1H), 6.89 (dd, J_1 = 17.5 Hz, J_2 = 11.0 Hz, 1H), 6.27 (d, *J* = 17.5 Hz, 1H), 5.83 (s, 1H), 5.53 (d, *J* = 4.5 Hz, 1H), 1.04 (s, 9H), 0.32 (s, 3H), 0.25 (s, 3H); **^{13}C NMR (126 MHz, $CDCl_3$)** δ 160.3, 154.8, 137.2, 136.8, 129.7, 128.7, 125.0, 121.8, 119.9, 118.9, 118.3, 89.2, 80.7, 67.2, 25. 8, 18.3, -4.5, -4.9; **IR (cm^{-1})**: *v* 3108, 2928, 2856, 2226, 1620, 1360, 1083, 930; **HRMS** (ESI) m/z calculated for $C_{20}H_{26}NOSSi$ $[M + H]^+$: 356.1499, found: 356.1497.

2-(3-(benzofuran-2-yl)-1-((tert-butyldimethylsilyl)oxy)prop-2-yn-1-yl)-6-vinylpyridine

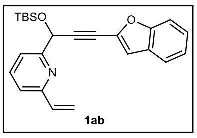

1ab

Compound **1ab** was obtained as a yellow liquid; **^{1}H NMR (400 MHz, $CDCl_3$)** δ 7.72 (t, *J* = 7.6 Hz, 1H), 7.61 (d, *J* = 7.6 Hz, 1H), 7.54 (d, *J* = 7.6 Hz, 1H), 7.49 (d, *J* = 7.6 Hz, 1H), 7.34 – 7.29 (m, 2H), 7.25 – 7.21 (m, 1H), 6.94 (s, 1H), 6.86 (dd, J_1 = 17.6 Hz, J_2 = 10.8 Hz, 1H), 6.26 (dd, J_1 = 17.6 Hz, J_2 = 1.2 Hz, 1H), 5.85 (s, 1H), 5.51 (dd, J_1 = 10.8 Hz, J_2 = 1.2 Hz, 1H), 1.01 (s, 9H), 0.30 (s, 3H), 0.22 (s, 3H); **^{13}C NMR (101 MHz, $CDCl_3$)** δ 159.5, 154.9, 154.7, 138.3, 137.4, 136.7, 127.4, 125.5, 123.1, 121.1, 120.2, 119.0, 118.5, 111.8, 111.1, 95.5, 75.9, 67.2, 25.8, 18.3, -4.6, -5.0; **IR (cm^{-1})**: *v* 3060, 2929, 2856, 2213, 1670, 1451, 1347, 1109, 991, 749; **HRMS** (ESI) m/z calculated for $C_{24}H_{28}NO_2Si$ $[M + H]^+$: 390.1884, found: 390.1882.

2-(3-((tert-butyldimethylsilyl)oxy)-3-(6-vinylpyridin-2-yl)prop-1-yn-1-yl)-1-methyl-1H-indole

Compound **1ac** was obtained as a yellow liquid; **^{1}H NMR (500 MHz, $CDCl_3$)** δ 7.73 (t, *J* = 8.0 Hz, 1H), 7.61 (d, *J* = 8.0 Hz, 1H), 7.57 (d, *J* = 8.0 Hz, 1H), 7.30 (d, *J* = 8.0 Hz, 1H), 7.25 – 7.24 (m, 2H), 7.12 – 7.09 (m, 1H), 6.85 (dd, J_1 = 17.5 Hz, J_2 = 11.0 Hz, 1H), 6.75 (s, 1H), 6.25 (d, *J* = 17.5 Hz, 1H), 5.87 (s, 1H), 5.50 (d, *J* = 11.0 Hz, 1H), 3.76 (s, 3H), 1.00 (s, 9H), 0.29 (s, 3H), 0.22 (s, 3H); **^{13}C NMR (126 MHz, $CDCl_3$)** δ 160.1, 154.9, 137.4, 137.1, 136.8, 127.1, 122.9, 121.7, 120.9, 120.1, 120.0, 118.9, 118.4, 109.3, 107.3, 95.7, 77.4, 67.4, 30.5, 25.8, 18.4, -4.5, -4.9; **IR (cm^{-1})**: *v* 3054, 2928, 2855, 2224, 1651, 1455, 1342, 1100, 991, 748; **HRMS** (ESI) m/z calculated for $C_{25}H_{31}N_2OSi$ [M + H]$^+$: 403.2200, found: 403.2209.

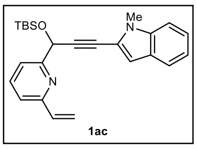

1ac

(*E*)-2-(1-((tert-butyldimethylsilyl)oxy)-5-phenylpent-4-en-2-yn-1-yl)-6-phenylpyridine

Compound **1ad** was obtained as a yellow liquid; **^{1}H NMR (400 MHz, $CDCl_3$)** δ 8.12 – 8.10 (m, 2H), 7.85 (t, *J* = 8.0 Hz, 1H), 7.71 – 7.69 (m, 2H), 7.55 – 7.29 (m, 8H), 7.01 (d, *J* = 16.0 Hz, 1H), 6.27 (dd, J_1 = 16.0 Hz, J_2 = 1.6 Hz, 1H), 5.89 (s, 1H), 1.06 (s, 9H), 0.34 (s, 3H), 0.27 (s, 3H); **^{13}C NMR (101 MHz, $CDCl_3$)** δ 160.7, 156.3, 141.4, 139.2, 137.6, 136.1, 128.8, 128.6, 128.5, 127.0, 126.2, 119.4, 118.6, 107.9, 92.0, 84.7, 67.5, 25.8, 18.4, -4.5, -4.9; **IR (cm^{-1})**: *v* 3030, 2928, 2856, 2206, 1570, 1470, 1309, 1061, 953; **HRMS** (ESI) m/z calculated for $C_{28}H_{32}NOSi$ [M + H]$^+$: 426.2248, found: 426.2239.

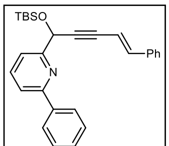

1ad

(*E*)-1,5-diphenyl-1-(6-vinylpyridin-2-yl)pent-4-en-2-yn-1-yl acetate

Compound **1ae** was obtained as a yellow liquid; **^{1}H NMR (400 MHz, $CDCl_3$)** δ 7.73 – 7.70 (m, 2H), 7.66 – 7.58 (m, 2H), 7.40 – 7.24 (m, 8H), 7.15 – 7.07 (m, 2H), 6.76 (ddd, J_1 = 17.2 Hz, J_2 = 10.8 Hz, J_3 = 1.6 Hz, 1H), 6.33 (dd, J_1 = 16.0 Hz, J_2 = 1.6 Hz, 1H), 6.20 (dt, J_1 = 17.2 Hz, J_2 = 1.6 Hz, 1H), 5.42 (dt, J_1 = 10.8 Hz, J_2 = 1.6 Hz, 1H), 2.22 (s, 3H); **^{13}C NMR (101 MHz, $CDCl_3$)** δ 168.7, 158.9, 154.8, 142.6, 141.1, 137.1, 136.8, 136.0, 128.8, 128.7, 128.1, 128.0, 126.5, 126.3, 119.8, 119.7, 118.2, 107.3, 89.7, 88.3, 80.0, 21.7; **IR (cm^{-1})**: *v* 3028, 2925, 2213, 1753, 1567, 1451, 1230, 957, 749; **HRMS** (ESI) m/z calculated for $C_{26}H_{22}NO_2$ [M + H]$^+$: 380.1645, found: 380.1648.

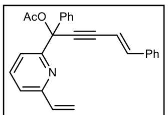

1ae

(*E*)-6-phenyl-2-(6-vinylpyridin-2-yl)hex-5-en-3-yn-2-yl acetate

Compound **1af** was obtained as a yellow liquid; **^{1}H NMR (400 MHz, $CDCl_3$)** δ 7.67 – 7.61 (m, 2H), 7.38 – 7.21 (m, 6H), 7.03 (d, *J* = 16.0 Hz, 1H), 6.80 (dd, J_1 = 17.6 Hz, J_2 = 10.8 Hz, 1H), 6.28 – 6.18 (m, 2H), 5.45 (dd, J_1 = 10.8 Hz, J_2 = 1.2 Hz, 1H), 2.09 (s, 3H), 1.99 (s, 3H); **^{13}C NMR (101 MHz, $CDCl_3$)** δ 168.9, 159.3, 154.9, 142.2, 136.9, 136.8, 136.0, 128.7, 128.6, 126.2, 119.9, 119.4, 118.2, 107.3, 90.6, 85.9, 76.1, 29.8, 21.5; **IR (cm^{-1})**: *v* 3028, 2935, 2214, 1747, 1569, 1454, 1240, 953, 749; **HRMS** (ESI) m/z calculated for $C_{21}H_{19}NNaO_2$ [M + Na]$^+$: 340.1308, found: 340.1308.

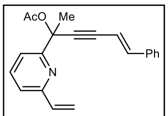

1af

(E)-2-(1-((tert-butyldimethylsilyl)oxy)-5-(4-methoxyphenyl)pent-4-en-2-yn-1-yl-1-d)-6-vinylpyridine

Compound **1a'** was obtained as a yellow liquid; **^{1}H NMR (400 MHz, $CDCl_3$)** δ 7.51 (t, J = 7.6 Hz, 1H), 7.38 (d, J = 7.6 Hz, 1H), 7.12 – 7.08 (m, 3H), 6.72 – 6.63 (m, 4H), 6.03 (d, J = 16.0 Hz, 1H), 5.86 (d, J = 16.0 Hz, 1H), 5.30 (dd, J_1 = 10.8 Hz, J_2 = 0.8 Hz, 1H), 3.60 (s, 3H), 0.79 (s, 9H), 0.07 (s, 3H), 0.00 (s, 3H); **^{13}C NMR (101 MHz, $CDCl_3$)** δ 160.5, 159.9, 154.8, 140.9, 137.3, 136.9, 129.0, 127.5, 119.8, 119.0, 118.2, 114.0, 105.4, 90.9, 85.1, 55.2, 25.8, 18.3, -4.5, -4.9; **IR (cm^{-1})**: v 3033, 2929, 2856, 2208, 1605, 1454, 1250, 1082, 780; **HRMS** (ESI) m/z calculated for $C_{25}H_{31}DNO_2Si$ [M + H]$^+$: 407.2260, found: 407.2251.

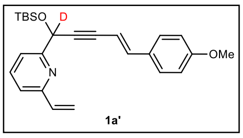

(E)-2-(2-phenyl-1-(4-phenylbut-3-en-1-yn-1-yl)cyclopropyl)-6-vinylpyridine

Compound **3a** was obtained as a colorless liquid; **^{1}H NMR (400 MHz, $CDCl_3$)** δ 7.74 (d, J = 7.6 Hz, 1H), 7.58 (d, J = 7.6 Hz, 1H), 7.34 – 7.28 (m, 10H), 7.11 (d, J = 7.6 Hz, 1H), 6.78 (dd, J_1 = 17.6 Hz, J_2 = 10.8 Hz, 1H), 6.64 (d, J = 16.4 Hz, 1H), 6.26 (d, J = 17.6 Hz, 1H), 6.06 (d, J = 16.4 Hz, 1H), 5.44 (d, J = 10.8 Hz, 1H), 3.18 (t, J = 7.6 Hz, 1H), 2.46 (dd, J_1 = 8.8 Hz, J_2 = 4.0 Hz, 1H), 1.93 (dd, J_1 = 7.6 Hz, J_2 = 4.0 Hz, 1H); **^{13}C NMR (101 MHz, $CDCl_3$)** δ 158.8, 154.7, 140.5, 137.8, 137.0, 136.5, 136.4, 128.6, 128.6, 128.3, 127.8, 126.4, 126.0, 120.3, 118.7, 117.8, 108.3, 92.0, 83.0, 38.0, 28.2, 25.6; **IR (cm^{-1})**: v 3051, 2925, 2850, 2206, 1570, 1237, 952, 696; **HRMS** (ESI) m/z calculated for $C_{26}H_{22}N$ [M + H]$^+$: 348.1747, found: 348.1754.

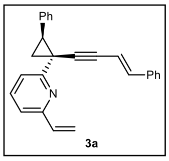

(E)-2-(2-phenyl-1-(4-(thiophen-3-yl)but-3-en-1-yn-1-yl)cyclopropyl)-6-vinylpyridine

Compound **3b** was obtained as a colorless liquid; **^{1}H NMR (400 MHz, $CDCl_3$)** δ 7.73 (d, J = 7.6 Hz, 1H), 7.57 (t, J = 7.6 Hz, 1H), 7.33 – 7.32 (m, 4H), 7.26 – 7.22 (m, 2H), 7.13 – 7.09 (m, 3H), 6.78 (dd, J_1= 17.2 Hz, J_2 = 10.8 Hz, 1H), 6.65 (d, J = 16.0 Hz, 1H), 6.25 (d, J = 17.2 Hz, 1H), 5.90 (d, J = 16.0 Hz, 1H), 5.43 (d, J = 10.8 Hz, 1H), 3.19 – 3.15 (m, 1H), 2.45 (ddd, J_1 = 8.8 Hz, J_2 = 4.0 Hz, J_3 = 1.6 Hz, 1H), 1.92 (ddd, J_1 = 7.6 Hz, J_2 = 4.0 Hz, J_3 = 1.6 Hz, 1H); **^{13}C NMR (101 MHz, $CDCl_3$)** δ 158.9, 154.6, 139.3, 137.8, 137.0, 134.6, 128.6, 127.7, 126.4, 126.3, 124.3, 122.9, 120.3, 118.7, 117.8, 108.1, 91.8, 82.9, 77.3, 77.0, 76.7, 37.9, 28.1, 25.6; **IR (cm^{-1})**: v 3029, 2931, 2855, 2209, 1570, 1274, 991, 697; **HRMS** (ESI) m/z calculated for $C_{24}H_{20}NS$ [M + H]$^+$: 354.1311, found: 354.1318.

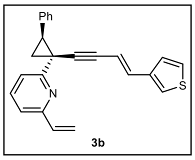

2-(2-phenyl-1-((3E,5E)-6-phenylhexa-3,5-dien-1-yn-1-yl)cyclopropyl)-6-vinylpyridine

Compound **3c** was obtained as a colorless liquid; **^{1}H NMR (400 MHz, $CDCl_3$)** δ 7.75 (d, J = 7.6 Hz, 1H), 7.60 (t, J = 7.6 Hz, 1H), 7.41 – 7.23 (m, 10H), 7.13 (d, J = 7.6 Hz, 1H), 6.85 – 6.73 (m, 2H), 6.57 – 6.47 (m, 2H), 6.29 (d, J = 17.2 Hz, 1H), 5.66 (d, J = 15.2 Hz, 1H), 5.47 (d, J = 10.8 Hz, 1H), 3.21 (t, J = 7.6 Hz, 1H), 2.49 (ddd, J_1 = 5.2 Hz, J_2 = 4.0 Hz, J_3 = 1.2 Hz, 1H), 1.96 (ddd, J_1 = 5.2 Hz, J_2 = 4.0 Hz, J_3 = 1.2 Hz, 1H); **^{13}C NMR (101 MHz, $CDCl_3$)** δ 158.8, 154.6, 141.1, 137.8, 136.9, 136.8, 136.5, 133.7, 128.6, 128.5, 128.1, 127.9, 127.7, 126.5, 126.4, 120.3, 118.8, 117.8, 111.7, 93.2, 83.4, 38.1, 28.2, 25.7; **IR (cm^{-1})**: v 3025, 2925, 2855, 2207, 1631, 1271, 983, 692; **HRMS** (ESI) m/z calculated for $C_{28}H_{24}N$ [M + H]$^+$: 374.1903, found: 374.1900.

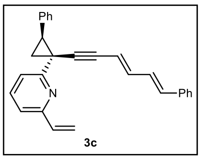

(*E*)-2-(2-phenyl-1-(undec-3-en-1-yn-1-yl)cyclopropyl)-6-vinylpyridine

Compound **3d** was obtained as a colorless liquid; **^{1}H NMR (400 MHz, $CDCl_3$)** δ 7.72 (d, *J* = 7.6 Hz, 1H), 7.57 (t, *J* = 7.6 Hz, 1H), 7.34 – 7.30 (m, 4H), 7.24 – 7.21 (m, 1H), 7.10 (d, *J* = 7.6 Hz, 1H), 6.78 (dd, J_1 = 17.6 Hz, J_2 = 10.8 Hz, 1H), 6.25 (d, *J* = 17.6 Hz, 1H), 5.91 – 5.84 (m, 1H), 5.45 – 5.36 (m, 2H), 3.13 (t, *J* = 7.6 Hz, 1H), 2.41 (dd, J_1 = 8.8 Hz, J_2 = 4.0 Hz, 1H), 2.03 (dd, J_1 = 14.0 Hz, J_2 = 6.8 Hz, 2H), 1.87 (dd, J_1 = 7.6 Hz, J_2 = 4.0 Hz, 1H), 1.35 – 1.27 (m, 10H), 0.89 (t, *J* = 6.8 Hz, 3H); **^{13}C NMR (101 MHz, $CDCl_3$)** δ 159.1, 154.6, 144.1, 138.0, 137.0, 136.4, 128.6, 127.6, 126.3, 120.3, 118.6, 117.7, 109.3, 87.5, 82.6, 37.7, 32.9, 31.8, 29.1, 29.0, 28.8, 27.9, 25.5, 22.6, 14.1; **IR (cm^{-1})**: *v* 3034, 2925, 2854, 2205, 1570, 1233, 954, 696; **HRMS** (ESI) m/z calculated for $C_{27}H_{32}N$ $[M + H]^+$: 370.2529, found: 370.2538.

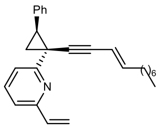

3d

(*E*)-2-(1-(4-cyclohexylbut-3-en-1-yn-1-yl)-2-phenylcyclopropyl)-6-vinylpyridine

Compound **3e** was obtained as a colorless liquid; **^{1}H NMR (400 MHz, $CDCl_3$)** δ 7.72 (d, *J* = 7.6 Hz, 1H), 7.56 (d, *J* = 7.6 Hz, 1H), 7.33 – 7.29 (m, 4H), 7.25 – 7.21 (m, 1H), 7.09 (d, *J* = 7.6 Hz, 1H), 6.77 (dd, J_1 = 17.2 Hz, J_2 = 10.8 Hz, 1H), 6.25 (dd, J_1 = 17.2 Hz, J_2 = 1.2 Hz, 1H), 5.83 (dd, J_1 = 16.0 Hz, J_2 = 7.6 Hz, 1H), 5.43 (dd, J_1 = 10.8 Hz, J_2 = 1.2 Hz, 1H), 5.33 (dd, J_1 = 16.0 Hz, J_2 = 1.2 Hz, 1H), 3.12 (t, *J* = 7.6 Hz, 1H), 2.41 (dd, J_1 = 8.8 Hz, J_2 = 4.0 Hz, 1H), 1.97 – 1.93 (m, 1H), 1.86 (dd, J_1 = 7.6 Hz, J_2 = 4.0 Hz, 1H), 1.73 – 1.62 (m, 5H), 1.29 – 0.98 (m, 5H); **^{13}C NMR (101 MHz, $CDCl_3$)** δ 159.1, 154.5, 149.4, 137.9, 137.0, 136.4, 128.5, 127.6, 126.3, 120.3, 118.6, 117.7, 107.1, 87.8, 82.8, 41.1, 37.7, 32.3, 28.0, 26.0, 25.8, 25.5; **IR (cm^{-1})**: *v* 3028, 2924, 2850, 2208, 1605, 1273, 957, 696; **HRMS** (ESI) m/z calculated for $C_{26}H_{28}N$ $[M + H]^+$: 354.2216, found: 354.2226.

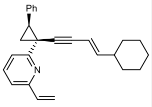

3e

2-(2-phenyl-1-(thiophen-2-ylethynyl)cyclopropyl)-6-vinylpyridine

Compound **3f** was obtained as a colorless liquid; **^{1}H NMR (400 MHz, $CDCl_3$)** δ 7.77 (d, *J* = 7.6 Hz, 1H), 7.62 (t, *J* = 7.6 Hz, 1H), 7.37 – 7.36 (m, 4H), 7.30 – 7.26 (m, 1H), 7.17 – 7.14 (m, 2H), 6.99 (dd, J_1 = 4.0 Hz, J_2 = 1.6 Hz, 1H), 6.91 (dd, J_1 = 5.2 Hz, J_2 = 4.0 Hz, 1H), 6.82 (dd, J_1 = 17.6 Hz, J_2 = 10.8 Hz, 1H), 6.30 (dd, J_1 = 17.6 Hz, J_2 = 1.6 Hz, 1H), 5.48 (dd, J_1 = 10.8 Hz, J_2 = 1.6 Hz, 1H), 3.25 (dd, J_1 = 8.8 Hz, J_2 = 7.6 Hz, 1H), 2.53 (dd, J_1 = 8.8 Hz, J_2 = 4.4 Hz, 1H), 2.02 (dd, J_1 = 7.6 Hz, J_2 = 4.4 Hz, 1H); **^{13}C NMR (101 MHz, $CDCl_3$)** δ 158.5, 154.7, 137.6, 136.9, 136.5, 131.4, 128.5, 127.8, 126.7, 126.5, 126.3, 123.6, 120.3, 118.8, 117.9, 93.6, 38.1, 28.0, 25.6; **IR (cm^{-1})**: *v* 3055, 2922, 2851, 2210, 1603, 1223, 991, 699; **HRMS** (ESI) m/z calculated for $C_{22}H_{18}NS$ $[M + H]^+$: 328.1154, found: 328.1153.

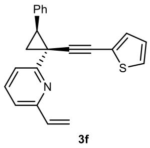

3f

2-(2-phenyl-1-(thiophen-3-ylethynyl)cyclopropyl)-6-vinylpyridine

Compound **3g** was obtained as a colorless liquid; **^{1}H NMR (400 MHz, CDCl$_3$)** δ 7.78 (d, J = 8.0 Hz, 1H), 7.58 (t, J = 8.0 Hz, 1H), 7.37 – 7.31 (m, 4H), 7.27 – 7.23 (m, 1H), 7.18 – 7.15 (m, 2H), 7.11 (d, J = 8.0 Hz, 1H), 6.86 (dd, J_1 = 4.8 Hz, J_2 = 1.6 Hz, 1H), 6.79 (dd, J_1 = 17.6 Hz, J_2 = 10.8 Hz, 1H), 6.27 (dd, J_1 = 17.6 Hz, J_2 = 1.6 Hz, 1H), 5.45 (dd, J_1 = 10.8 Hz, J_2 = 1.6 Hz, 1H), 3.20 (dd, J_1 = 8.8 Hz, J_2 = 7.6 Hz, 1H), 2.47 (dd, J_1 = 8.8 Hz, J_2 = 4.0 Hz, 1H), 1.97 (dd, J_1 = 7.6 Hz, J_2 = 4.0 Hz, 1H); **^{13}C NMR (101 MHz, CDCl$_3$)** δ 158.8, 154.6, 137.9, 137.0, 136.5, 129.9, 128.6, 127.9, 127.7, 126.4, 124.9, 122.5, 120.3, 118.8, 117.8, 89.0, 78.6, 37.8, 27.8, 25.3; **IR (cm^{-1})**: v 3058, 2923, 2851, 2229, 1602, 1272, 932, 697; **HRMS** (ESI) m/z calculated for $C_{22}H_{18}NS$ [M + H]$^+$: 328.1154, found: 328.1150.

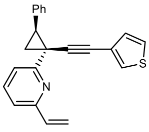

3g

1-methyl-2-((2-phenyl-1-(6-vinylpyridin-2-yl)cyclopropyl)ethynyl)-1H-indole

Compound **3h** was obtained as a colorless liquid; **^{1}H NMR (400 MHz, CDCl$_3$)** δ 7.81 (d, J = 7.6 Hz, 1H), 7.63 (t, J = 7.6 Hz, 1H), 7.54 (d, J = 7.6 Hz, 1H), 7.39 – 7.32 (m, 4H), 7.29 – 7.24 (m, 1H), 7.22 – 7.15 (m, 3H), 7.09 (t, J = 7.6 Hz, 1H), 6.82 (dd, J_1 = 17.2 Hz, J_2 = 10.8 Hz, 1H), 6.65 (s, 1H), 6.31 (d, J = 17.2 Hz, 1H), 5.48 (d, J = 10.8 Hz, 1H), 3.33 (t, J = 8.0 Hz, 1H), 3.27 (s, 3H), 2.53 (dd, J_1 = 8.0 Hz, J_2 = 4.4 Hz, 1H), 2.09 (dd, J_1 = 7.6 Hz, J_2 = 4.4 Hz, 1H); **^{13}C NMR (101 MHz, CDCl$_3$)** δ 158.5, 154.7, 137.6, 136.9, 136.8, 136.6, 128.8, 128.0, 127.0, 126.7, 122.6, 122.2, 120.6, 120.3, 119.8, 118.9, 118.0, 109.2, 106.8, 95.6, 74.9, 37.9, 29.9, 27.9, 25.2; **IR (cm^{-1})**: v 3057, 2925, 2851, 2215, 1629, 1265, 991, 693; **HRMS** (ESI) m/z calculated for $C_{27}H_{23}N_2$ [M + H]$^+$: 375.1856, found: 375.1855.

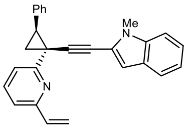

3h

(E)-2-(1-(4-phenylbut-3-en-1-yn-1-yl)-2-(p-tolyl)cyclopropyl)-6-vinylpyridine

Compound **3i** was obtained as a colorless liquid; **^{1}H NMR (400 MHz, CDCl$_3$)** δ 7.76 (d, J = 8.0 Hz, 1H), 7.60 (t, J = 8.0 Hz, 1H), 7.34 – 7.29 (m, 4H), 7.27 – 7.23 (m, 3H), 7.17 – 7.11 (m, 3H), 6.80 (dd, J_1 = 17.2 Hz, J_2 = 10.8 Hz, 1H), 6.68 (d, J = 16.0 Hz, 1H), 6.27 (dd, J_1 = 17.2 Hz, J_2 = 1.2 Hz, 1H), 6.10 (d, J = 16.0 Hz, 1H), 5.45 (dd, J_1 = 10.8 Hz, J_2 = 1.2 Hz, 1H), 3.15 (t, J = 8.0 Hz, 1H), 2.46 (dd, J_1 = 8.8 Hz, J_2 = 4.4 Hz, 1H), 2.37 (s, 3H), 1.92 (dd, J_1 = 8.0 Hz, J_2 = 4.4 Hz, 1H); **^{13}C NMR (101 MHz, CDCl$_3$)** δ 158.9, 154.6, 140.4, 137.0, 136.5, 136.4, 135.9, 134.7, 128.6, 128.5, 128.4, 128.3, 126.0, 120.3, 118.7, 117.8, 108.4, 92.2, 83.0, 37.9, 28.1, 25.7, 21.1; **IR (cm^{-1})**: v 3025, 2922, 2851, 2200, 1570, 1275, 747; **HRMS** (ESI) m/z calculated for $C_{27}H_{24}N$ [M + H]$^+$: 362.1903, found: 362.1911.

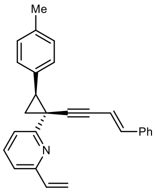

3i

(*E*)-2-(2-(4-fluorophenyl)-1-(4-phenylbut-3-en-1-yn-1-yl)cyclopropyl)-6-vinylpyridine

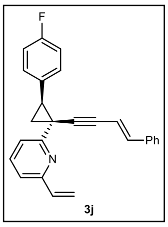

Compound **3j** was obtained as a colorless liquid; **^{1}H NMR (400 MHz, CDCl$_3$)** δ 7.78 (dd, J_1 = 8.0 Hz, J_2 = 2.4 Hz, 1H), 7.6 (td, J_1 = 8.0 Hz, J_2 = 1.2 Hz, 1H), 7.35 – 7.26 (m, 7H), 7.14 (d, J = 8.0 Hz, 1H), 7.08 – 7.03 (m, 2H), 6.81(ddd, J_1 = 17.2 Hz, J_2 = 10.8 Hz, J_3 = 2.0 Hz, 1H), 6.70 (dd, J_1 = 16.0 Hz, J_2 = 2.4 Hz, 1H), 6.28 (dt, J_1 = 17.2 Hz, J_2 = 2.0 Hz, 1H), 6.10 (dd, J_1 = 16.0 Hz, J_2 = 2.0 Hz, 1H), 5.47 (dt, J_1 = 10.8 Hz, J_2 = 2.0 Hz, 1H), 3.22 – 3.18 (m, 1H), 2.49 – 2.45 (m, 1H), 1.96 – 1.89 (m, 1H); **^{13}C NMR (101 MHz, CDCl$_3$)** δ 162.9, 160.5, 158.6, 154.6, 140.7, 136.7 (d, J = 38.7 Hz), 136.3, 133.6 (d, J = 3.3 Hz), 130. 0 (d, J = 8.0 Hz), 128.6, 128.4, 126.0, 120.3, 118.8, 117.9, 114.6 (d, J = 21.2 Hz), 108.1, 91.7, 83.1, 37.0, 27.9, 25.8; **^{19}F NMR (377 MHz, CDCl$_3$)** δ -116.44 (s, 1F); **IR (cm^{-1}):** *v* 3056, 2926, 2849, 2208, 1604, 1228, 952, 747; **HRMS** (ESI) m/z calculated for $C_{26}H_{21}FN$ [M + H]$^+$: 366.1653, found: 366.1655.

2-((tert-butyldimethylsilyl)oxy)-8-(4-methoxyphenyl)-6,7-dihydroazepino[2,1,7-cd]indolizine

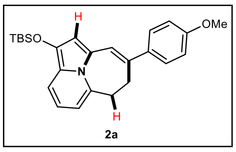

According to the procedure A, compound **2a** was obtained as a yellow amorphous solid in 96% yield; **^{1}H NMR (400 MHz, CDCl$_3$)** δ 7.41 – 7.37 (m, 2H), 7.30 (d, J = 8.8 Hz, 1H), 6.94 – 6.90 (m, 2H), 6.77 (s, 1H), 6.54 (dd, J_1 = 8.8 Hz, J_2 = 6.8 Hz, 1H), 6.45 (s, 1H), 6.24 (d, J = 6.8 Hz, 1H), 3.85 (s, 3H), 3.18 (t, J = 4.8 Hz, 2H), 3.07 (t, J = 4.8 Hz, 2H), 1.10 (s, 9H), 0.27 (s, 6H); **^{13}C NMR (101 MHz, CDCl$_3$)** δ 158.1, 138.3, 136.3, 133.5, 126.4, 126.1, 120.8, 117.8, 114.8, 114.8, 113.7, 110.3, 108.2, 55.3, 34.5, 32.3, 25.8, 18.2, -4.6; **IR (cm^{-1})**: *v* 3023, 2929, 2856, 1618, 1433, 1395, 1252, 1037, 807; **HRMS** (ESI) m/z calculated for $C_{25}H_{32}NO_2Si$ [M + H]$^+$: 406.2197, found: 406.2199.

2-((tert-butyldimethylsilyl)oxy)-8-phenyl-6,7-dihydroazepino[2,1,7-cd]indolizine

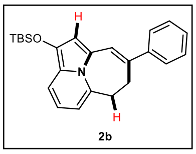

According to the procedure A, compound **2b** was obtained as a yellow thick liquid in 92% yield; **^{1}H NMR (400 MHz, CDCl$_3$)** δ 7.46 – 7.44 (m, 2H), 7.38 – 7.34 (m, 2H), 7.28 (d, J = 8.8 Hz, 1H), 7.22 (t, J = 7.2 Hz, 1H), 6.82 (s, 1H), 6.54 (dd, J_1 = 8.8 Hz, J_2 = 7.2 Hz, 1H), 6.44 (s, 1H), 6.25 (d, J = 7.2 Hz, 1H), 3.19 (t, J = 4.0 Hz, 2H), 3.11 (t, J = 4.0 Hz, 2H), 1.07 (s, 9H), 0.25 (s, 6H); **^{13}C NMR (101 MHz, CDCl$_3$)** δ 143.5, 138.5, 133.7, 133.6, 128.3, 126.5, 125.9, 125.3, 120.7, 119.3, 115.2, 114.8, 110.5, 108.6, 34.5, 32.2, 25.8, 18.2, -4.6; **IR (cm^{-1})**: *v* 3021, 2928, 2851, 1617, 1447, 1361, 1254, 1046, 741; **HRMS** (ESI) m/z calculated for $C_{24}H_{30}NOSi$ [M + H]$^+$: 376.2091, found: 376.2096.

8-phenyl-6,7-dihydroazepino[2,1,7-cd]indolizin-2-yl acetate

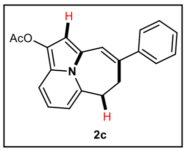

According to the procedure A, compound **2c** was obtained as a yellow thick liquid in 90% yield; **^{1}H NMR (500 MHz, CDCl$_3$)** δ 7.49 – 7.48 (m, 2H), 7.43 – 7.40 (m, 2H), 7.31 – 7.29 (m, 2H), 6.94 (s, 1H), 6.91 (s, 1H), 6.72 (t, J = 7.5 Hz, 1H), 6.35 (d, J = 7.5 Hz, 1H), 3.20 (t, J = 5.0 Hz, 2H), 3.10 (t, J = 5.0 Hz, 2H), 2.41 (s, 3H); **^{13}C NMR (126 MHz, CDCl$_3$)** δ 168.6, 143.0, 138.7, 134.9, 128.1, 127.8, 126.1, 126.0, 125.2, 121.6, 119.0, 117.4, 113.7, 110.7, 110.3, 34.2, 31.9, 20.6; **IR (cm^{-1})**: *v* 3051, 2988, 2856, 1764, 1620, 1201, 1011, 761; **HRMS** (ESI) m/z calculated for $C_{20}H_{18}NO_2$ [M + H]$^+$: 304.1332, found: 304.1336.

2-((tert-butyldimethylsilyl)oxy)-8-(p-tolyl)-6,7-dihydroazepino[2,1,7-cd]indolizine

According to the procedure A, compound **2d** was obtained as a yellow thick liquid in 93% yield;

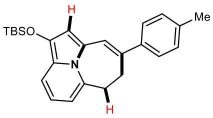

2d

^{1}H NMR (400 MHz, CDCl$_3$) δ 7.25 – 7.23 (m, 2H), 7.16 (d, *J* = 8.0 Hz, 1H), 7.07 – 7.05 (m, 2H), 6.68 (s, 1H), 6.42 (t, *J* = 6.8 Hz, 1H), 6.31 (s, 1H), 6.13 (d, *J* = 6.8 Hz, 1H), 3.07 (t, *J* = 4.0 Hz, 2H), 2.98 (t, *J* = 4.0 Hz, 2H), 2.26 (s, 3H), 0.95 (s, 9H), 0.13 (s, 6H); **^{13}C NMR (101 MHz, CDCl$_3$)** δ 140.7, 138.4, 135.6, 133.8, 133.6, 129.0, 126.3, 125.2, 120.8, 118.5, 115.0, 114.8, 110.4, 108.4, 34.5, 32.2, 25.8, 21.0, 18.2, -4.6; **IR (cm^{-1})**: *v* 3021, 2927, 2850, 1619, 1429, 1394, 1252, 1047, 716; **HRMS** (ESI) m/z calculated for $C_{25}H_{32}NOSi$ [M + H]$^+$: 390.2248, found: 390.2241.

8-(4-bromophenyl)-6,7-dihydroazepino[2,1,7-cd]indolizin-2-yl acetate

According to the procedure A, compound **2e** was obtained as a yellow thick liquid in 91% yield;

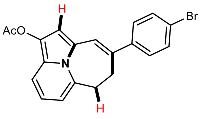

2e

^{1}H NMR (400 MHz, CDCl$_3$) δ 7.47 – 7.43 (m, 2H), 7.29 – 7.25 (m, 2H), 7.22 (d, *J* = 8.8 Hz, 1H), 6.85 (s, 1H), 6.80 (s, 1H), 6.70 (dd, J_1 = 8.8 Hz, J_2 = 6.4 Hz, 1H), 6.34 (d, *J* = 6.4 Hz, 1H), 3.18 (t, *J* = 4.4 Hz, 2H), 3.02 (t, *J* = 4.4 Hz, 2H), 2.37 (s, 3H); **^{13}C NMR (101 MHz, CDCl$_3$)** δ 169.1, 142.0, 138.8, 133.5, 131.3, 127.9, 126.9, 126.5, 121.5, 119.9, 119.5, 117.9, 113.9, 111.0, 110.7, 34.3, 31.8, 20.8; **IR (cm^{-1})**: *v* 3049, 2952, 2855, 1767, 1619, 1201, 1027, 712; **HRMS** (ESI) m/z calculated for $C_{20}H_{16}BrNNaO_2$ [M + Na]$^+$: 404.0257, found: 404.0251.

2-((tert-butyldimethylsilyl)oxy)-8-(4-fluorophenyl)-6,7-dihydroazepino[2,1,7-cd]indolizine

According to the procedure A, compound **2f** was obtained as a yellow thick liquid in 90% yield;

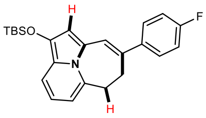

2f

^{1}H NMR (500 MHz, CDCl$_3$) δ 7.40 – 7.37 (m, 2H), 7.31 (d, *J* = 8.5 Hz, 1H), 7.05 (t, *J* = 8.5 Hz, 2H), 6.76 (s, 1H), 6.56 (t, *J* = 7.5 Hz, 1H), 6.45 (s, 1H), 6.25 (d, *J* = 6.0 Hz, 1H), 3.17 (t, *J* = 5.0 Hz, 2H), 3.06 (t, *J* = 5.0 Hz, 2H), 1.09 (s, 9H), 0.26 (s, 6H); **^{13}C NMR (126 MHz, CDCl$_3$)** δ 162.3, 160.4, 139.7 (d, *J* = 3.3 Hz), 138.3, 133.7, 132.6, 126.7 (d, *J* = 7.7 Hz), 126.5, 120.4, 119.1, 115.2 (d, *J* = 10.1 Hz), 114.9 (d, *J* = 12.5 Hz), 110.6, 108.6, 34.5, 32.3, 25.8, 18.2, -4.6; **^{19}F NMR (564 MHz, CDCl$_3$)** δ -117.11 (s, 1F); **IR (cm^{-1})**: *v* 3043, 2952, 2857, 1617, 1435, 1391, 1252, 1049, 716; **HRMS** (ESI) m/z calculated for $C_{24}H_{29}FNOSi$ [M + H]$^+$: 394.1997, found: 394.1997.

2-((tert-butyldimethylsilyl)oxy)-8-(thiophen-3-yl)-6,7-dihydroazepino[2,1,7-cd]indolizine

According to the procedure A, compound **2g** was obtained as a yellow amorphous solid in 92%

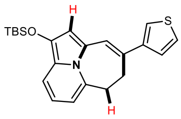

2g

yield; **^{1}H NMR (500 MHz, CDCl$_3$)** δ 7.32 – 7.26 (m, 3H), 7.09 (s, 1H), 6.91 (s, 1H), 6.54 (t, *J* = 8.5 Hz, 1H), 6.43 (d, *J* = 6.0 Hz, 1H), 6.24 (d, *J* = 6.0 Hz, 1H), 3.17 (t, *J* = 5.0 Hz, 2H), 3.07 (t, *J* = 5.0 Hz, 2H), 1.07 (s, 9H), 0.25 (s, 6H); **^{13}C NMR (126 MHz, CDCl$_3$)** δ 144.7, 138.2, 133.7, 129.1, 126.6, 125.7, 125.0, 120.3, 117.9, 117.7, 115.2, 114.8, 110.5, 108.4, 34.3, 31.9, 25.8, 18.2, -4.6; **IR (cm^{-1})**: *v* 3092, 2950, 2857, 1612, 1435, 1361, 1252, 1047, 715; **HRMS** (ESI) m/z calculated for $C_{22}H_{28}NOSSi$ [M + H]$^+$: 382.1655, found: 382.1655.

2-((tert-butyldimethylsilyl)oxy)-8-(pyridin-2-yl)-6,7-dihydroazepino[2,1,7-cd]indolizine

According to the procedure A, compound **2h** was obtained as a yellow thick liquid in 91% yield; **^{1}H NMR (500 MHz, CDCl$_3$)** δ 8.54 (s, 1H), 7.58 (t, *J* = 7.5 Hz, 1H), 7.44 (d, 7.5 Hz, 1H), 7.39 (s, 1H), 7.24 (d, *J* = 6.0 Hz, 1H), 7.02 – 6.99 (m, 1H), 6.56 (t, *J* = 7.5 Hz, 1H), 6.49 (s, 1H), 6.26 (d, *J* = 6.0 Hz, 1H), 3.21 – 3.17 (m, 4H), 1.01 (s, 9H), 0.20 (s, 6H); **^{13}C NMR (126 MHz, CDCl$_3$)** δ 158.7, 148.8, 139.0, 136.2, 134.0, 131.8, 127.6, 121.5, 120.3, 120.1, 118.9, 116.2, 114.8, 110.9, 109.6, 34.5, 29.7, 25.8, 18.2, -4.6; **IR (cm^{-1})**: *v* 3054, 2928, 2855, 1617, 1435, 1260, 1049, 719; **HRMS** (ESI) m/z calculated for $C_{23}H_{29}N_2OSi$ [M + H]$^+$: 377.2044, found: 377.2034.

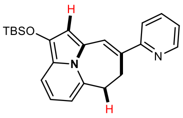

2h

(*E*)-8-styryl-6,7-dihydroazepino[2,1,7-cd]indolizin-2-yl acetate

According to the procedure A, compound **2i** was obtained as a yellow amorphous solid in 91% yield; **^{1}H NMR (400 MHz, CDCl$_3$)** δ 7.47 – 7.45 (m, 2H), 7.36 – 7.32 (m, 2H), 7.24 – 7.20 (m, 2H), 6.95 (d, *J* = 16.0 Hz, 1H), 6.84 (s, 1H), 6.71 (dd, J_1 = 8.8 Hz, J_2 = 6.4 Hz, 1H), 6.63 (s, 1H), 6.48 (d, *J* = 16.0 Hz, 1H), 6.36 (d, *J* = 6.4 Hz, 1H), 3.15 (t, *J* = 4.4 Hz, 2H), 2.92 (t, *J* = 4.4 Hz, 2H), 2.37 (s, 3H); **^{13}C NMR (101 MHz, CDCl$_3$)** δ 169.1, 139.0, 137.9, 133.9, 132.4, 128.6, 128.2, 126.9, 126.7, 126.0, 125.0, 123.0, 121.7, 118.1, 113.9, 111.3, 110.5, 33.7, 27.4, 20.8; **IR (cm^{-1})**: *v* 3052, 2928, 2855, 1749, 1588, 1200, 1049, 719; **HRMS** (ESI) m/z calculated for $C_{22}H_{20}NO_2$ [M + H]$^+$: 330.1489, found: 330.1493.

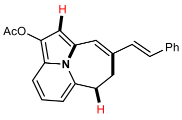

2i

2-((tert-butyldimethylsilyl)oxy)-8-cyclohexyl-6,7-dihydroazepino[2,1,7-cd]indolizine

According to the procedure A, compound **2j** was obtained as a yellow thick liquid in 90% yield; **^{1}H NMR (400 MHz, CDCl$_3$)** δ 7.23 (d, *J* = 8.8 Hz, 1H), 6.42 (dd, J_1 = 8.8 Hz, J_2 = 6.8 Hz, 1H), 6.29 (s, 1H), 6.24 (s, 1H), 6.11 (d, *J* = 6.8 Hz, 1H), 3.01 (t, *J* = 4.8 Hz, 2H), 2.61 (t, *J* = 4.8 Hz, 2H), 2.05 – 2.00 (m, 1H), 1.83 – 7.73 (m, 5H), 1.40 – 1.17 (m, 5H), 1.08 (s, 9H), 0.25 (s, 6H); **^{13}C NMR (101 MHz, CDCl$_3$)** δ 142.0, 137.9, 132.9, 125.1, 120.9, 114.9, 114.7, 113.8, 109.8, 106.8, 77.3, 77.0, 76.7, 48.0, 34.7, 31.9, 31.7, 26.6, 26.3, 25.8, 18.2, -4.6; **IR (cm^{-1})**: *v* 3048, 2926, 2854, 1659, 1458, 1384, 1256, 1059, 759; **HRMS** (ESI) m/z calculated for $C_{24}H_{35}NOSi$ [M]: 381.2488, found: 381.2480.

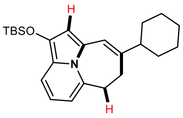

2j

2-((tert-butyldimethylsilyl)oxy)-8-heptyl-6,7-dihydroazepino[2,1,7-cd]indolizine

According to the procedure A, compound **2k** was obtained as a yellow thick liquid in 89% yield; **^{1}H NMR (500 MHz, CDCl$_3$)** δ 7.22 (d, *J* = 8.5 Hz, 1H), 6.42 (t, *J* = 8.5 Hz, 1H), 6.26 (s, 1H), 6.23 (s, 1H), 6.12 (d, *J* = 6.0 Hz, 1H), 3.03 (t, *J* = 4.0 Hz, 2H), 2.61 (t, *J* = 4.0 Hz, 2H), 2.19 (t, *J* = 7.0 Hz, 2H), 1.52 – 1.49 (m, 2H), 1.35 – 1.32 (m, 8H), 1.06 (s, 9H), 0.94 – 0.91 (m, 3H), 0.23 (s, 6H); **^{13}C NMR (126 MHz, CDCl$_3$)** δ 138.0, 137.1, 132.9, 125.1, 120.8, 116.8, 114.8, 113.8, 110.0, 106.7, 40.5, 34.4, 33.2, 31.9, 29.3, 29.2, 28.2, 25.8, 22.7, 18.2, 14.1, -4.6; **IR (cm^{-1})**: *v* 3022, 2927, 2855, 1621, 1434, 1361, 1250, 1047, 715; **HRMS** (ESI) m/z calculated for $C_{25}H_{40}NOSi$ [M + H]$^+$: 398.2874, found: 398.2875.

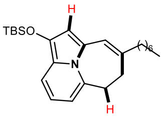

2k

9-(4-methoxyphenyl)-6,7-dihydroazepino[2,1,7-cd]indolizin-2-yl acetate

According to the procedure A, compound **2l** was obtained as a yellow thick liquid in 95% yield; **^{1}H NMR (400 MHz, CDCl$_3$)** δ 7.38 – 7.35 (m, 2H), 7.20 (d, J = 9.2 Hz, 1H), 6.93 – 6.89 (m, 2H), 6.68 (dd, J_1 = 9.2 Hz, J_2 = 6.8 Hz, 1H), 6.44 (d, J = 1.6 Hz, 1H), 6.31 (t, J = 6.8 Hz, 1H), 5.79 (t, J = 6.8 Hz, 1H), 3.85 (s, 3H), 3.20 (t, J = 4.4 Hz, 2H), 2.76 – 2.72 (m, 2H), 2.30 (m, 3H); **^{13}C NMR (101 MHz, CDCl$_3$)** δ 169.2, 158.7, 139.4, 135.9, 135.4, 130.1, 127.0, 126.6, 126.1, 123.9, 117.4, 113.8, 113.2, 111.0, 110.9, 55.2, 35.9, 28.8, 20.7; **IR (cm^{-1})**: v 3052, 2928, 2856, 1770, 1631, 1208, 1083, 719; **HRMS** (ESI) m/z calculated for $C_{21}H_{20}NO_3$ [M + H]$^+$: 334.1438, found: 334.1432.

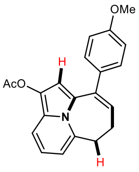

2l

9-(4-chlorophenyl)-6,7-dihydroazepino[2,1,7-cd]indolizin-2-yl acetate

According to the procedure A, compound **2m** was obtained as a yellow thick liquid in 92% yield; **^{1}H NMR (400 MHz, CDCl$_3$)** δ 7.37 – 7.31 (m, 4H), 7.19 (dd, J_1 = 8.8 Hz, J_2 = 1.2 Hz, 1H), 6.70 (dd, J_1 = 8.8 Hz, J_2 = 6.4 Hz, 1H), 6.36 (s, 1H), 6.33 (d, J = 6.4 Hz, 1H), 5.78 (t, J = 6.4 Hz, 1H), 3.19 (t, J = 4.8 Hz, 2H), 2.76 – 2.72 (m, 2H), 2.30 (s, 3H); **^{13}C NMR (101 MHz, CDCl$_3$)** δ 169.3, 141.9, 139.4, 134.9, 132.9, 130.5, 128.1, 127.1, 126.8, 123.2, 117.7, 113.9, 111.3, 110.8, 35.8, 28.9, 20.7; **IR (cm^{-1})**: v 3048, 2928, 2854, 1766, 1618, 1218, 1014, 763; **HRMS** (ESI) m/z calculated for $C_{20}H_{16}ClNNaO_2$ [M + Na]$^+$: 360.0762, found: 360.0765.

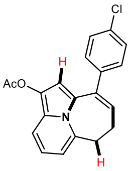

2m

2-((tert-butyldimethylsilyl)oxy)-9-methyl-6,7-dihydroazepino[2,1,7-cd]indolizine

According to the procedure A, compound **2n** was obtained as a yellow thick liquid in 88% yield; **^{1}H NMR (500 MHz, CDCl$_3$)** δ 7.14 (d, J = 8.5 Hz, 1H), 6.38 – 6.35 (m, 2H), 6.01 (d, J = 6.0 Hz, 1H), 5.54 (t, J = 6.0 Hz, 1H), 2.93 (t, J = 4.5 Hz, 2H), 2.49 (t, J = 4.5 Hz, 2H), 2.06 (s, 3H), 0.96 (s, 9H), 0.13 (s, 6H); **^{13}C NMR (126 MHz, CDCl$_3$)** δ 138.9, 132.8, 127.7, 125.9, 123.3, 122.6, 114.7, 114.6, 110.2, 105.8, 35.4, 29.0, 25.8, 23.5, 18.2, -4.6; **IR (cm^{-1})**: v 3010, 2928, 2856, 1628, 1431, 1387, 1251, 1058, 716; **HRMS** (ESI) m/z calculated for $C_{19}H_{28}NOSi$ [M + H]$^+$: 314.1935, found: 314.1934.

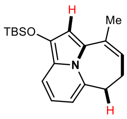

2n

9-methyl-7-phenyl-6,7-dihydroazepino[2,1,7-cd]indolizin-2-yl acetate

According to the procedure A, compound **2o** was obtained as a yellow thick liquid in 92% yield; **^{1}H NMR (400 MHz, CDCl$_3$)** δ 7.32 – 7.29 (m, 2H), 7.24 – 7.21 (m, 2H), 7.15 – 7.13 (m, 2H), 7.00 (s, 1H), 6.63 (dd, J_1 = 8.4 Hz, J_2 = 6.8 Hz, 1H), 6.19 (d, J = 6.0 Hz, 1H), 5.80 (d, J = 4.0 Hz, 2H), 3.91 (s, 1H), 3.31 – 3.20 (m, 2H), 2.38 (s, 3H), 2.27 (s, 3H); **^{13}C NMR (101 MHz, CDCl$_3$)** δ 169.4, 145.6, 137.4, 128.6, 127.7, 127.4, 127.2, 127.0, 126.4, 126.2, 123.2, 117.5, 113.8, 112.1, 108.0, 45.6, 43.2, 23.7, 20.9; **IR (cm^{-1})**: v 3022, 2924, 2855, 1766, 1631, 1210, 1013, 701; **HRMS** (ESI) m/z calculated for $C_{21}H_{20}NO_2$ [M + H]$^+$: 318.1489, found: 318.1483.

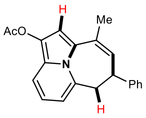

2o

7,8-diphenyl-6,7-dihydroazepino[2,1,7-cd]indolizin-2-yl acetate

According to the procedure A, compound **2p** was obtained as a yellow thick liquid in 86% yield; **^{1}H NMR (400 MHz, CDCl$_3$)** δ 7.40 – 7.38 (m, 2H), 7.30 – 7.26 (m, 2H), 7.21 – 7.16 (m, 3H), 7.14 – 7.06 (m, 3H), 7.02 – 7.01 (m, 3H), 6.55 (t, J = 7.6 Hz, 1H), 6.12 (d, J = 7.6 Hz, 1H), 4.83 (d, J = 4.8 Hz, 1H), 3.48 (dd, J_1 = 14.4 Hz, J_2 = 6.4 Hz, 1H), 3.22 (d, J = 6.4 Hz, 1H), 2.36 (s, 3H); **^{13}C NMR (101 MHz, CDCl$_3$)** δ 169.0, 143.3, 143.2, 136.0, 135.0, 128.6, 128.4, 128.0, 127.2, 126.4, 126.3, 126.2, 125.6, 121.1, 120.5, 117.9, 113.7, 113.2, 110.9, 49.3, 41.3, 21.0; **IR (cm^{-1})**: v 3058, 2924, 2855, 1765, 1621, 1201, 1028, 699; **HRMS** (ESI) m/z calculated for $C_{26}H_{21}NNaO_2$ [M + Na]$^+$: 402.1465, found: 402.1469.

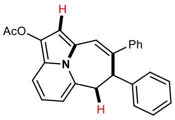

2p

7-(4-methoxyphenyl)-8-phenyl-6,7-dihydroazepino[2,1,7-cd]indolizin-2-yl acetate

According to the procedure A, compound **2q** was obtained as a yellow thick liquid in 88% yield; **^{1}H NMR (400 MHz, CDCl$_3$)** δ 7.45 – 7.42 (m, 2H), 7.32 – 7.28 (m, 2H), 7.22 – 7.19 (m, 3H), 7.05 (d, J = 2.4 Hz, 1H), 6.97 – 6.95 (m, 2H), 6.68 – 6.65 (m, 2H), 6.58 (dd, J_1 = 8.4 Hz, J_2 = 6.8 Hz, 1H), 6.15 (d, J = 6.8 Hz, 1H), 4.79 (d, J = 6.4 Hz, 1H), 3.67 (s, 3H), 3.45 (dd, J_1 = 14.4 Hz, J_2 = 6.4 Hz, 1H), 3.21 (d, J = 14.4 Hz, 1H), 2.38 (s, 3H); **^{13}C NMR (101 MHz, CDCl$_3$)** δ 169.0, 157.9, 143.1, 136.1, 135.4, 135.3, 128.3, 128.1, 127.9, 126.2, 126.1, 125.5, 121.0, 120.2, 117.9, 113.8, 113.6, 113.2, 110.8, 54.9, 48.4, 41.3, 20.9; **IR (cm^{-1})**: v 3051, 2928, 2855, 1749, 1609, 1249, 1034, 739; **HRMS** (ESI) m/z calculated for $C_{27}H_{24}NO_3$ [M + H]$^+$: 410.1751, found: 410.1744.

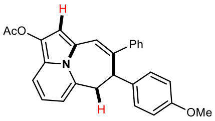

2q

8-phenyl-7-(p-tolyl)-6,7-dihydroazepino[2,1,7-cd]indolizin-2-yl acetate

According to the procedure A, compound **2r** was obtained as a yellow thick liquid in 86% yield; **^{1}H NMR (400 MHz, CDCl$_3$)** δ 7.44 – 7.42 (m, 2H), 7.31 – 7.28 (m, 2H), 7.22 – 7.18 (m, 3H), 7.05 (s, 1H), 6.94 – 6.93 (m, 4H), 6.57 (dd, J_1 = 8.8 Hz, J_2 = 6.4 Hz, 1H), 6.15 (d, J = 6.4 Hz, 1H), 4.81 (d, J = 5.2 Hz, 1H), 3.48 (dd, J_1 = 14.4 Hz, J_2 = 6.4 Hz, 1H), 3.23 (d, J = 14.4 Hz, 1H), 2.38 (s, 3H), 2.22 (s, 3H); **^{13}C NMR (101 MHz, CDCl$_3$)** δ 169.0, 143.2, 140.2, 136.2, 135.7, 135.3, 129.3, 128.3, 127.9, 127.0, 126.2, 126.1, 125.6, 121.1, 120.3, 117.9, 113.6, 113.2, 110.8, 48.9, 41.2, 20.9; **IR (cm^{-1})**: v 3025, 2922, 2851, 1766, 1621, 1221, 1017, 720; **HRMS** (ESI) m/z calculated for $C_{27}H_{24}NO_2$ [M + H]$^+$: 394.1802, found: 394.1801.

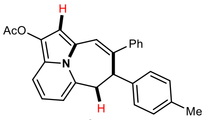

2r

7-(4-bromophenyl)-8-phenyl-6,7-dihydroazepino[2,1,7-cd]indolizin-2-yl acetate

According to the procedure A, compound **2s** was obtained as a yellow thick liquid in 81% yield; **^{1}H NMR (400 MHz, CDCl$_3$)** δ 7.27 – 7.24 (m, 2H), 7.21 – 7.16 (m, 2H), 7.14 – 7.08 (m, 5H), 6.93 (t, J = 4.0 Hz, 1H), 6.81 – 6.78 (m, 2H), 6.47 (t, J = 7.6 Hz, 1H), 6.03 (d, J = 6.4 Hz, 1H), 4.68 (d, J = 4.8 Hz, 1H), 3.34 (dd, J_1 = 14.4 Hz, J_2 = 6.4 Hz, 1H), 3.10 (d, J = 14.4 Hz, 1H), 2.26 (s, 3H); **^{13}C NMR (101 MHz, CDCl$_3$)** δ 169.0, 142.9, 142.2, 135.6, 134.4, 131.7, 129.0, 128.4, 128.1, 126.4, 126.4, 125.5, 120.9, 120.7, 120.3, 118.0, 114.0, 113.4, 111.1, 48.7, 41.0, 20.9; **IR (cm^{-1})**: v 3027, 2922, 2855, 1765, 1621, 1210, 1010, 697; **HRMS** (ESI) m/z calculated for $C_{26}H_{21}BrNO_2$ [M + H]$^+$: 458.0750, found: 458.0759.

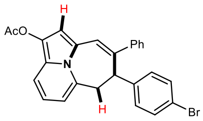

2s

8-phenyl-7-(pyridin-2-yl)-6,7-dihydroazepino[2,1,7-cd]indolizin-2-yl acetate

According to the procedure A, compound **2t** was obtained as a yellow thick liquid in 85% yield; **^{1}H NMR (500 MHz, $CDCl_3$)** δ 8.57 (d, *J* = 4.0 Hz, 1H), 7.43 – 7.42 (m, 2H), 7.31 – 7.16 (m, 6H), 6.97 (t, *J* = 6.5 Hz, 1H), 6.63 (d, *J* = 8.5 Hz, 1H), 6.59 (s, 1H), 6.42 (dd, J_1 = 8.5 Hz, J_2 = 6.5 Hz, 1H), 6.11 (d, *J* = 6.0 Hz, 1H), 5.11 (d, *J* = 6.0 Hz, 1H), 3.84 (dd, J_1 = 14.5 Hz, J_2 = 6.5 Hz, 1H), 3.13 (d, *J* = 14.5 Hz, 1H), 1.05 (s, 9H), 0.27 (s, 3H), 0.24 (s, 3H); **^{13}C NMR (126 MHz, $CDCl_3$)** δ 162.4, 149.2, 142.7, 136.7, 135.3, 133.5, 132.2, 128.4, 126.9, 125.9, 125.3, 121.4, 121.1, 120.7, 119.9, 115.9, 114.5, 112.9, 109.2, 77.3, 77.0, 76.7, 51.5, 39.7, 25.7, 18.2, -4.5, -4.7; **IR (cm^{-1})**: *v* 3055, 2953, 2855, 1617, 1425, 1360, 1252, 1051, 694; **HRMS** (ESI) m/z calculated for $C_{29}H_{33}N_2OSi$ [M + H]$^+$: 453.2357, found: 453.2351.

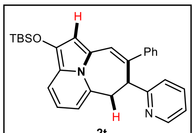

2t

(*E*)-8-phenyl-7-styryl-6,7-dihydroazepino[2,1,7-cd]indolizin-2-yl acetate

According to the procedure A, compound **2u** was obtained as a yellow amorphous solid in 91% yield; **^{1}H NMR (400 MHz, $CDCl_3$)** δ 7.55 – 7.53 (m, 2H), 7.36 – 7.32 (m, 2H), 7.26 – 7.20 (m, 2H), 7.18 – 7.09 (m, 5H), 7.04 (s, 1H), 6.97 (s, 1H), 6.69 (dd, J_1 = 8.0 Hz, J_2 = 6.8 Hz, 1H), 6.41 (d, *J* = 6.8 Hz, 1H), 6.34 (d, *J* = 15.6 Hz, 1H), 6.05 (dd, J_1 = 15.6 Hz, J_2 = 6.8 Hz, 1H), 4.37 (t, *J* = 6.4 Hz, 1H), 3.42 (dd, J_1 = 14.4 Hz, J_2 = 6.4 Hz, 1H), 3.04 (d, *J* = 14.4 Hz, 1H), 2.34 (s, 3H); **^{13}C NMR (101 MHz, $CDCl_3$)** δ 169.0, 143.0, 137.0, 136.2, 135.2, 131.0, 130.2, 128.4, 128.2, 128.0, 127.1, 126.3, 126.2, 125.6, 121.1, 119.3, 117.8, 114.0, 112.9, 110.8, 46.3, 39.9, 20.9; **IR (cm^{-1})**: *v* 3049, 2924, 2851, 1746, 1637, 1223, 1019, 692; **HRMS** (ESI) m/z calculated for $C_{28}H_{24}NO_2$ [M + H]$^+$: 406.1802, found: 406.1795.

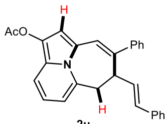

2u

7-cyclohexyl-8-phenyl-6,7-dihydroazepino[2,1,7-cd]indolizin-2-yl acetate

According to the procedure A, compound **2v** was obtained as a yellow thick liquid in 82% yield; **^{1}H NMR (400 MHz, $CDCl_3$)** δ 7.37 – 7.35 (m, 2H), 7.24 – 7.20 (m, 2H), 7.17 – 7.15 (m, 1H), 7.11 – 7.08 (m, 1H), 6.80 – 6.78 (m, 1H), 6.73 (d, 1H, *J* = 2.0 Hz), 6.62 – 6.58 (m, 1H), 6.32 (dd, J_1 = 6.8 Hz, J_2 = 1.2 Hz, 1H), 3.36 – 3.24 (m, 2H), 2.71 (d, *J* = 14.4 Hz, 1H), 2.23 (s, 3H), 1.95 – 1.93 (m, 1H), 1.49 – 1.32 (m, 4H), 0.91 – 0.66 (m, 6H); **^{13}C NMR (101 MHz, $CDCl_3$)** δ 169.1, 145.2, 139.5, 136.3, 128.3, 127.7, 126.0, 125.7, 125.6, 121.3, 118.4, 117.3, 113.7, 113.1, 109.9, 48.6, 42.2, 36.5, 31.8, 30.6, 26.3, 26.1, 25.9, 20.9; **IR (cm^{-1})**: *v* 3051, 2925, 2851, 1764, 1618, 1213, 1026, 761; **HRMS** (ESI) m/z calculated for $C_{26}H_{28}NO_2$ [M + H]$^+$: 386.2115, found: 386.2108.

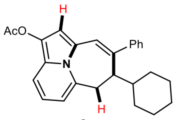

2v

7-methyl-8-phenyl-6,7-dihydroazepino[2,1,7-cd]indolizin-2-yl acetate

According to the procedure A, compound **2w** was obtained as a yellow thick liquid in 86% yield; **^{1}H NMR (400 MHz, $CDCl_3$)** δ 7.56 – 7.54 (m, 2H), 7.42 – 7.26 (m, 4H), 6.95 (s, 1H), 6.90 (s, 1H), 6.77 (dd, J_1 = 8.8 Hz, J_2 = 6.8 Hz, 1H), 6.49 (d, *J* = 6.8 Hz, 1H), 3.74 – 3.68 (m, 1H), 3.20 (dd, J_1 = 14.4 Hz, J_2 = 6.4 Hz, 1H), 2.95 (d, *J* = 6.4 Hz, 1H), 2.40 (s, 3H), 1.07 (d, *J* = 7.6 Hz, 3H); **^{13}C NMR (101 MHz, $CDCl_3$)** δ 169.2, 142.9, 139.9, 136.5, 128.4, 127.9, 126.3, 126.0, 125.4, 121.3, 117.6, 117.5, 113.9, 113.0, 110.4, 40.0, 36.6, 20.9, 19.8; **IR (cm^{-1})**: *v* 3033, 2958, 2854, 1750, 1617, 1225, 1049, 702; **HRMS** (ESI) m/z calculated for $C_{21}H_{20}NO_2$ [M + H]$^+$: 318.1489, found: 318.1481.

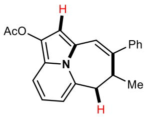

2w

4,5,6,7,8,9-hexahydrobenzo[3,4]azepino[2,1,7-cd]indolizin-11-yl acetate

According to the procedure A, compound **2x** was obtained as a yellow thick liquid in 90% yield; **^{1}H NMR (400 MHz, CDCl$_3$)** δ 7.20 (d, J = 8.8 Hz, 1H), 6.46 (s, 1H), 6.39 (dd, J_1 = 8.8 Hz, J_2 = 6.8 Hz, 1H), 6.08 (d, J = 6.8 Hz, 1H), 3.04 (t, J = 4.0 Hz, 2H), 2.52 – 2.46 (m, 4H), 2.22 (t, J = 4.0 Hz, 2H), 1.75 – 1.60 (m, 4H), 1.05 (s, 9H), 0.21 (s, 6H); **^{13}C NMR (101 MHz, CDCl$_3$)** δ 138.4, 132.9, 132.7, 124.7, 124.0, 123.0, 114.6, 113.4, 109.2, 104.2, 77.3, 77.0, 76.7, 35.3, 35.0, 33.6, 29.4, 25.8, 23.0, 22.9, 18.2, -4.5; **IR (cm^{-1})**: v 3013, 2928, 2856, 1629, 1420, 1387, 1250, 756; **HRMS** (ESI) m/z calculated for $C_{22}H_{32}NOSi$ $[M + H]^+$: 354.2248, found: 354.2248.

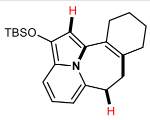

2x

5-phenyl-4,5,6,7,8,9-hexahydrobenzo[3,4]azepino[2,1,7-cd]indolizin-11-yl acetate

According to the procedure A, compound **2y** was obtained as a yellow thick liquid in 92% yield; **^{1}H NMR (400 MHz, CDCl$_3$)** δ 7.14 – 7.06 (m, 5H), 6.94 – 6.93 (m, 2H), 6.44 (dd, J_1 = 8.8 Hz, J_2 = 6.8 Hz, 1H), 5.99 (d, J = 6.8 Hz, 1H), 4.05 (d, J = 4.8 Hz, 1H), 3.36 – 3.25 (m, 2H), 2.78 – 2.76 (m, 2H), 2.37 – 2.22 (m, 5H), 2.00 – 1.97 (m, 1H), 1.83 – 1.80 (m, 1H), 1.72 – 1.62 (m, 1H), 1.54 – 1.44 (m, 1H); **^{13}C NMR (101 MHz, CDCl$_3$)** δ 169.2, 142.5, 136.0, 133.7, 128.1, 127.6, 127.1, 126.1, 124.8, 124.2, 116.4, 113.3, 111.8, 106.2, 51.7, 40.8, 33.1, 29.6, 22.9, 22.8, 20.8; **IR (cm^{-1})**: v 3058, 2930, 2858, 1747, 1610, 1224, 1038, 702; **HRMS** (ESI) m/z calculated for $C_{24}H_{24}NO_2$ $[M + H]^+$: 358.1802, found: 358.1793.

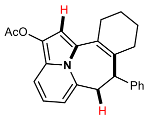

2y

5-phenyl-4,5,6,7,8,9-hexahydrobenzo[3,4]azepino[2,1,7-cd]indolizin-11-yl acetate

According to the procedure A, compound **2z** was obtained as a yellow amorphous solid in 89% yield; **^{1}H NMR (400 MHz, CDCl$_3$)** δ 7.28 (d, J = 8.8 Hz, 1H), 6.91 (d, J = 4.8 Hz, 1H), 6.74 (d, J = 4.8 Hz, 1H), 6.63 (s, 1H), 6.45 (dd, J_1 = 8.8 Hz, J_2 = 6.4 Hz, 1H), 6.21 (d, J = 6.4 Hz, 1H), 3.19 – 3.12 (m, 4H), 1.09 (s, 9H), 0.27 (s, 6H); **^{13}C NMR (101 MHz, CDCl$_3$)** δ 137.6, 135.8, 133.6, 132.2, 129.8, 125.3, 119.7, 117.0, 115.4, 114.0, 111.7, 104.6, 35.0, 29.6, 25.8, 18.2, -4.6; **IR (cm^{-1})**: v 3049, 2952, 2856, 1630, 1458, 1390, 1247, 1053, 722; **HRMS** (ESI) m/z calculated for $C_{20}H_{25}NOSSi$ [M]: 355.1426, found: 355.1416.

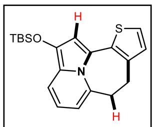

2z

5-((tert-butyldimethylsilyl)oxy)-9,10-dihydrothieno[3',2':3,4]azepino[2,1,7-cd]indolizine

According to the procedure A, compound **2aa** was obtained as a yellow amorphous solid in 93% yield; **^{1}H NMR (500 MHz, CDCl$_3$)** δ 7.26 – 7.23 (m, 2H), 7.00 (d, J = 5.5 Hz, 1H), 6.61 (s, 1H), 6.41 (t, J = 7.5 Hz, 1H), 6.18 (d, J = 7.5 Hz, 1H), 3.23 – 3.18 (m, 4H), 1.04 (s, 9H), 0.21 (s, 6H); **^{13}C NMR (126 MHz, CDCl$_3$)** δ 137.2, 133.3, 132.9, 130.7, 127.3, 125.0, 121.9, 119.1, 115.4, 113.5, 111.5, 104.6, 35.7, 28.9, 25.8, 18.2, -4.5; **IR (cm^{-1})**: v 3055, 2952, 2856, 1632, 1426, 1360, 1247, 1086, 730; **HRMS** (ESI) m/z calculated for $C_{20}H_{25}NOSSi$ [M]: 355.1426, found: 355.1416.

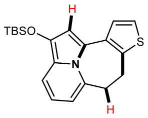

2aa

12-((tert-butyldimethylsilyl)oxy)-4,5-dihydrobenzofuro[2',3':3,4]azepino[2,1,7-cd]indolizine

According to the procedure A, compound **2ab** was obtained as a yellow amorphous solid in 92% yield; **^{1}H NMR (400 MHz, CDCl$_3$)** δ 7.50 – 7.48 (m, 1H), 7.43 – 7.38 (m, 2H), 7.26 – 7.21 (m, 2H), 7.03 (t, J = 2.0 Hz, 1H), 6.61 – 6.57 (m, 1H), 6.36 (d, J = 6.4 Hz, 1H), 3.28 – 3.25 (m, 2H), 3.20 – 3.16 (m, 2H), 1.13 (t, J = 1.6 Hz, 9H), 0.33 (t, J = 1.6 Hz, 6H); **^{13}C NMR (101 MHz, CDCl$_3$)** δ 153.7, 146.9, 137.7, 134.1, 130.1, 126.9, 122.8, 122.5, 117.6, 115.6, 115.3, 114.1, 113.0, 112.0, 110.5, 103.9, 34.3, 25.8, 22.8, 18.2, -4.5; **IR (cm^{-1})**: v 3056, 2953, 2855, 1621, 1457, 1300, 1249, 1052, 741; **HRMS** (ESI) m/z calculated for $C_{24}H_{27}NO_2Si$ [M]: 389.1811, found: 389.1802.

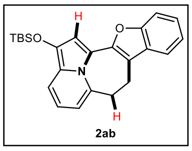

12-((tert-butyldimethylsilyl)oxy)-aza-methyl-4,5-dihydrobenzofuro[2',3':3,4]azepino[2,1,7-cd]indolizine

According to the procedure A, compound **2ac** was obtained as a yellow amorphous solid in 91% yield; **^{1}H NMR (500 MHz, CDCl$_3$)** δ 7.38 (d, J = 7.0 Hz, 1H), 7.21 – 7.17 (m, 2H), 7.09 – 7.06 (m, 1H), 7.03 – 7.00 (m, 1H), 6.61 (d, J = 2.5 Hz, 1H), 6.41 – 6.37 (m, 1H), 6.13 (d, J = 7.0 Hz, 1H), 3.82 (s, 3H), 3.1 – 3.10 (m, 4H), 0.97 (t, J = 3.0 Hz, 9H), 0.16 (t, J = 3.0 Hz, 6H); **^{13}C NMR (126 MHz, CDCl$_3$)** δ 138.8, 138.5, 133.4, 131.4, 127.2, 125.7, 121.1, 119.4, 117.1, 115.2, 114.8, 114.7, 114.6, 111.9, 109.1, 105.8, 37.8, 33.0, 25.8, 23.7, 18.3, -4.5; **IR (cm^{-1})**: v 3053, 2952, 2855, 1632, 1469, 1361, 1248, 1059, 737; **HRMS** (ESI) m/z calculated for $C_{25}H_{31}N_2OSi$ $[M + H]^+$: 403.2200, found: 403.2209.

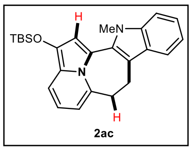

(*E*)-1-((tert-butyldimethylsilyl)oxy)-5-phenyl-3-styrylindolizine

According to the procedure A, compound **2ad** was obtained as a yellow liquid in 83% yield; **^{1}H NMR (400 MHz, CDCl$_3$)** δ 7.52 – 7.47 (m, 5H), 7.33 (dt, J_1 = 8.8 Hz, J_2 = 1.2 Hz, 1H), 7.18 – 7.14 (m, 2H), 7.09 – 7.05 (m, 1H), 6.84 – 6.82 (m, 2H), 6.66 (s, 1H), 6.55 (dd, J_1 = 8.8 Hz, J_2 = 6.4 Hz, 1H), 6.49 (d, J = 16.0 Hz, 1H), 6.31 – 6.25 (m, 2H), 1.08 (s, 9H), 0.27 (s, 6H); **^{13}C NMR (101 MHz, CDCl$_3$)** δ 138.1, 137.7, 136.4, 134.4, 128.8, 128.8, 128.6, 128.3, 126.1, 126.0, 125.5, 121.9, 121.5, 120.1, 116.4, 114.8, 114.0, 103.4, 25.8, 18.2, -4.5; **IR (cm^{-1})**: v 3057, 2928, 2857, 1549, 1439, 1321, 1088, 900; **HRMS** (ESI) m/z calculated for $C_{24}H_{20}NO_2$ $[M + H]^+$: 426.2248, found: 426.2239.

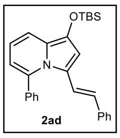

1-phenyl-2-(8-phenyl-6,7-dihydroazepino[2,1,7-cd]indolizin-2-yl)ethyl acetate

According to the procedure B, compound **4a** was obtained as a yellow thick liquid, HOAc in 63% yield, NaOAc in 80% yield; **^{1}H NMR (400 MHz, CDCl$_3$)** δ 7.28 – 7.04 (m, 11H), 6.70 (s, 1H), 6.50 (t, J = 7.6 Hz, 1H), 6.44 (s, 1H), 6.14 (d, J = 7.6 Hz, 1H), 5.87 – 5.82 (m, 1H), 3.24 (dd, J_1 = 14.4 Hz, J_2 = 7.6 Hz, 1H), 3.11 – 3.05 (m, 1H), 3.01 (t, J = 3.2 Hz, 2H), 2.90 (t, J = 3.2 Hz, 2H), 1.9 (s, 3H); **^{13}C NMR (101 MHz, CDCl$_3$)** δ 170.1, 143.4, 140.3, 139.3, 134.9, 133.6, 128.2, 127.8, 126.5, 125.9, 125.2, 124.3, 119.8, 119.2, 117.4, 115.1, 110.4, 109.6, 76.5, 34.3, 33.0, 32.0, 21.2; **IR (cm^{-1})**: v 3027, 2959, 2851, 1740, 1630, 1236, 1022, 758; **HRMS** (ESI) m/z calculated for $C_{28}H_{26}NO_2$ $[M + H]^+$: 408.1958, found: 408.1960.

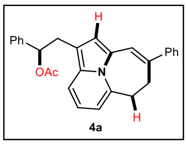

1-phenyl-2-(8-(thiophen-3-yl)-6,7-dihydroazepino[2,1,7-cd]indolizin-2-yl)ethyl acetate

According to the procedure B, compound **4b** was obtained as a yellow thick liquid in 89% yield; **^{1}H NMR (400 MHz, CDCl$_3$)** δ 7.32 – 7.25 (m, 7H), 7.20 (d, J = 9.2 Hz, 1H), 7.05 (s, 1H), 6.89 (s, 1H), 6.62 (t, J = 6.4 Hz, 1H), 6.54 (s, 1H), 6.28 (d, J = 6.4 Hz, 1H), 5.94 (t, J = 6.4 Hz, 1H), 3.35 (dd, J_1 = 14.8 Hz, J_2 = 7.6 Hz, 1H), 3.21 – 3.15 (m, 3H), 3.02 (t, J = 3.2 Hz, 2H), 2.03 (s, 3H); **^{13}C NMR (101 MHz, CDCl$_3$)** δ 170.2, 144.7, 140.4, 139.2, 135.0, 129.0, 128.3, 127.8, 126.6, 125.7, 125.0, 124.0, 119.6, 117.9, 117.5, 115.2, 110.5, 109.7, 76.6, 34.2, 33.0, 31.8, 21.3; **IR (cm^{-1})**: v 3033, 2921, 2855, 1736, 1618, 1238, 1024, 699; **HRMS** (ESI) m/z calculated for $C_{26}H_{24}NO_2S$ [M + H]$^+$: 414.1522, found: 414.1525.

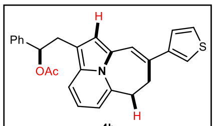

4b

(*E*)-1-phenyl-2-(8-styryl-6,7-dihydroazepino[2,1,7-cd]indolizin-2-yl)ethyl acetate

According to the procedure B, compound **4c** was obtained as a yellow amorphous solid in 75% yield; **^{1}H NMR (400 MHz, CDCl$_3$)** δ 7.46 – 7.44 (m, 2H), 7.35 – 7.30 (m, 7H), 7.25 – 7.18 (m, 2H), 6.95 (d, J = 16.0 Hz, 1H), 6.69 (dd, J_1 = 8.8 Hz, J_2 = 6.4 Hz, 1H), 6.23 (s, 1H), 6.56 (s, 1H), 6.45 (d, J = 16.0 Hz, 1H), 6.36 (d, J = 6.4 Hz, 1H), 5.96 (t, J = 6.4 Hz, 1H), 3.37 (dd, J_1 = 14.8 Hz, J_2 = 7.6 Hz, 1H), 3.23 – 3.14 (m, 3H), 2.93 (t, J = 3.2 Hz, 2H), 2.07 (s, 3H); **^{13}C NMR (101 MHz, CDCl$_3$)** δ 170.3, 140.3, 139.5, 138.1, 135.6, 132.7, 132.6, 128.6, 128.3, 127.9, 126.6, 126.5, 125.9, 124.5, 124.3, 123.2, 120.1, 118.1, 115.2, 110.9, 110.2, 76.5, 33.8, 33.0, 27.5, 21.3; **IR (cm^{-1})**: v 3028, 2922, 2855, 1736, 1604, 1207, 1026, 699; **HRMS** (ESI) m/z calculated for $C_{30}H_{28}NO_2$ [M + H]$^+$: 434.2115, found: 434.2106.

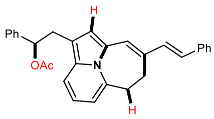

4c

2-(8-heptyl-6,7-dihydroazepino[2,1,7-cd]indolizin-2-yl)-1-phenylethyl acetate

According to the procedure B, compound **4d** was obtained as a yellow thick liquid in 75% yield; **^{1}H NMR (400 MHz, CDCl$_3$)** δ 7.35 – 7.26 (m, 5H), 7.18 (d, J = 8.8 Hz, 1H), 6.56 (dd, J_1 = 8.8 Hz, J_2 = 6.4 Hz, 1H), 6.42 (s, 1H), 6.27 (s, 1H), 6.21 (d, J = 6.4 Hz, 1H), 5.95 (t, J = 6.4 Hz, 1H), 3.36 (dd, J_1 = 14.8 Hz, J = 7.6 Hz, 1H), 3.19 (dd, J_1 = 14.8 Hz, J_2 = 6.4 Hz, 1H), 3.06 (t, J = 4.4 Hz, 2H), 2.61 (t, J = 4.4 Hz, 2H), 2.18 (t, J = 7.2 Hz, 2H), 2.05 (s, 3H), 1.51 – 1.44 (m, 2H), 1.34 – 1.30 (m, 8H), 0.92 (t, J = 6.4 Hz, 3H); **^{13}C NMR (101 MHz, CDCl$_3$)** δ 170.2, 140.6, 139.0, 137.0, 133.9, 128.2, 127.8, 126.6, 124.5, 117.4, 116.8, 116.3, 115.1, 110.0, 108.7, 76.8, 40.5, 34.3, 33.1, 31.8, 29.3, 29.2, 28.1, 22.6, 21.3, 14.1; **IR (cm^{-1})**: v 3025, 2925, 2853, 1739, 1618, 1236, 1022, 699; **HRMS** (ESI) m/z calculated for $C_{29}H_{36}NO_2$ [M + H]$^+$: 430.2741, found: 430.2734.

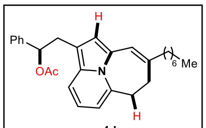

4d

2-(8-cyclohexyl-6,7-dihydroazepino[2,1,7-cd]indolizin-2-yl)-1-phenylethyl acetate

According to the procedure B, compound **4e** was obtained as a yellow thick liquid in 83% yield; **^{1}H NMR (400 MHz, $CDCl_3$)** δ 7.39 – 7.29 (m, 5H), 7.19 (d, *J* = 8.8 Hz, 1H), 6.52 – 6.48 (m, 1H), 6.47 (s, 1H), 6.23 (s, 1H), 6.14 (d, *J* = 6.4 Hz, 1H), 5.94 – 5.90 (m, 1H), 3.38 (dd, J_1 = 14.4 Hz, J_2 = 7.6 Hz, 1H), 3.32 (dd, J_1 = 14.4 Hz, J_2 = 6.0 Hz, 1H), 2.99 (t, *J* = 4.4 Hz, 2H), 2.56 (t, *J* = 4.4 Hz, 2H), 2.04 – 1.94 (m, 4H), 1.79 – 1.63 (m, 5H), 1.34 – 1.14 (m, 5H); **^{13}C NMR (101 MHz, $CDCl_3$)** δ 170.1, 141.8, 140.6, 139.0, 133.9, 128.2, 127.7, 126.6, 124.7, 117.5, 116.3, 115.0, 114.8, 109.8, 108.7, 76.7, 47.9, 34.6, 33.1, 31.8, 31.6, 26.6, 26.3, 21.3; **IR (cm^{-1})**: *v* 3035, 2923, 2848, 1740, 1633, 1236, 1023, 699; **HRMS** (ESI) m/z calculated for $C_{28}H_{32}NO_2$ $[M + H]^+$: 414.2428, found: 414.2422.

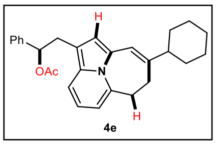

2-(6,7-dihydrothieno[3',2':3,4]azepino[2,1,7-cd]indolizin-2-yl)-1-phenylethyl acetate

According to the procedure B, compound **4f** was obtained as a yellow thick liquid in 69% yield; **^{1}H NMR (400 MHz, $CDCl_3$)** δ 7.37 – 7.27 (m, 5H), 7.22 (d, *J* = 8.8 Hz, 1H), 6.92 (d, *J* = 5.2 Hz, 1H), 6.81 (s, 1H), 6.74 (d, *J* = 5.2 Hz, 1H), 6.57 (dd, J_1 = 8.8 Hz, J_2 = 6.4 Hz, 1H), 6.29 (d, *J* = 6.4 Hz, 1H), 5.96 (dd, J_1 = 7.6 Hz, J_2 = 6.4 Hz, 1H), 3.37 (dd, J_1 = 14.4 Hz, J_2 = 7.6 Hz, 1H), 3.24 – 3.19 (m, 3H), 3.13 (t, *J* = 4.0 Hz, 2H), 2.07 (s, 3H); **^{13}C NMR (101 MHz, $CDCl_3$)** δ 170.1, 140.3, 138.6, 135.6, 133.8, 132.0, 129.7, 128.2, 127.8, 126.5, 120.7, 120.0, 116.3, 115.6, 115.2, 111.6, 109.7, 76.6, 34.9, 33.1, 29.4, 21.2; **IR (cm^{-1})**: *v* 3025, 2913, 2854, 1736, 1615, 1237, 1023, 700; **HRMS** (ESI) m/z calculated for $C_{24}H_{22}NO_2S$ $[M + H]^+$: 388.1366, found: 388.1367.

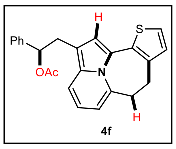

2-(6,7-dihydrothieno[3',2':3,4]azepino[2,1,7-cd]indolizin-2-yl)-1-phenylethyl acetate

According to the procedure B, compound **4g** was obtained as a yellow thick liquid in 78% yield; **^{1}H NMR (400 MHz, $CDCl_3$)** δ 7.34 – 7.27 (m, 5H), 7.23 – 7.20 (m, 2H), 6.99 (d, *J* = 5.6 Hz, 1H), 6.78 (s, 1H), 6.54 (dd, J_1 = 8.8 Hz, J_2 = 6.4 Hz, 1H), 6.26 (d, *J* = 6.4 Hz, 1H), 5.98 – 5.94 (m, 1H), 3.41 – 3.35 (m, 1H), 3.24 – 3.19 (m, 5H), 2.03 (s, 3H); **^{13}C NMR (101 MHz, $CDCl_3$)** δ 170.2, 140.4, 138.3, 133.7, 132.8, 130.6, 128.3, 127.8, 127.3, 126.6, 122.8, 121.9, 116.0, 115.8, 115.0, 111.5, 109.1, 77.3, 77.0, 76.7, 35.6, 33.2, 28.8, 21.3; **IR (cm^{-1})**: *v* 3033, 2955, 2850, 1736, 1631, 1238, 1032, 700; **HRMS** (ESI) m/z calculated for $C_{24}H_{22}NO_2S$ $[M + H]^+$: 388.1366, found: 388.1367.

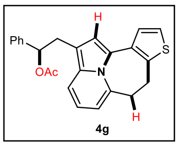

2-(aza-methyl-6,7-dihydrobenzo[4',5']indole[2',3':3,4]azepino[2,1,7-cd]indolizin-2-yl)-1-phenylethyl acetate

According to the procedure B, compound **4h** was obtained as a yellow thick liquid in 71% yield; **^{1}H NMR (400 MHz, $CDCl_3$)** δ 7.53 (d, *J* = 7.6 Hz, 1H), 7.39 – 7.30 (m, 7H), 7.22 (t, *J* = 7.6 Hz, 1H), 7.15 (t, *J* = 7.6 Hz, 1H), 6.72 – 6.67 (m, 2H), 6.38 (d, *J* = 6.8 Hz, 1H), 6.03 (td, J_1 = 6.8 Hz, J_2 = 1.6 Hz, 1H), 3.80 (s, 3H), 3.49 (dd, J_1 = 14.4 Hz, J_2 = 6.8 Hz, 1H), 3.34 – 3.22 (m, 5H), 2.10 (m, 3H); **^{13}C NMR (101 MHz, $CDCl_3$)** δ 170.3, 140.4, 139.9, 138.4, 134.3, 131.3, 128.3, 127.9, 127.1, 126.8, 121.1, 119.3, 118.4, 117.1, 117.1, 116.3, 115.6, 114.4, 111.9, 109.1, 76.6, 37.6, 33.1, 33.0, 23.6, 21.4; **IR (cm^{-1})**: *v* 3055, 2924, 2853, 1734, 1636, 1238, 1023, 700; **HRMS** (ESI) m/z calculated for $C_{29}H_{27}N_2O_2$ $[M + H]^+$: 435.2067, found: 435.2065.

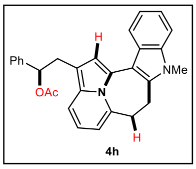

2-(8-phenyl-6,7-dihydroazepino[2,1,7-cd]indolizin-2-yl)-1-(p-tolyl)ethyl acetate

According to the procedure B, compound **4i** was obtained as a yellow thick liquid in 85% yield; **^{1}H NMR (400 MHz, CDCl$_3$)** δ 7.35 – 7.33 (m, 2H), 7.28 – 7.24 (m, 2H), 7.20 – 7.11 (m, 4H), 7.06 – 7.05 (m, 2H), 6.75 (d, J = 2.0 Hz, 1H), 6.60 – 6.56 (m, 1H), 6.50 (d, J = 2.0 Hz, 1H), 6.22 (d, J = 6.8 Hz, 1H), 5.86 (td, J_1 = 6.8 Hz, J_2 = 2.0 Hz, 1H), 3.29 (ddd, J_1 = 14.4 Hz, J_2 = 7.6 Hz, J_3 = 2.0 Hz, 1H), 3.15 – 3.09 (m, 3H), 2.99 (t, J = 3.2 Hz, 2H), 2.26 (s, 3H), 1.95 (s, 3H); **^{13}C NMR (101 MHz, CDCl$_3$)** δ 170.2, 143.5, 139.4, 137.5, 137.4, 134.9, 133.6, 129.0, 128.3, 126.6, 125.9, 125.3, 124.3, 119.8, 119.3, 117.4, 115.2, 110.5, 109.9, 77.3, 77.0, 76.7, 76.5, 34.4, 32.9, 32.1, 21.3, 21.1., 110.5, 109.9, 76.5, 34.4, 32.9, 32.1, 21.3, 21.1; **IR (cm^{-1})** v 3033, 2922, 2850, 1737, 1620, 1237, 1020, 697; **HRMS** (ESI) m/z calculated for $C_{29}H_{28}NO_2$ [M + H]$^+$: 422.2115, found: 422.2111.

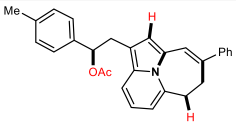

4i

1-(4-fluorophenyl)-2-(8-phenyl-6,7-dihydroazepino[2,1,7-cd]indolizin-2-yl)ethyl acetate

According to the procedure B, compound **4j** was obtained as a yellow thick liquid in 77% yield; **^{1}H NMR (400 MHz, CDCl$_3$)** δ 7.46 – 7.43 (m, 2H), 7.39 – 7.35 (m, 2H), 7.32 – 7.28 (m, 2H), 7.25 – 7.21 (m, 2H), 7.04 – 7.00 (m, 2H), 6.85 (d, J = 1.2 Hz, 1H), 6.70 – 6.65 (m, 1H), 6.56 (d, J = 1.2 Hz, 1H), 6.34 (d, J = 6.8 Hz, 1H), 5.95 (td, J_1 = 6.8 Hz, J_2 = 2.0 Hz, 1H), 3.37 (ddd, J_1 = 14.4 Hz, J_2 = 7.2 Hz, J_3 = 1.6 Hz, 1H), 3.22 – 3.17 (m, 3H), 3.11 (t, J = 3.6 Hz, 2H), 2.07 (s, 3H); **^{13}C NMR (101 MHz, CDCl$_3$)** δ 170.2, 163.5, 161.1, 143.4, 139.5, 136.2 (d, J = 3.1 Hz), 134.4 (d, J = 112.0 Hz), 128.4 (d, J = 8.2 Hz), 128.3, 126.0, 125.3, 124.4, 119.5 (d, J = 61.5 Hz), 117.6, 115.3, 115.1 (d, J = 2.7 Hz), 110.5, 109.3, 76.0, 34.5, 33.0, 32.1, 21.3; **^{19}F NMR (377 MHz, CDCl$_3$)** δ -114.25 (t, J = 4.1 Hz, 1F); **IR (cm^{-1})**: v 3023, 2924, 2851, 1739, 1620, 1227, 1028, 698; **HRMS** (ESI) m/z calculated for $C_{28}H_{25}FNO_2$ [M + H]$^+$: 426.1864, found: 426.1857.

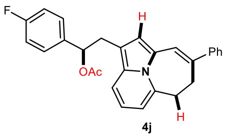

4j

1-phenyl-2-(8-phenyl-6,7-dihydroazepino[2,1,7-cd]indolizin-2-yl)ethyl formate

According to the procedure B, compound **4k** was obtained as a yellow thick liquid in 63% yield; **^{1}H NMR (400 MHz, CDCl$_3$)** δ 8.09 (s, 1H), 7.45 – 7.31 (m, 9H), 7.25 – 7.22 (m, 2H), 6.85 (s, 1H), 6.70 – 6.66 (m, 1H), 6.61 (s, 1H), 6.34 (d, J = 6.8 Hz, 1H), 6.08 (t, J = 6.8 Hz, 1H), 3.43 (dd, J_1 = 14.4 Hz, J_2 = 6.8 Hz, 1H), 3.29 – 3.20 (m, 3H), 3.10 (t, J = 4.4 Hz, 2H); **^{13}C NMR (101 MHz, CDCl$_3$)** δ 160.4, 143.4, 139.7, 139.5, 134.9, 133.9, 128.4, 128.3, 128.1, 126.7, 126.0, 125.3, 124.5, 119.8, 119.2, 117.7, 115.0, 110.5, 109.2, 76.5, 34.5, 33.0, 32.2; **IR (cm^{-1})**: v 3025, 2923, 2854, 1723, 1622, 1260, 698; **HRMS** (ESI) m/z calculated for $C_{27}H_{23}NNaO_2$ [M + Na]$^+$: 416.1621, found: 416.1630.

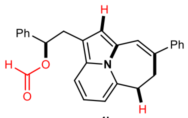

4k

2-(2-(4-methoxyphenoxy)-2-phenylethyl)-8-phenyl-6,7-dihydroazepino[2,1,7-cd]indolizine

According to the procedure B, compound **4l** was obtained as a yellow thick liquid in 52% yield; **^{1}H NMR (400 MHz, CDCl$_3$)** δ 7.45 – 7.20 (m, 11H), 6.86 (s, 1H), 6.77 – 6.63 (m, 6H), 6.32 (d, J = 6.4 Hz, 1H), 5.21 (dd, J_1 = 7.6 Hz, J_2 = 5.2 Hz, 1H), 3.69 (s, 3H), 3.44 (dd, J_1 = 14.4 Hz, J_2 = 7.6 Hz, 1H), 3.25 – 3.20 (m, 3H), 3.11 (t, J = 4.0 Hz, 2H); **^{13}C NMR (101 MHz, CDCl$_3$)** δ 153.6, 152.3, 143.5, 142.0, 139.4, 135.0, 133.5, 128.4, 128.3, 127.5, 126.2, 125.9, 125.3, 124.3, 120.1, 119.4, 117.3, 117.0, 115.4, 114.4, 110.9, 110.4, 81.9, 55.6, 35.3, 34.5, 32.2; **IR (cm^{-1})**: v 3022, 2922, 2851, 1619, 1227, 1033, 699; **HRMS** (ESI) m/z calculated for $C_{33}H_{30}NO_2$ [M + H]$^+$: 472.2271, found: 472.2279.

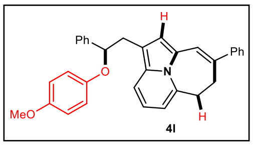

2-((1-phenyl-2-(8-phenyl-6,7-dihydroazepino[2,1,7-cd]indolizin-2-yl)ethyl)thio)benzo[d]oxazole

According to the procedure B, compound **4m** was obtained as a yellow amorphous solid in 63% yield; **^{1}H NMR (400 MHz, CDCl$_3$)** δ 7.56 (d, J = 8.0 Hz, 1H), 7.41 – 7.14 (m, 14H), 6.70 (s, 1H), 6.66 (dd, J_1 = 8.8 Hz, J_2 = 6.4 Hz, 1H), 6.46 (s, 1H), 6.27 (d, J = 6.4 Hz, 1H), 5.19 – 5.15 (m, 1H), 3.69 (dd, J_1 = 14.4 Hz, J_2 = 6.4 Hz, 1H), 3.49 (dd, J_1 = 14.4 Hz, J_2 = 8.8 Hz, 1H), 3.09 (t, J = 4.0 Hz, 2H), 2.98 (t, J = 4.0 Hz, 2H); **^{13}C NMR (101 MHz, CDCl$_3$)** δ 164.1, 151.6, 143.4, 141.9, 140.0, 139.4, 134.9, 133.6, 128.6, 128.3, 128.0, 127.9, 125.9, 125.3, 124.3, 124.1, 123.8, 119.9, 119.2, 118.5, 117.7, 115.3, 110.7, 110.5, 109.8, 52.8, 34.4, 33.6, 32.1; **IR (cm^{-1})**: v 3058, 2922, 2851, 1619, 1452, 1221, 1095, 697; **HRMS** (ESI) m/z calculated for $C_{33}H_{27}N_2OS$ [M + H]$^+$: 499.1839, found: 499.1836.

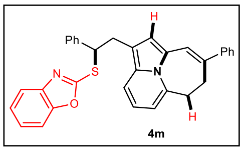

N-(1-phenyl-2-(8-phenyl-6,7-dihydroazepino[2,1,7-cd]indolizin-2-yl)ethyl)aniline

According to the procedure B, compound **4n** was obtained as a yellow amorphous solid in 72% yield; **^{1}H NMR (400 MHz, CDCl$_3$)** δ 7.44 – 7.30 (m, 7H), 7.24 – 7.19 (m, 3H), 7.15 (d, J = 8.8 Hz, 1H), 7.05 – 7.01 (m, 2H), 6.84 (s, 1H), 6.68 – 6.60 (m, 3H), 6.45 (d, J = 8.0 Hz, 2H), 6.34 (d, J = 6.4 Hz, 1H), 4.63 (dd, J_1 = 8.0 Hz, J_2 = 5.2 Hz, 1H), 4.24 (s, 1H), 3.31 – 3.11 (m, 6H); **^{13}C NMR (101 MHz, CDCl$_3$)** δ 147.4, 144.0, 143.4, 139.6, 135.0, 134.1, 128.9, 128.5, 128.3, 126.9, 126.4, 126.1, 125.4, 124.7, 119.5, 119.2, 117.7, 117.3, 115.0, 113.7, 110.6, 110.1, 58.7, 35.3, 34.5, 32.2; **IR (cm^{-1})**: v 3405, 3024, 2920, 2856, 1600, 1258, 1029, 697; **HRMS** (ESI) m/z calculated for $C_{32}H_{29}N_2$ [M + H]$^+$: 441.2325, found: 441.2328.

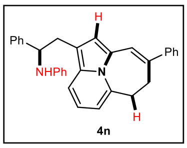

2-(2-azido-2-phenylethyl)-8-phenyl-6,7-dihydroazepino[2,1,7-cd]indolizine

According to the procedure B, compound **4o** was obtained as a yellow thick liquid in 71% yield; **^{1}H NMR (400 MHz, CDCl$_3$)** δ 7.47 – 7.33 (m, 9H), 7.26 – 7.22 (m, 2H), 6.89 (s, 1H), 6.72 – 6.68 (m, 2H), 6.35 (d, J = 6.8 Hz, 1H), 4.73 (dd, J_1 = 8.0 Hz, J_2 = 6.8 Hz, 1H), 3.30 – 3.17 (m, 4H), 3.11 (t, J = 3.2 Hz, 2H); **^{13}C NMR (101 MHz, CDCl$_3$)** δ 143.4, 139.7, 139.6, 134.8, 134.0, 128.7, 128.3, 128.2, 126.9, 126.0, 125.3, 124.6, 119.6, 119.2, 117.7, 115.0, 110.6, 110.1, 67.4, 34.5, 33.2, 32.2; **IR (cm^{-1})**: v 3028, 2922, 2855, 2096, 1620, 1258, 698; **HRMS** (ESI) m/z calculated for $C_{26}H_{23}N_4$ [M + H]$^+$: 391.1917, found: 391.1918.

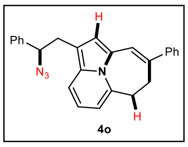

8-phenyl-2-(2-phenyl-2-(4-(p-tolyl)-2H-1,2,3-triazol-2-yl)ethyl)-6,7-dihydroazepino[2,1,7-cd]indolizine

According to the procedure B, compound **4p** was obtained as a yellow amorphous solid in 56% yield; **^{1}H NMR (400 MHz, CDCl$_3$)** δ 7.81 (s, 1H), 7.71 – 7.68 (m, 2H), 7.50 – 7.47 (m, 2H), 7.40 – 7.26 (m, 8H), 7.23 – 7.18 (m, 3H), 6.76 (s, 1H), 6.65 (t, J = 7.6 Hz, 1H), 6.49 (s, 1H), 6.30 (d, J = 6.4 Hz, 1H), 5.94 (dd, J_1 = 9.2 Hz, J_2 = 5.2 Hz, 1H), 4.11 (dd, J_1 = 14.8 Hz, J_2 = 9.2 Hz, 1H), 3.67 (dd, J_1 = 14.8 Hz, J_2 = 5.2 Hz, 1H), 3.16 (t, J = 4.0 Hz, 2H), 3.06 (t, J = 4.0 Hz, 2H), 2.38 (s, 3H); **^{13}C NMR (101 MHz, CDCl$_3$)** δ 147.5, 143.5, 139.8, 139.5, 138.1, 134.7, 133.8, 130.7, 129.4, 128.6, 128.3, 128.1, 127.8, 127.0, 126.0, 125.9, 125.3, 124.6, 119.3, 119.3, 117.6, 115.1, 110.5, 110.2, 70.6, 34.5, 32.2, 21.3; **IR (cm^{-1})**: v 3033, 2924, 2853, 1624, 1449, 1224, 695; **HRMS** (ESI) m/z calculated for $C_{35}H_{30}N_4$ [M]: 506.2470, found: 506.2470.

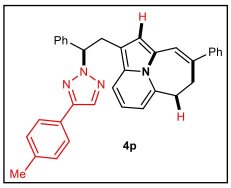

8-phenyl-2-(2-phenyl-2-(5-phenyl-2H-tetrazol-2-yl)ethyl)-6,7-dihydroazepino[2,1,7-cd]indolizine

According to the procedure B, compound **4q** was obtained as a yellow amorphous solid in 66% yield; **^{1}H NMR (400 MHz, CDCl$_3$)** δ 8.19 – 8.16 (m, 2H), 7.58 – 7.56 (m, 2H), 7.51 – 7.20 (m, 12H), 6.78 (s, 1H), 6.67 (dd, J_1 = 8.8 Hz, J_2 = 6.8 Hz, 1H), 6.54 (s, 1H), 6.31 (d, J = 6.8 Hz, 1H), 6.22 (dd, J_1 = 8.8 Hz, J_2 = 5.2 Hz, 1H), 4.16 (dd, J_1 = 14.8 Hz, J_2 = 9.6 Hz, 1H), 3.77 (dd, J_1 = 14.8 Hz, J_2 = 5.2 Hz, 1H), 3.17 (t, J = 4.4 Hz, 2H), 3.06 (t, J = 4.4 Hz, 2H); **^{13}C NMR (101 MHz, CDCl$_3$)** δ 164.9, 143.5, 139.6, 138.0, 134.7, 134.2, 130.2, 128.9, 128.8, 128.8, 128.4, 127.7, 127.3, 126.9, 126.1, 125.4, 124.8, 119.2, 119.1, 118.0, 114.8, 110.7, 109.0, 69.7, 34.5, 32.2, 32.2; **IR (cm^{-1})**: v 3032, 2924, 2853, 1628, 1232, 695; **HRMS** (ESI) m/z calculated for $C_{33}H_{28}N_5$ [M + H]$^+$: 494.2339, found: 494.2333.

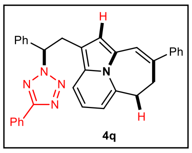

2-(1-phenyl-2-(8-phenyl-6,7-dihydroazepino[2,1,7-cd]indolizin-2-yl)ethyl)malononitrile

According to the procedure B, compound **4r** was obtained as a yellow amorphous solid in 60% yield; **^{1}H NMR (400 MHz, CDCl$_3$)** δ 7.50 – 7.42 (m, 7H), 7.41 – 7.36 (m, 2H), 7.30 (d, J = 8.4 Hz, 1H), 7.27 – 7.23 (m, 1H), 6.87 (s, 1H), 6.77 (dd, J_1 = 8.4 Hz, J_2 = 6.4 Hz, 1H), 6.73 (s, 1H), 6.42 (d, J = 6.4 Hz, 1H), 4.02 – 4.01 (m, 1H), 3.53 – 3.44 (m, 2H), 3.41 – 3.35 (m, 1H), 3.25 (t, J = 4.4 Hz, 2H), 3.13 (, J = 4.4 Hz t, 2H); **^{13}C NMR (101 MHz, CDCl$_3$)** δ 143.2, 139.9, 136.8, 135.2, 134.7, 129.1, 129.0, 128.4, 128.0, 126.4, 125.4, 125.3, 118.8, 118.8, 118.6, 114.7, 112.4, 111.7, 111.1, 108.8, 48.1, 34.5, 32.3, 28.8, 28.3; **IR (cm^{-1})**: v 3033, 2924, 2855, 2241, 1619, 1210, 700; **HRMS** (ESI) m/z calculated for $C_{29}H_{24}N_3$ [M + H]$^+$: 414.1965, found: 414.1961.

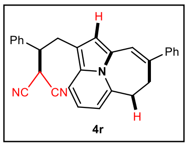

2-(2-(5-methoxy-1H-indol-3-yl)-2-phenylethyl)-8-phenyl-6,7-dihydroazepino[2,1,7-cd]indolizine

According to the procedure B, compound **4s** was obtained as a yellow amorphous solid in 51% yield; 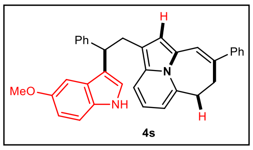

^{1}H NMR (400 MHz, CDCl$_3$) δ 7.83 (s, 1H), 7.42 – 7.32 (m, 6H), 7.28 – 7.16 (m, 6H), 7.05 (d, J = 1.6 Hz, 1H), 6.83 – 6.79 (m, 3H), 6.61 (dd, J_1 = 8.8 Hz, J_2 = 6.4 Hz, 1H), 6.51 (s, 1H), 6.29 (d, J = 6.4 Hz, 1H), 4.53 (t, J = 7.6 Hz, 1H), 3.73 (s, 3H), 3.64 (dd, J_1 = 14.4 Hz, J_2 = 6.4 Hz, 1H), 3.44 (dd, J_1 = 14.4 Hz, J_2 = 8.0 Hz, 1H), 3.18 (t, J = 4.0 Hz, 2H), 3.07 (t, J = 4.0 Hz, 2H); **^{13}C NMR (101 MHz, CDCl$_3$)** δ 153.6, 145.1, 143.5, 139.4, 134.5, 133.3, 131.6, 128.3, 128.2, 128.1, 127.5, 126.0, 125.9, 125.3, 124.1, 122.2, 119.8, 119.6, 119.4, 117.0, 115.2, 113.7, 111.9, 111.6, 110.3, 101.5, 5.7, 44.2, 34.5, 32.5, 32.1; **IR (cm^{-1})**: v 3440, 3033, 2925, 2853, 1621, 1213, 1031, 699; **HRMS** (ESI) m/z calculated for $C_{35}H_{31}N_2O$ [M + H]$^+$: 495.2431, found: 495.2438.

1-phenyl-2-(8-phenyl-6,7-dihydroazepino[2,1,7-cd]indolizin-2-yl)ethyl 5-(2,5-dimethylphenoxy)-2,2-dimethylpentanoate

According to the procedure B, compound **4t** was obtained as a yellow thick liquid in 77% yield; 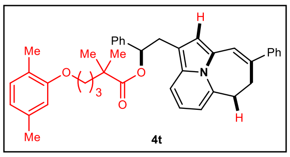

^{1}H NMR (400 MHz, CDCl$_3$) δ 7.39 – 7.16 (m, 11H), 6.97 (d, J = 7.2 Hz, 1H), 6.81 (s, 1H), 6.65 – 6.61 (m, 3H), 6.51 (s, 1H), 6.28 (d, J = 7.2 Hz, 1H), 5.97 – 5.93 (m, 1H), 3.73 – 3.63 (m, 2H), 3.35 (dd, J_1 = 14.4 Hz, J_2 = 8.0 Hz, 1H), 3.19 – 3.13 (m, 3H), 3.03 (t, J = 4.0 Hz, 2H), 2.27 (s, 3H), 2.13 (s, 3H), 1.67 – 1.57 (m, 2H), 1.52 – 1.38 (m, 2H), 1.15 (s, 3H), 1.13 (s, 3H); **^{13}C NMR (101 MHz, CDCl$_3$)** δ 176.9, 156.9, 143.4, 140.8, 139.4, 136.3, 134.9, 133.7, 130.2, 128.3, 127.7, 126.4, 125.9, 125.3, 124.3, 123.5, 120.5, 119.8, 119.1, 117.5, 115.2, 111.8, 110.5, 109.8, 76.4, 67.8, 42.0, 37.2, 34.4, 33.2, 32.1, 25.1, 25.0, 24.9, 21.4, 15.8; **IR (cm^{-1})**: v 3034, 2926, 2855, 1736, 1637, 1255, 1033, 699; **HRMS** (ESI) m/z calculated for $C_{41}H_{44}NO_3$ [M + H]$^+$: 598.3316, found: 598.3306.

1-phenyl-2-(8-phenyl-2a1,7-dihydro-6H-benzo[cd]azulen-2-yl)ethyl 2-(1-(4-chlorobenzoyl)-5-methoxy-2-methyl-1H-indol-3-yl)acetate

According to the procedure B, compound **4u** was obtained as a yellow amorphous solid in 82% yield; 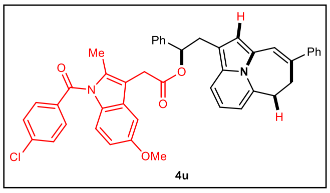

^{1}H NMR (400 MHz, CDCl$_3$) δ 7.62 – 7.59 (m, 2H), 7.46 – 7.39 (m, 6H), 7.35 – 7.34 (m, 4H), 7.29 – 7.26 (m, 2H), 7.20 (d, J = 8.0 Hz, 1H), 7.00 – 6.95 (m, 2H), 6.75 – 6.66 (m, 3H), 6.49 (d, J = 2.8 Hz, 1H), 6.35 (d, J = 6.8 Hz, 1H), 6.10 – 6.06 (m, 1H), 3.78 (s, 3H), 3.71 (s, 2H), 3.43 – 3.36 (m, 1H), 3.28 – 3.23 (m, 1H), 3.20 (t, J = 4.0 Hz, 2H), 3.08 (t, J = 4.0 Hz, 2H), 2.29 (s, 3H); **^{13}C NMR (101 MHz, CDCl$_3$)** δ 169.9, 168.1, 155.9, 143.3, 140.2, 139.3, 139.0, 135.5, 134.8, 133.8, 133.7, 131.0, 130.7, 130.5, 128.9, 128.2, 128.2, 127.9, 126.4, 125.9, 125.3, 124.3, 119.7, 119.0, 117.5, 114.9, 114.9, 112.5, 111.8, 110.4, 109.4, 101.0, 77.1 , 55.5, 34.3, 33.0, 32.0, 30.5, 13.3; **IR (cm^{-1})**: v 3059, 2929, 2839, 1734, 1683, 1223, 1014, 698; **HRMS** (ESI) m/z calculated for $C_{46}H_{39}ClNO_4$ [M + H]$^+$: 704.2562, found: 704.2561.

(8*R*,9*S*,13*S*)-13-methyl-3-(1-phenyl-2-(8-phenyl-6,7-dihydroazepino[2,1,7-cd]indolizin-2-yl)ethoxy)-6,7,8,9,11,12,13,14,15,16-decahydro-17H-cyclopenta[a]phenanthren-17-one

According to the procedure B, compound **4v** was obtained as a yellow amorphous solid in 46% yield; **^{1}H NMR (400 MHz, CDCl$_3$)** δ 7.45 – 7.30 (m, 8H), 7.28 – 7.20 (m, 3H), 7.06 (dd, J_1 = 8.4 Hz, J_2 = 4.0 Hz, 1H), 6.86 (s, 1H), 6.72 (s, 1H), 6.68 – 6.57 (m, 3H), 6.32 (d, J = 6.4 Hz, 1H), 5.30 – 5.25 (m, 1H), 3.43 (dd, J_1 = 14.8 Hz, J_2 = 7.6 Hz, 1H), 3.24 – 3.19 (m, 3H), 3.10 (t, J = 4.0 Hz, 2H), 2.81 – 2.75 (m, 2H), 2.48 (dd, J_1 = 19.2 Hz, J_2 = 8.4 Hz, 1H), 2.33 – 2.27 (m, 1H), 2.19 – 1.90 (m, 5H), 1.59 – 1.27 (m, 6H), 0.86 (s, 3H); **^{13}C NMR (101 MHz, CDCl$_3$)** δ 156.1, 156.1, 143.5, 142.1, 142.0, 139.4, 137.5, 137.4, 135.0, 135.0, 133.5, 131.8, 131.8, 128.5, 128.3, 127.4, 126.1, 126.0, 125.9, 125.3, 124.3, 120.1, 119.4, 117.3, 116.0, 115.7, 115.4, 115.4, 113.3, 113.0, 110.9, 110.4, 80.9, 80.8, 50.3, 47.9, 43.9, 43.9, 38.2, 38.2, 35.8, 35.4, 35.4, 34.5, 32.2, 31.5, 29.6, 29.5, 26.5, 26.4, 25.8, 25.7, 21.5, 13.8; **IR (cm^{-1})**: *v* 3057, 2921, 2855, 1728, 1618, 1221, 1047, 698; **HRMS** (ESI) m/z calculated for $C_{44}H_{44}NO_2$ [M + H]$^+$: 618.3367, found: 618.3371.

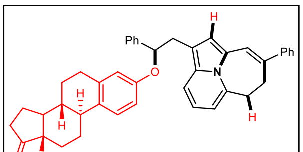

4v

N-(1-phenyl-2-(8-phenyl-6,7-dihydroazepino[2,1,7-cd]indolizin-2-yl)ethyl)-6-(trifluoromethoxy)benzo[d]thiazol-2-amine

According to the procedure B, compound **4w** was obtained as a yellow amorphous solid in 42% yield; **^{1}H NMR (400 MHz, CDCl$_3$)** δ 7.43 – 7.32 (m, 11H), 7.26 – 7.21 (m, 1H), 7.11 – 7.08 (m, 2H), 6.81 (s, 1H), 6.66 (t, J = 7.6 Hz, 1H), 6.58 (s, 1H), 6.35 (d, J = 7.6 Hz, 1H), 6.22 (s, 1H), 4.95 (t, J = 6.4 Hz, 1H), 3.41 (dd, J_1 = 14.4 Hz, J_2 = 5.2 Hz, 1H), 3.30 (dd, J_1 = 14.4 Hz, J_2 = 6.4 Hz, 1H), 3.20 (t, J = 4.0 Hz, 2H), 3.10 (t, J = 4.0 Hz, 2H); **^{13}C NMR (101 MHz, CDCl$_3$)** δ 167.8, 150.0, 143.5 (d, J = 1.9 Hz), 143.4, 141.0, 139.7, 134.8 (d, J = 64.3 Hz), 131.0, 128.8, 128.3, 127.9, 126.7, 126.1, 125.4, 124.9, 121.8, 119.7, 119.6, 119.3, 119.1, 118.9, 118.1, 114.8, 114.0, 110.8, 108.4, 77.3, 77.0, 76.7, 60.5, 34.5, 34.3, 32.2; **^{19}F NMR (377 MHz, CDCl$_3$)** δ -58.26 (s, 3F); IR (cm^{-1}): *v* 3423, 3033, 2944, 2855, 1610, 1460, 1255, 698; **HRMS** (ESI) m/z calculated for $C_{34}H_{27}F_3N_3OS$ [M + H]$^+$: 582.1821, found: 582.1816.

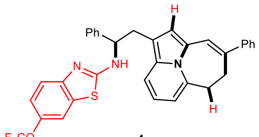

4w

1-(((cyclohexyloxy)carbonyl)oxy)ethyl 2-ethoxy-1-((2'-(1-(1-phenyl-2-(8-phenyl-6,7-dihydroazepino[2,1,7-cd]indolizin-2-yl)ethyl)-1H-tetrazol-5-yl)-[1,1'-biphenyl]-4-yl)methyl)-1H-benzo[d]imidazole-7-carboxylate

According to the procedure B, compound **4x** was obtained as a yellow amorphous solid in 63% yield; **^{1}H NMR (400 MHz, CDCl$_3$)** δ 7.76 (ddd, J_1 = 5.2 Hz, J_2 = 4.0 Hz, J_3 = 1.2 Hz, 1H), 7.60 (d, J = 7.6 Hz, 1H), 7.46 (dd, J_1 = 7.6 Hz, J_2 = 2.8 Hz, 1H), 7.39 – 7.10 (m, 13H), 7.03 – 6.95 (m, 4H), 6.82 – 6.77 (m, 3H), 6.64 (d, J = 5.2 Hz, 1H), 6.57 – 6.52 (m, 1H), 6.23 – 6.19 (m, 2H), 5.90 (dd, J_1 = 9.6 Hz, J_2 = 6.0 Hz, 1H), 5.91 – 5.47 (m, 2H), 4.57 – 4.47 (m, 3H), 3.66 (ddd, J_1 = 11.6 Hz, J_2 = 9.6 Hz, J_3 = 2.0 Hz, 1H), 3.44 – 3.36 (m, 1H), 3.08 (t, J = 4.0 Hz, 2H), 2.98 (t, J = 4.0 Hz, 2H), 1.85 – 1.77 (m, 2H), 1.65 – 1.58 (m, 2H), 1.43 – 1.09 (m, 11H), 0.81 – 0.75 (m, 1H); **^{13}C NMR (101 MHz, CDCl$_3$)** δ 164.8, 164.0, 163.9, 158.5, 152.5, 143.4, 141.4, 140.0, 140.0, 139.5, 137.8, 137.7, 135.8, 134.5, 134.1, 131.8, 130.7, 130.2, 129.8, 129.5, 129.5, 128.6, 128.5, 128.3, 127.4, 127.1, 127.1, 126.4, 126.2, 126.2, 126.0, 125.3, 124.6, 124.0, 122.4, 120.8, 119.1, 119.0, 117.8, 117.8, 114.7, 114.6, 110.6, 108.9, 91.7, 77.5, 69.3, 66.9, 47.0, 34.4, 32.1, 32.1, 32.0, 31.3, 29.7, 25.1, 23.5, 23.5, 19.5, 19.4, 14.5; **IR (cm^{-1})**: *v* 3032, 2927, 2855, 1752, 1618, 1451, 1278, 1036, 699; **HRMS** (ESI) m/z calculated for $C_{59}H_{56}N_7O_6$ [M + H]$^+$: 958.4287, found: 958.4282.

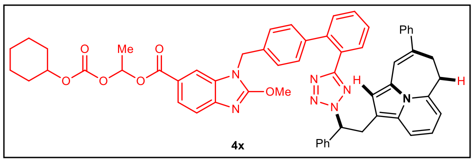

1-phenyl-2-(8-phenyl-6,7-dihydroazepino[2,1,7-cd]indolizin-2-yl)ethan-1-ol

Compound **4a-1** was obtained as a yellow amorphous solid in 96% yield; **^{1}H NMR (400 MHz, CDCl$_3$)** δ 7.44 – 7.42 (m, 4H), 7.37 – 7.32 (m, 4H), 7.30 – 7.19 (m, 3H), 6.87 (s, 1H), 6.73 (s, 1H), 6.67 (dd, J_1 = 8.8 Hz, J_2 = 6.4 Hz, 1H), 6.34 (d, J = 6.4 Hz, 1H), 4.92 (dd, J_1 = 8.8 Hz, J_2 = 4.4 Hz, 1H), 3.22 – 3.17 (m, 3H), 3.13 – 3.08 (m, 3H), 2.11 (s, 1H); **^{13}C NMR (101 MHz, CDCl$_3$)** δ 144.0, 143.4, 139.6, 135.1, 134.2, 128.4, 128.3, 127.4, 126.1, 125.8, 125.4, 124.7, 119.7, 119.1, 117.7, 115.3, 110.7, 110.2, 74.7, 36.3, 34.5, 32.2; **IR (cm^{-1})**: *v* 3423, 3033, 2928, 2853, 1619, 1295, 1024, 698; **HRMS** (ESI) m/z calculated for $C_{26}H_{24}NO$ [M + H]$^+$: 366.1852, found: 366.1853.

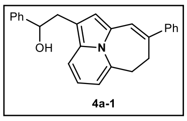

(*Z*)-1-phenyl-2-(8-phenyl-6,7-dihydroazepino[2,1,7-cd]indolizin-2-yl)vinyl acetate

Compound **4a-2** was obtained as a yellow thick liquid in 86% yield; **^{1}H NMR (400 MHz, CDCl$_3$)** δ 7.58 – 7.53 (m, 3H), 7.49 – 7.46 (m, 2H), 7.41 – 7.38 (m, 4H), 7.31 – 7.25 (m, 2H), 7.14 (s, 1H), 7.03 (s, 1H), 6.92 (s, 1H), 6.85 (dd, J_1 = 8.8 Hz, J_2 = 6.8 Hz, 1H), 6.45 (d, J = 6.8 Hz, 1H), 3.25 (t, J = 4.0 Hz, 2H), 3.13 (t, J = 4.0 Hz, 2H), 2.45 (s, 3H); **^{13}C NMR (101 MHz, CDCl$_3$)** δ 169.0, 143.3, 142.8, 139.9, 136.3, 135.8, 135.6, 128.6, 128.4, 127.4, 126.4, 126.4, 125.5, 123.8, 119.6, 119.1, 117.3, 115.1, 111.7, 108.8, 107.8, 34.4, 32.1, 21.3; **IR (cm^{-1})**: *v* 3052, 2924, 2853, 1757, 1641, 1204, 696; **HRMS** (ESI) m/z calculated for $C_{28}H_{24}NO_2$ [M + H]$^+$: 406.1802, found: 406.1811.

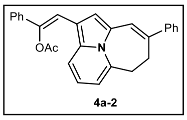

2-((tert-butyldimethylsilyl)oxy)-8-(4-methoxyphenyl)-6,7-dihydroazepino[2,1,7-cd]indolizine-1,6-d_2

According to the procedure A, compound **2a''** was obtained as a yellow thick liquid in 53% yield;

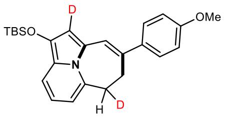

2a''

^{1}H NMR (400 MHz, $CDCl_3$) δ 7.41 – 7.38 (m, 2H), 7.30 (d, *J* = 8.8 Hz, 1H), 6.94 – 6.91 (m, 2H), 6.76 (s, 1H), 6.54 (t, *J* = 7.6 Hz, 1H), 6.44 (s, 0.1H), 6.24 (d, *J* = 6.4 Hz, 1H), 3.85 (s, 3H), 3.16 – 3.07 (m, 3H), 1.09 (s, 9H), 0.27 (s, 6H); **^{13}C NMR (101 MHz, $CDCl_3$)** δ 158.0, 138.3, 136.3, 133.5, 133.4, 126.4, 126.1, 120.7, 117.8, 114.8, 114.8, 113.7, 110.3, 108.2, 55.3, 34.4, 34.3, 34.1, 33.9, 32.2, 25.8, 18.2, -4.6; **IR (cm^{-1})**: *v* 3031, 2953, 2856, 1617, 1430, 1250, 1036, 714; **HRMS** (ESI) m/z calculated for $C_{25}H_{30}D_2NO_2Si$ $[M + H]^+$: 408.2322, found: 408.2329.

1.13 The X-ray structure of 2a, 2z, 3a, 4m

The single crystal was obtained by slow evaporation of a saturated solution in dichloromethane and petroleum ether in a lossely capped vial. Structure information was deposited at the Cambridge Crystallographic Data Center (CCDC).

Supplementary Figure 1. The X-ray crystallographic structure of **2a** (CCDC 2141700)

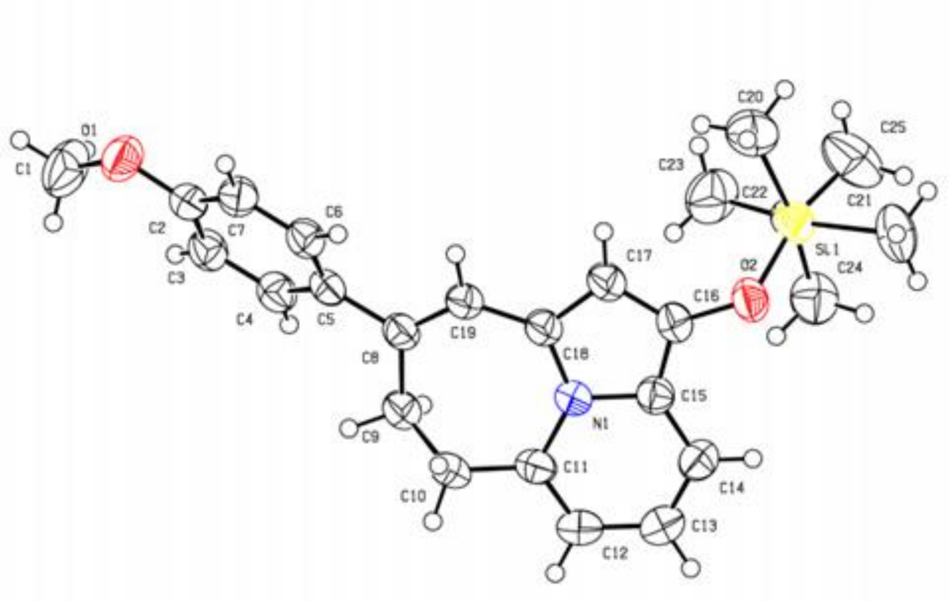

The X- ray of **2a**

The crystal **2a** was kept at 293(2) K during data collection. Using Olex2, the structure was solved with the SHELXT structure solution program using Intrinsic Phasing and refined with the SHELXL refinement package using Least Squares minimisation. The ORTEP image of compound and crystallographic refinement parameters are given below.

Crystal Data for $C_{25}H_{31}NO_2Si$ (M =405.60 g/mol): triclinic, space group P-1 (no. 2), a = 8.0760(6) Å, b = 11.8269(8) Å, c = 13.0889(12) Å, α = 95.809(7) °, β = 104.901(7) °, γ = 106.302(6) °, V = 1139.12(16) Å^3, Z = 2, T = 293(2) K, μ(Mo Kα) = 0.123 mm^{-1}, Dcalc = 1.183 g/cm^3, 8850 reflections measured (6.886° ≤ 2Θ ≤ 58.028°), 5153 unique (R_{int} = 0.0227, R_{sigma} = 0.0474) which were used in all calculations. The final R_1 was 0.0543 (I > 2σ(I)) and wR_2 was 0.1404

Supplementary Figure 2. The X-ray crystallographic structure of **2z** (CCDC 2141701)

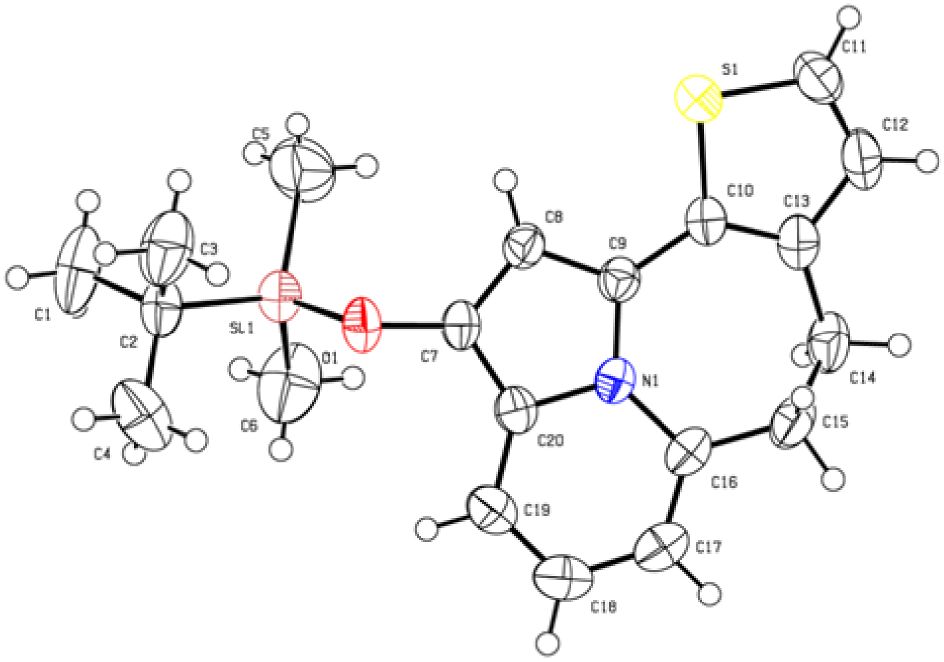

The X-ray of **2z**

The crystal **2z** was kept at 293(2) K during data collection. Using Olex2, the structure was solved with the SHELXT structure solution program using Intrinsic Phasing and refined with the SHELXL refinement package using Least Squares minimisation. The ORTEP image of compound and crystallographic refinement parameters are given below.

Crystal Data for $C_{20}H_{25}NOSSi$ (M =355.56 g/mol): monoclinic, space group $P2_1/c$ (no. 14), a = 7.7405(4) Å, b = 20.3269(14) Å, c = 12.9017(7) Å, β = 106.427(6)°, V = 1947.1(2) Å^3, Z = 4, T = 293(2) K, μ(Mo Kα) = 0.234 mm^{-1}, $Dcalc$ = 1.213 g/cm^3, 8117 reflections measured (6.796° ≤ 2Θ ≤ 58.13°), 4467 unique (R_{int} = 0.0273, R_{sigma} = 0.0522) which were used in all calculations. The final R_1 was 0.0527 ($I > 2\sigma(I)$) and wR_2 was 0.1338

Supplementary Figure 3. The X-ray crystallographic structure of **3a** (CCDC 2239536)

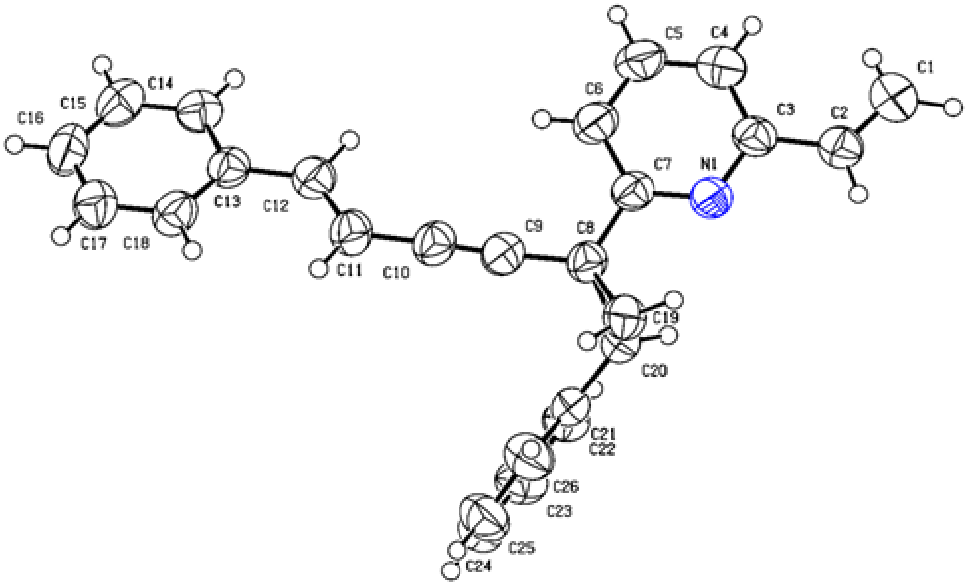

The X-ray of **3a**

The crystal **3a** was kept at 293(2) K during data collection. Using Olex2, the structure was solved with the SHELXT structure solution program using Intrinsic Phasing and refined with the SHELXL refinement package using Least Squares minimisation. The ORTEP image of compound and crystallographic refinement parameters are given below.

Crystal Data for $C_{26}H_{21}N$ (M =347.44 g/mol): monoclinic, space group $P2_1/n$ (no. 14), a = 5.5140(3) Å, b = 9.4541(5) Å, c = 37.289(2) Å, β = 92.380(5)°, V = 1942.20(18) Å^3, Z = 4, T = 293(2) K, μ(Mo Kα) = 0.068 mm^{-1}, $Dcalc$ = 1.188 g/cm^3, 8202 reflections measured (6.962° ≤ 2Θ ≤ 58.254°), 4368 unique (R_{int} = 0.0184, R_{sigma} = 0.0368) which were used in all calculations. The final R_1 was 0.0569 (I > 2σ(I)) and wR_2 was 0.1475 (all data).

Supplementary Figure 4. The X-ray crystallographic structure of **4m** (CCDC 2141703)

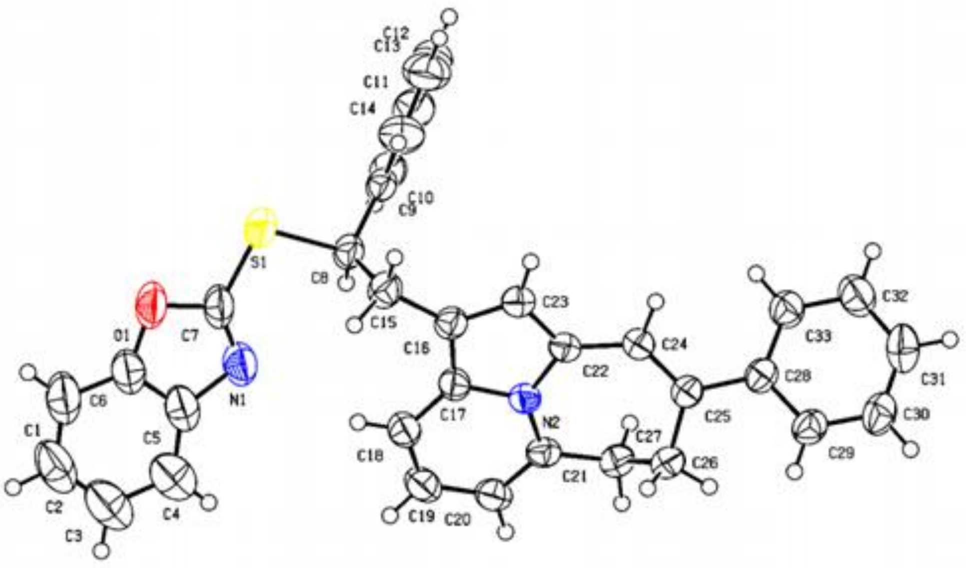

The X-ray of **4m**

The crystal **4m** was kept at 293(2) K during data collection. Using Olex2, the structure was solved with the SHELXT structure solution program using Intrinsic Phasing and refined with the SHELXL refinement package using Least Squares minimisation. The ORTEP image of compound and crystallographic refinement parameters are given below.

Crystal Data for $C_{33}H_{26}N_2OS$ (M =498.62 g/mol): monoclinic, space group P2$_1$/c (no. 14), a = 8.8644(3) Å, b = 9.5173(3) Å, c = 30.8497(9) Å, β = 90.765(3) °, V = 2602.41(14) Å^3, Z = 4, T = 293(2) K, μ(Mo Kα) = 0.154 mm^{-1}, $Dcalc$ = 1.273 g/cm^3, 22594 reflections measured (6.79° ≤ 2Θ ≤ 58.204°), 6201 unique (R_{int} = 0.0342, R_{sigma} = 0.0372) which were used in all calculations. The final R_1 was 0.0567 (I > 2σ(I)) and wR_2 was 0.1483 (all data).

1.14 Copies of NMR spectra

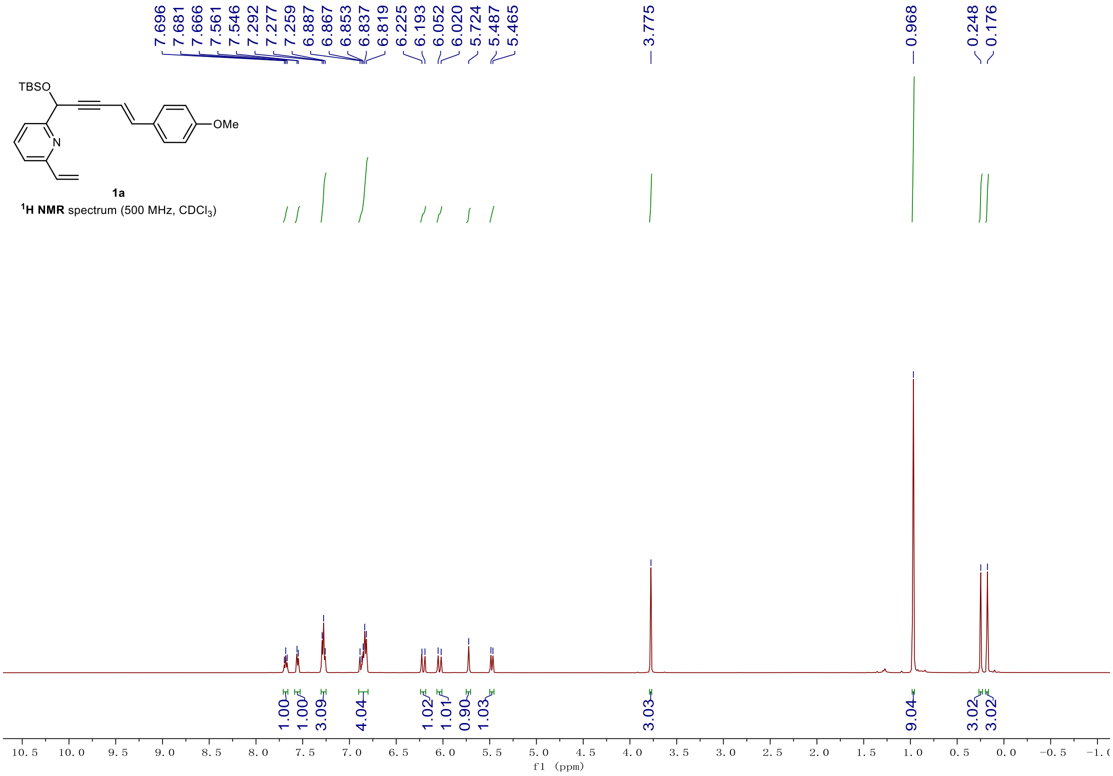

Supplementary Figure 5. ^{1}H NMR (500 MHz, $CDCl_3$) spectra for **1a**

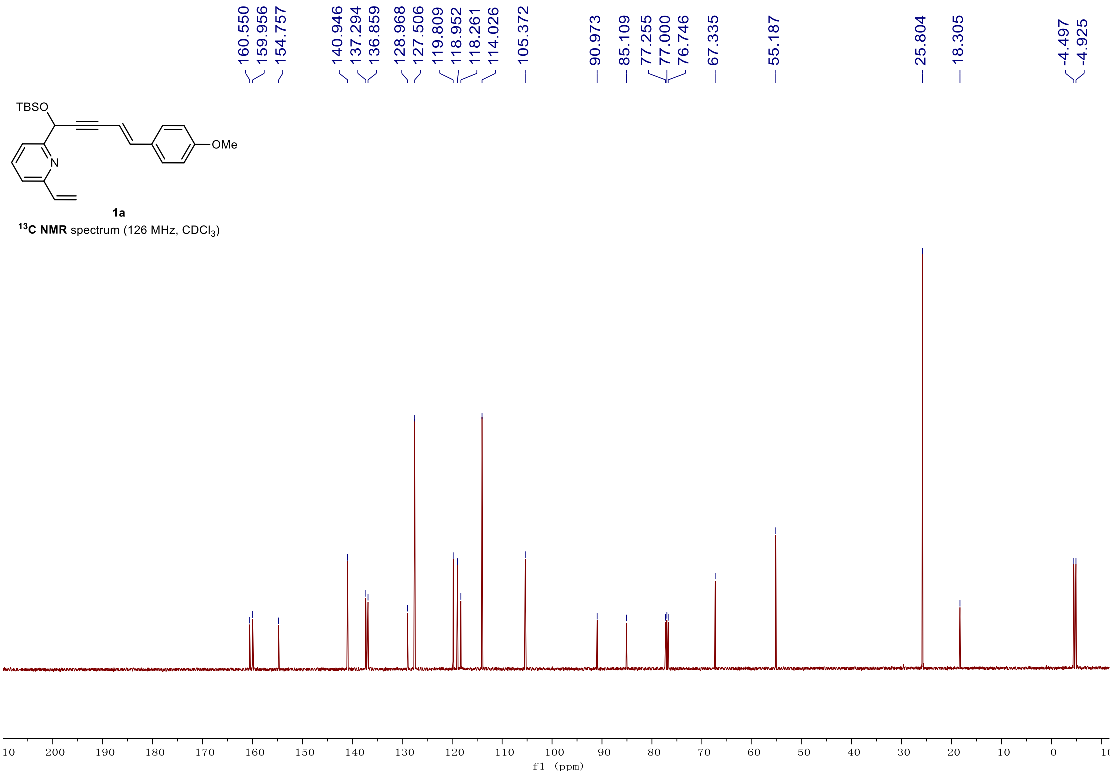

Supplementary Figure 6. ^{13}C NMR (126 MHz, $CDCl_3$) spectra for **1a**

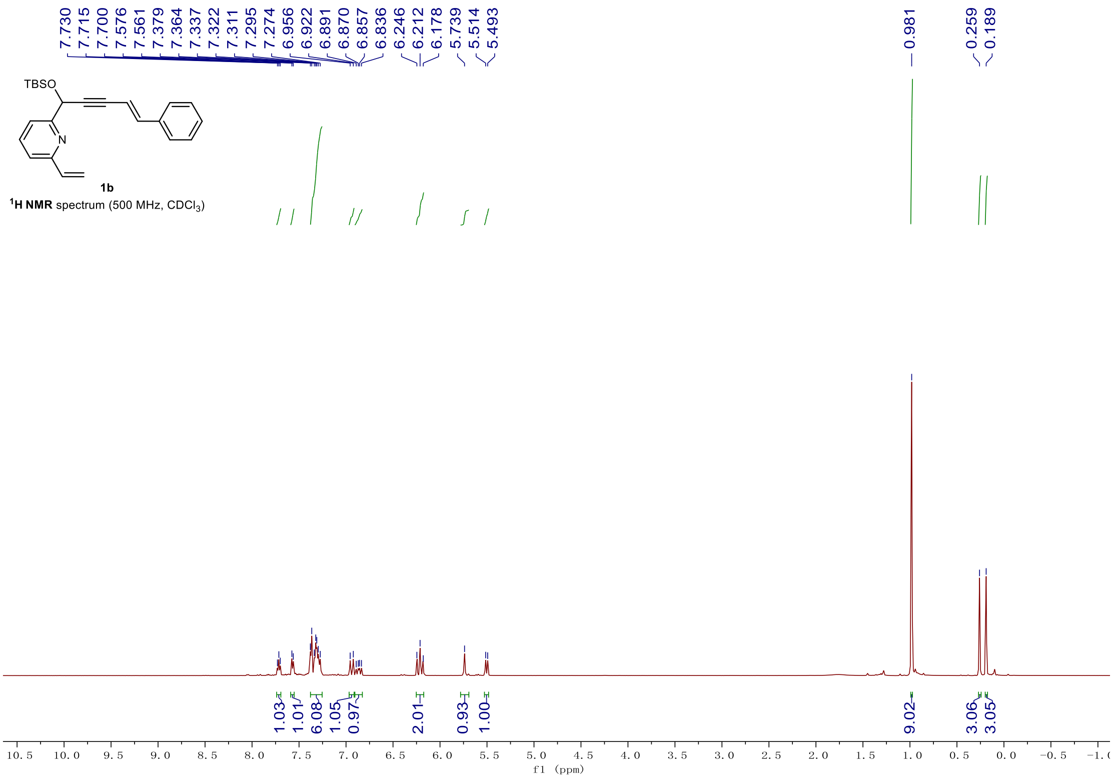

Supplementary Figure 7. ^{1}H NMR (500 MHz, $CDCl_3$) spectra for **1b**

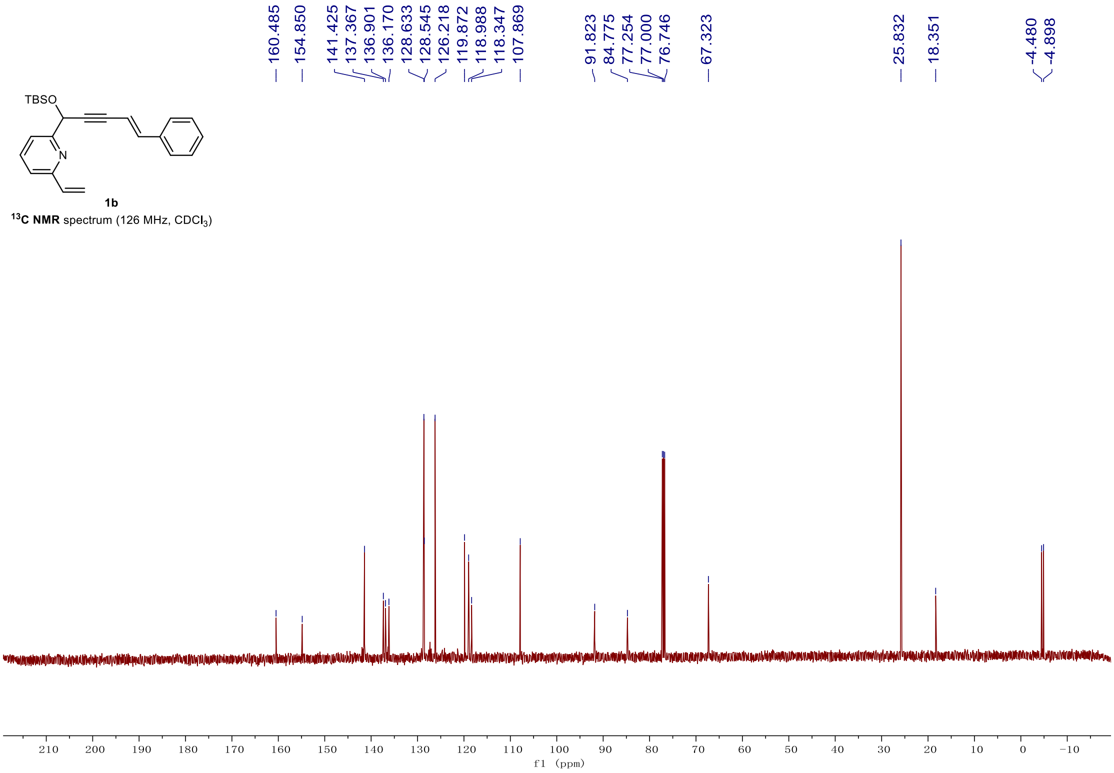

Supplementary Figure 8. ^{13}C NMR (126 MHz, $CDCl_3$) spectra for **1b**

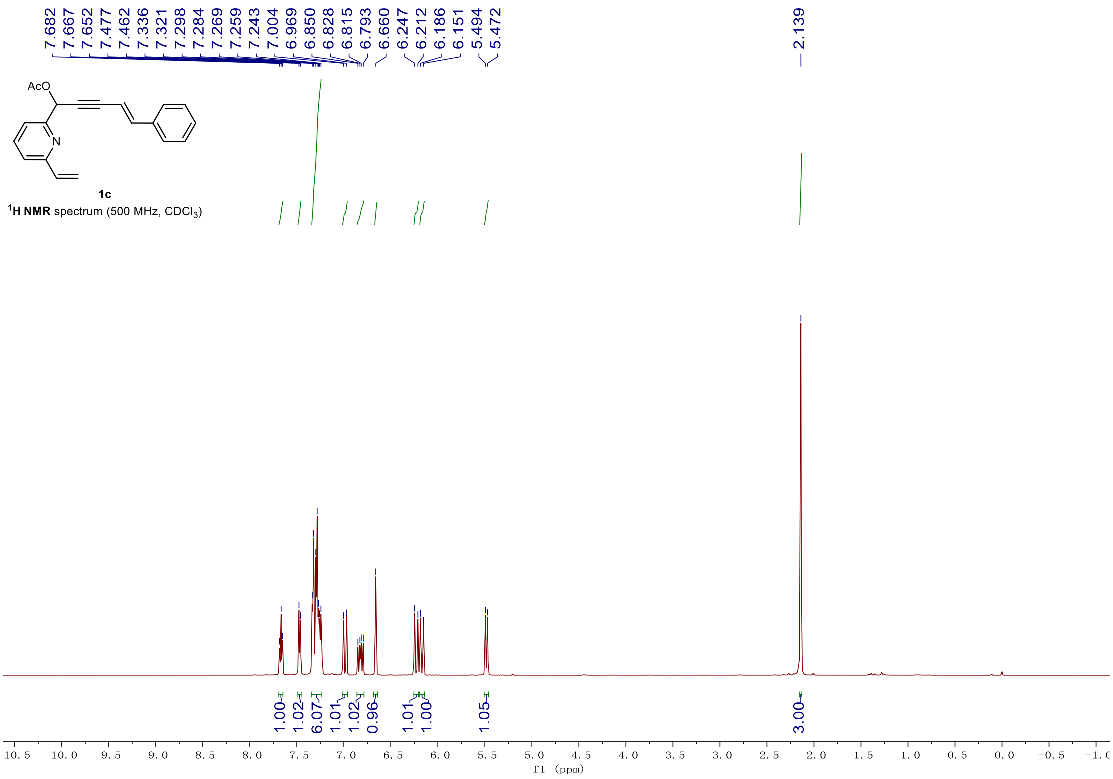

Supplementary Figure 9. ^{1}H NMR (500 MHz, $CDCl_3$) spectra for **1c**

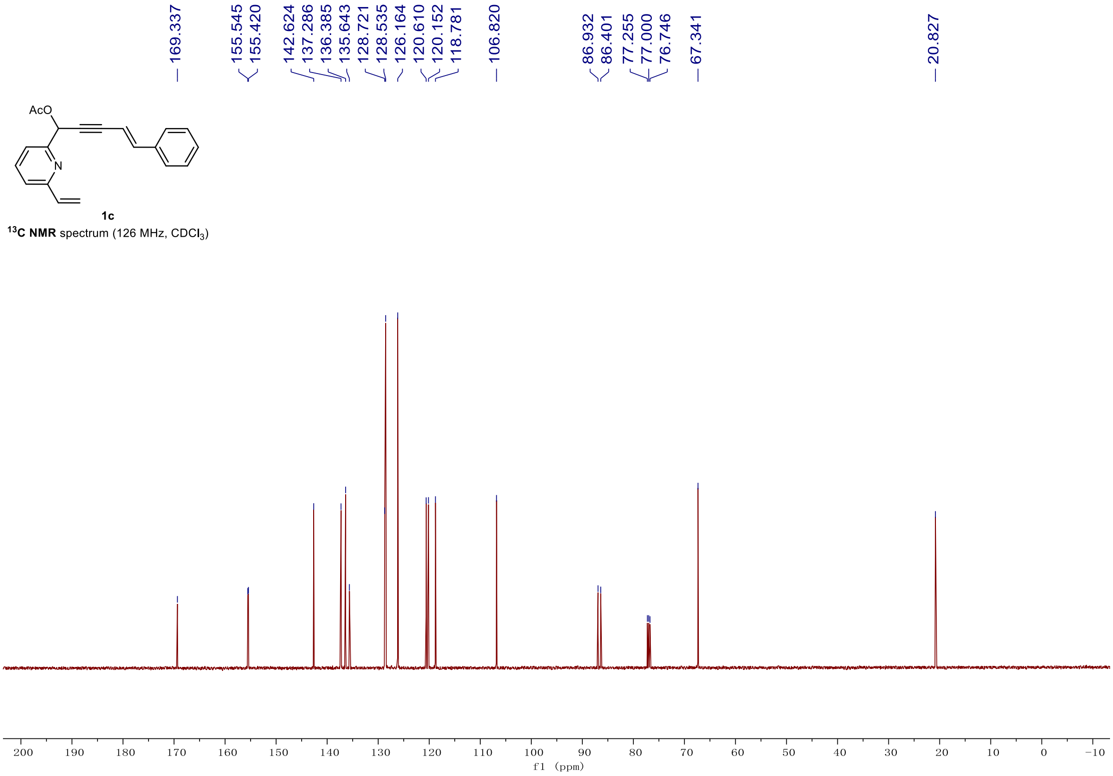

Supplementary Figure 10. ^{13}C NMR (126 MHz, $CDCl_3$) spectra for **1c**

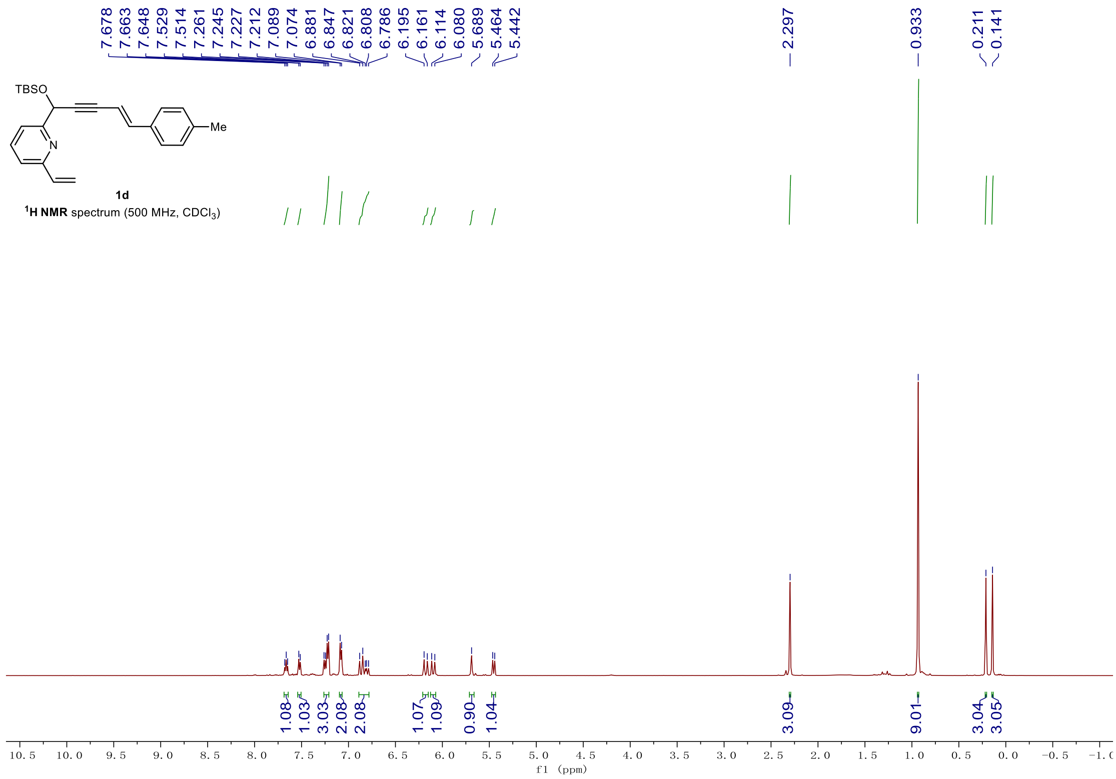

Supplementary Figure 11. ^{1}H NMR (500 MHz, $CDCl_3$) spectra for **1d**

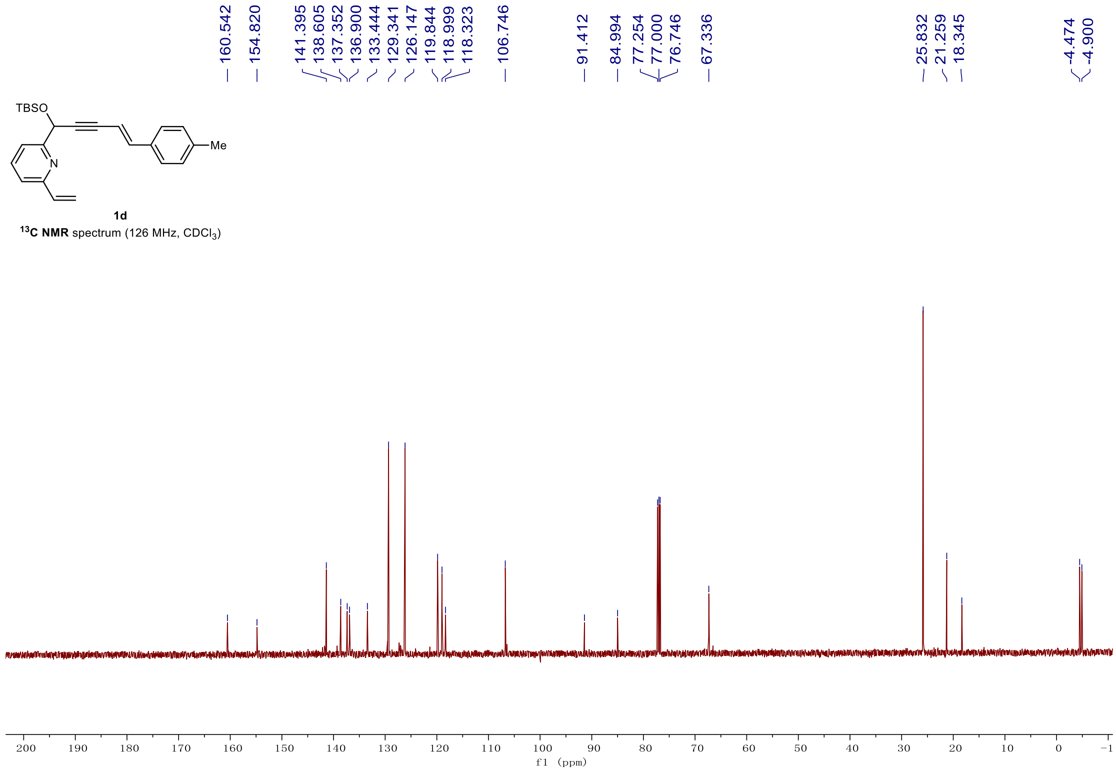

Supplementary Figure 12. ^{13}C NMR (126 MHz, $CDCl_3$) spectra for **1d**

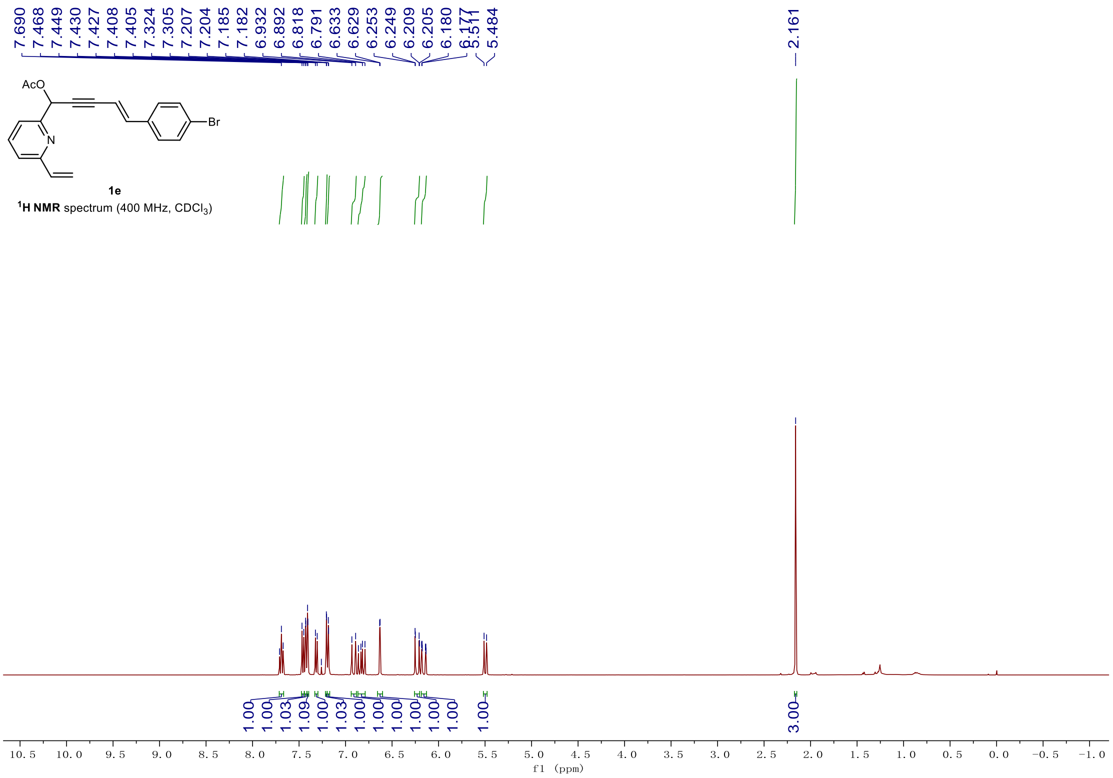

Supplementary Figure 13. ^{1}H NMR (400 MHz, $CDCl_3$) spectra for **1e**

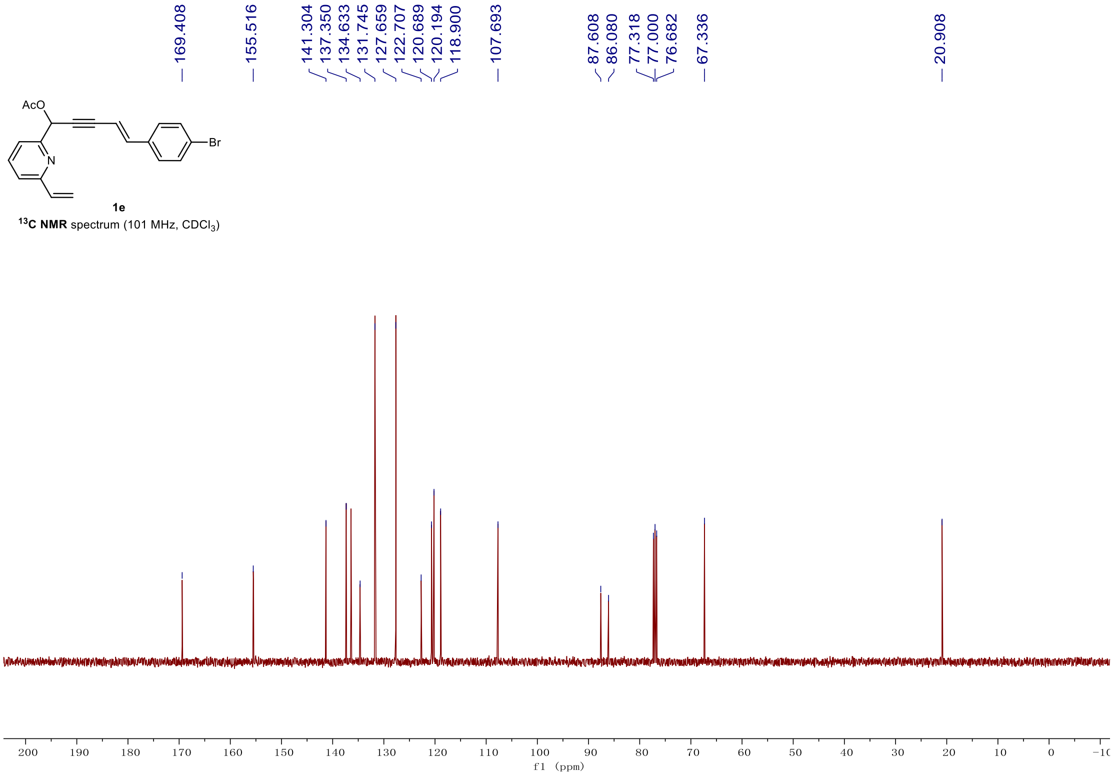

Supplementary Figure 14. ^{13}C NMR (101 MHz, $CDCl_3$) spectra for **1e**

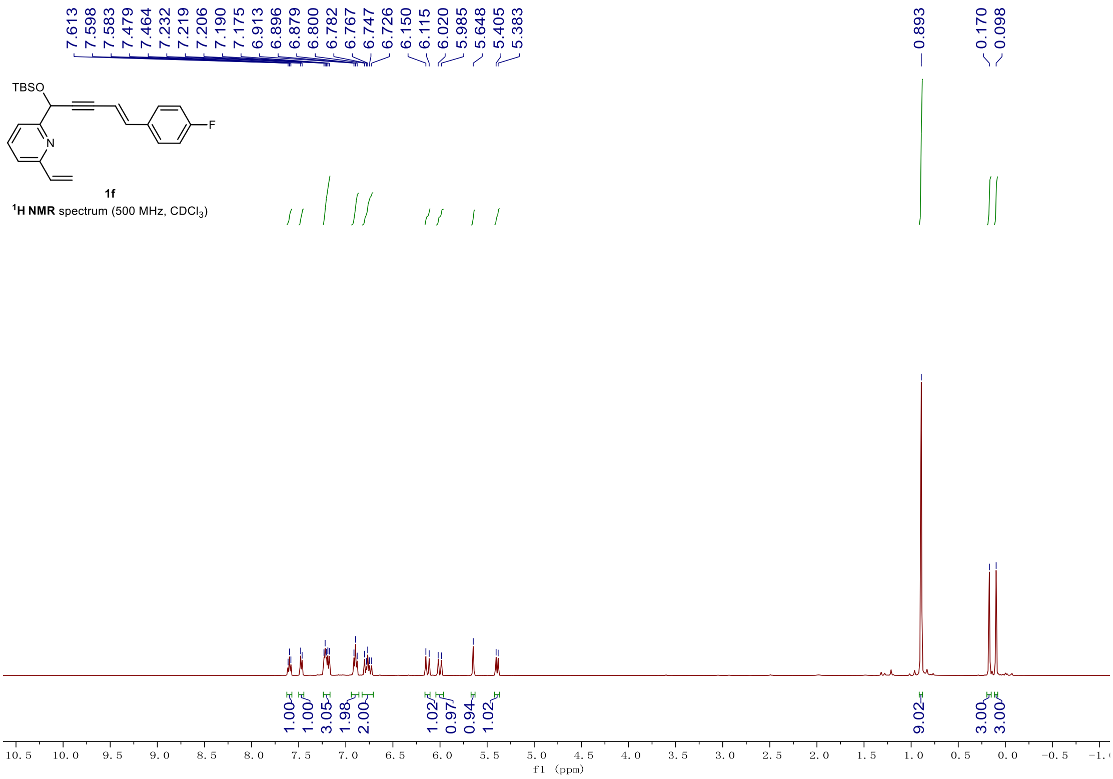

Supplementary Figure 15. ^{1}H NMR (500 MHz, $CDCl_3$) spectra for **1f**

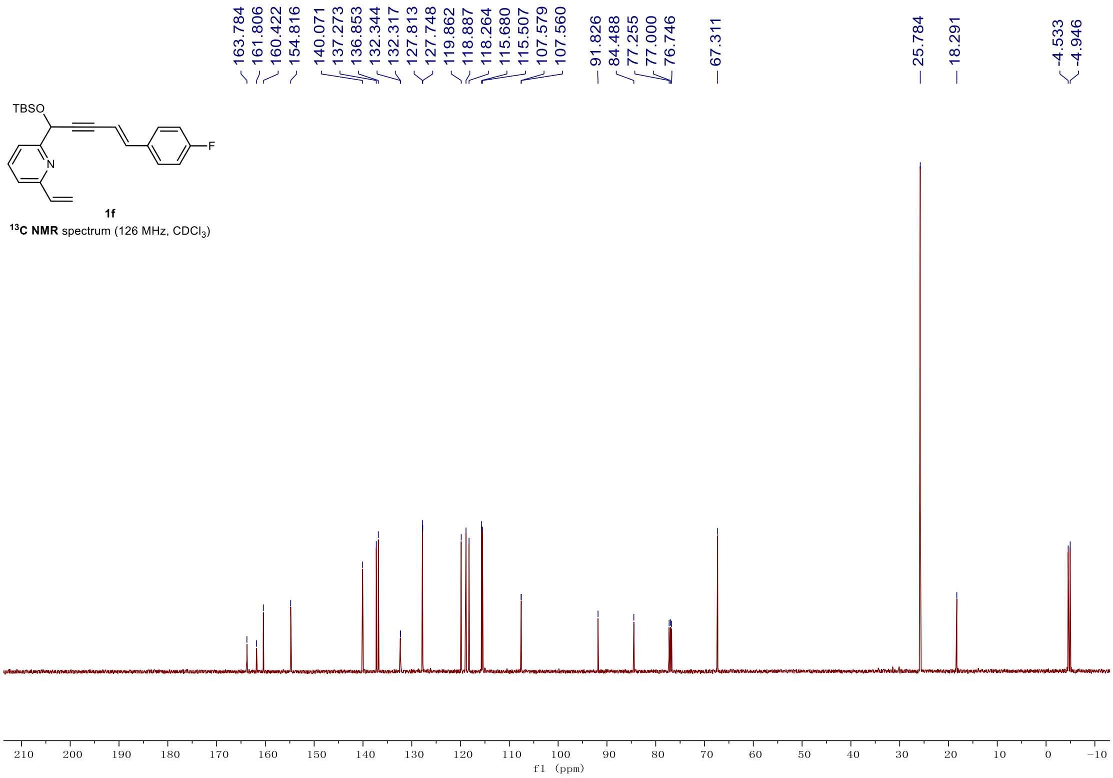

Supplementary Figure 16. ^{13}C NMR (126 MHz, $CDCl_3$) spectra for **1f**

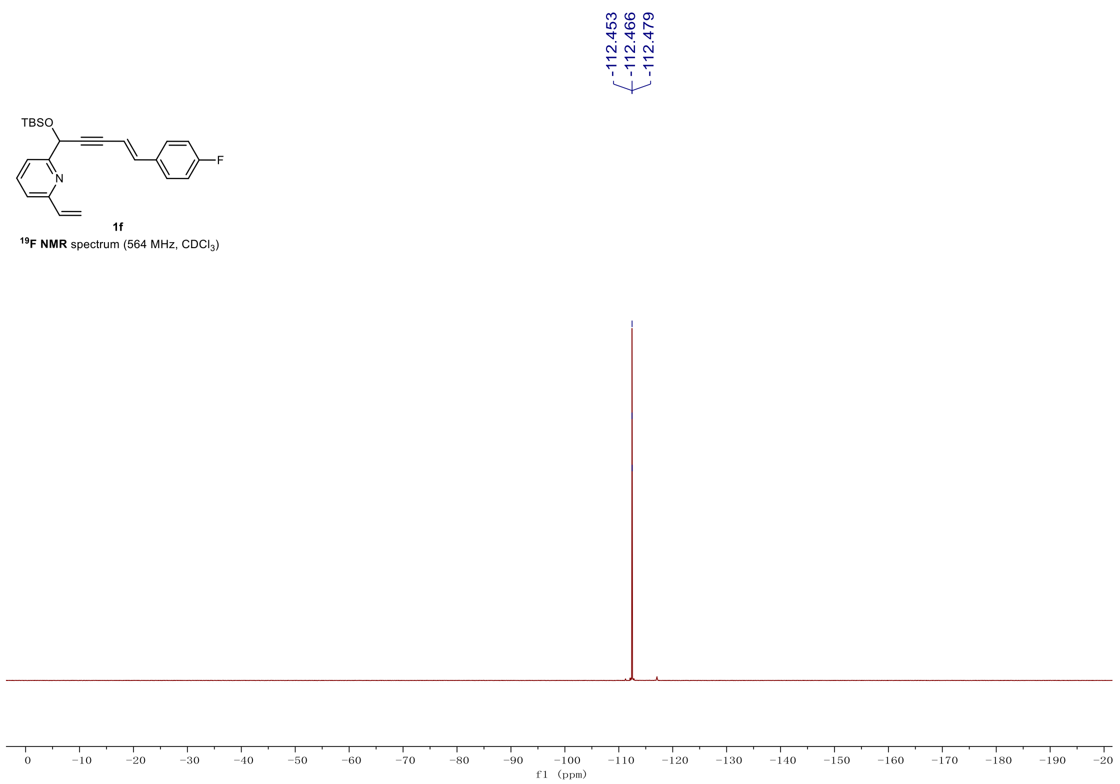

Supplementary Figure 17. ^{19}F NMR (564 MHz, $CDCl_3$) spectra for **1f**

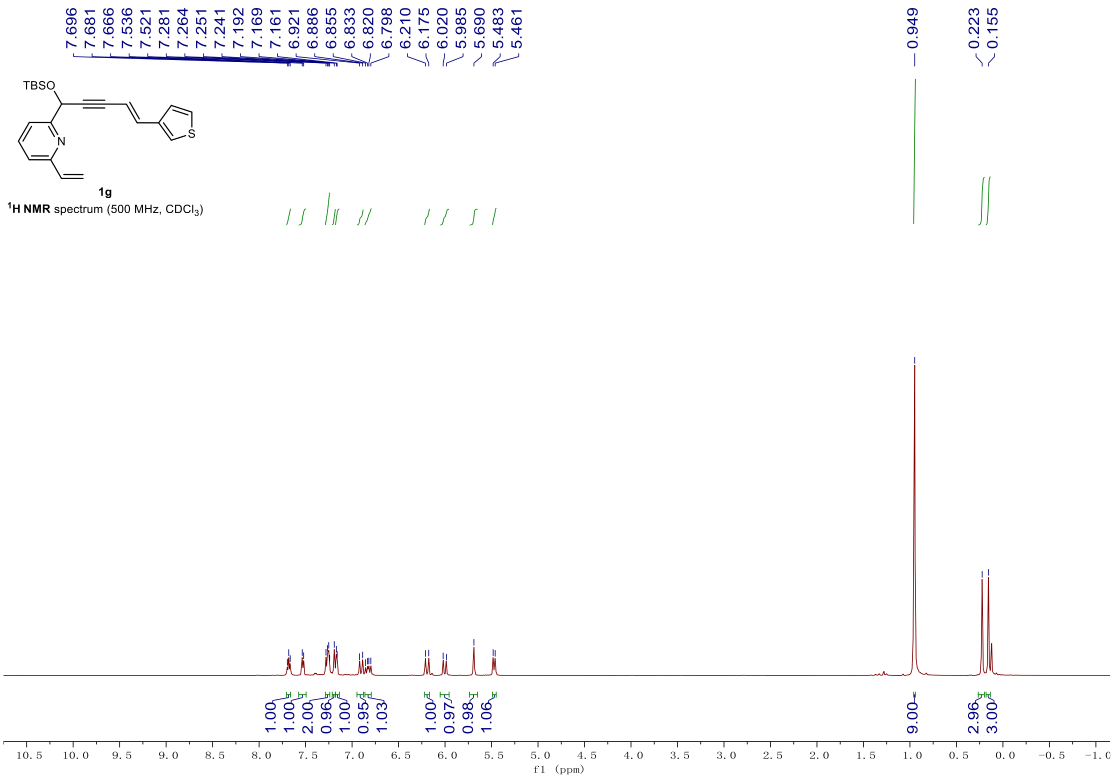

Supplementary Figure 18. ^{1}H NMR (500 MHz, $CDCl_3$) spectra for **1g**

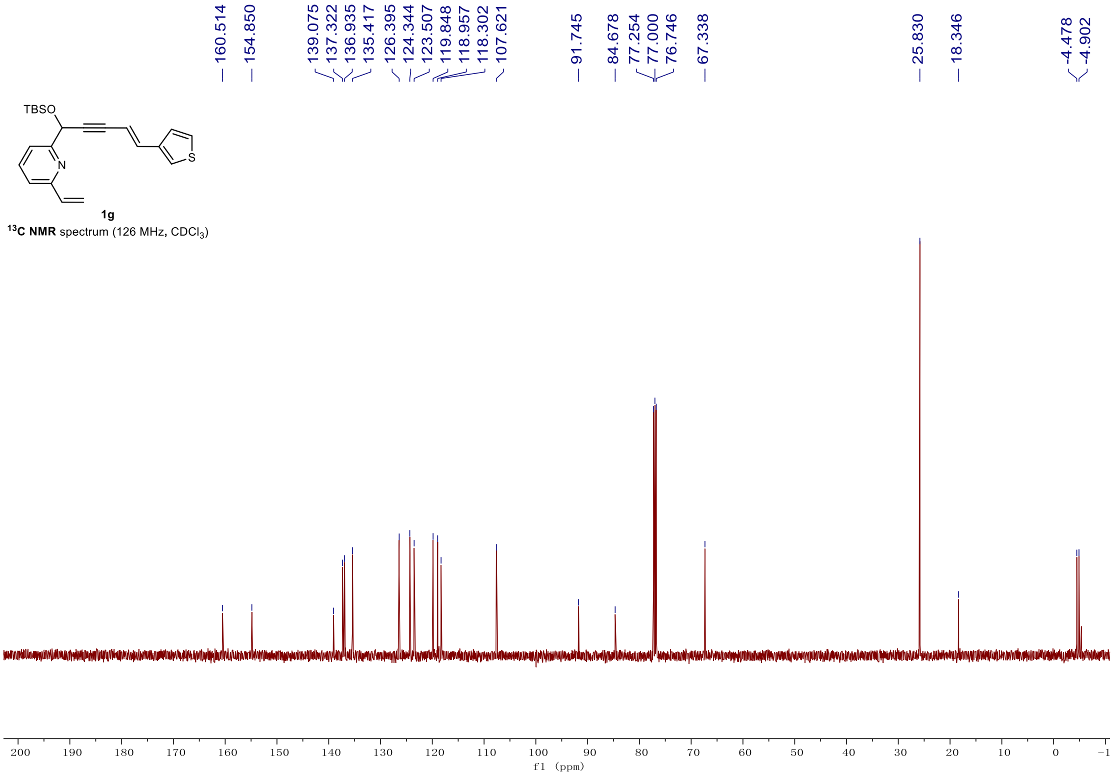

Supplementary Figure 19. ^{13}C NMR (126 MHz, $CDCl_3$) spectra for **1g**

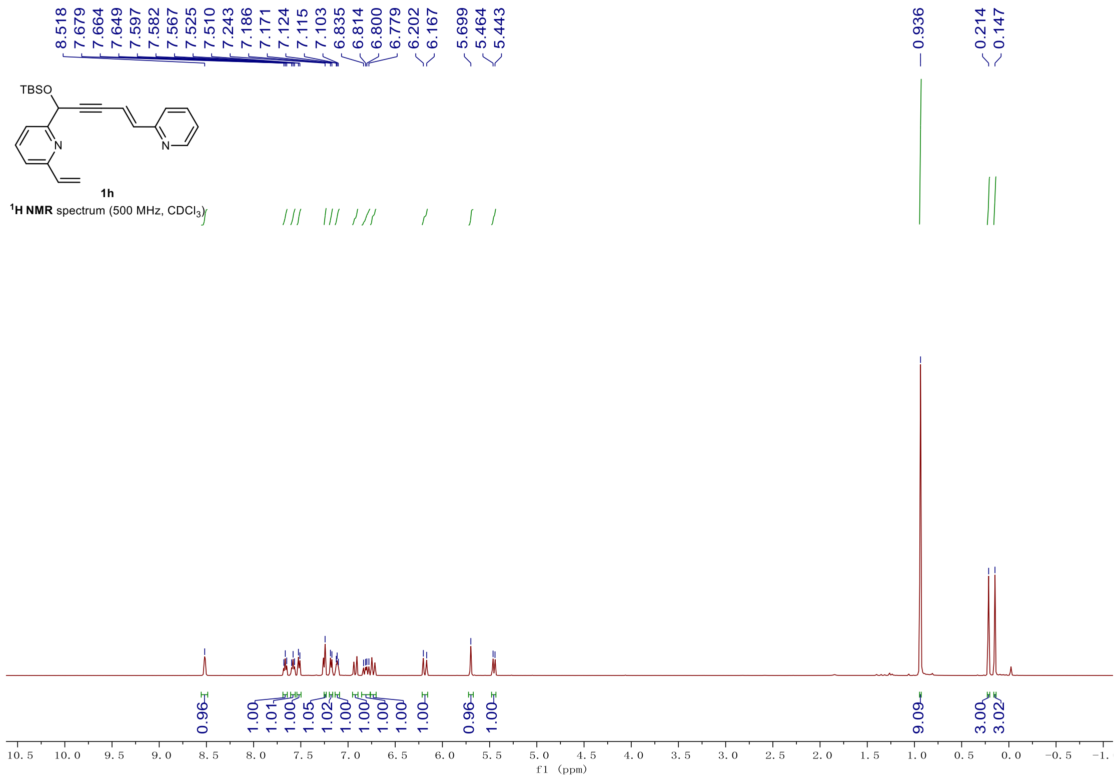

Supplementary Figure 20. ^{1}H NMR (500 MHz, $CDCl_3$) spectra for **1h**

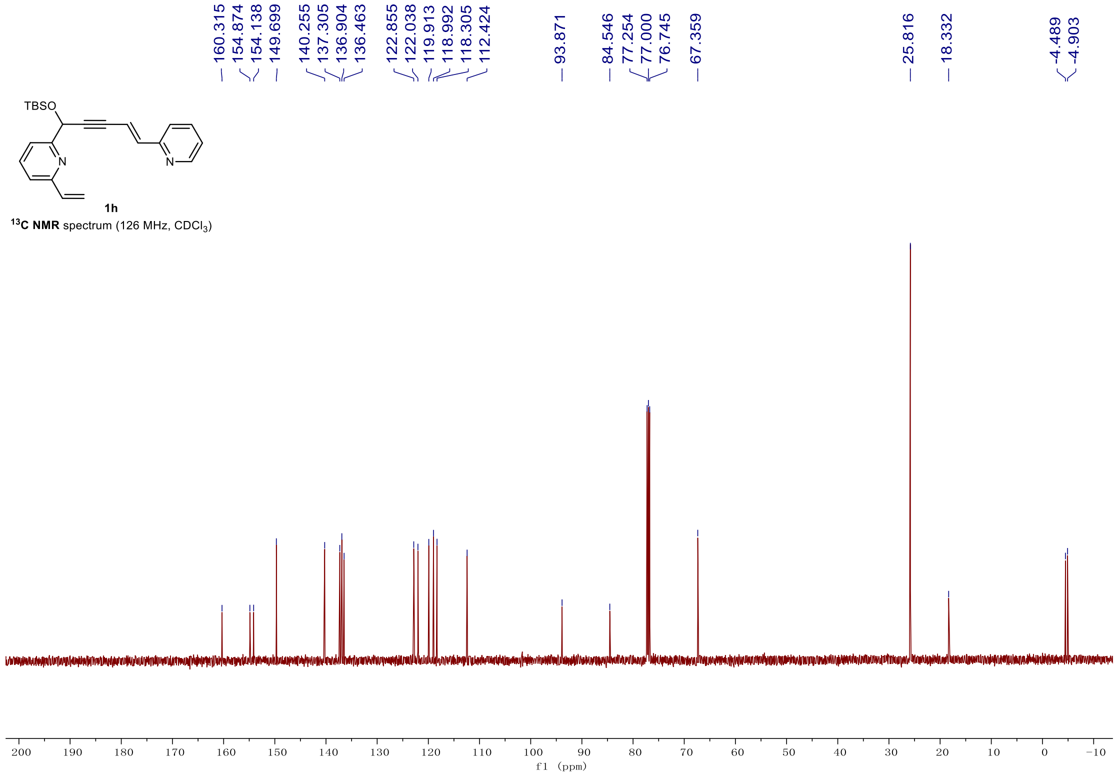

Supplementary Figure 21. ^{13}C NMR (126 MHz, $CDCl_3$) spectra for **1h**

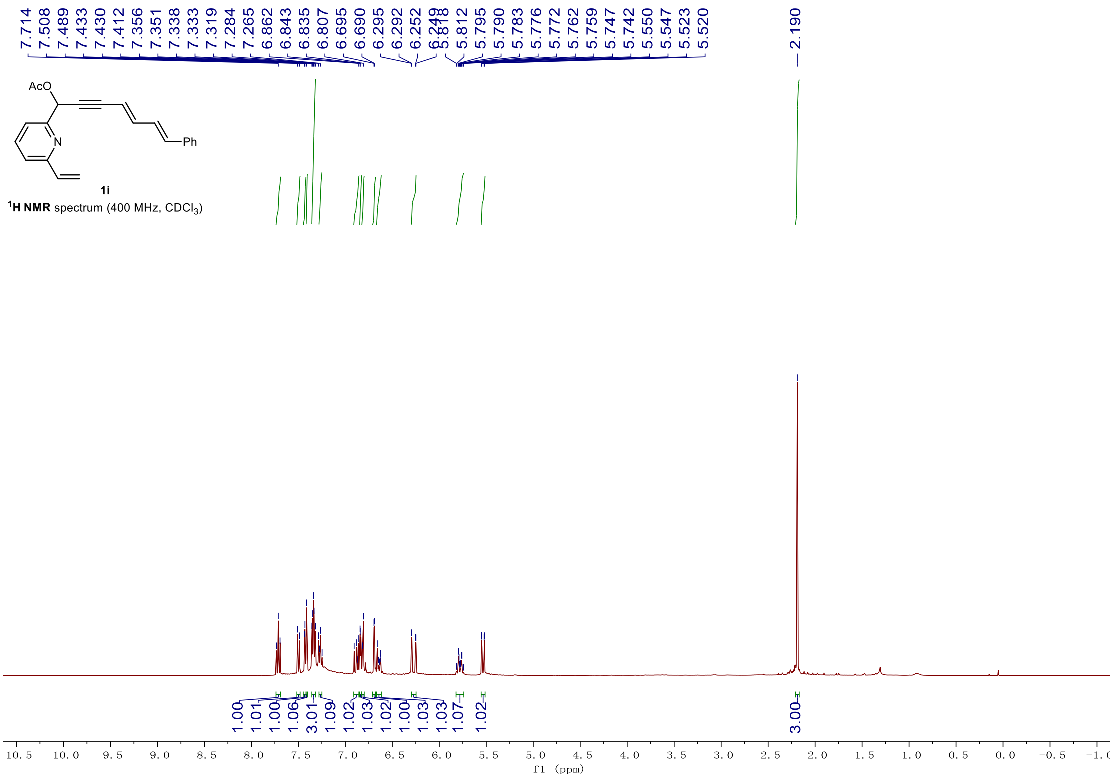

Supplementary Figure 22. ^{1}H NMR (400 MHz, $CDCl_3$) spectra for **1i**

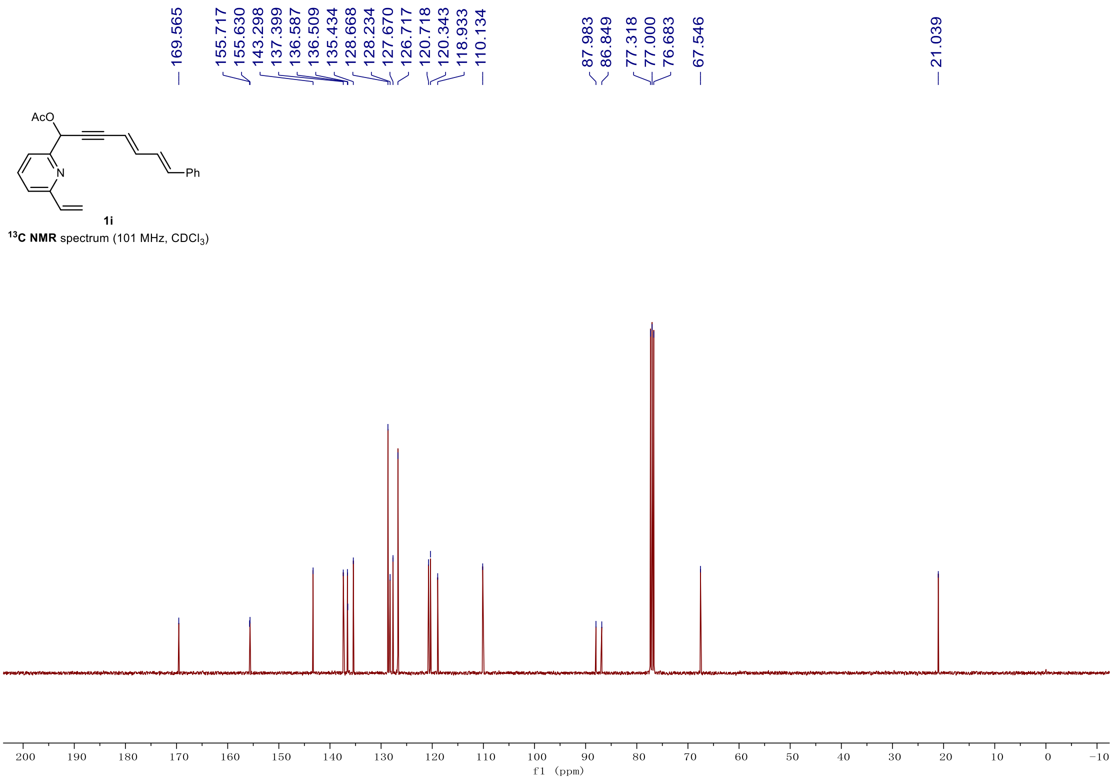

Supplementary Figure 23. ^{13}C NMR (101 MHz, $CDCl_3$) spectra for **1i**

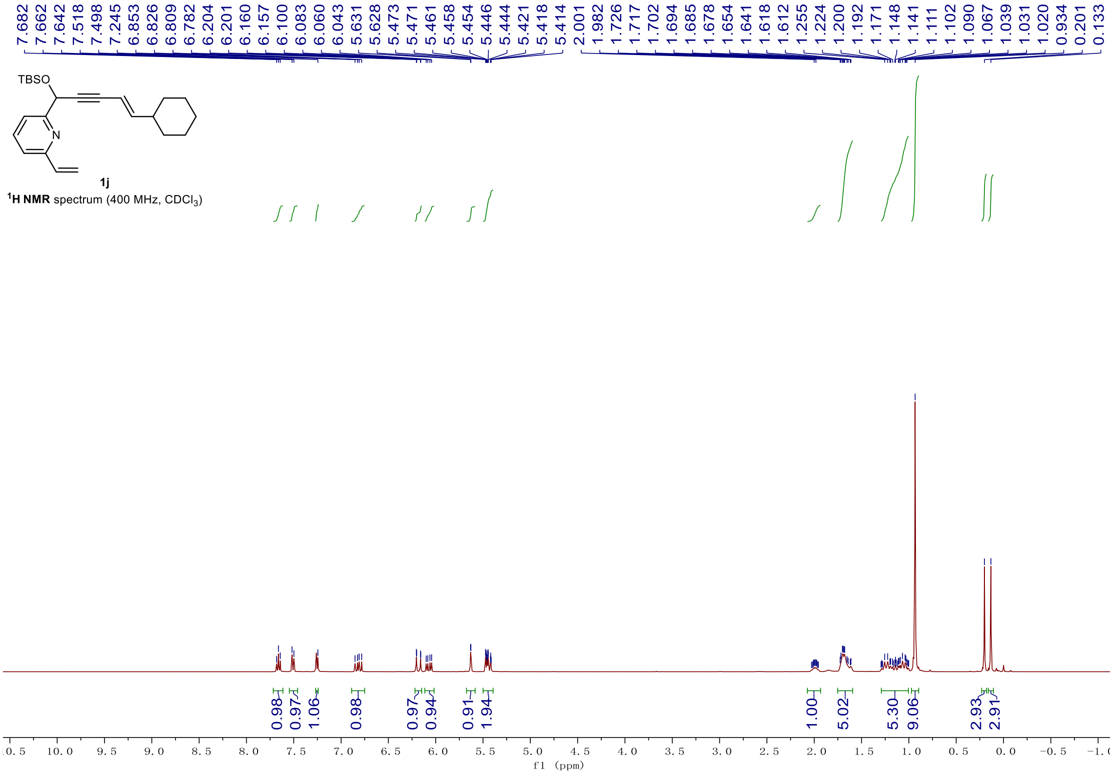

Supplementary Figure 24. ^{1}H NMR (400 MHz, $CDCl_3$) spectra for **1j**

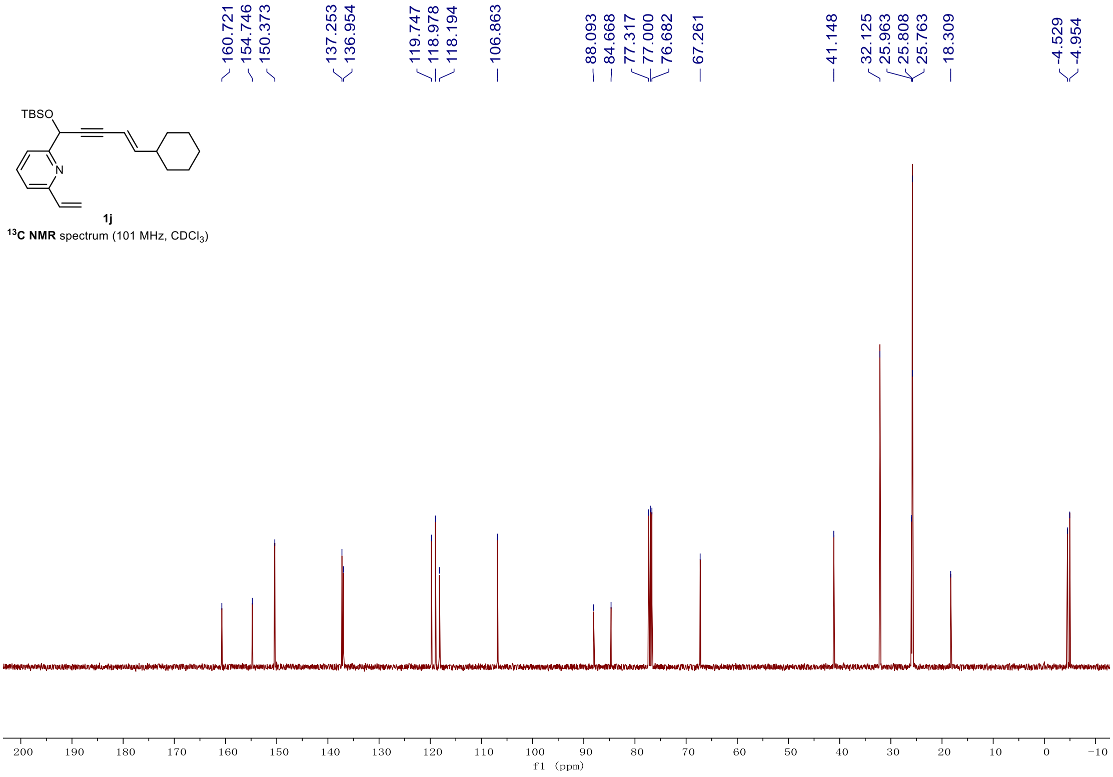

Supplementary Figure 25. ^{13}C NMR (101 MHz, $CDCl_3$) spectra for **1j**

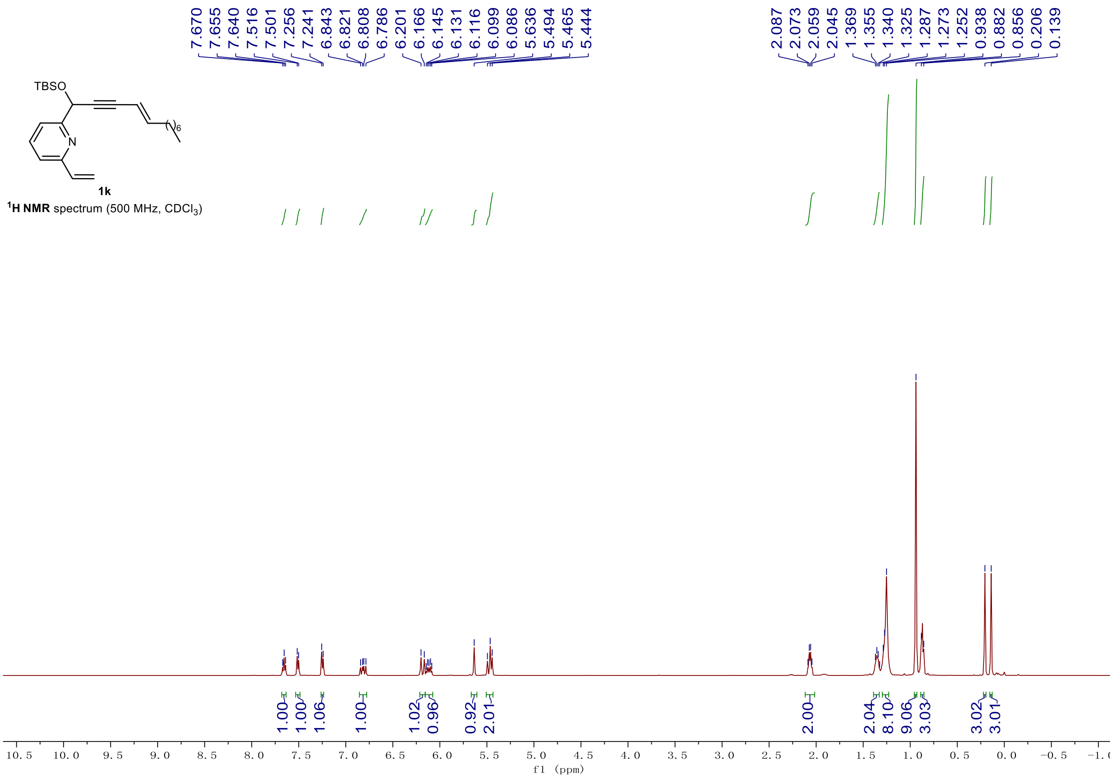

Supplementary Figure 26. ^{1}H NMR (500 MHz, $CDCl_3$) spectra for **1k**

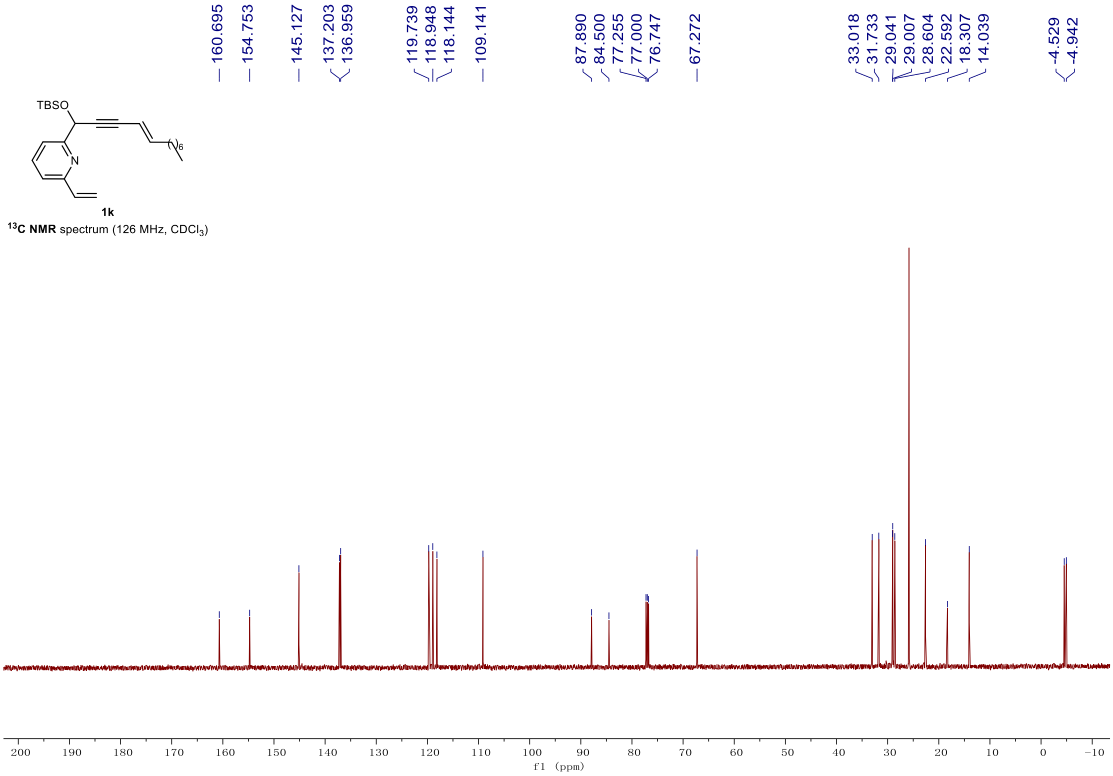

Supplementary Figure 27. ^{13}C NMR (126 MHz, $CDCl_3$) spectra for **1k**

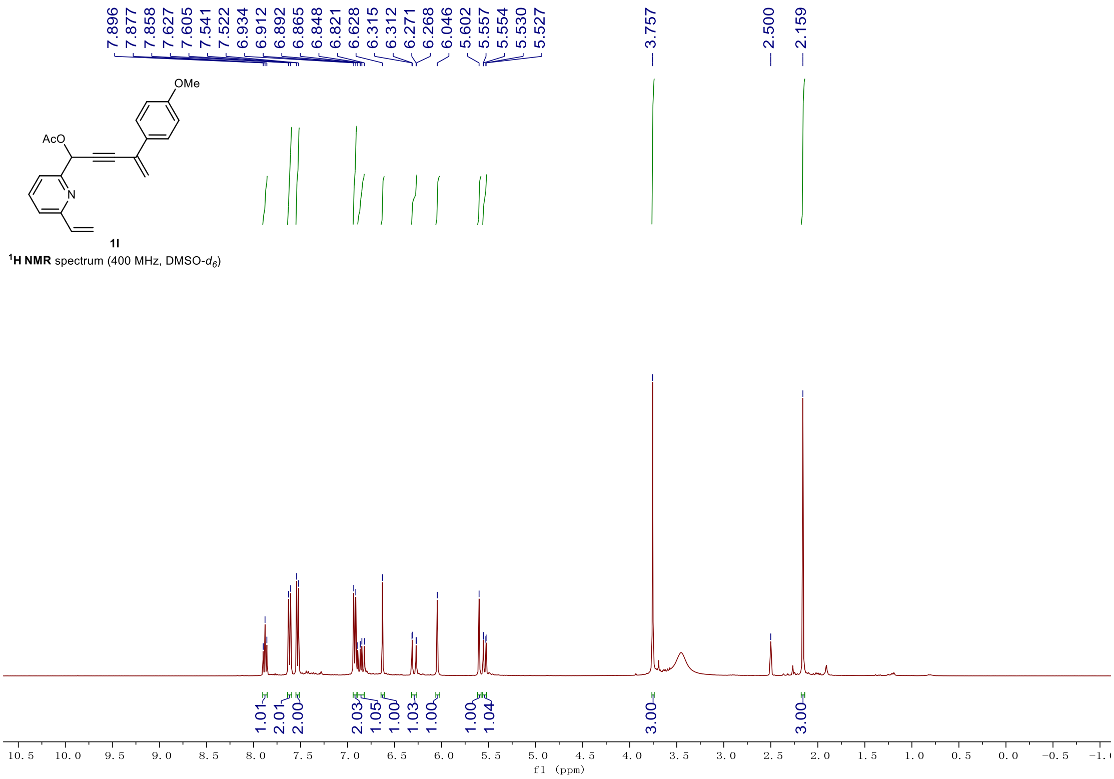

Supplementary Figure 28. ^{1}H NMR (400 MHz, DMSO-*d6*) spectra for **1l**

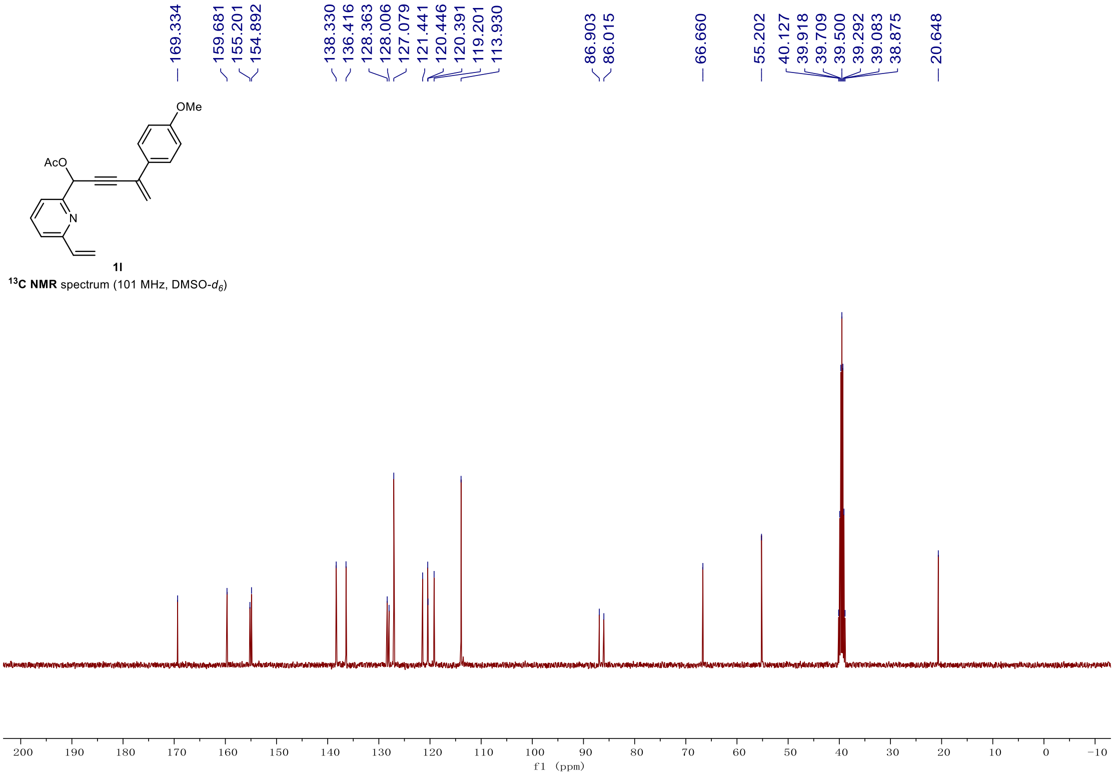

Supplementary Figure 29. ^{13}C NMR (101 MHz, DMSO-*d6*) spectra for **1l**

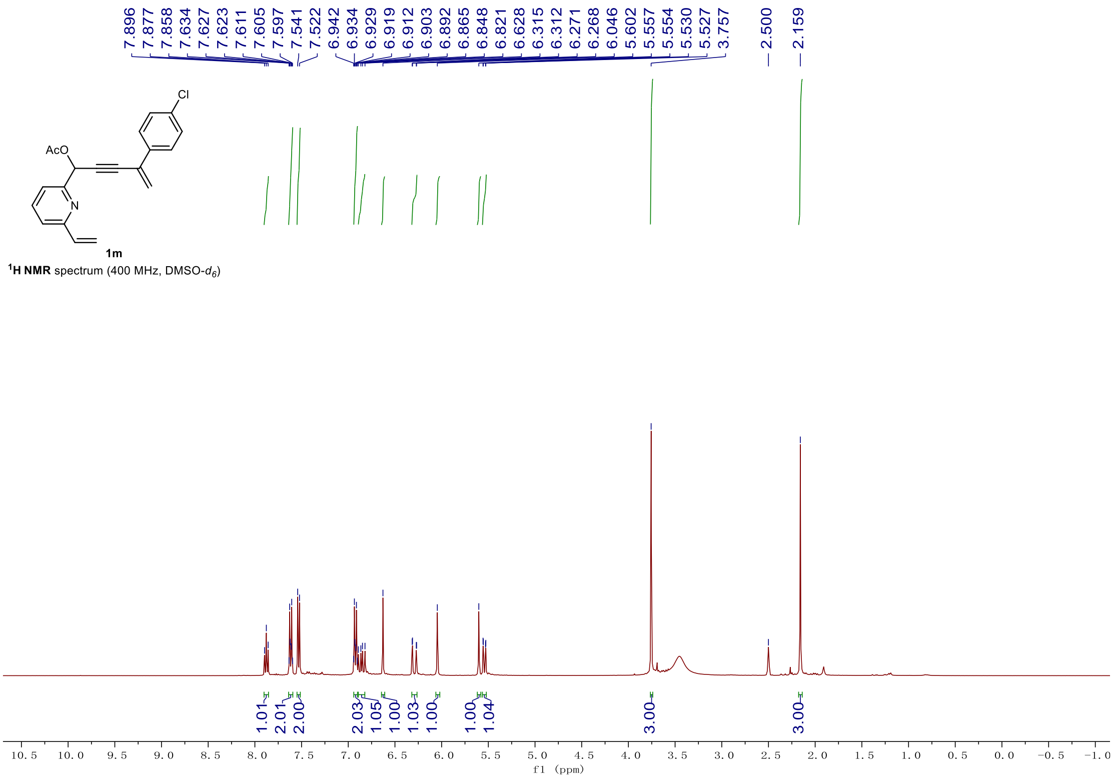

Supplementary Figure 30. ^{1}H NMR (400 MHz, DMSO-*d6*) spectra for **1m**

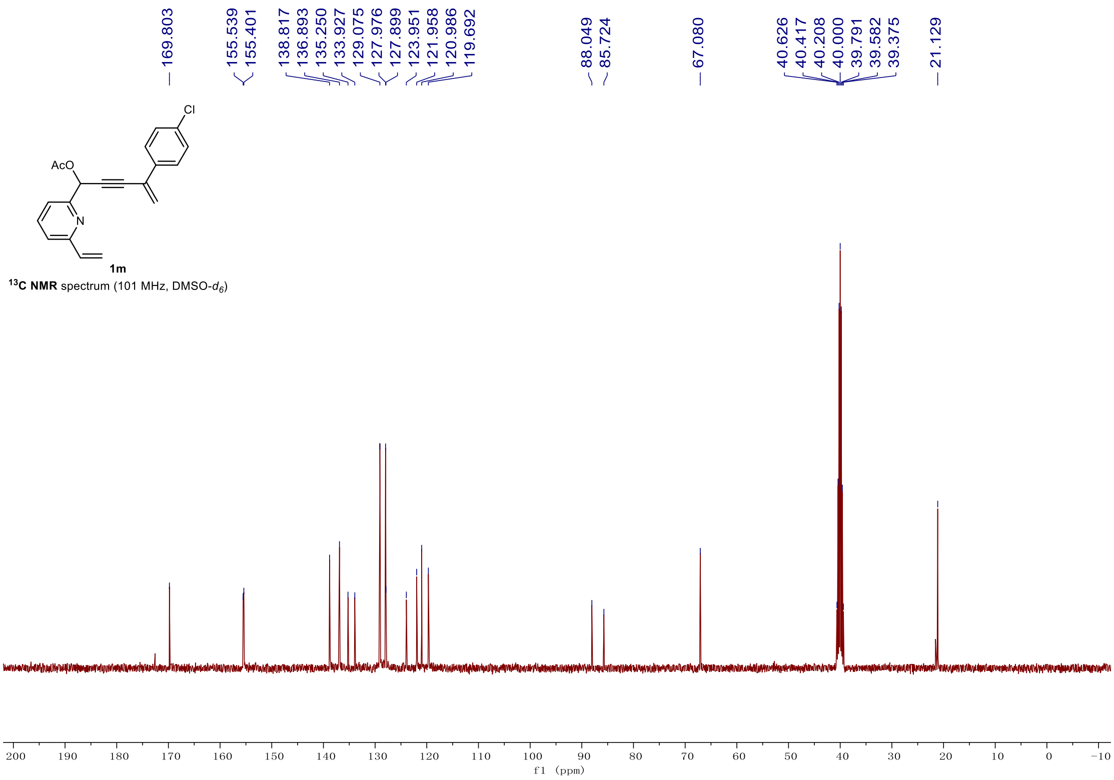

Supplementary Figure 31. ^{13}C NMR (101 MHz, DMSO-*d6*) spectra for **1m**

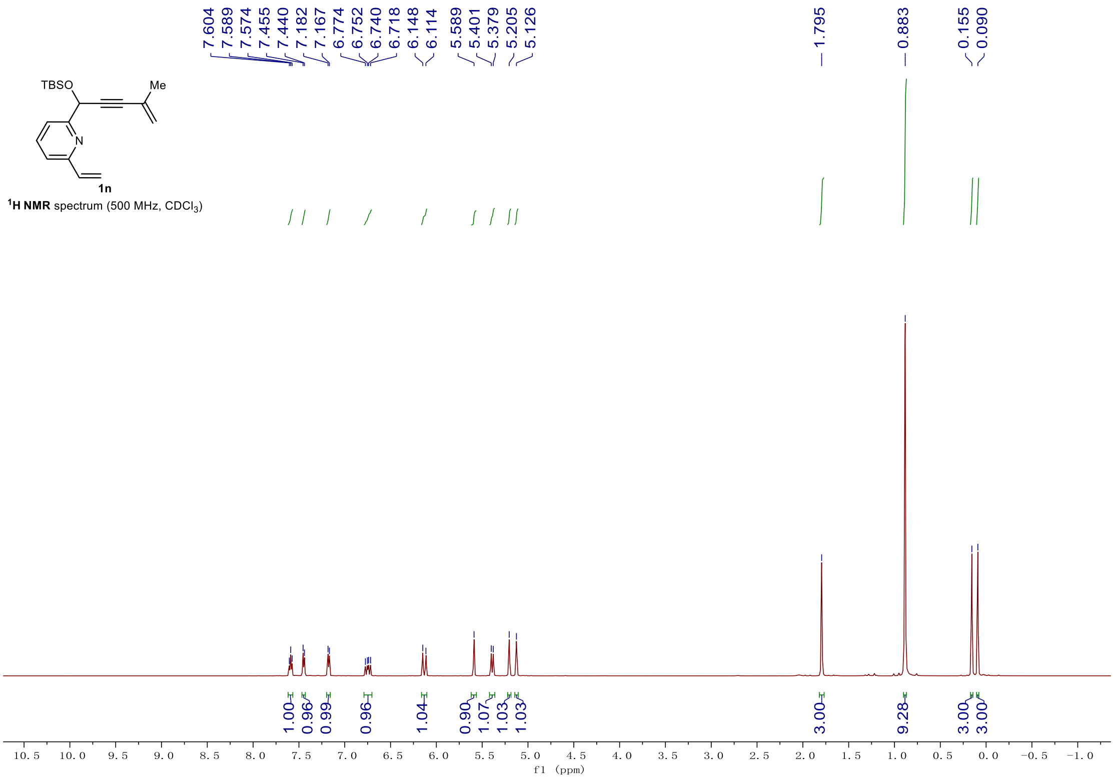

Supplementary Figure 32. ^{1}H NMR (500 MHz, $CDCl_3$) spectra for **1n**

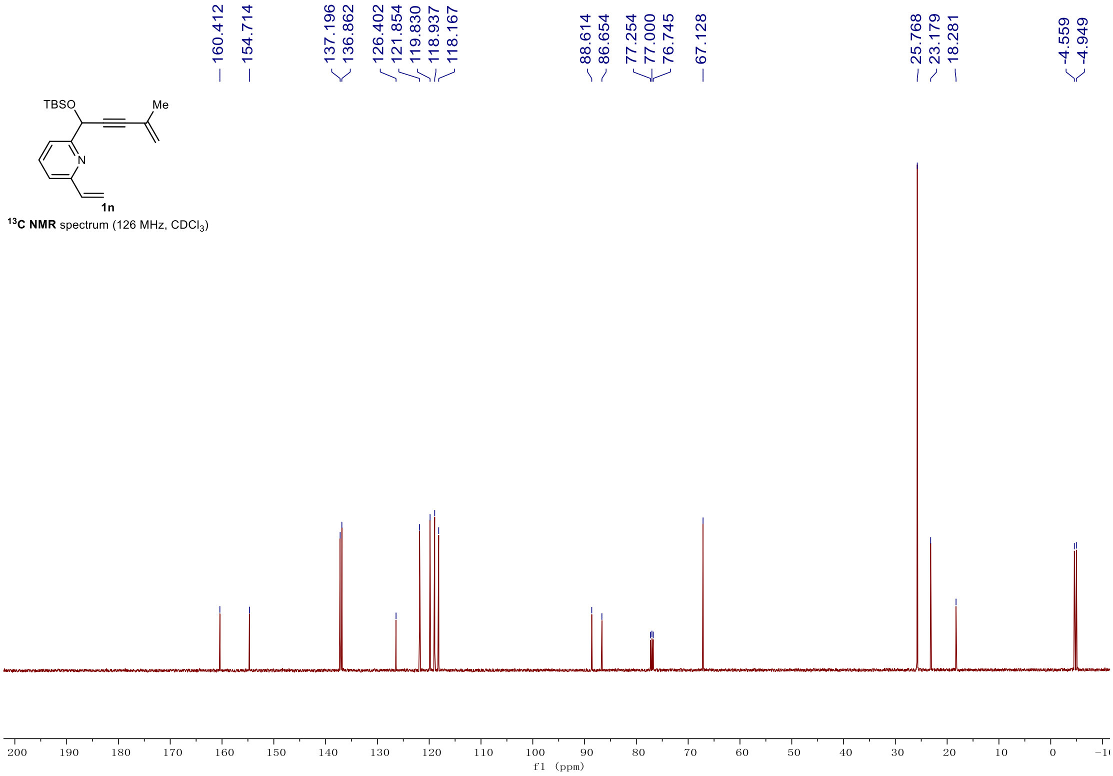

Supplementary Figure 33. ^{13}C NMR (126 MHz, $CDCl_3$) spectra for **1n**

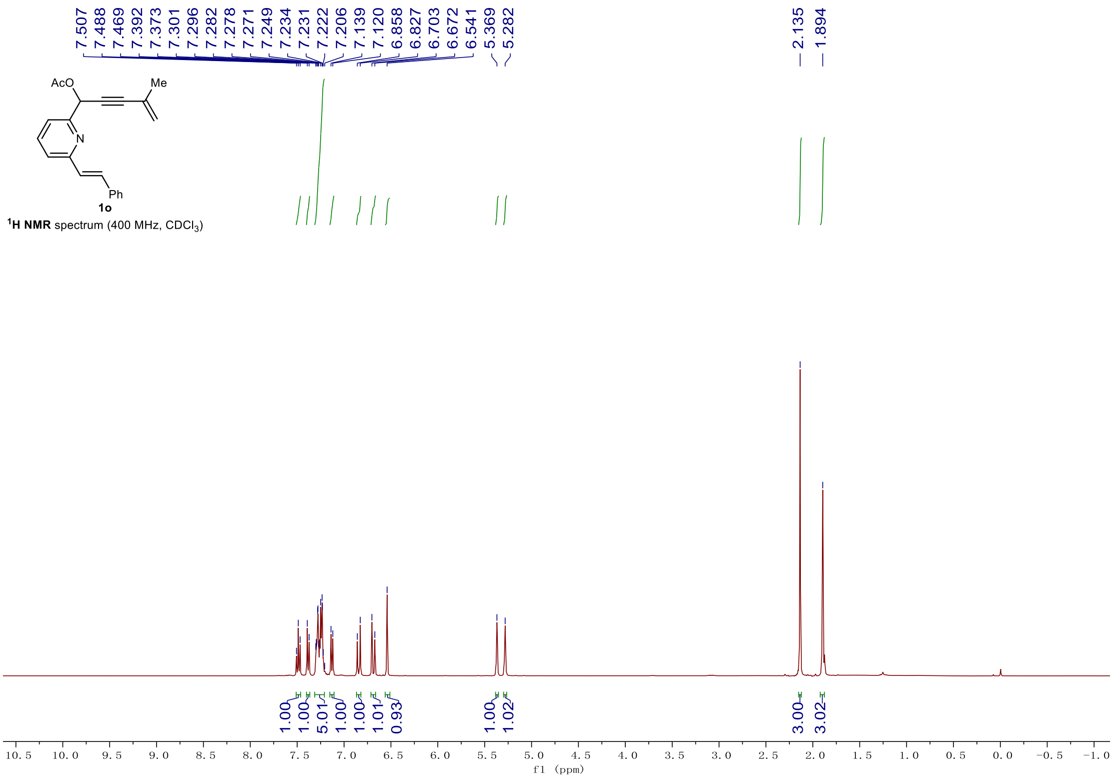

Supplementary Figure 34. ^{1}H NMR (400 MHz, $CDCl_3$) spectra for **1o**

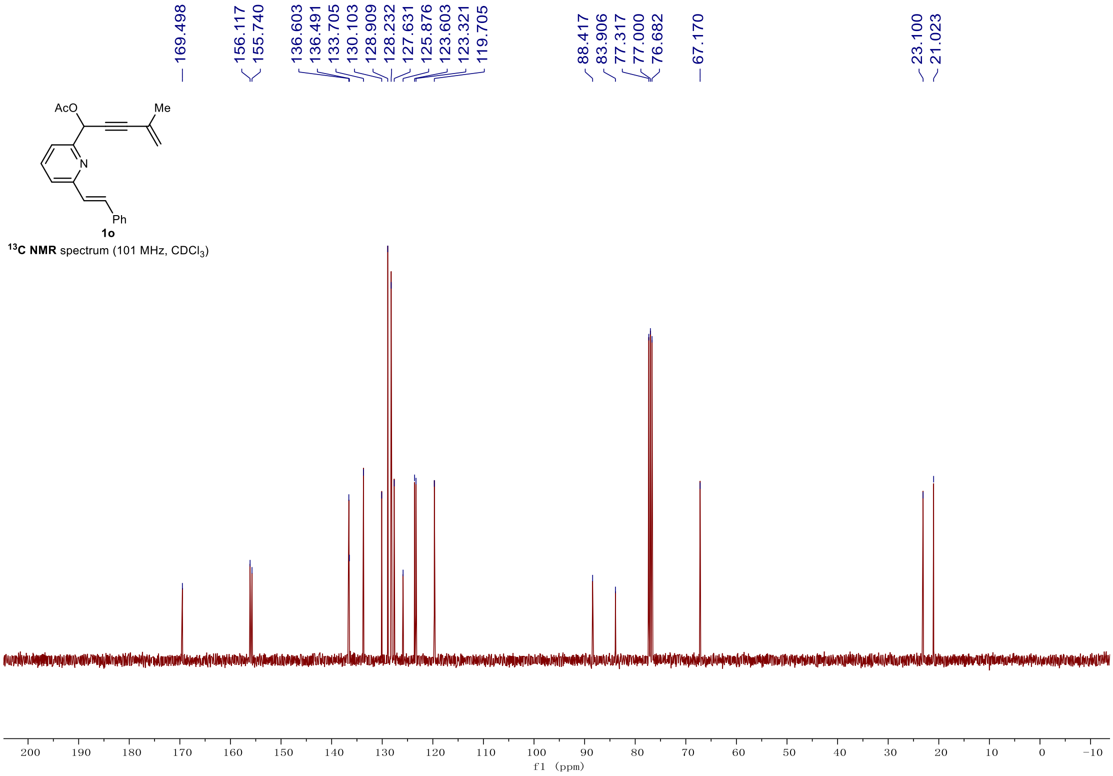

Supplementary Figure 35. ^{13}C NMR (101 MHz, $CDCl_3$) spectra for **1o**

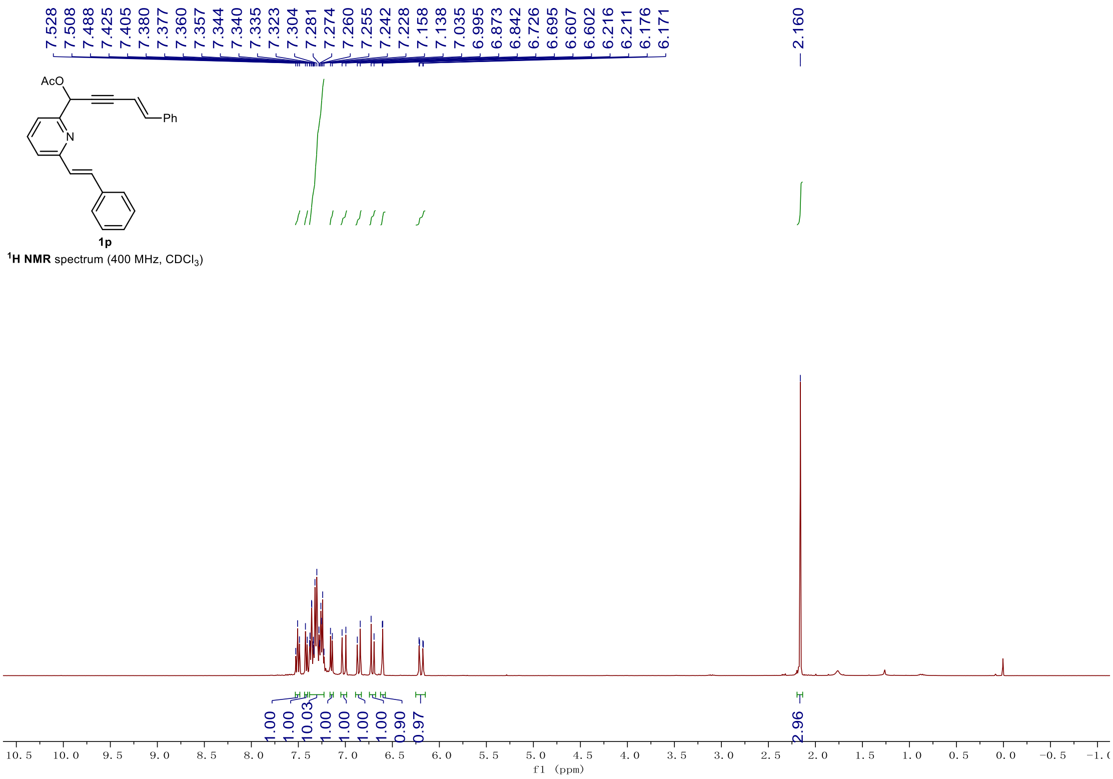

Supplementary Figure 36. ^{1}H NMR (400 MHz, $CDCl_3$) spectra for **1p**

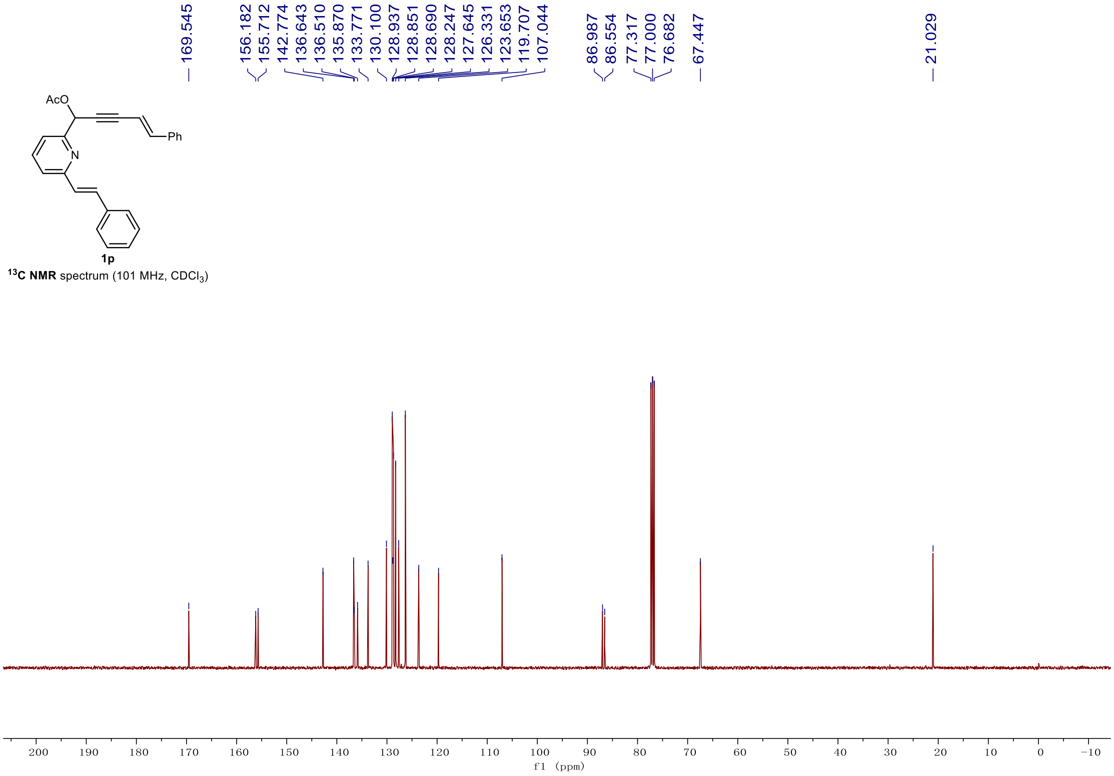

Supplementary Figure 37. ^{13}C NMR (101 MHz, $CDCl_3$) spectra for **1p**

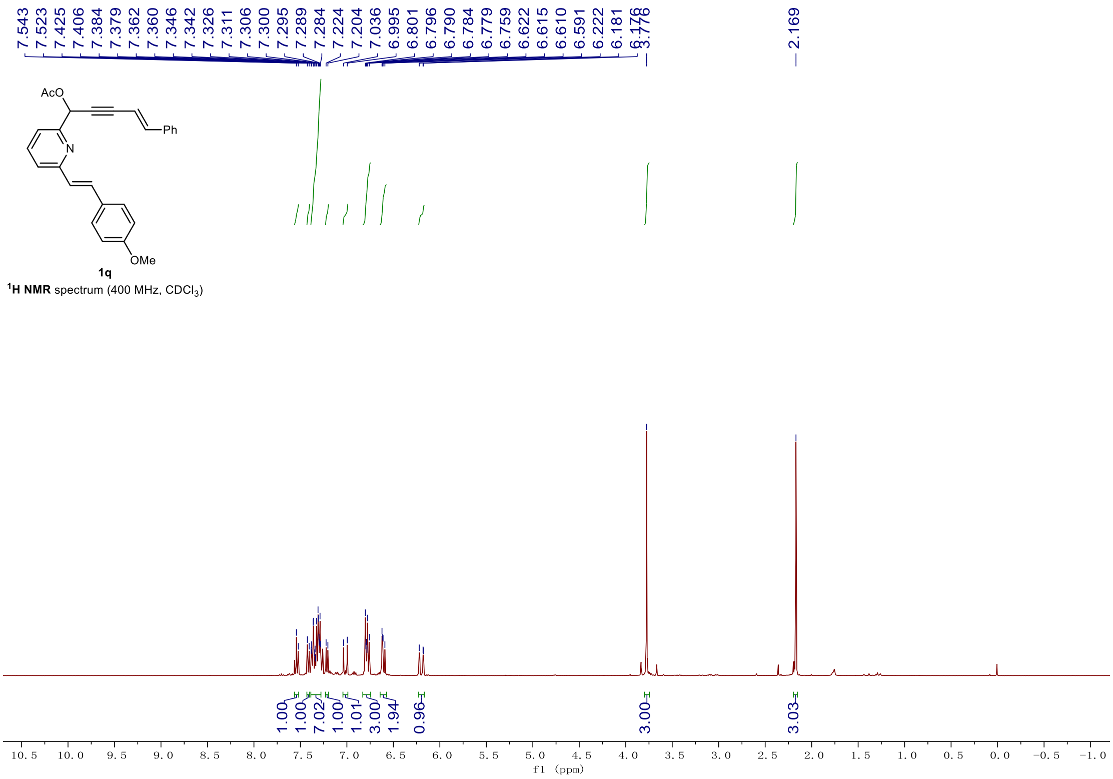

Supplementary Figure 38. ^{1}H NMR (400 MHz, $CDCl_3$) spectra for **1q**

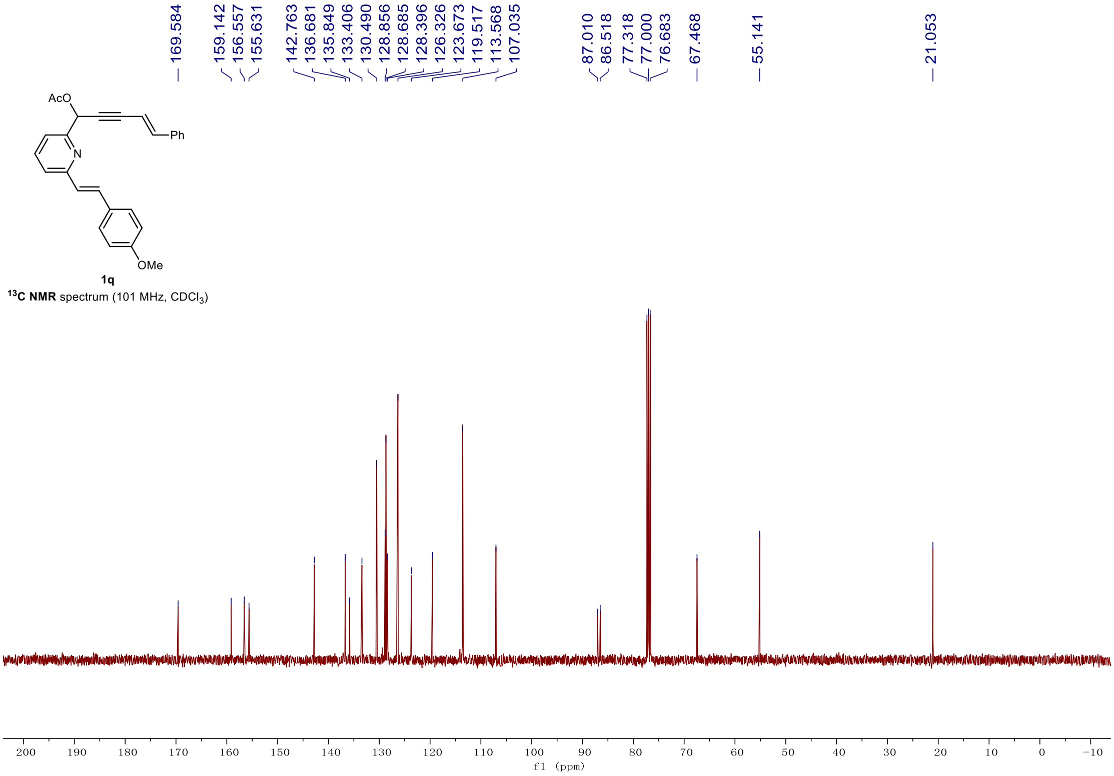

Supplementary Figure 39. ^{13}C NMR (101 MHz, $CDCl_3$) spectra for **1q**

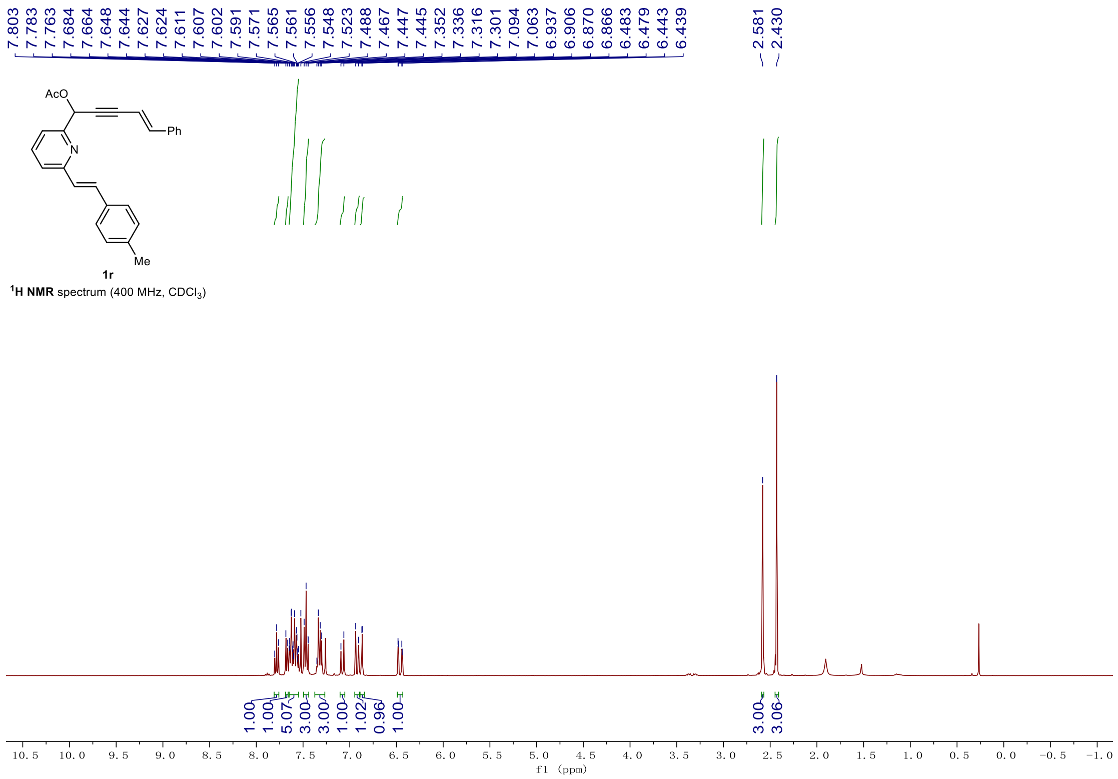

Supplementary Figure 40. ^{1}H NMR (400 MHz, $CDCl_3$) spectra for **1r**

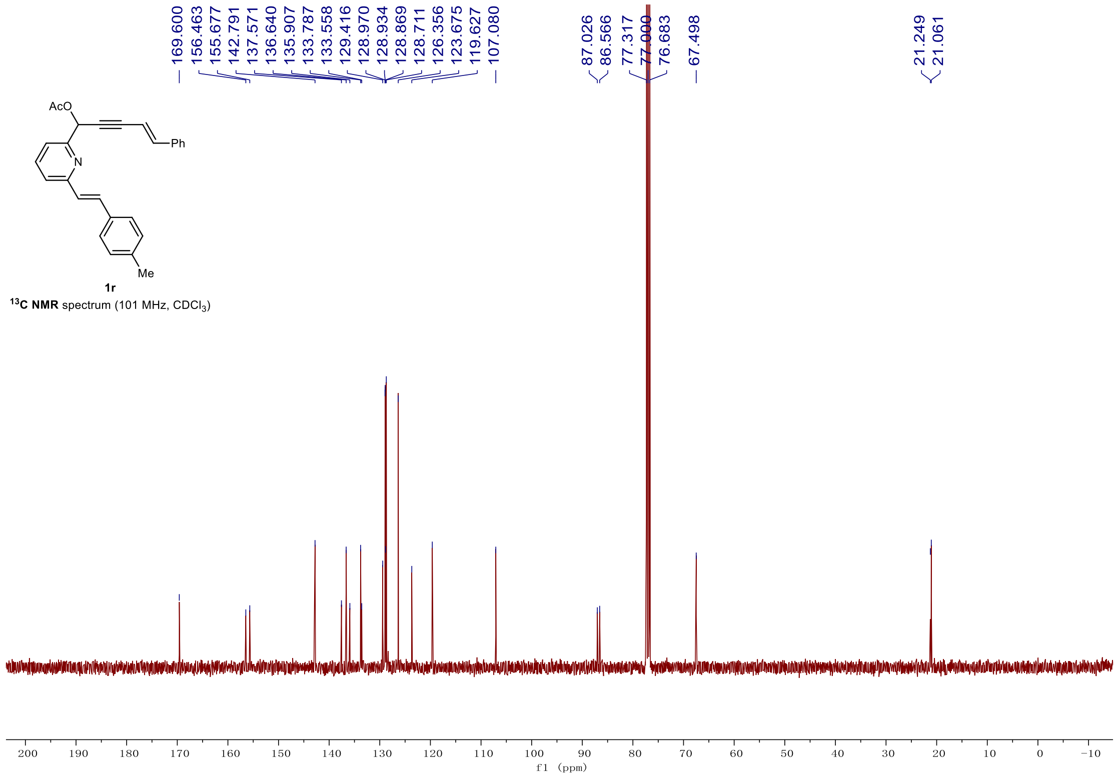

Supplementary Figure 41. ^{13}C NMR (101 MHz, $CDCl_3$) spectra for **1r**

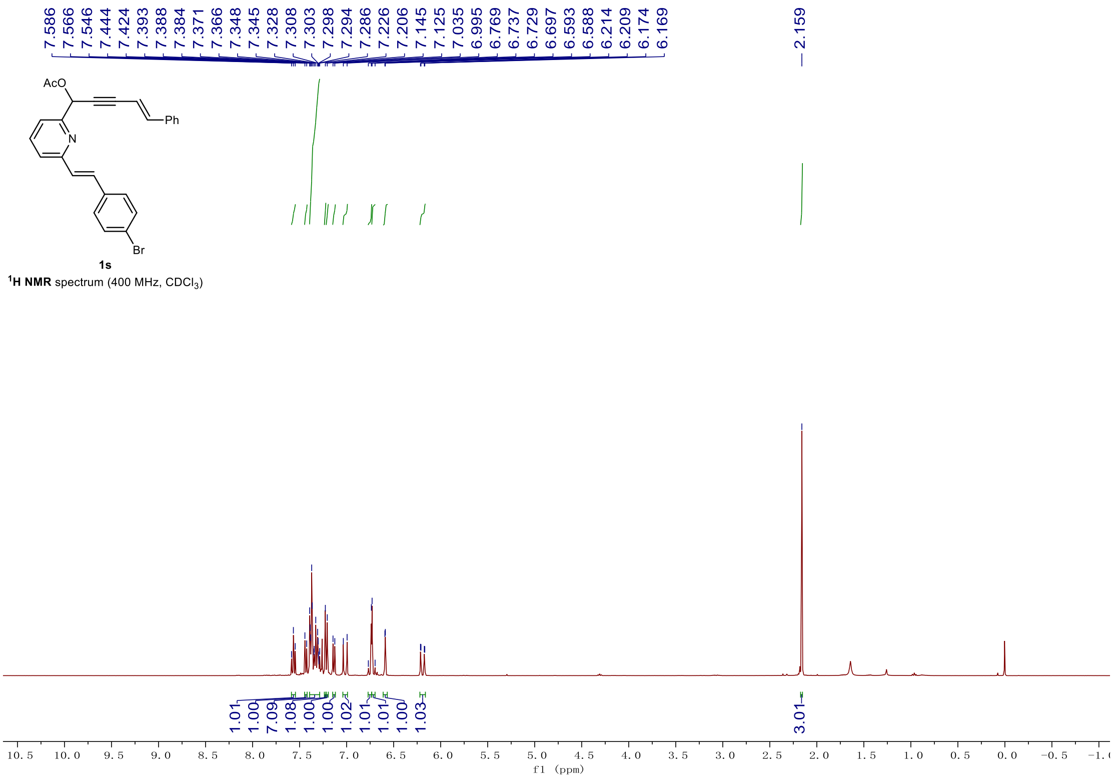

Supplementary Figure 42. ^{1}H NMR (400 MHz, $CDCl_3$) spectra for **1s**

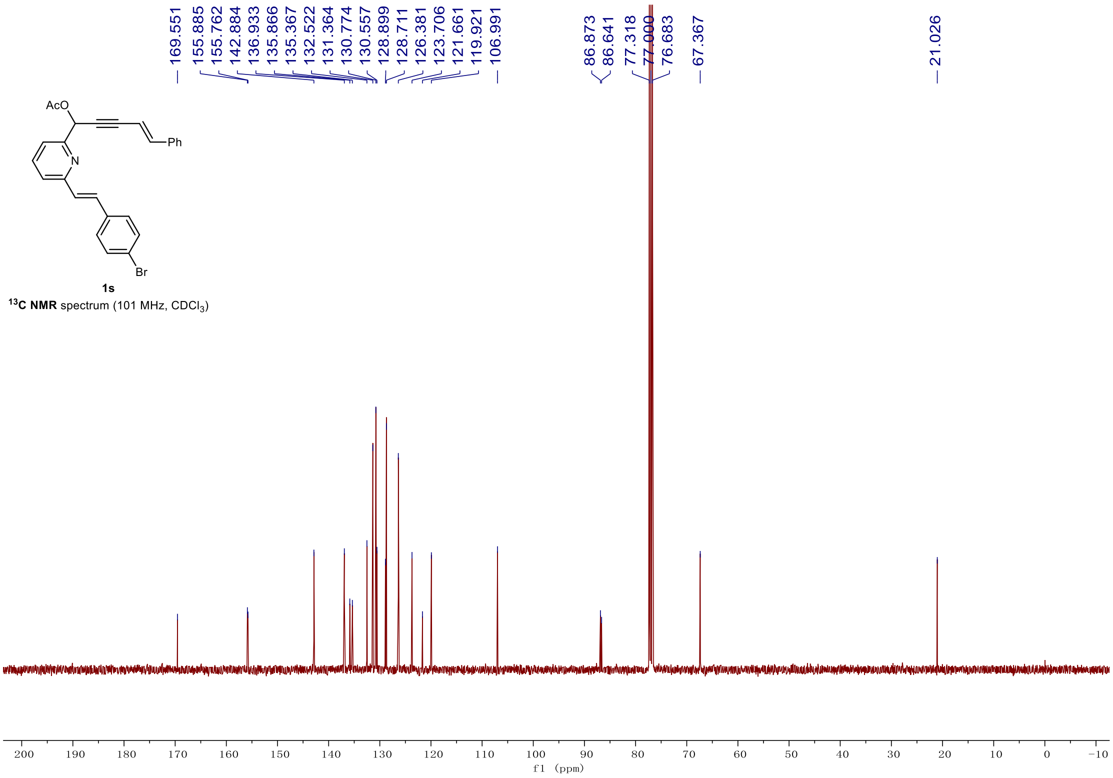

Supplementary Figure 43. ^{13}C NMR (101 MHz, $CDCl_3$) spectra for **1s**

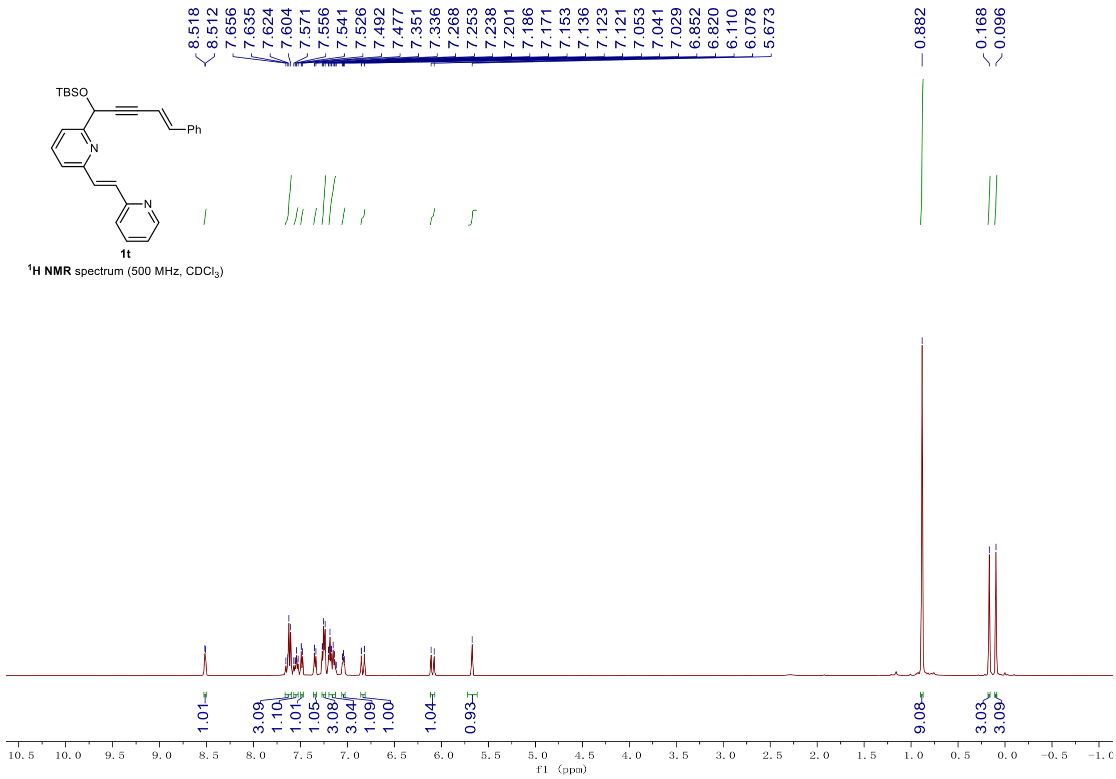

Supplementary Figure 44. ^{1}H NMR (500 MHz, $CDCl_3$) spectra for **1t**

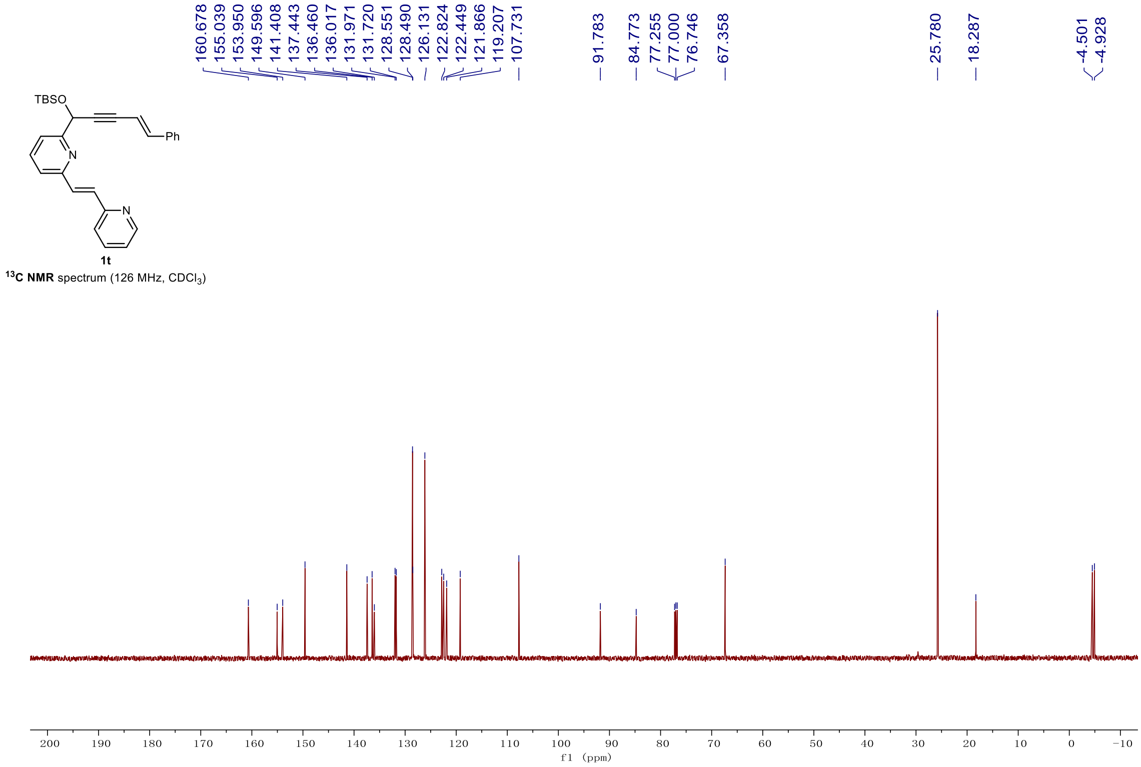

Supplementary Figure 45. ^{13}C NMR (126 MHz, $CDCl_3$) spectra for **1t**

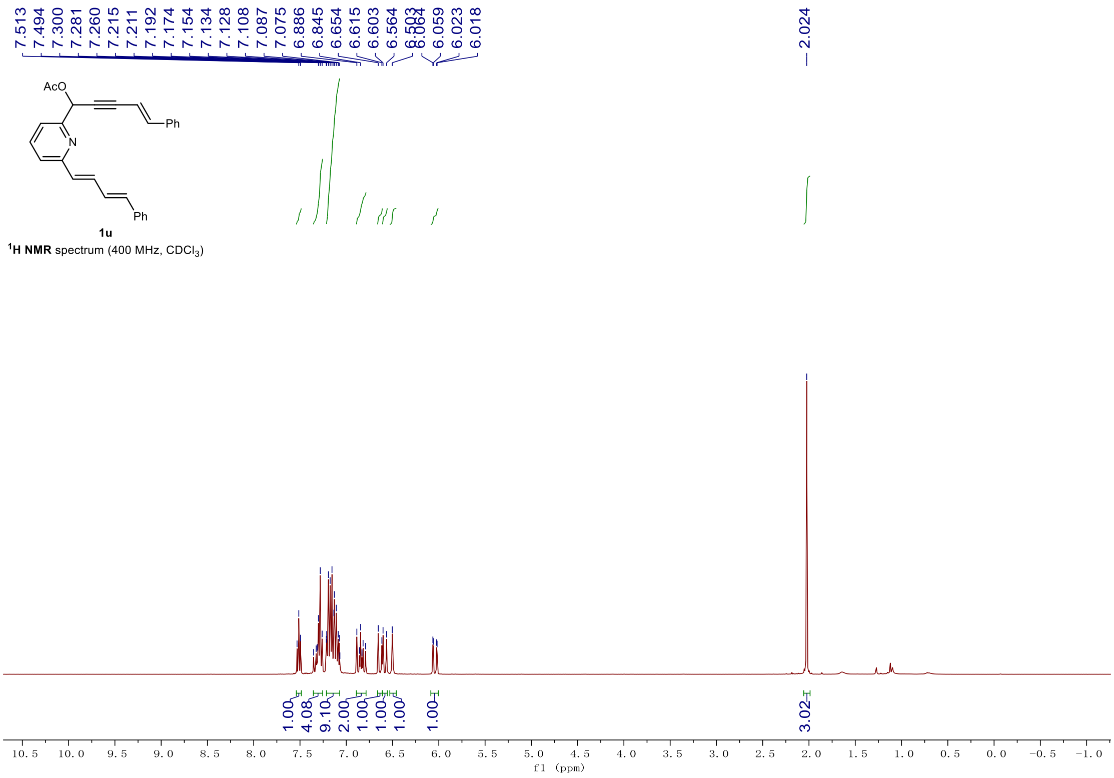

Supplementary Figure 46. ^{1}H NMR (400 MHz, $CDCl_3$) spectra for **1u**

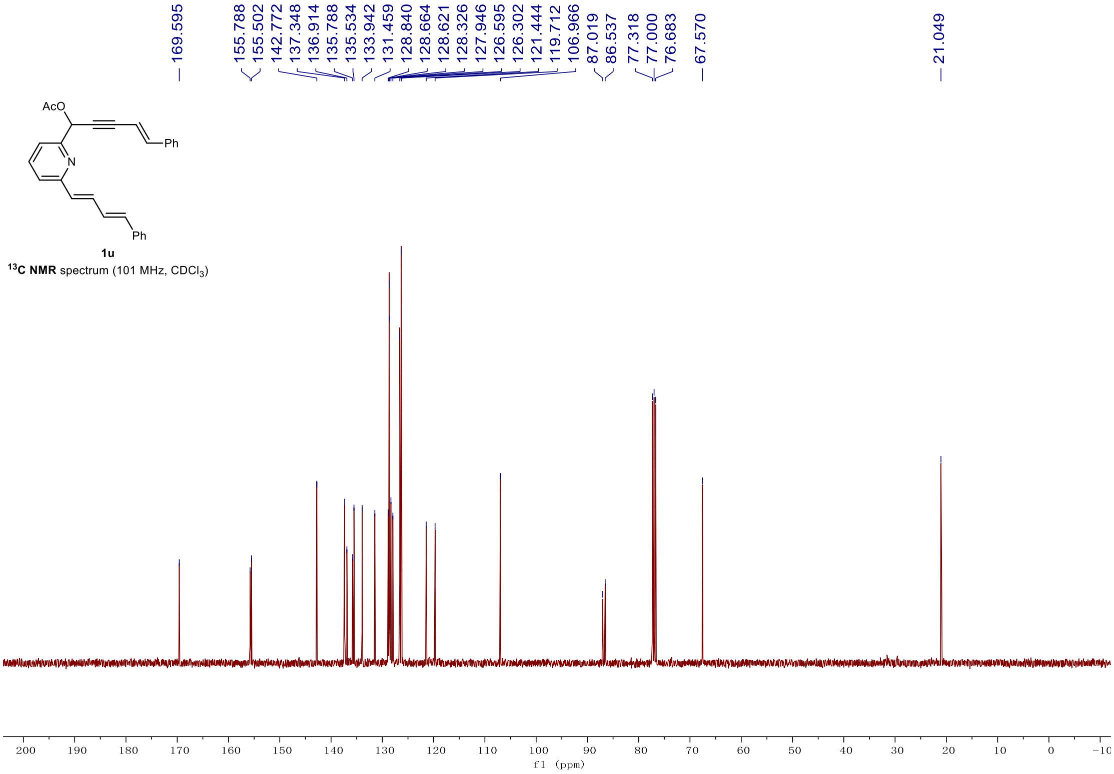

Supplementary Figure 47. ^{13}C NMR (101 MHz, $CDCl_3$) spectra for **1u**

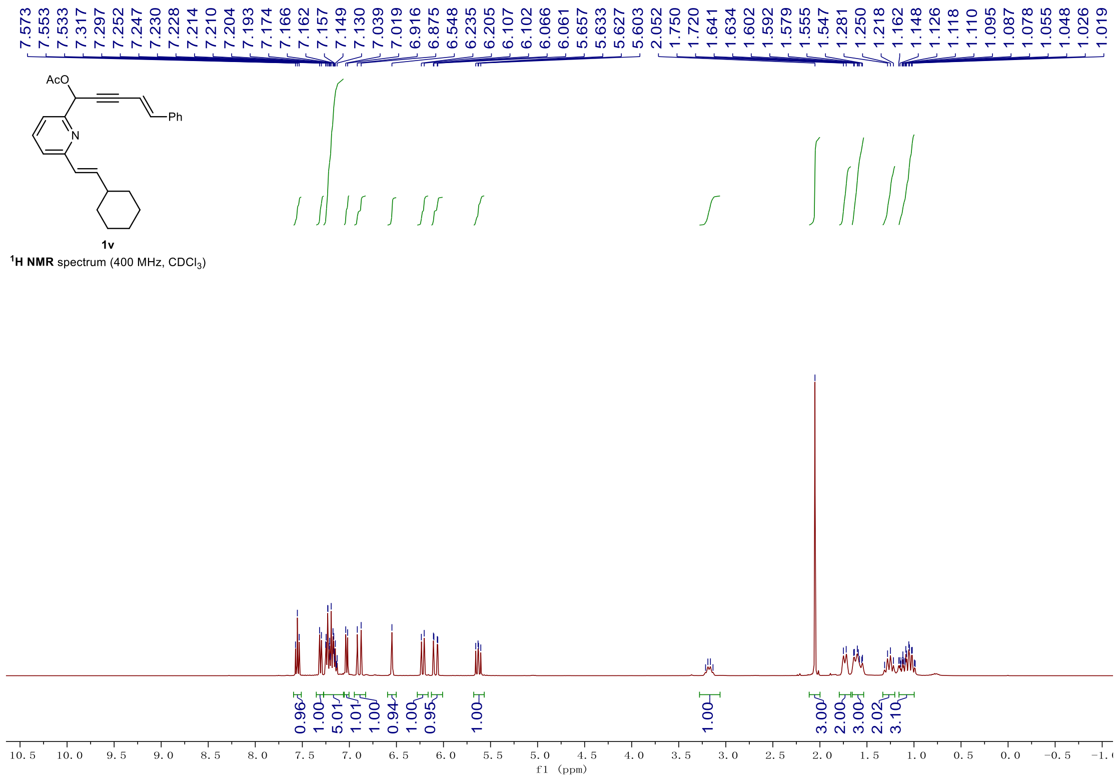

Supplementary Figure 48. ^{1}H NMR (400 MHz, $CDCl_3$) spectra for **1v**

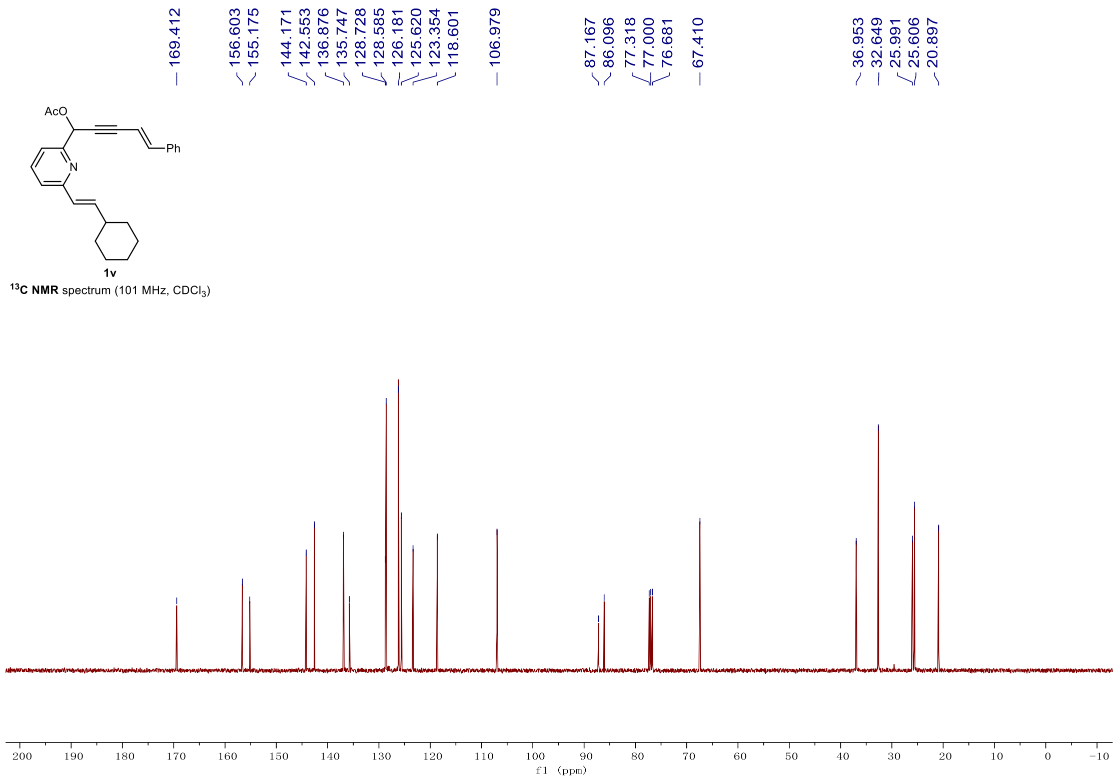

Supplementary Figure 49. ^{13}C NMR (101 MHz, $CDCl_3$) spectra for **1v**

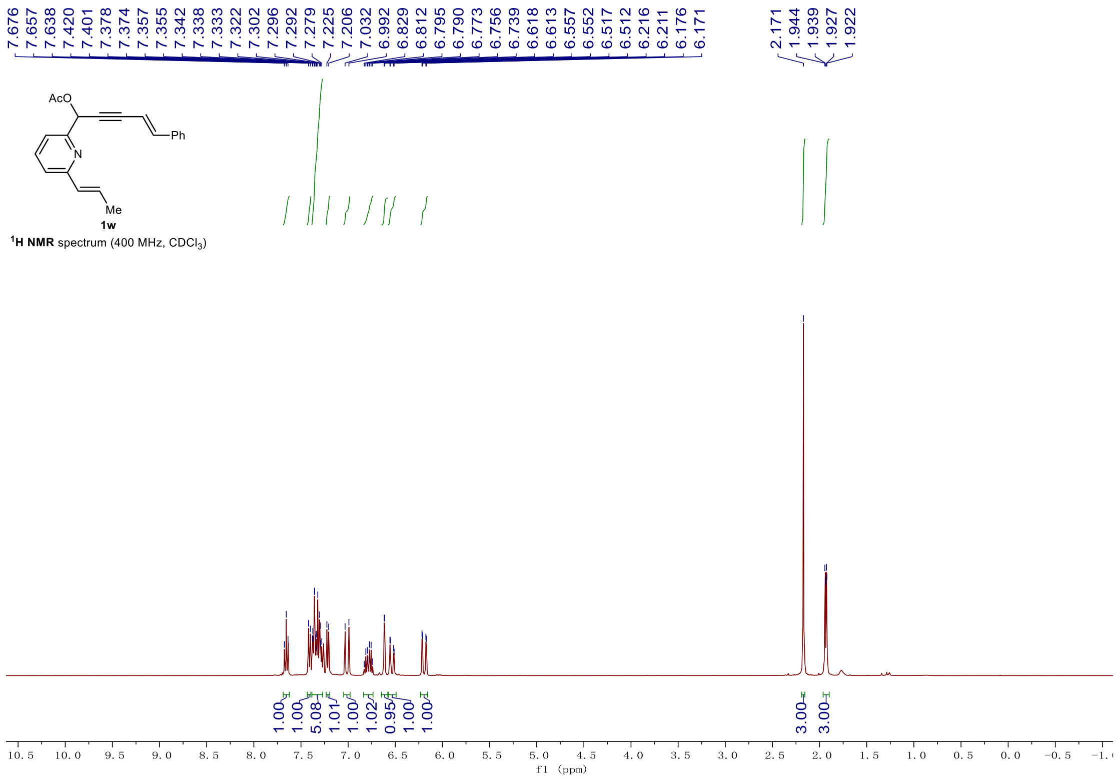

Supplementary Figure 50. ^{1}H NMR (400 MHz, $CDCl_3$) spectra for **1w**

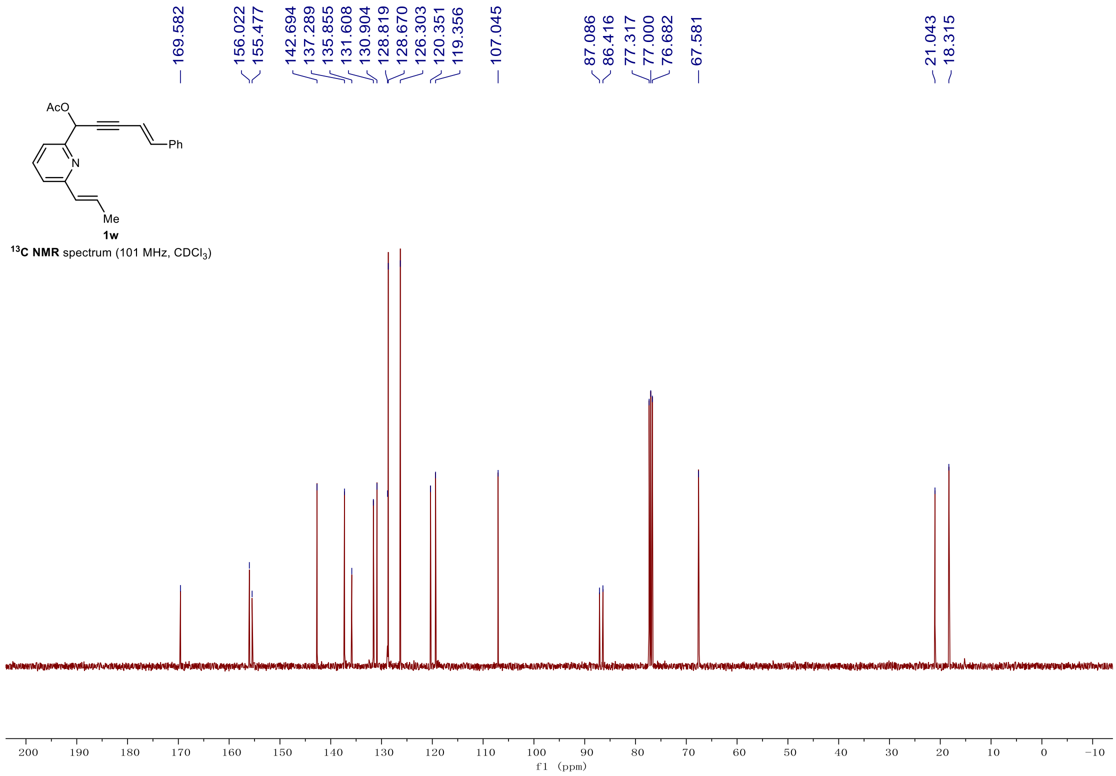

Supplementary Figure 51. ^{13}C NMR (101 MHz, $CDCl_3$) spectra for **1w**

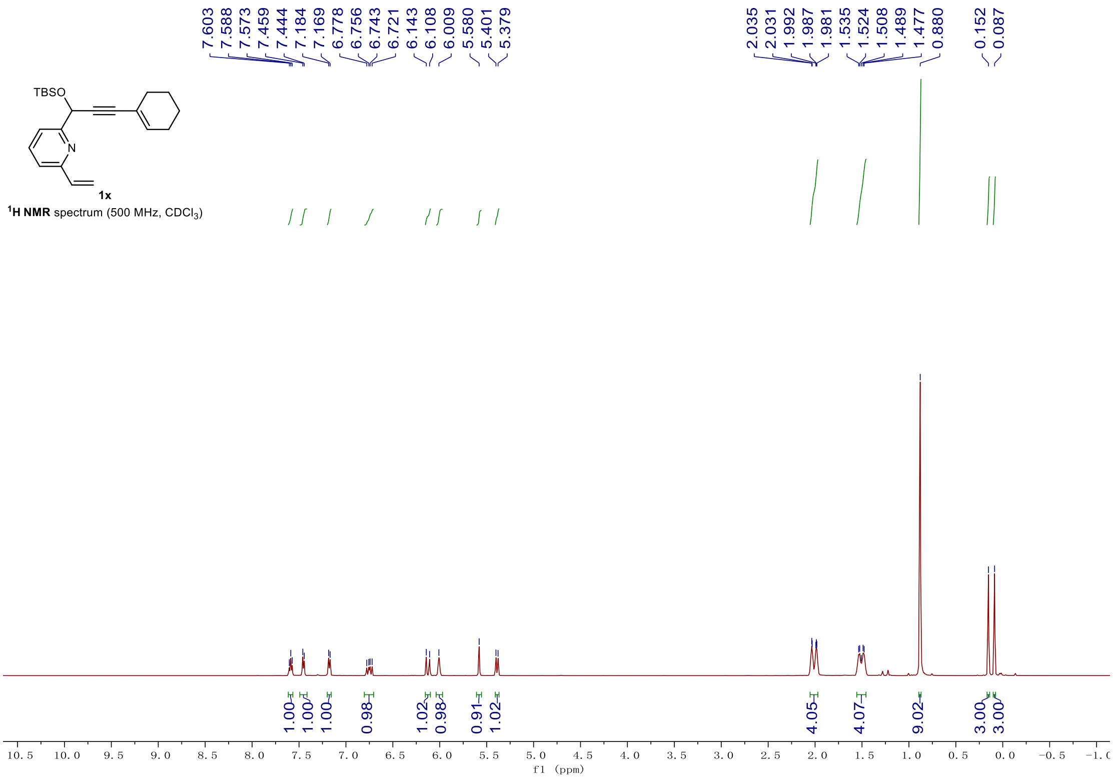

Supplementary Figure 52. ^{1}H NMR (500 MHz, $CDCl_3$) spectra for **1x**

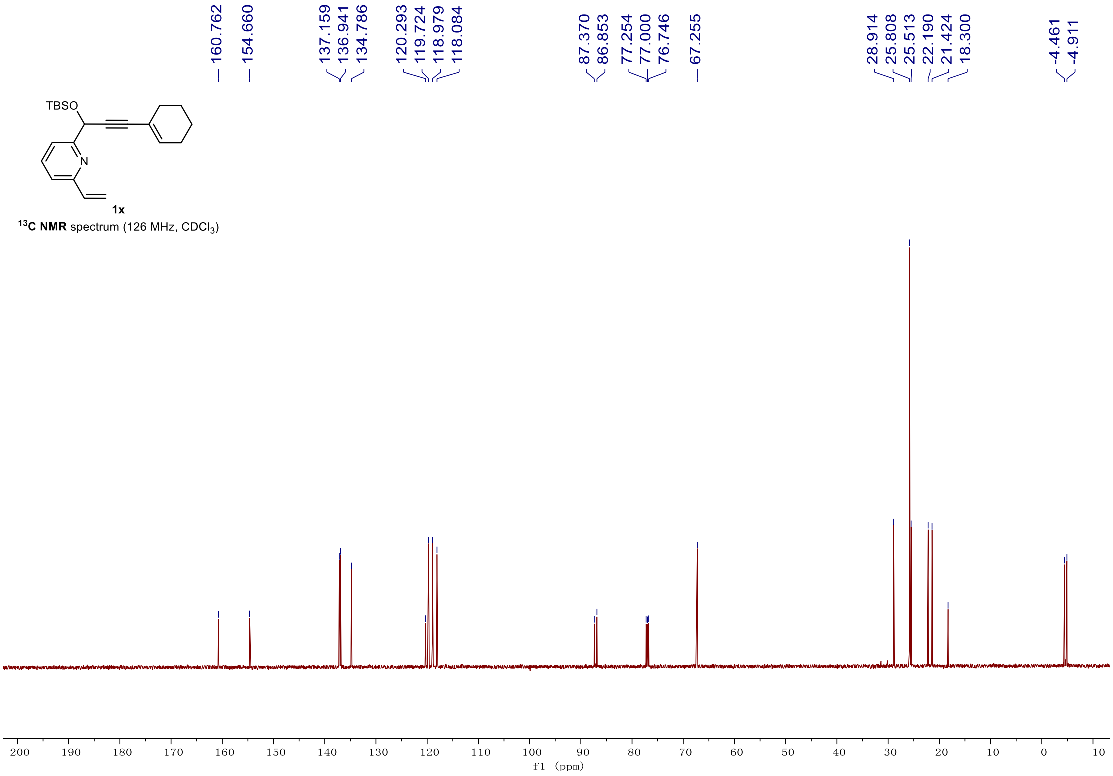

Supplementary Figure 53. ^{13}C NMR (126 MHz, $CDCl_3$) spectra for **1x**

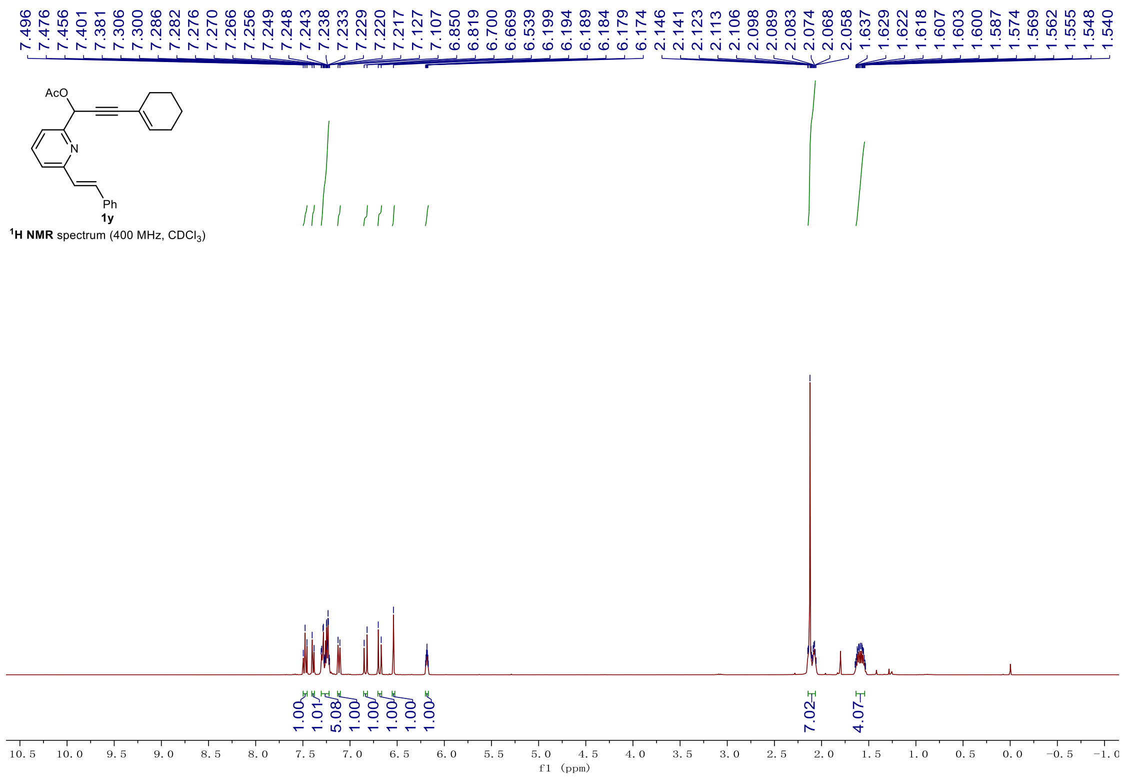

Supplementary Figure 54. ^{1}H NMR (400 MHz, $CDCl_3$) spectra for **1y**

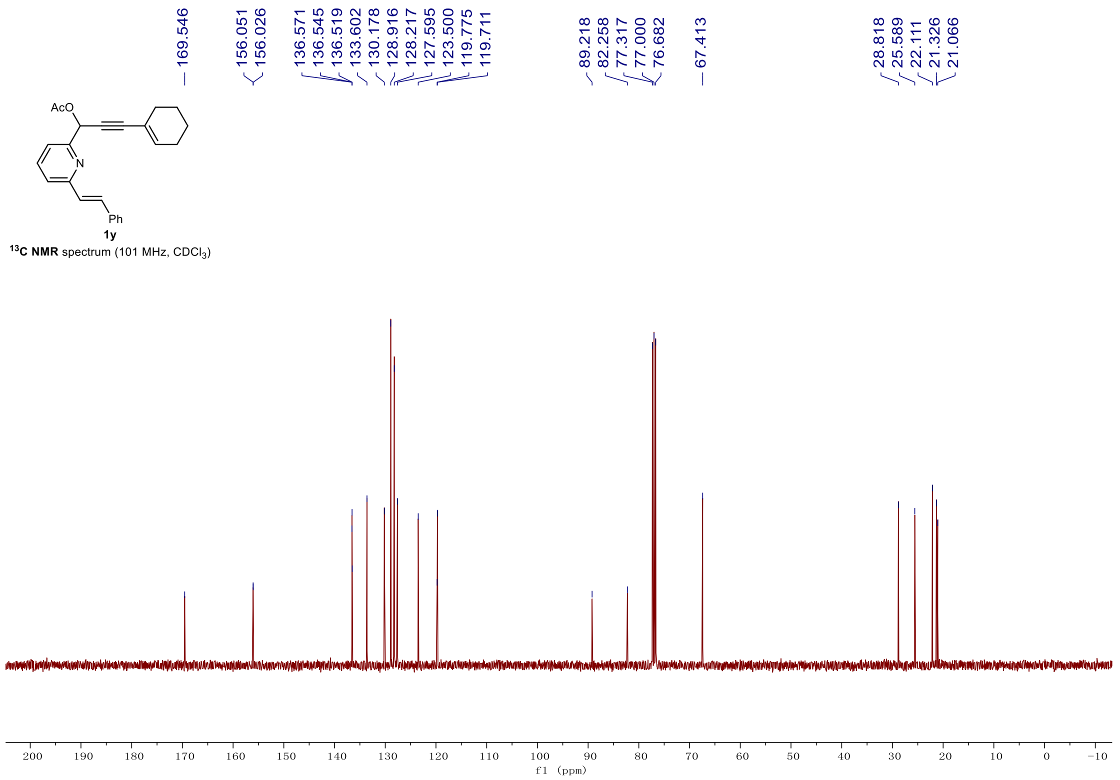

Supplementary Figure 55. ^{13}C NMR (101 MHz, $CDCl_3$) spectra for **1y**

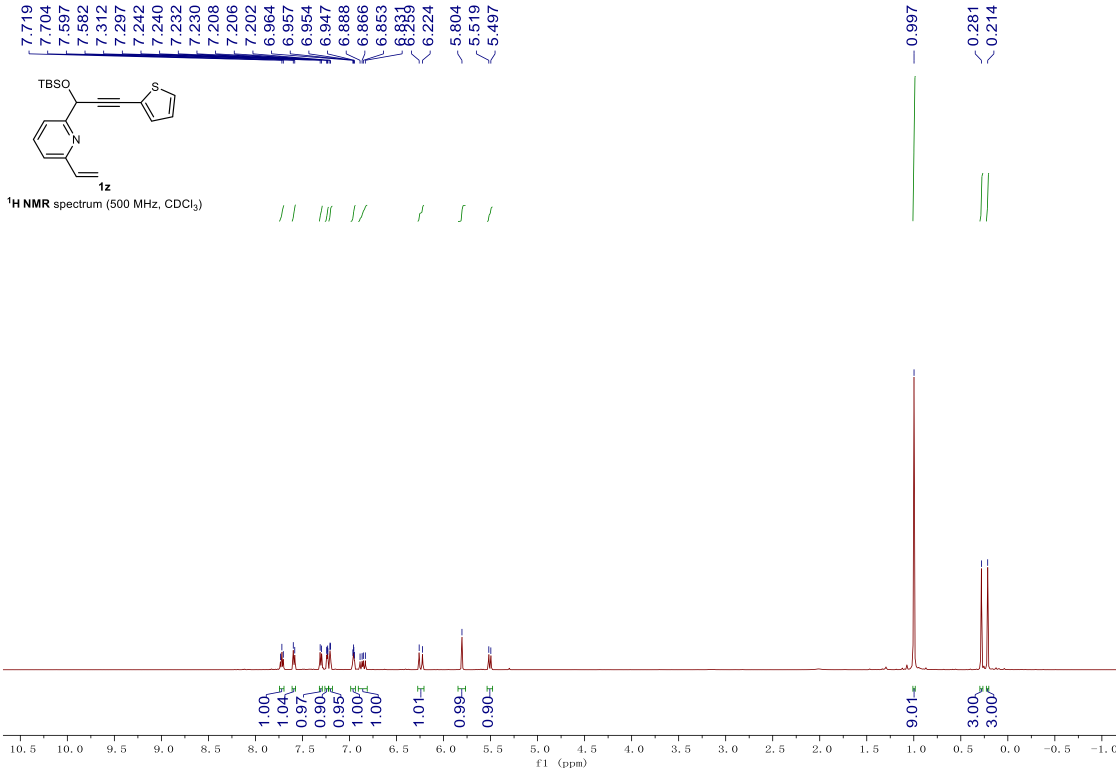

Supplementary Figure 56. ^{1}H NMR (500 MHz, $CDCl_3$) spectra for **1z**

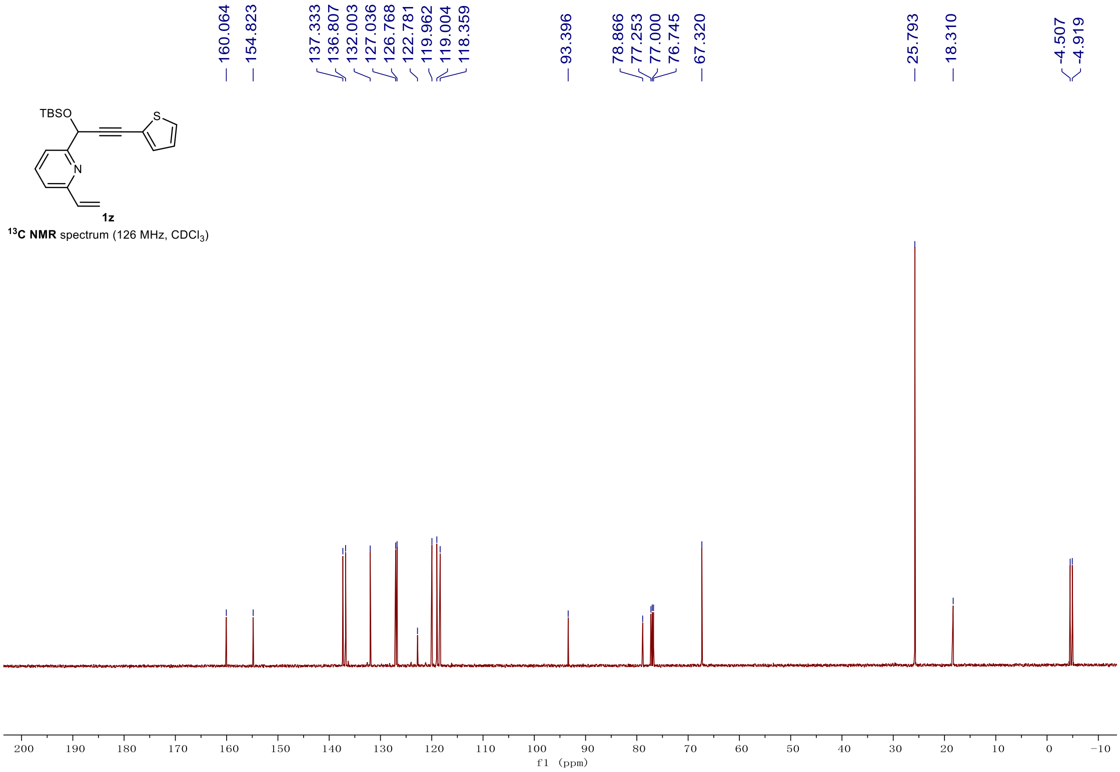

Supplementary Figure 57. ^{13}C NMR (126 MHz, $CDCl_3$) spectra for **1z**

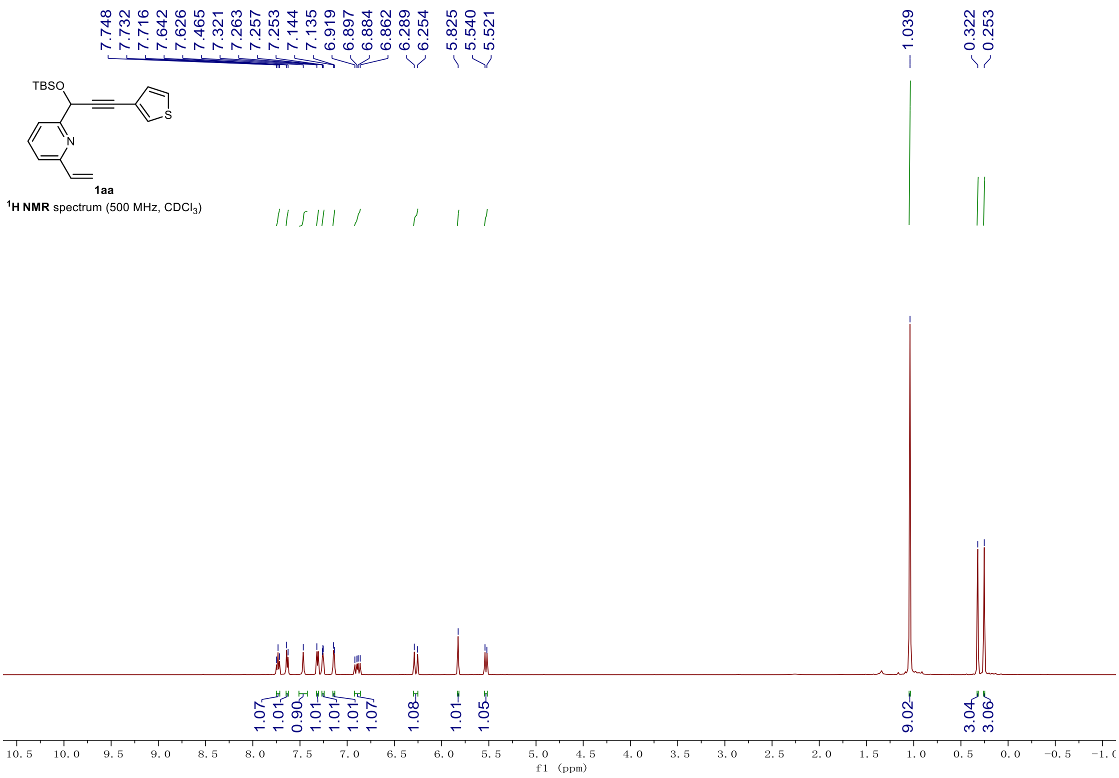

Supplementary Figure 58. ^{1}H NMR (500 MHz, $CDCl_3$) spectra for **1aa**

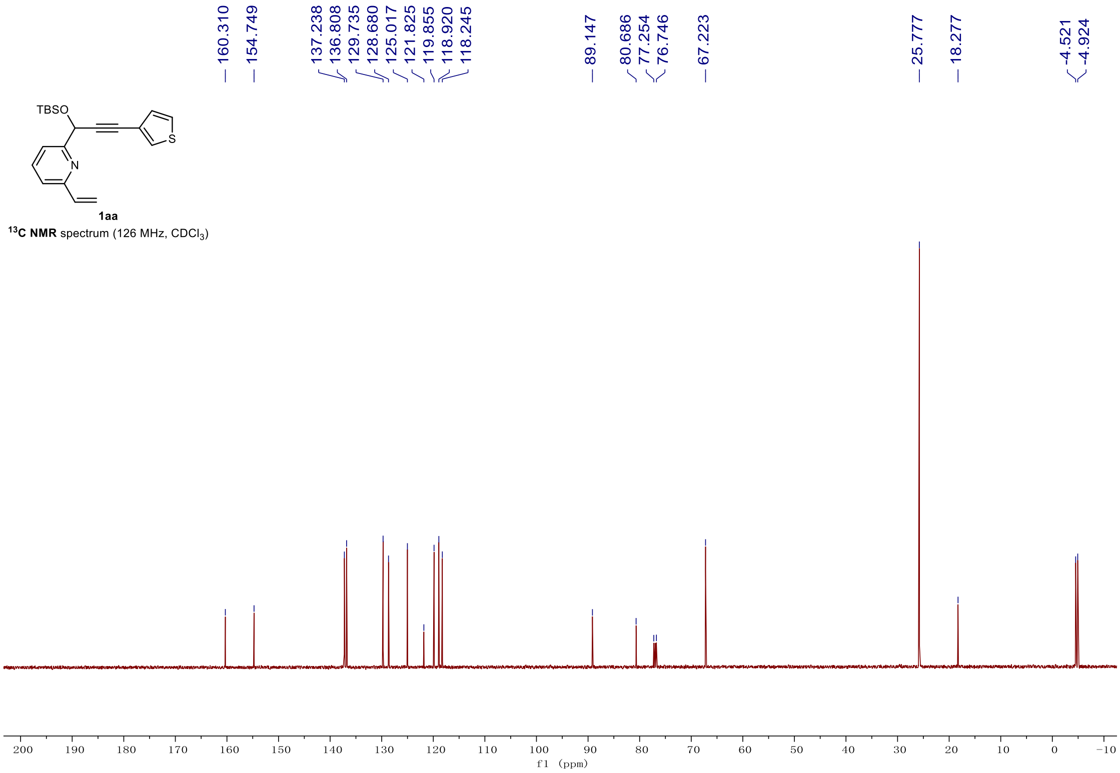

Supplementary Figure 59. ^{13}C NMR (126 MHz, $CDCl_3$) spectra for **1aa**

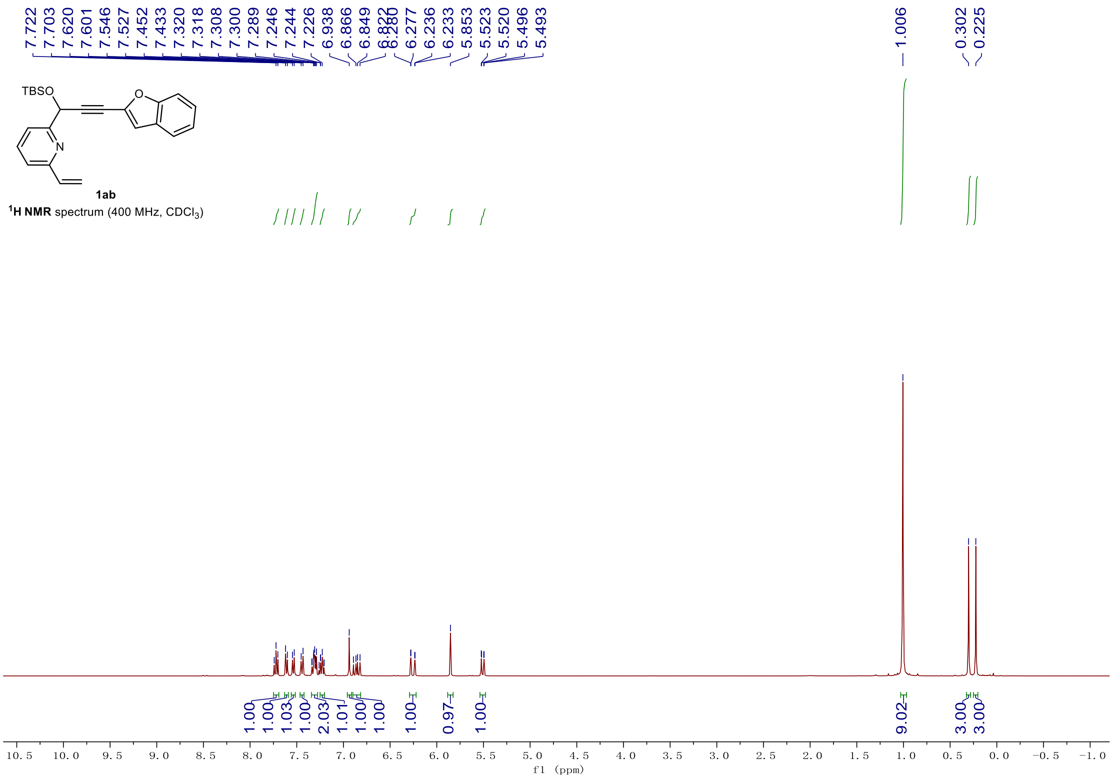

Supplementary Figure 60. ^{1}H NMR (400 MHz, $CDCl_3$) spectra for **1ab**

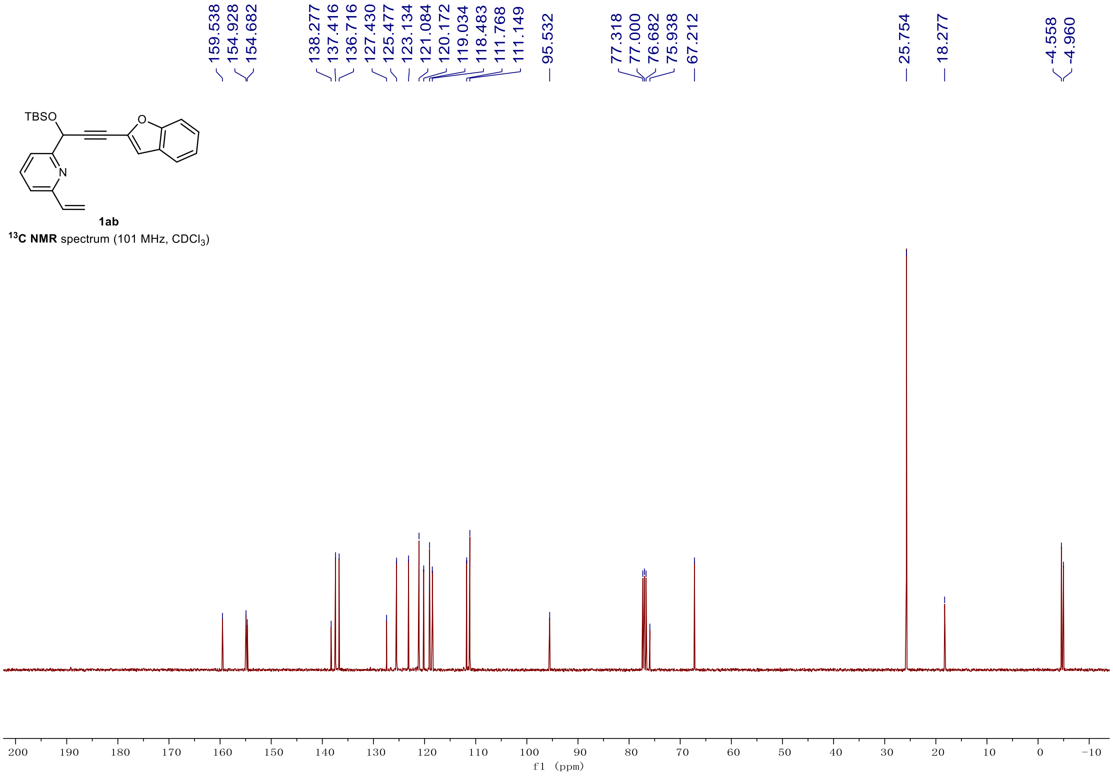

Supplementary Figure 61. ^{13}C NMR (101 MHz, $CDCl_3$) spectra for **1ab**

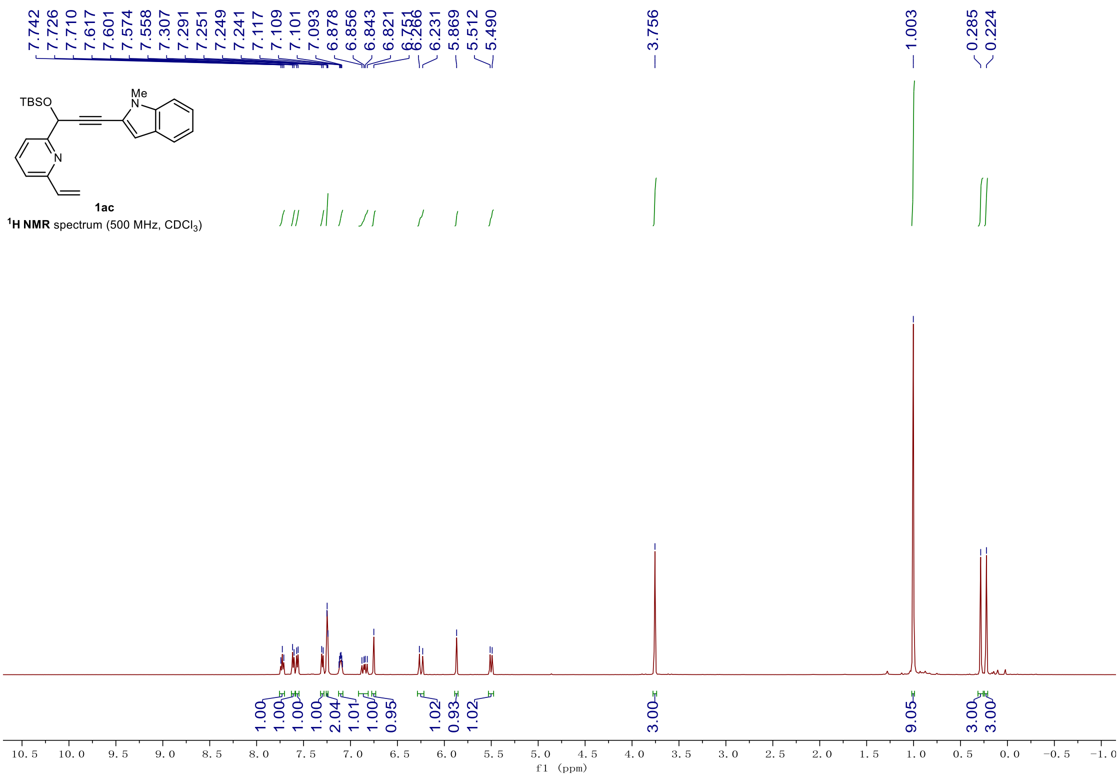

Supplementary Figure 62. ^{1}H NMR (500 MHz, $CDCl_3$) spectra for **1ac**

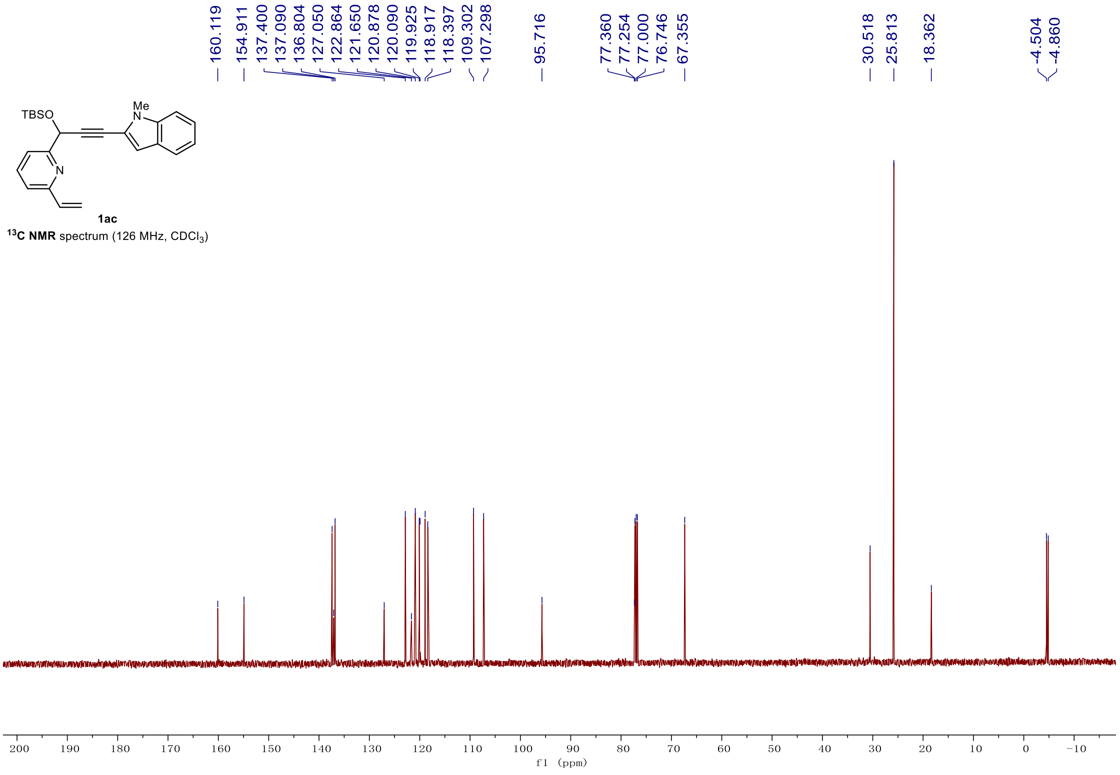

Supplementary Figure 63. ^{13}C NMR (126 MHz, $CDCl_3$) spectra for **1ac**

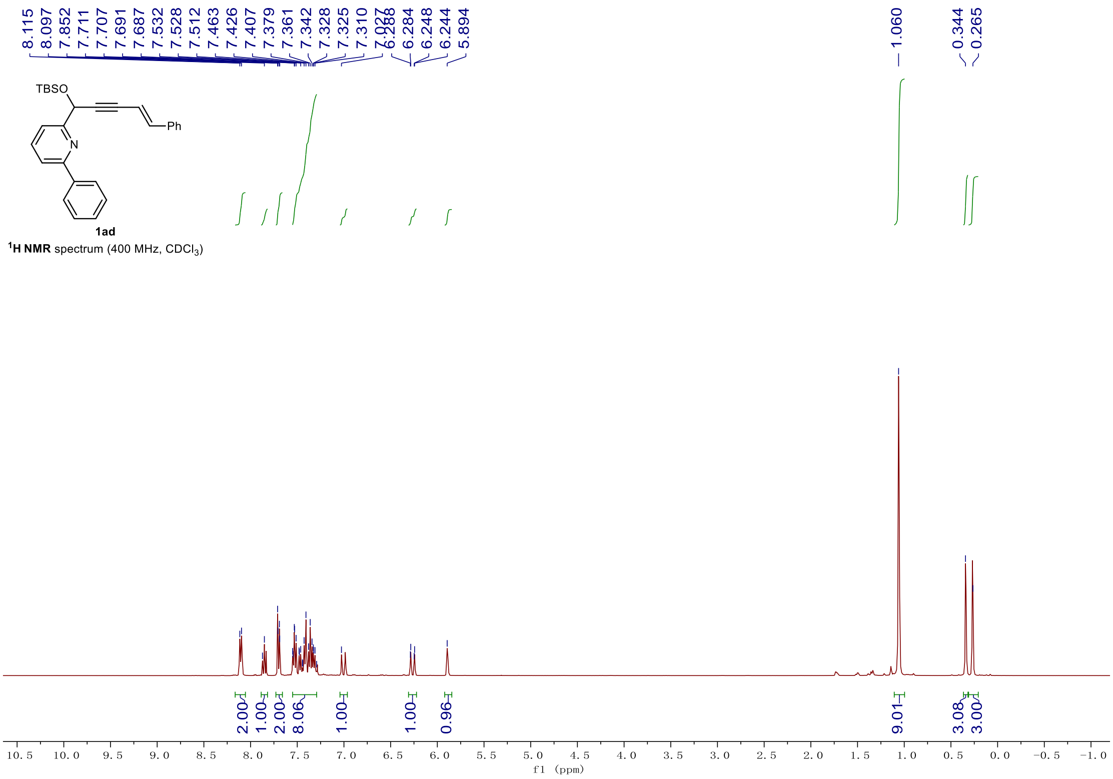

Supplementary Figure 64. ^{1}H NMR (400 MHz, $CDCl_3$) spectra for **1ad**

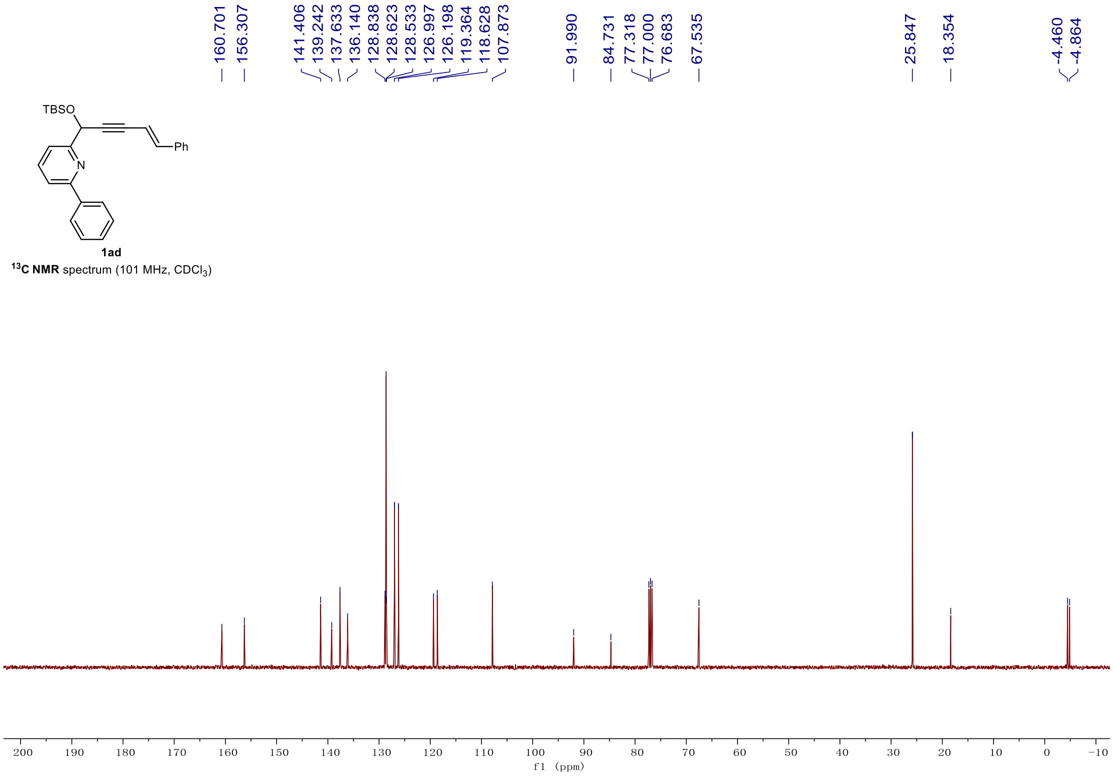

Supplementary Figure 65. ^{13}C NMR (101 MHz, $CDCl_3$) spectra for **1ad**

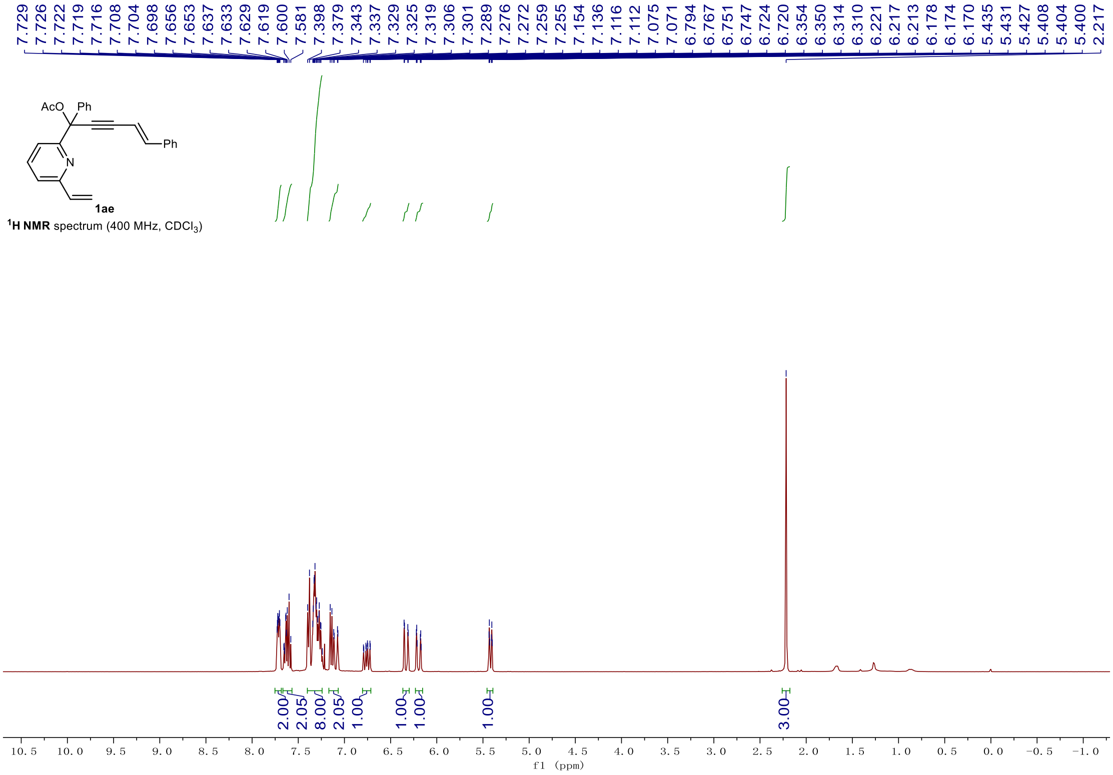

Supplementary Figure 66. ^{1}H NMR (400 MHz, $CDCl_3$) spectra for **1ae**

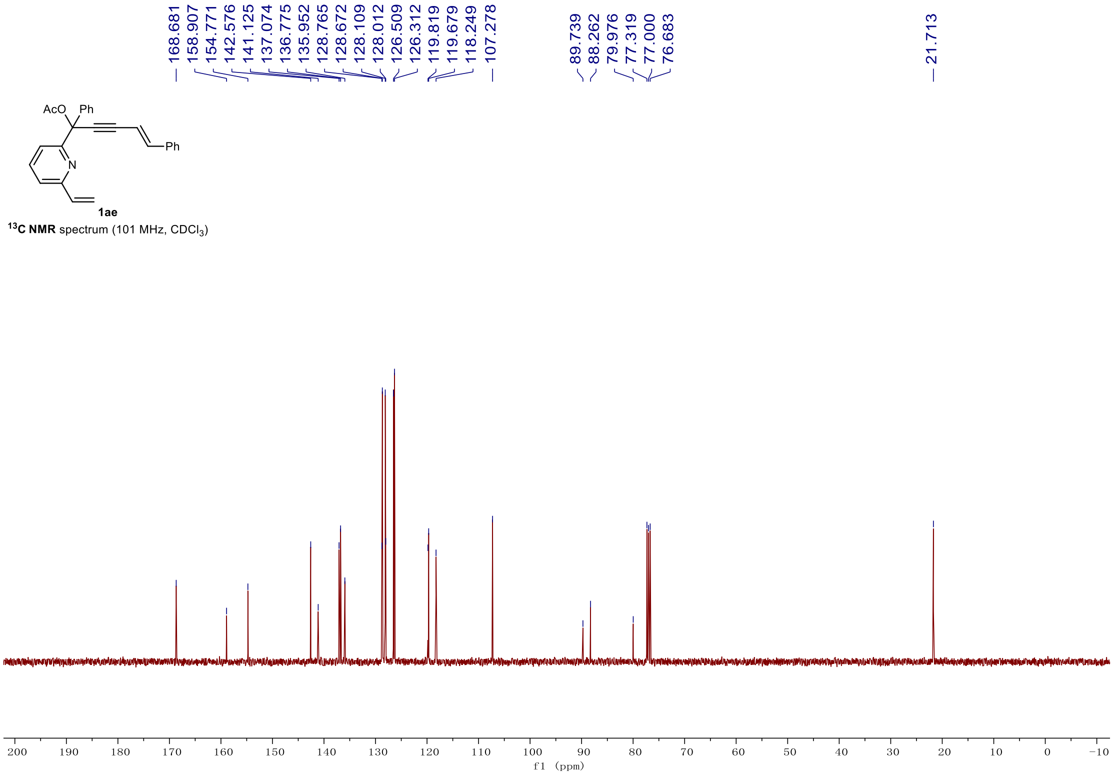

Supplementary Figure 67. ^{13}C NMR (101 MHz, $CDCl_3$) spectra for **1ae**

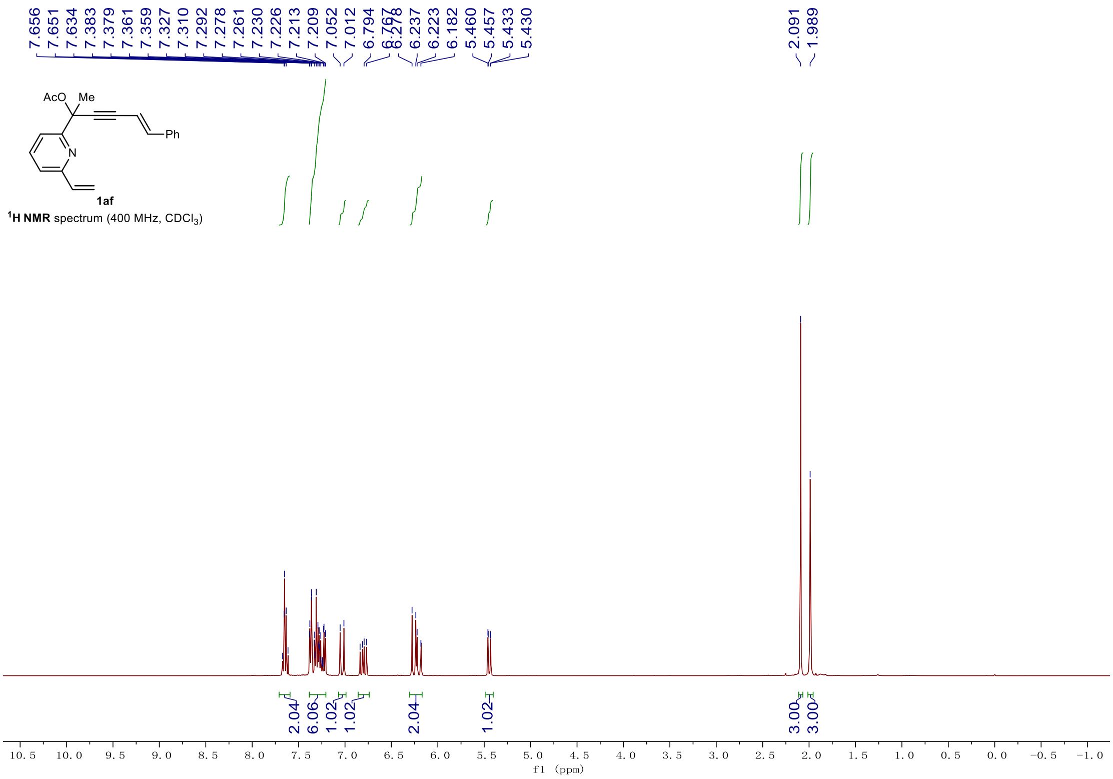

Supplementary Figure 68. ^{1}H NMR (400 MHz, $CDCl_3$) spectra for **1af**

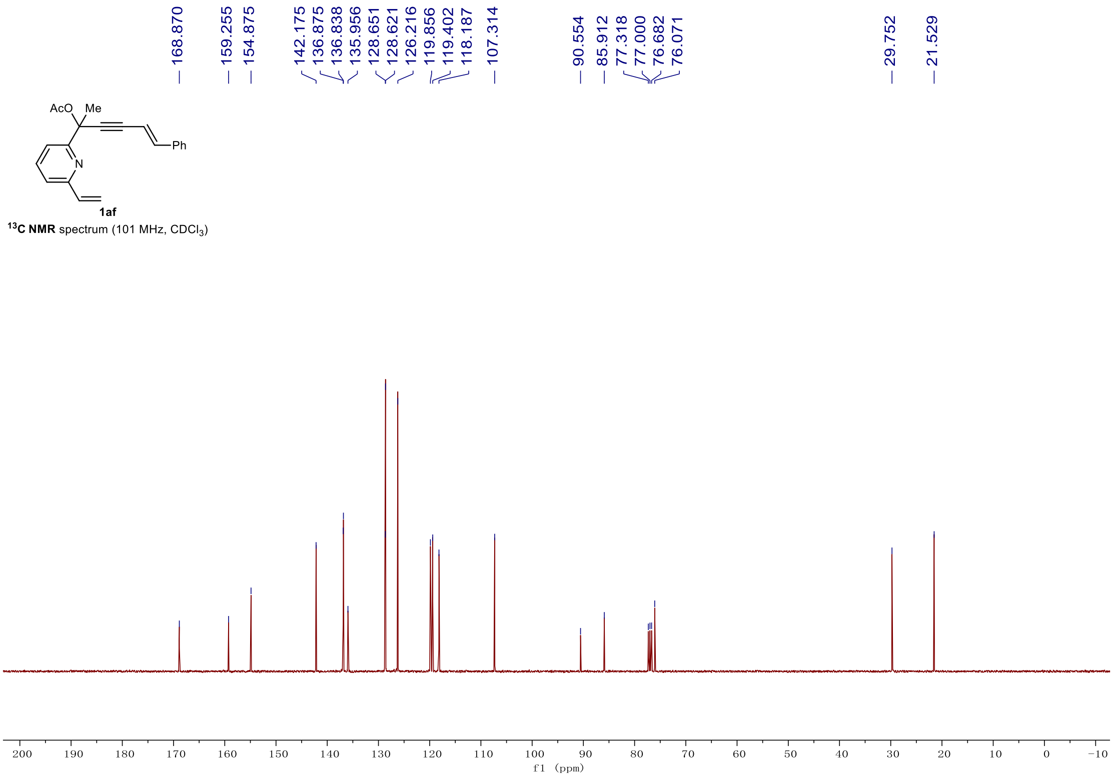

Supplementary Figure 69. ^{13}C NMR (101 MHz, $CDCl_3$) spectra for **1af**

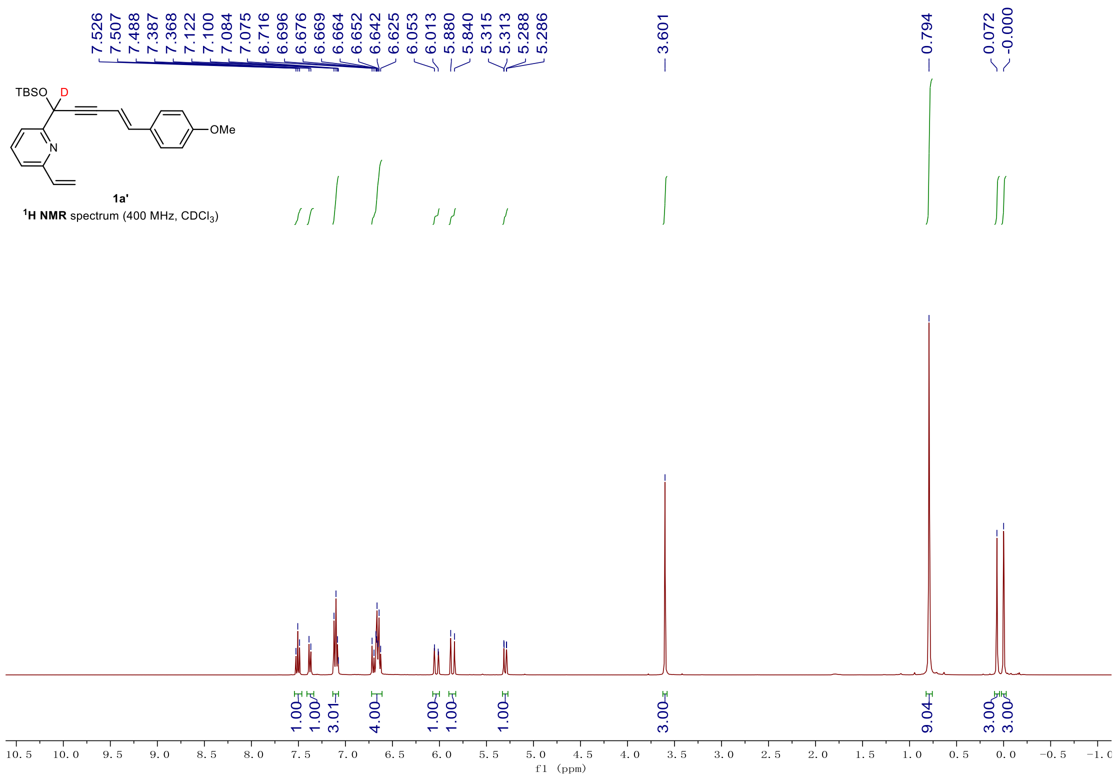

Supplementary Figure 70. ^{1}H NMR (400 MHz, $CDCl_3$) spectra for **1a'**

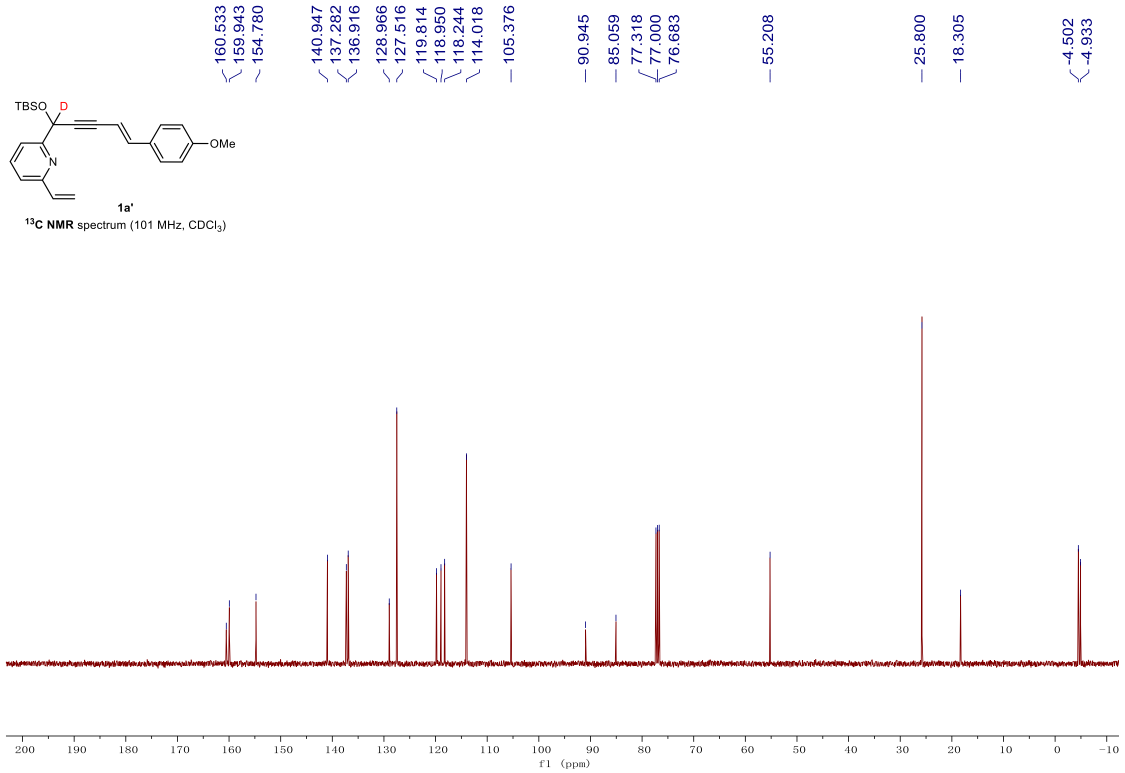

Supplementary Figure 71. ^{13}C NMR (101 MHz, $CDCl_3$) spectra for **1a'**

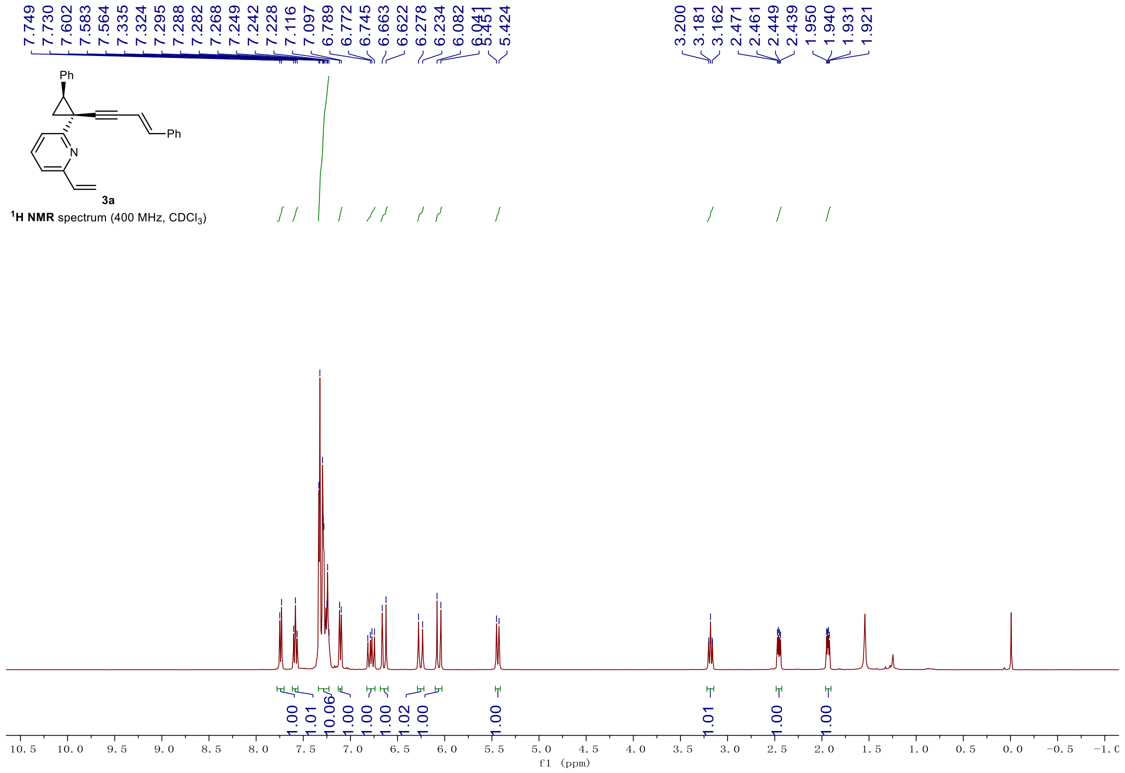

Supplementary Figure 72. ^{1}H NMR (400 MHz, $CDCl_3$) spectra for **3a**

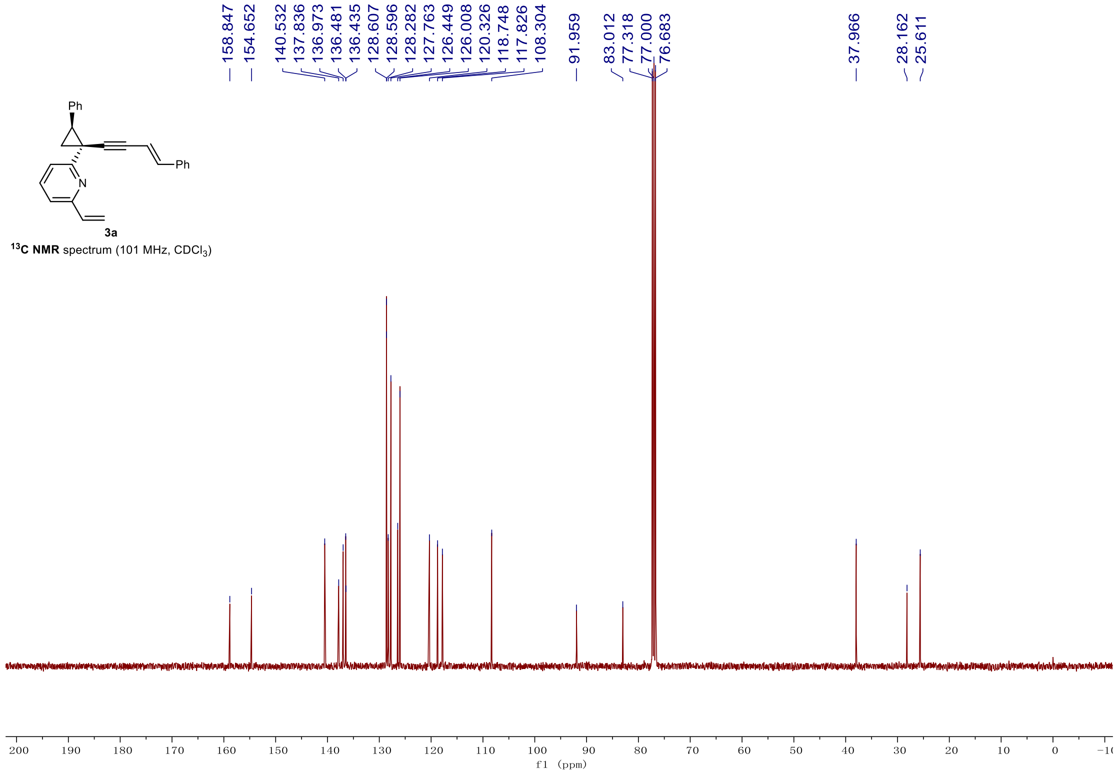

Supplementary Figure 73. ^{13}C NMR (101 MHz, $CDCl_3$) spectra for **3a**

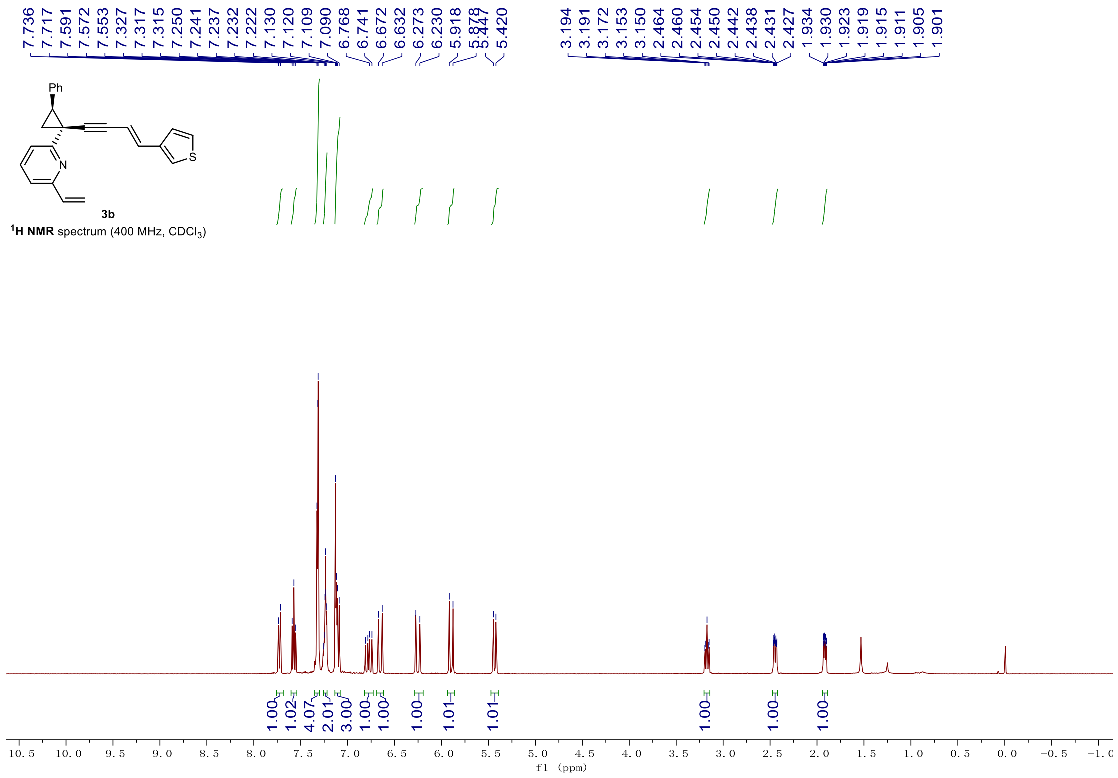

Supplementary Figure 74. ^{1}H NMR (400 MHz, $CDCl_3$) spectra for **3b**

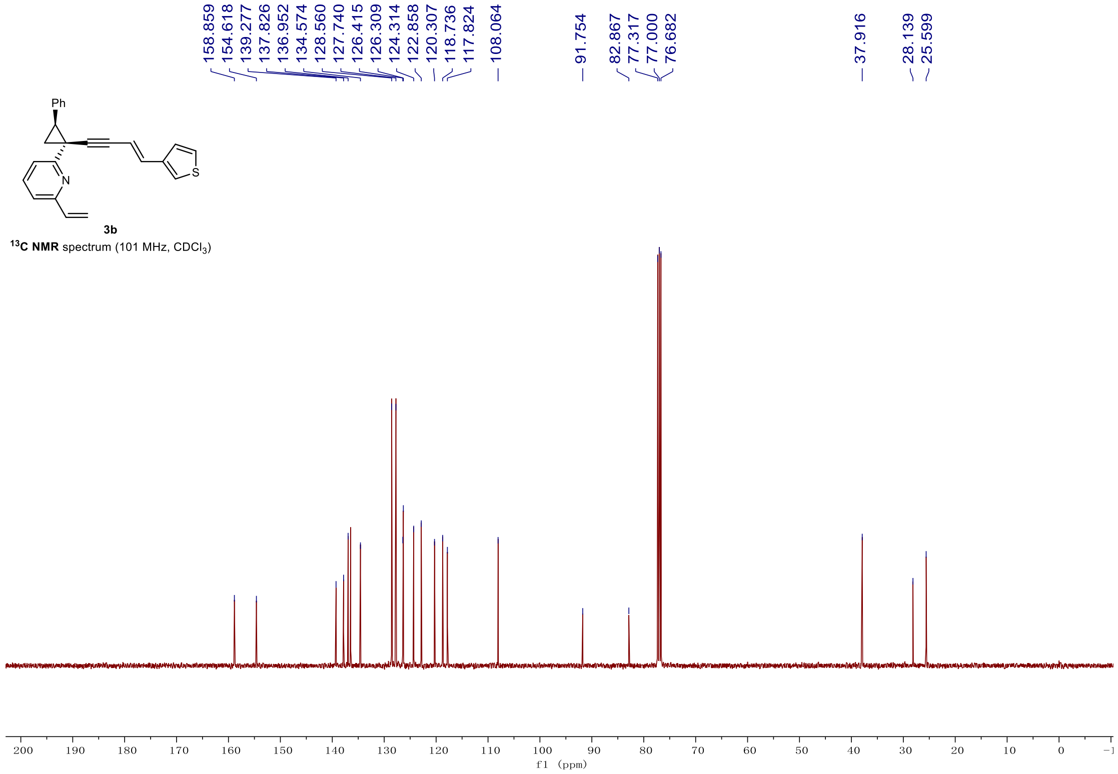

Supplementary Figure 75. ^{13}C NMR (101 MHz, $CDCl_3$) spectra for **3b**

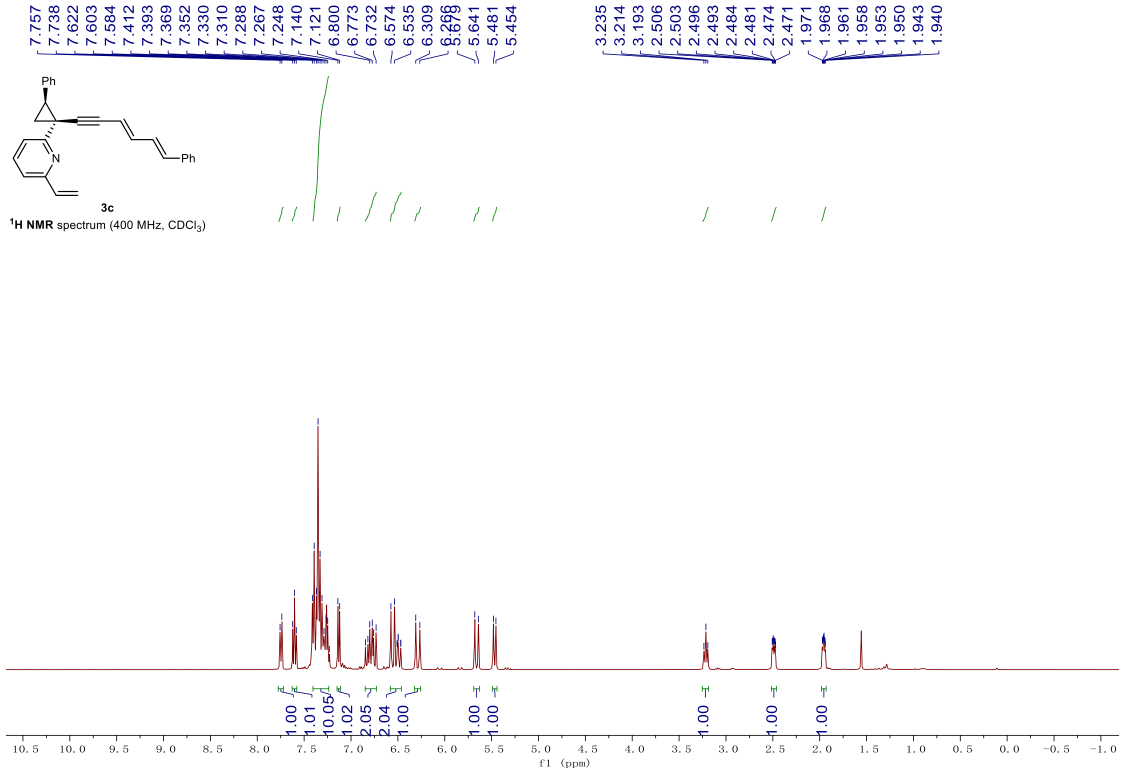

Supplementary Figure 76. ^{1}H NMR (400 MHz, $CDCl_3$) spectra for **3c**

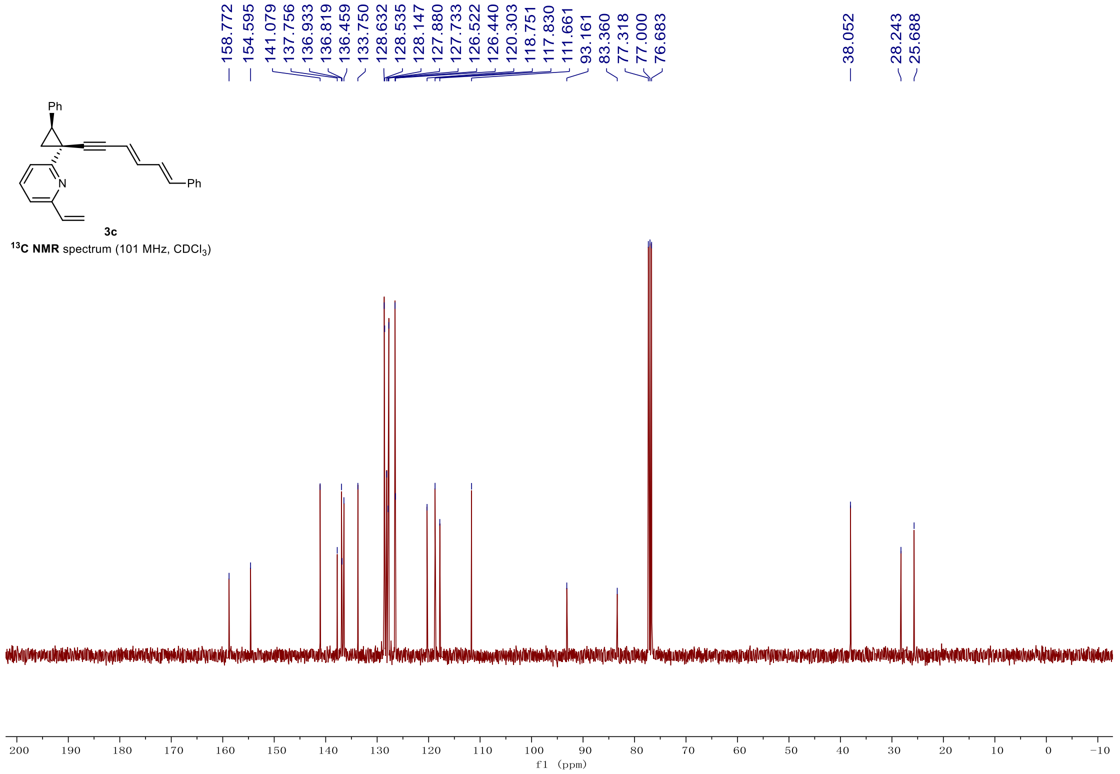

Supplementary Figure 77. ^{13}C NMR (101 MHz, $CDCl_3$) spectra for **3c**

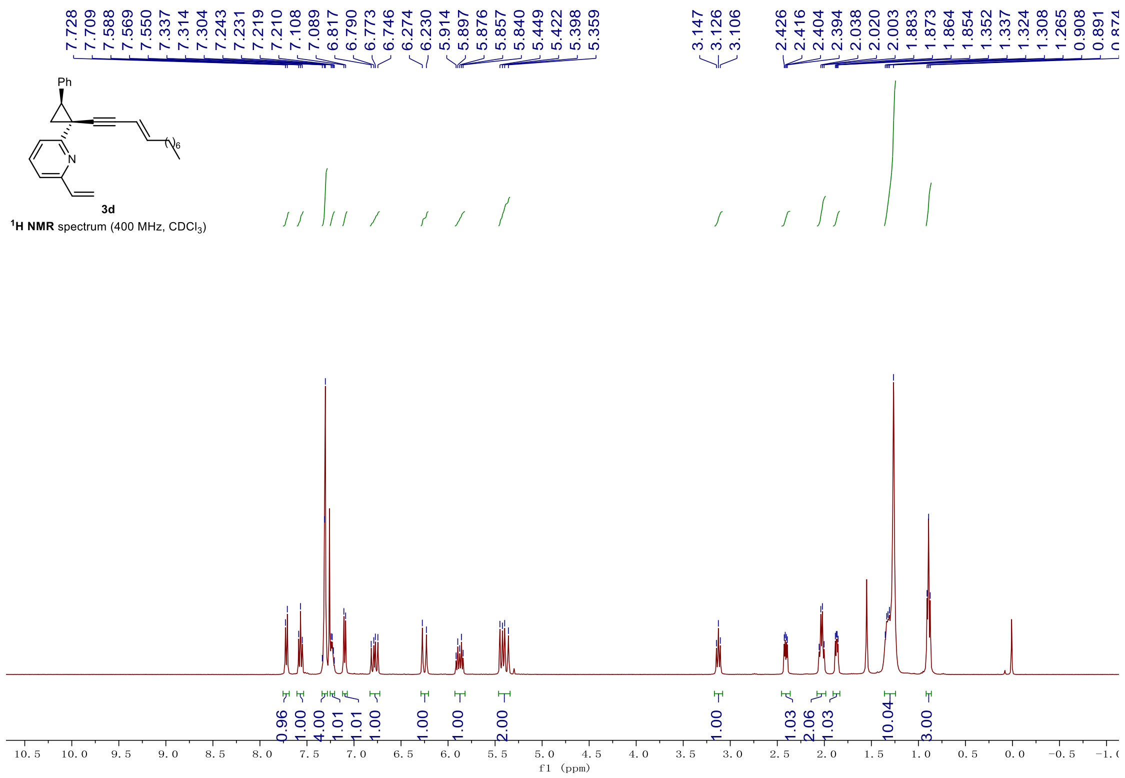

Supplementary Figure 78. ^{1}H NMR (400 MHz, $CDCl_3$) spectra for **3d**

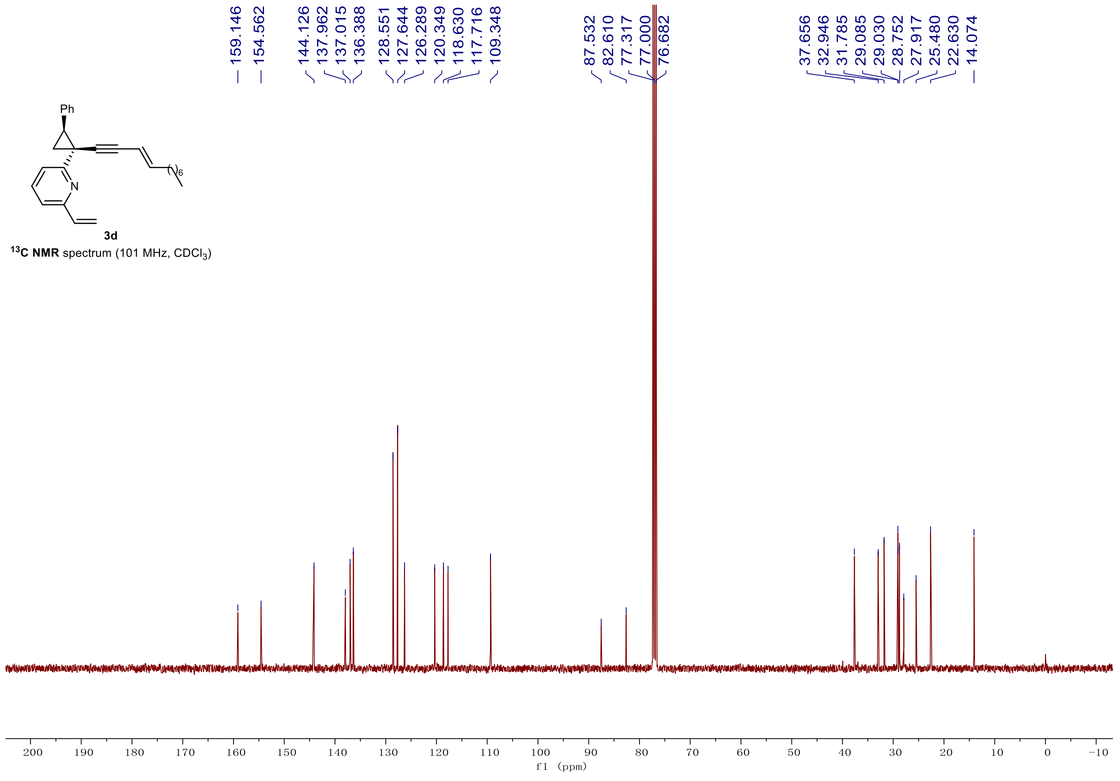

Supplementary Figure 79. ^{13}C NMR (101 MHz, $CDCl_3$) spectra for **3d**

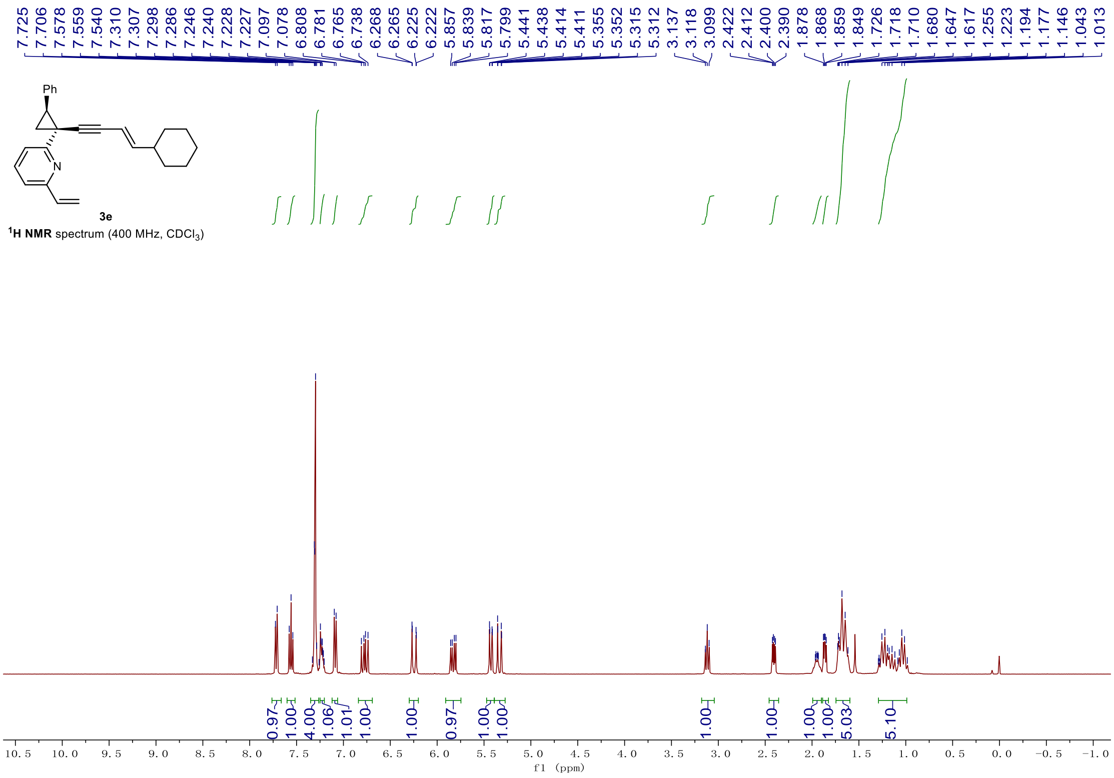

Supplementary Figure 80. ^{1}H NMR (400 MHz, $CDCl_3$) spectra for **3e**

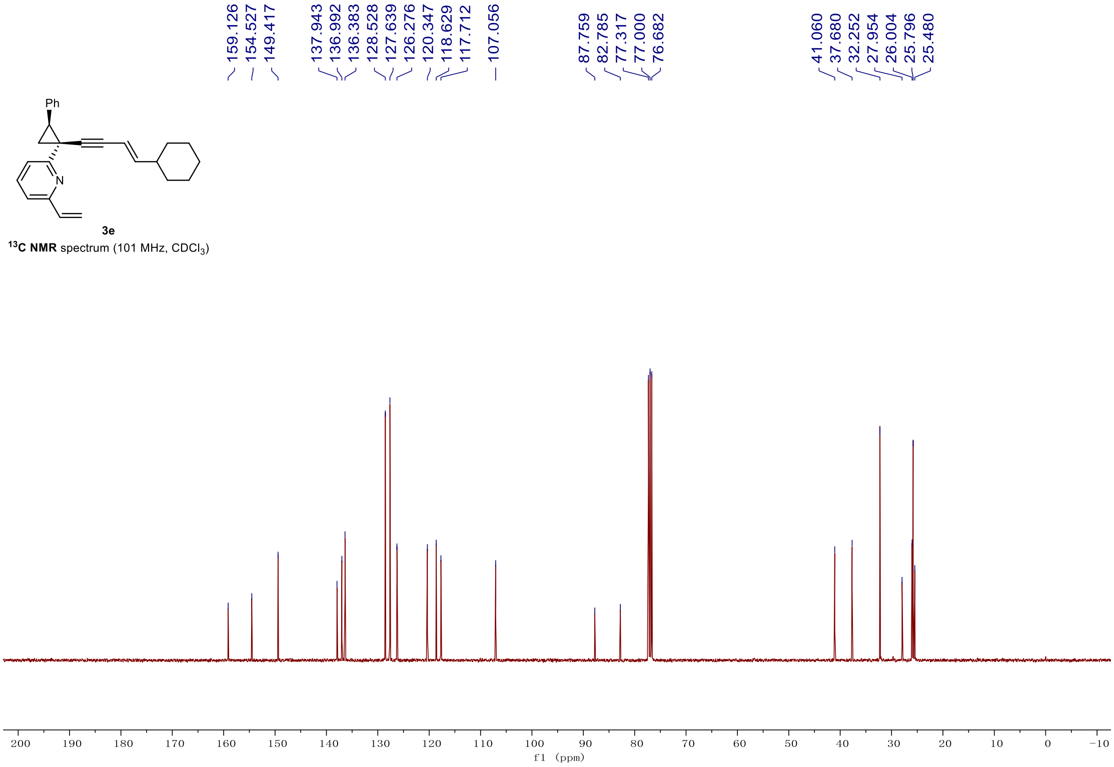

Supplementary Figure 81. ^{13}C NMR (101 MHz, $CDCl_3$) spectra for **3e**

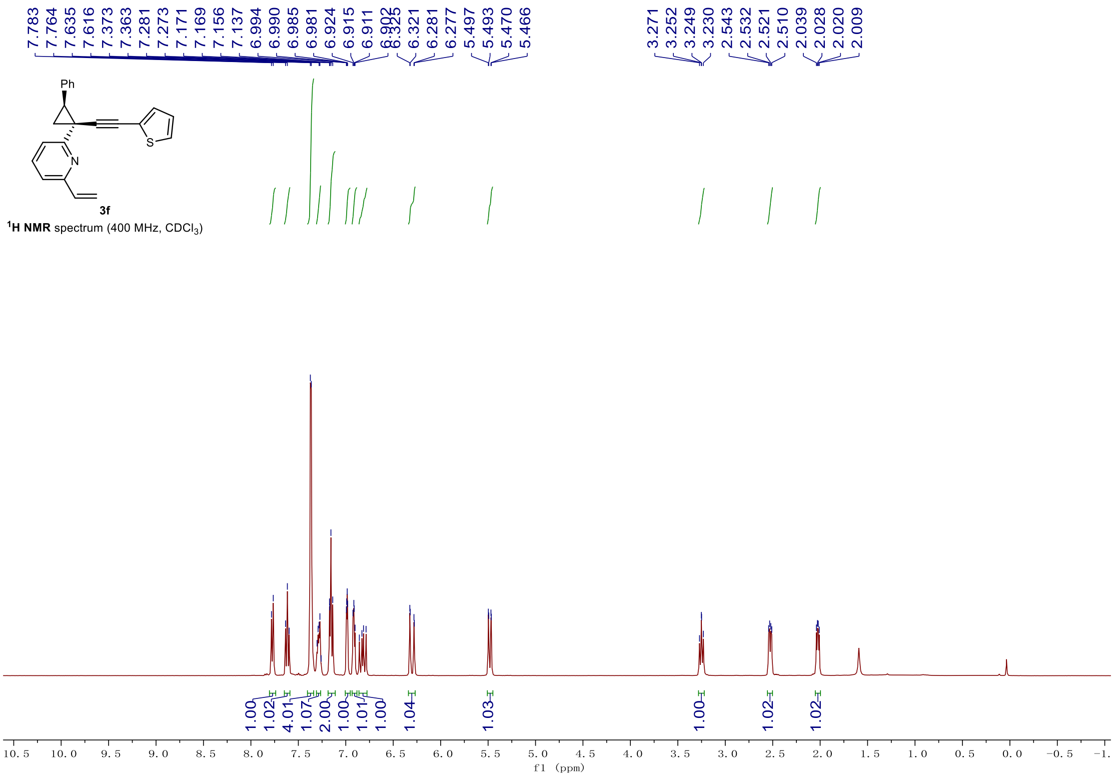

Supplementary Figure 82. ^{1}H NMR (400 MHz, $CDCl_3$) spectra for **3f**

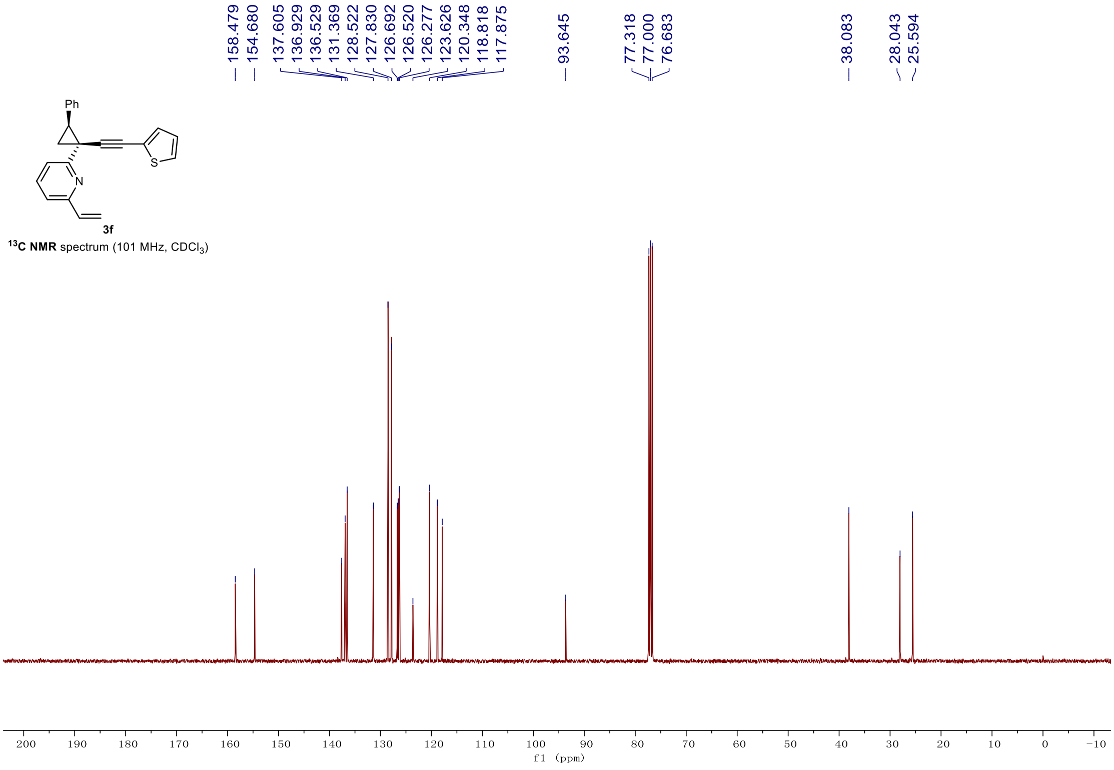

Supplementary Figure 83. ^{13}C NMR (101 MHz, $CDCl_3$) spectra for **3f**

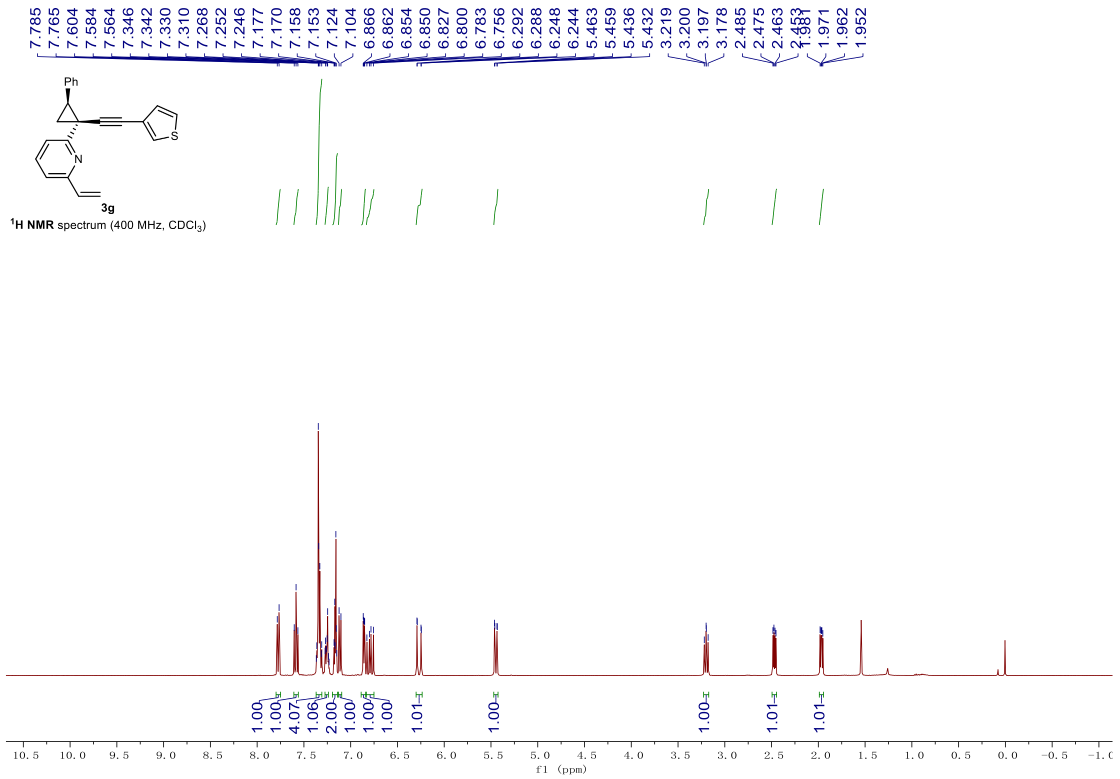

Supplementary Figure 84. ^{1}H NMR (400 MHz, $CDCl_3$) spectra for **3g**

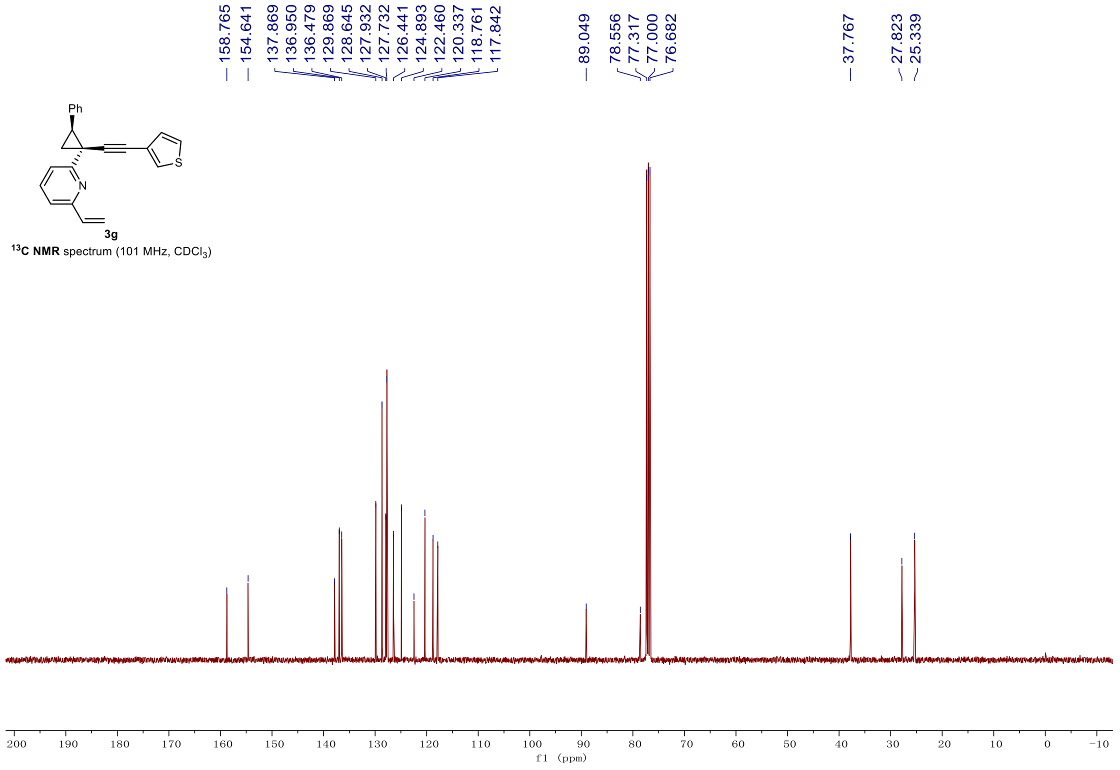

Supplementary Figure 85. ^{13}C NMR (101 MHz, $CDCl_3$) spectra for **3g**

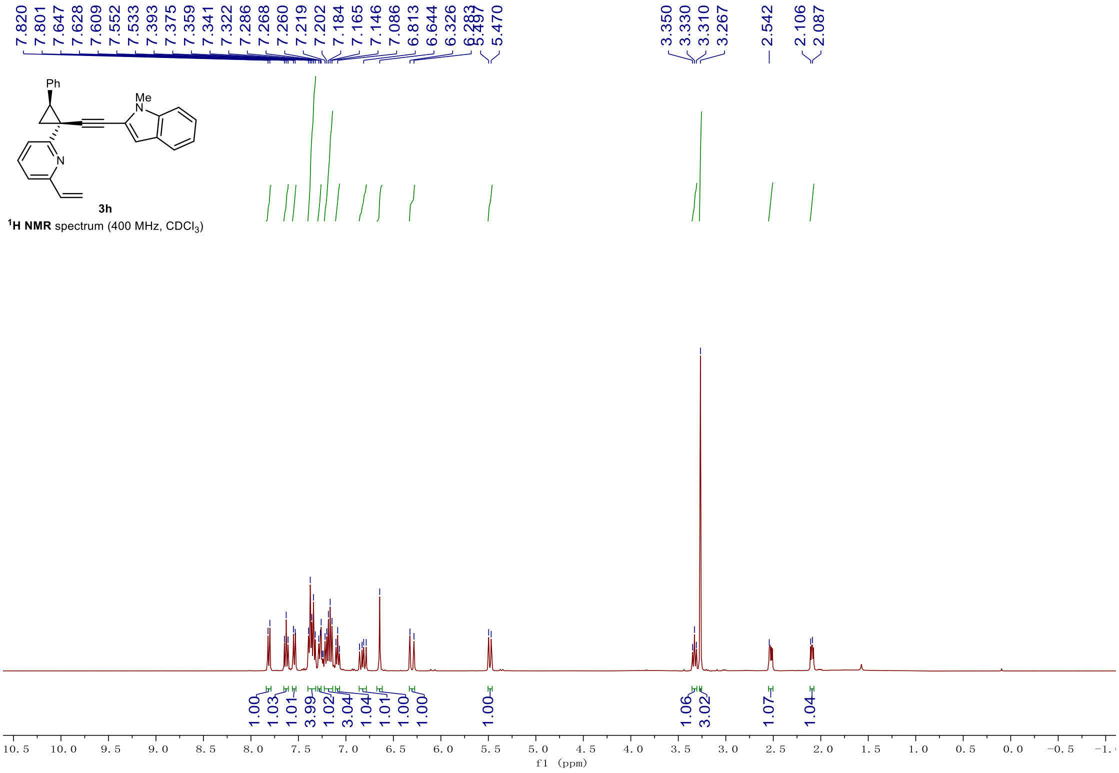

Supplementary Figure 86. ^{1}H NMR (400 MHz, $CDCl_3$) spectra for **3h**

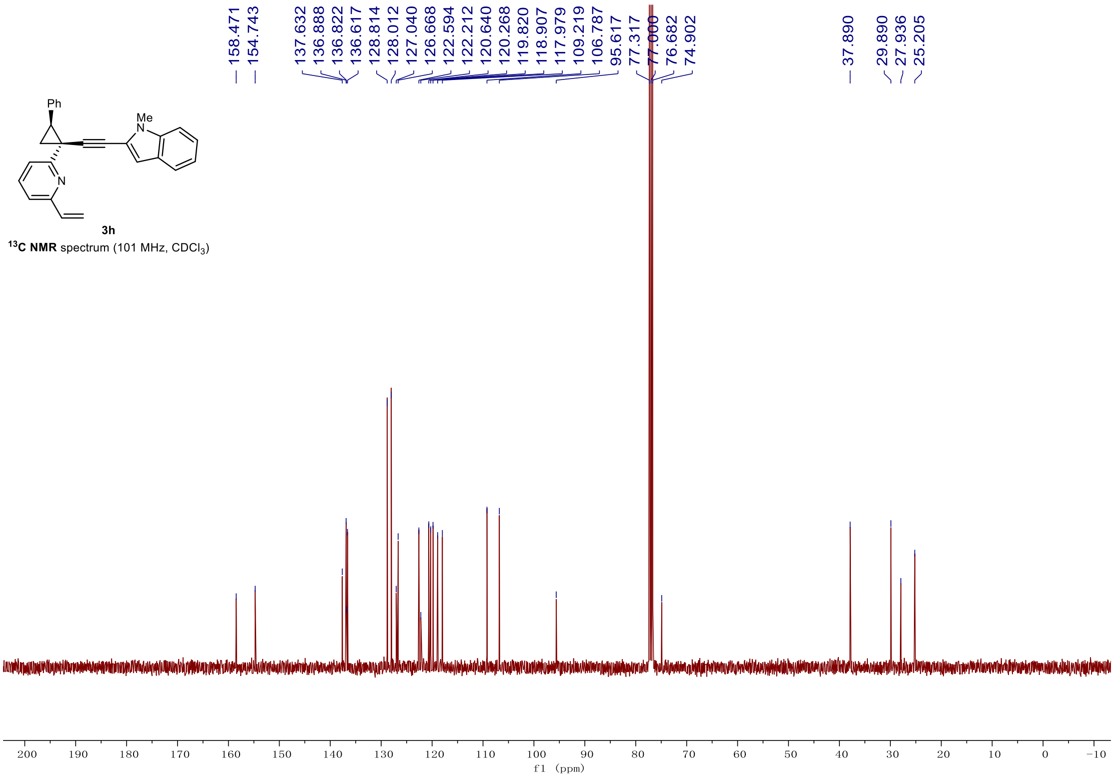

Supplementary Figure 87. ^{13}C NMR (101 MHz, $CDCl_3$) spectra for **3h**

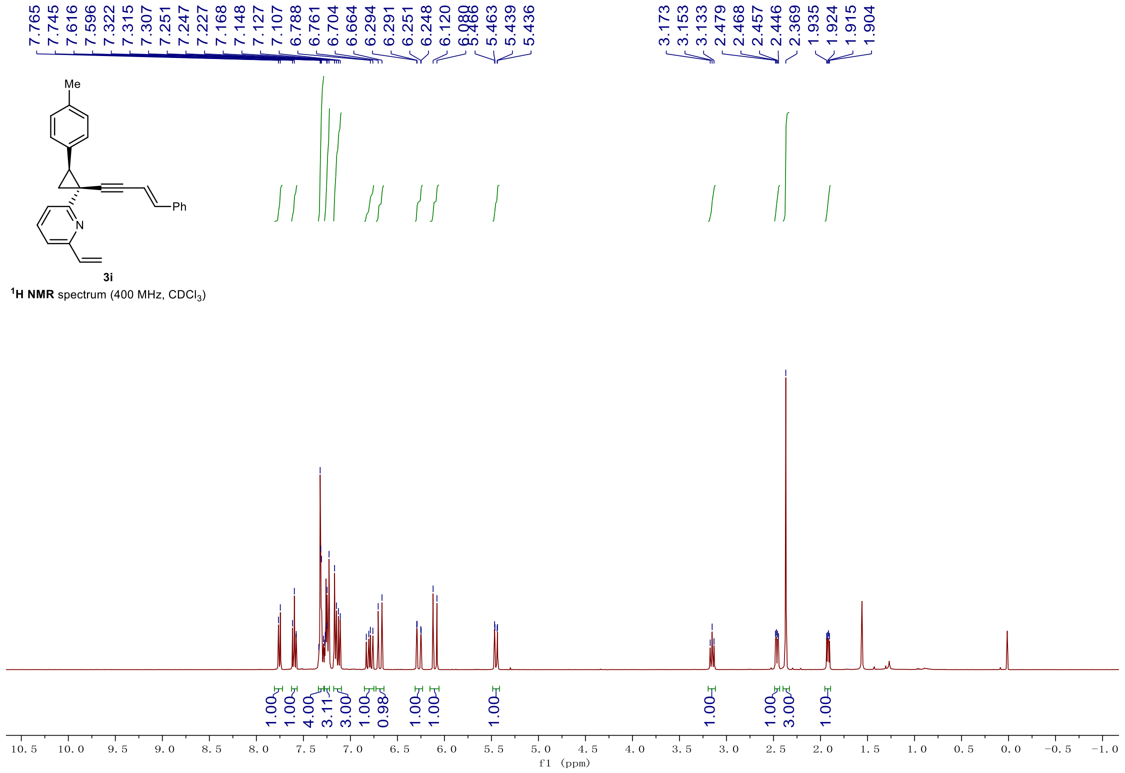

Supplementary Figure 88. ^{1}H NMR (400 MHz, $CDCl_3$) spectra for **3i**

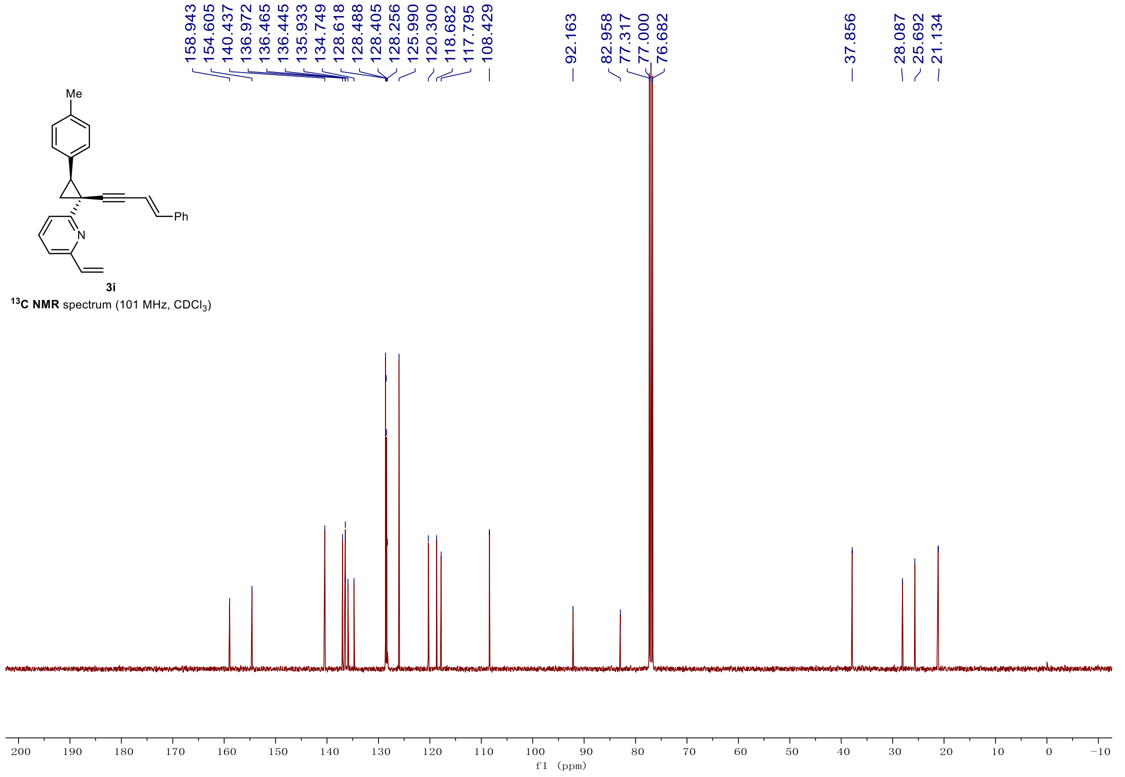

Supplementary Figure 89. ^{13}C NMR (101 MHz, $CDCl_3$) spectra for **3i**

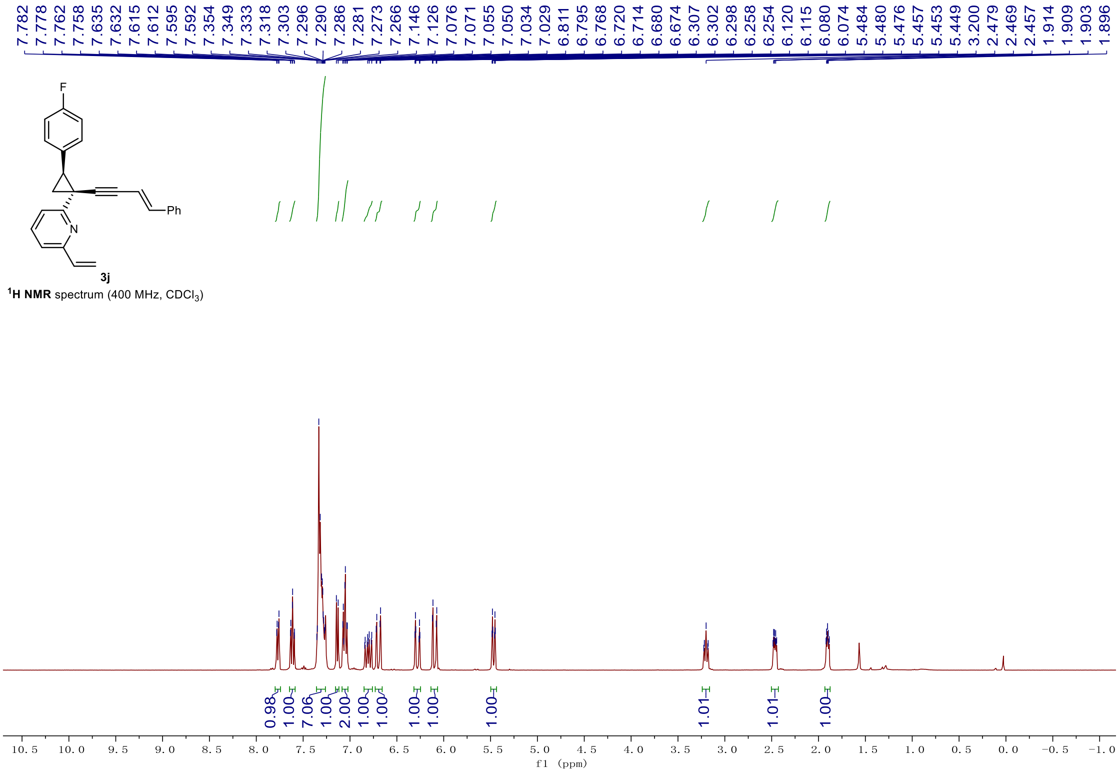

Supplementary Figure 90. ^{1}H NMR (400 MHz, $CDCl_3$) spectra for **3j**

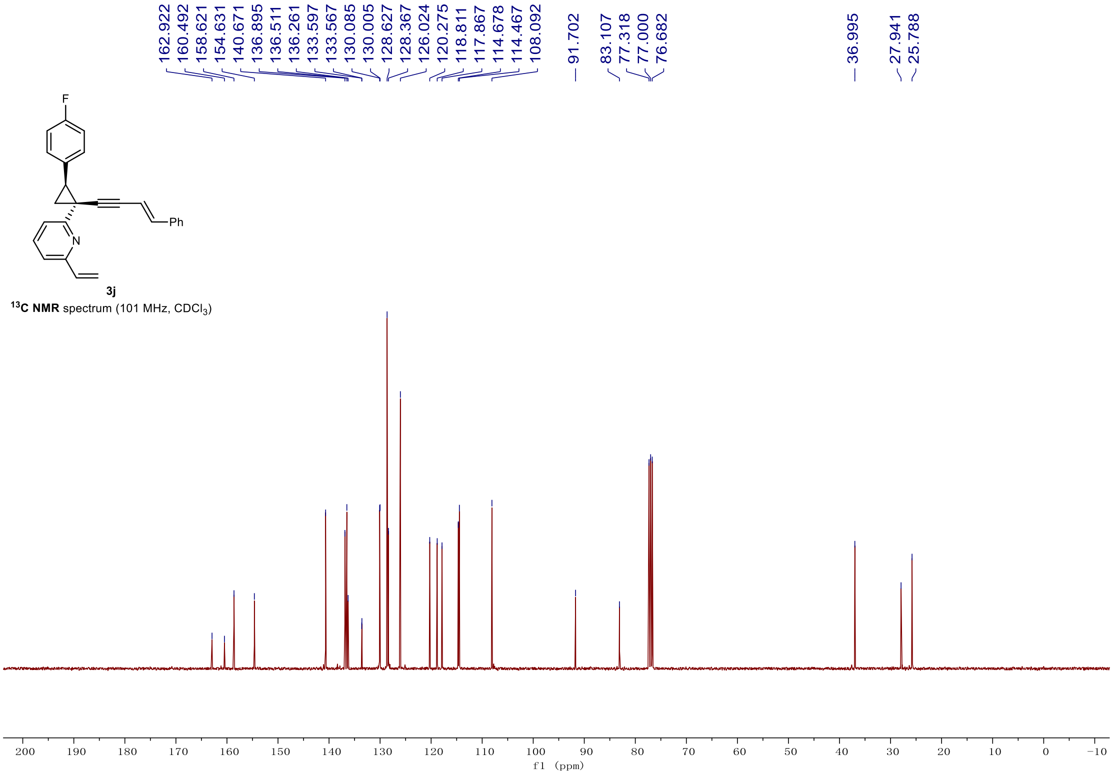

Supplementary Figure 91. ^{13}C NMR (101 MHz, $CDCl_3$) spectra for **3j**

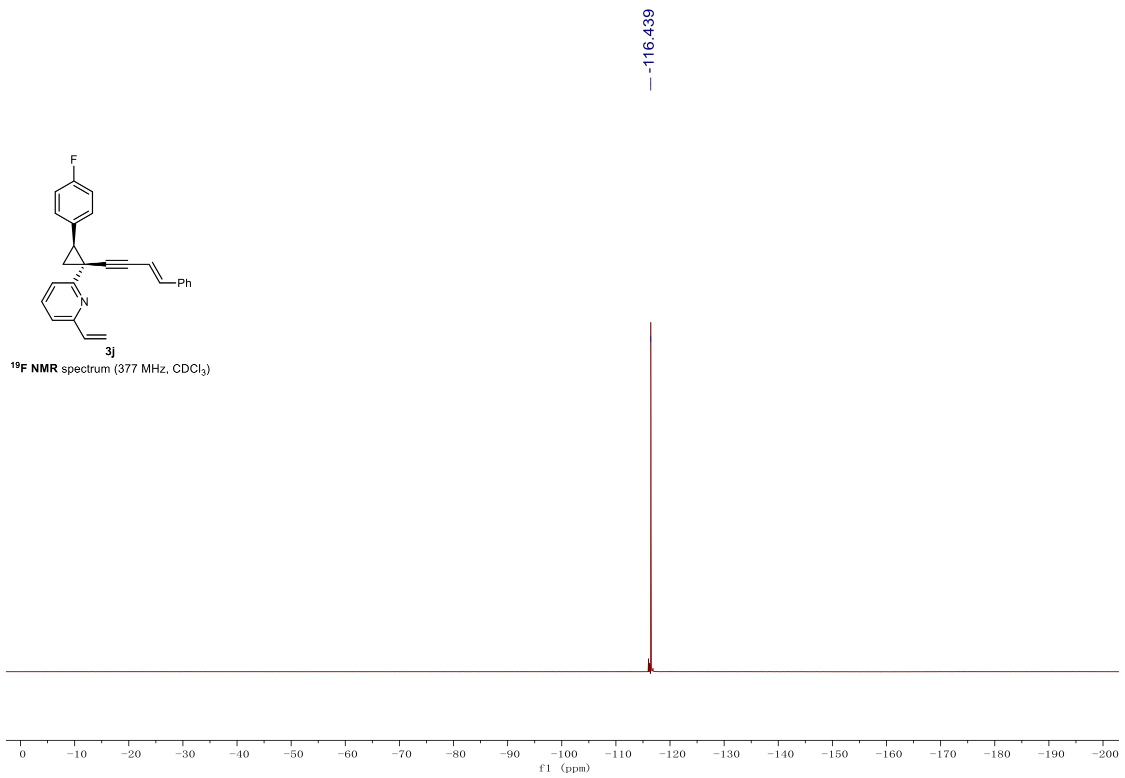

Supplementary Figure 92. ^{19}F NMR (377 MHz, $CDCl_3$) spectra for **3j**

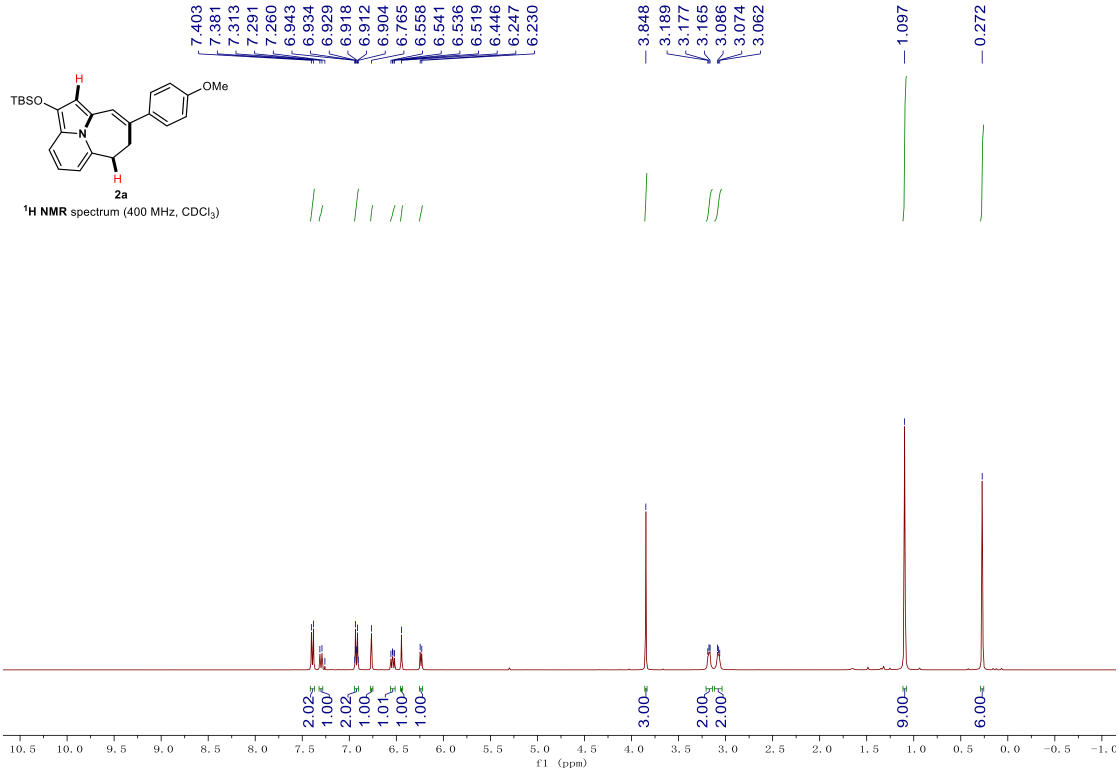

Supplementary Figure 93. ^{1}H NMR (400 MHz, $CDCl_3$) spectra for **2a**

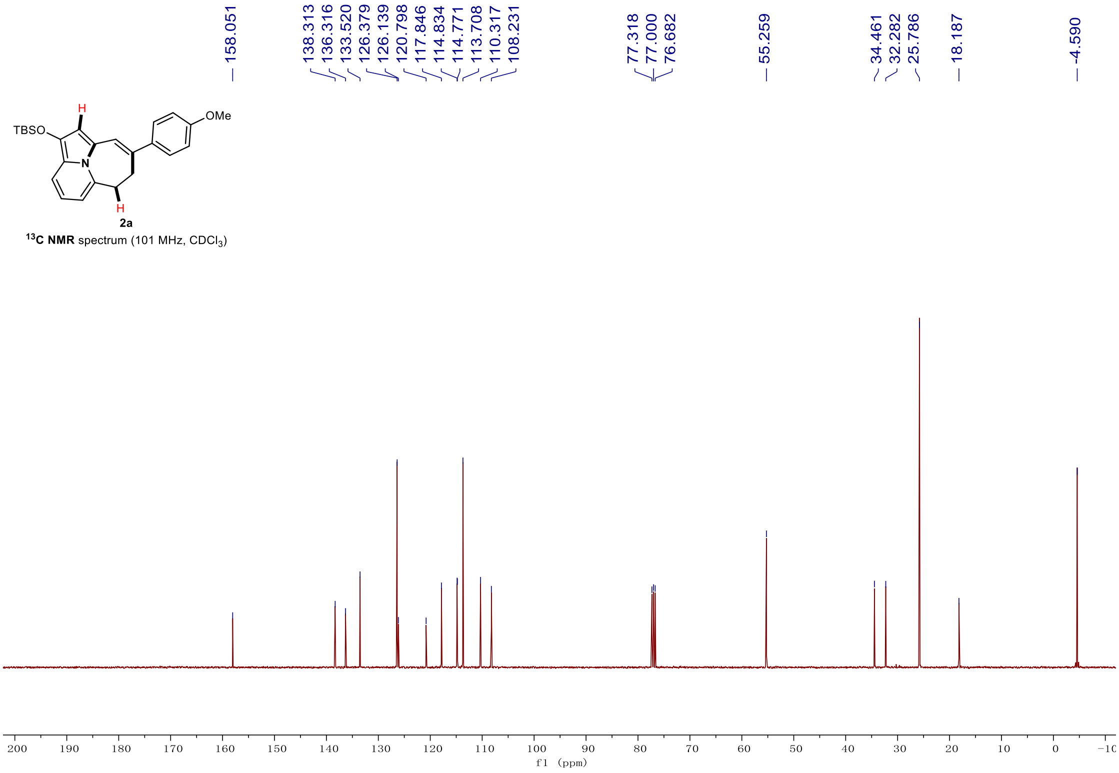

Supplementary Figure 94. ^{13}C NMR (101 MHz, $CDCl_3$) spectra for **2a**

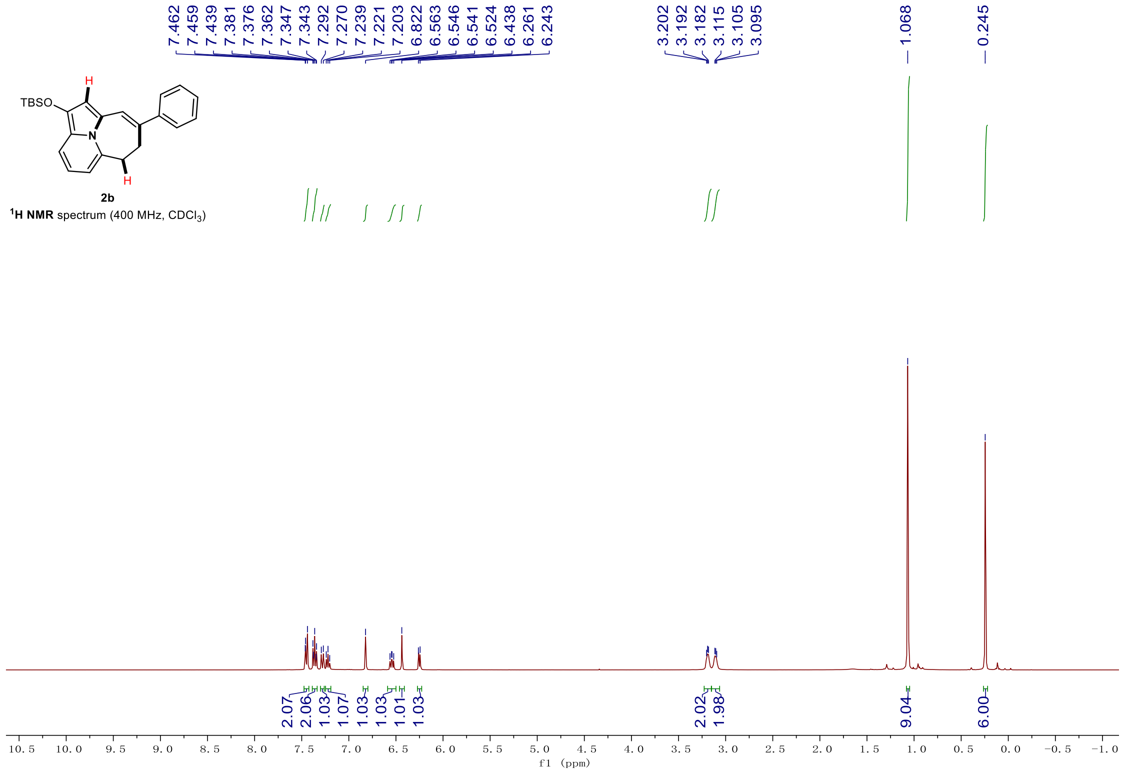

Supplementary Figure 95. ^{1}H NMR (400 MHz, $CDCl_3$) spectra for **2b**

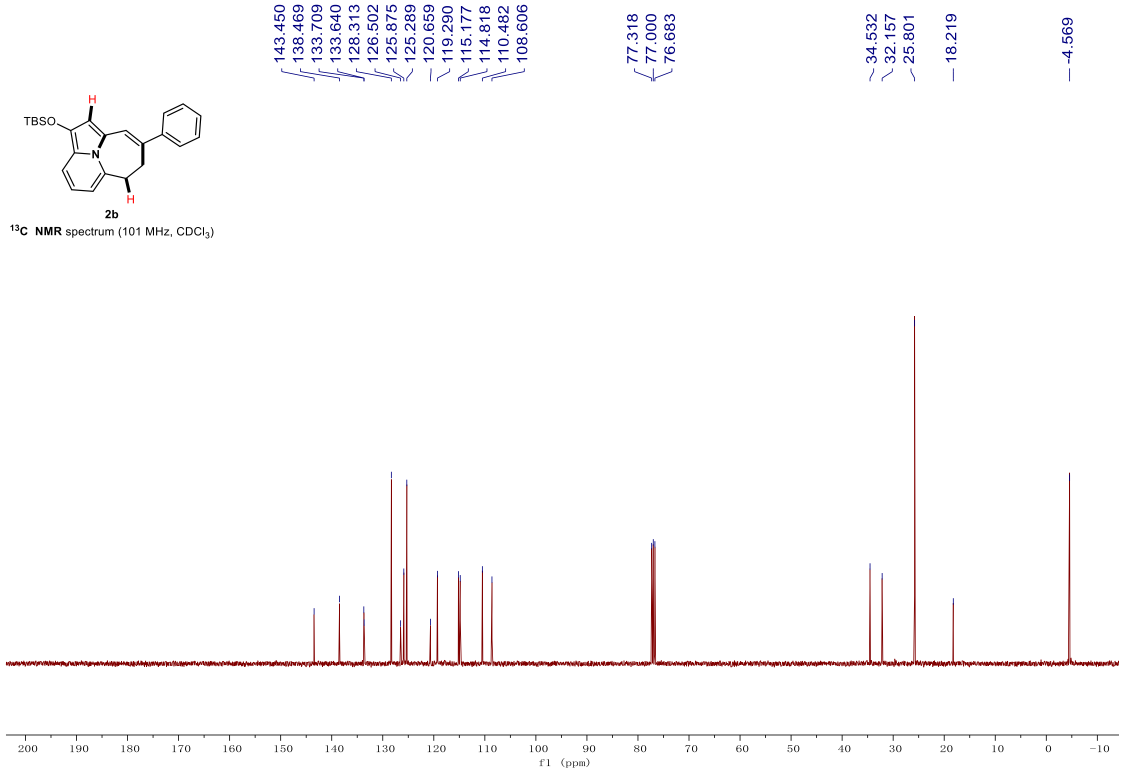

Supplementary Figure 96. ^{13}C NMR (101 MHz, $CDCl_3$) spectra for **2b**

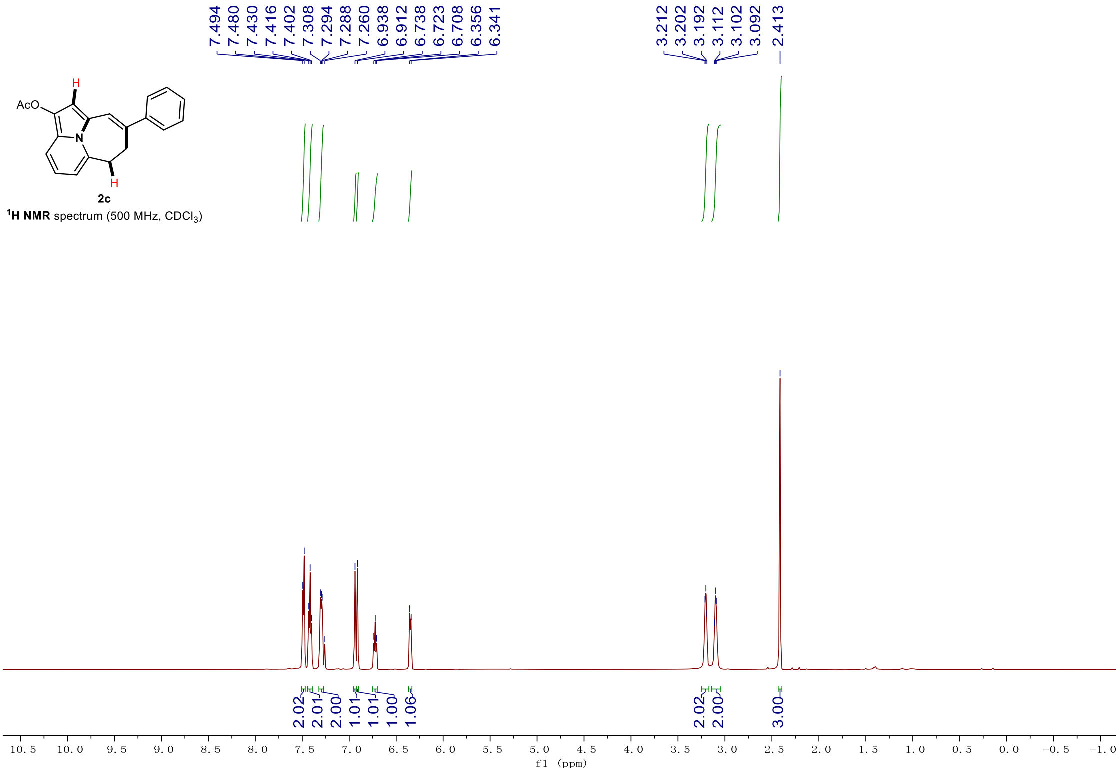

Supplementary Figure 97. ^{1}H NMR (500 MHz, $CDCl_3$) spectra for **2c**

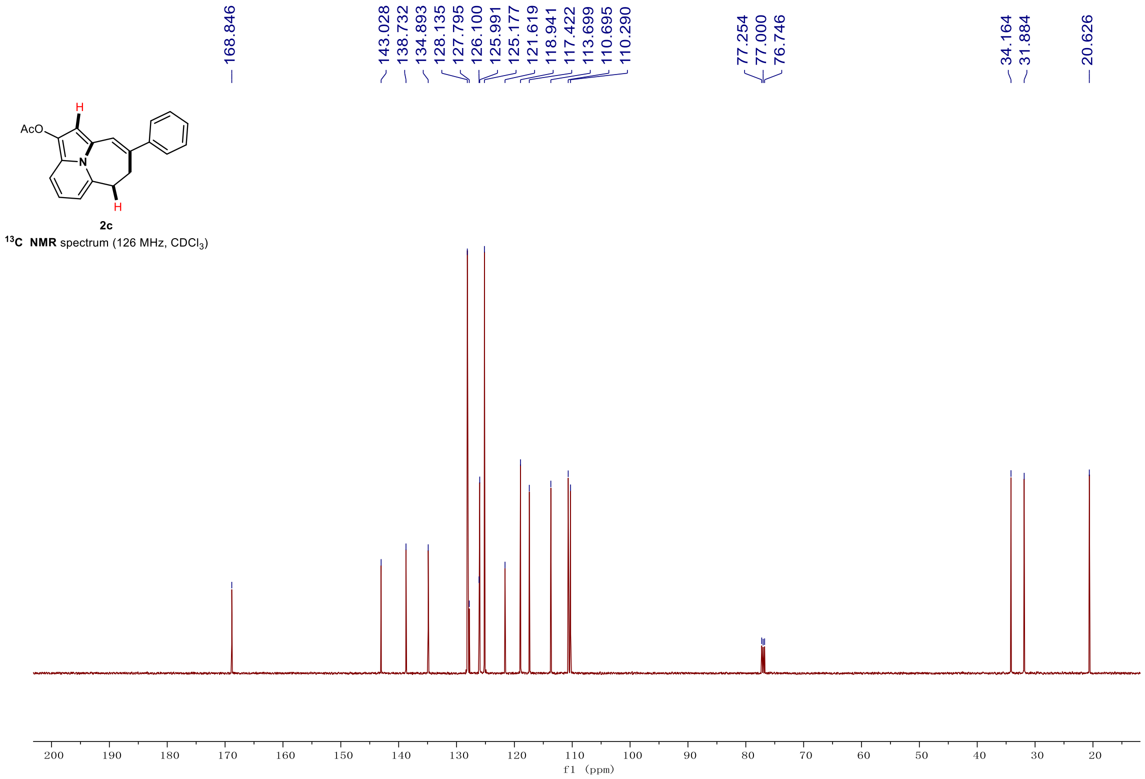

Supplementary Figure 98. ^{13}C NMR (126 MHz, $CDCl_3$) spectra for **2c**

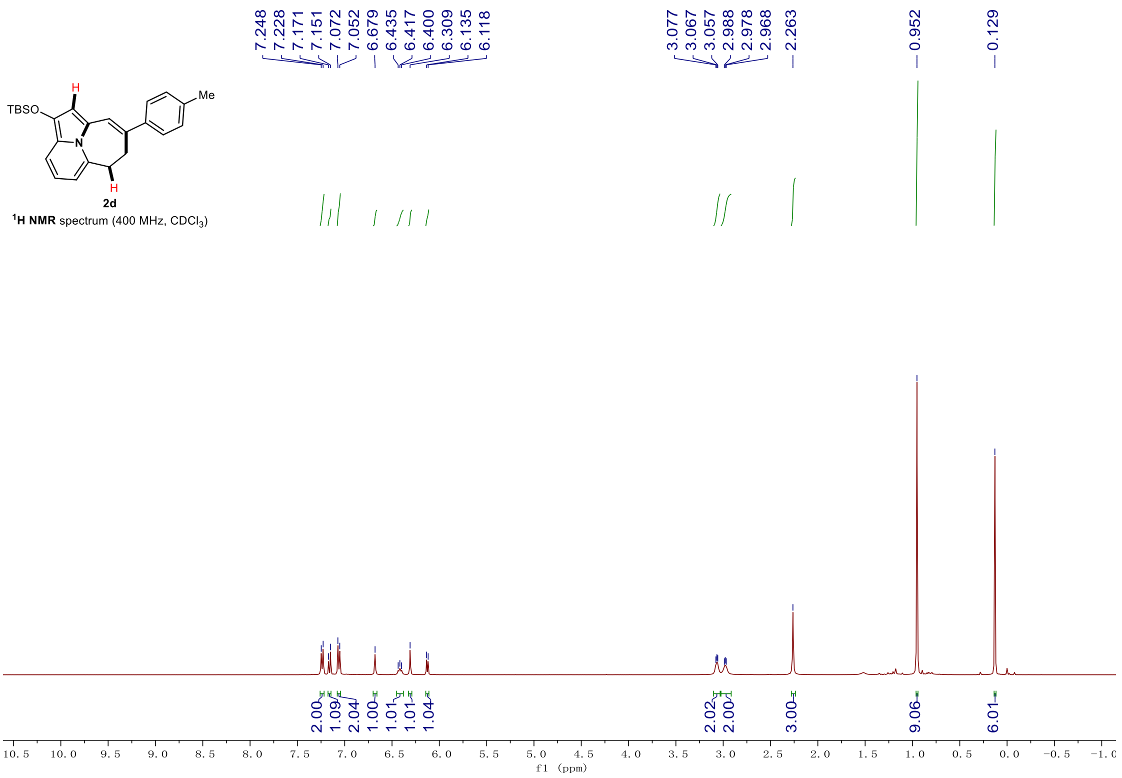

Supplementary Figure 99. ^{1}H NMR (400 MHz, $CDCl_3$) spectra for **2d**

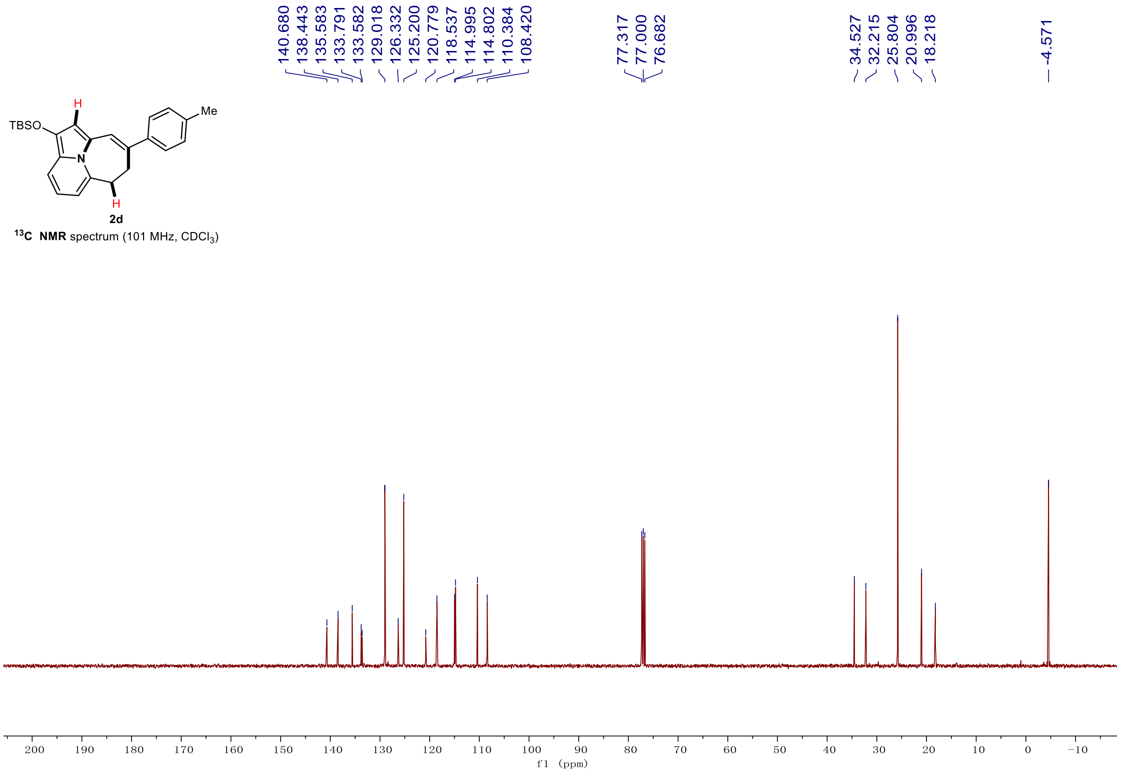

Supplementary Figure 100. ^{13}C NMR (101 MHz, $CDCl_3$) spectra for **2d**

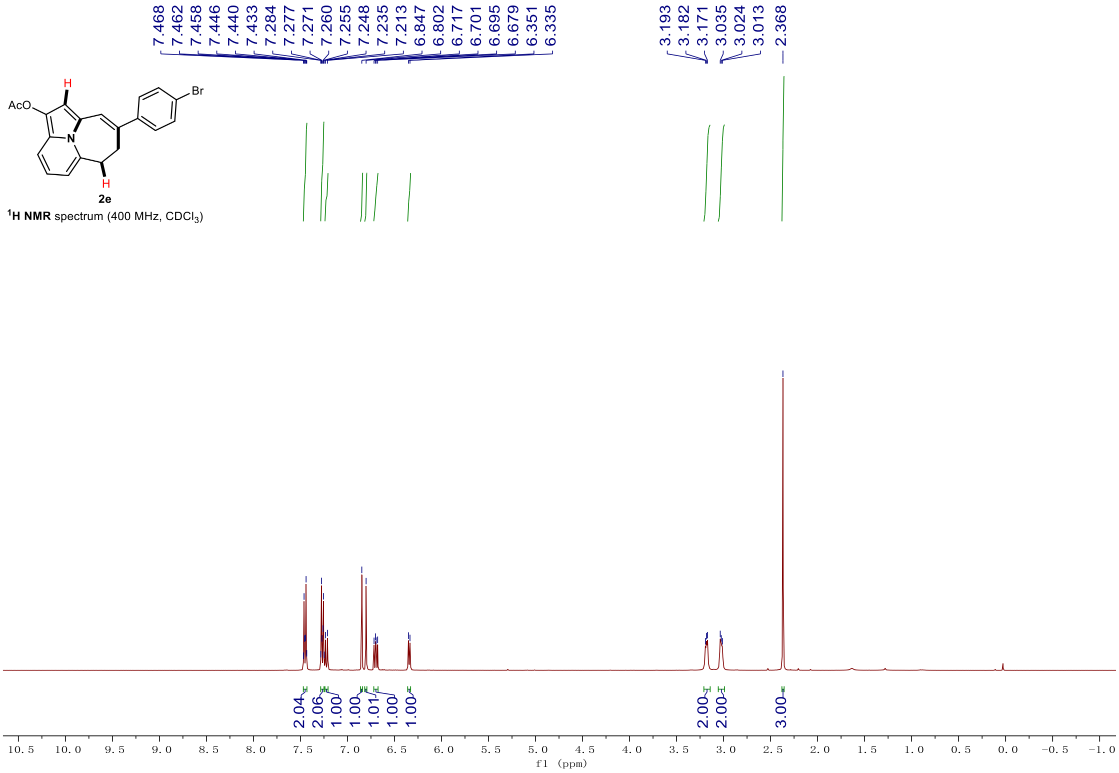

Supplementary Figure 101. ^{1}H NMR (400 MHz, $CDCl_3$) spectra for **2e**

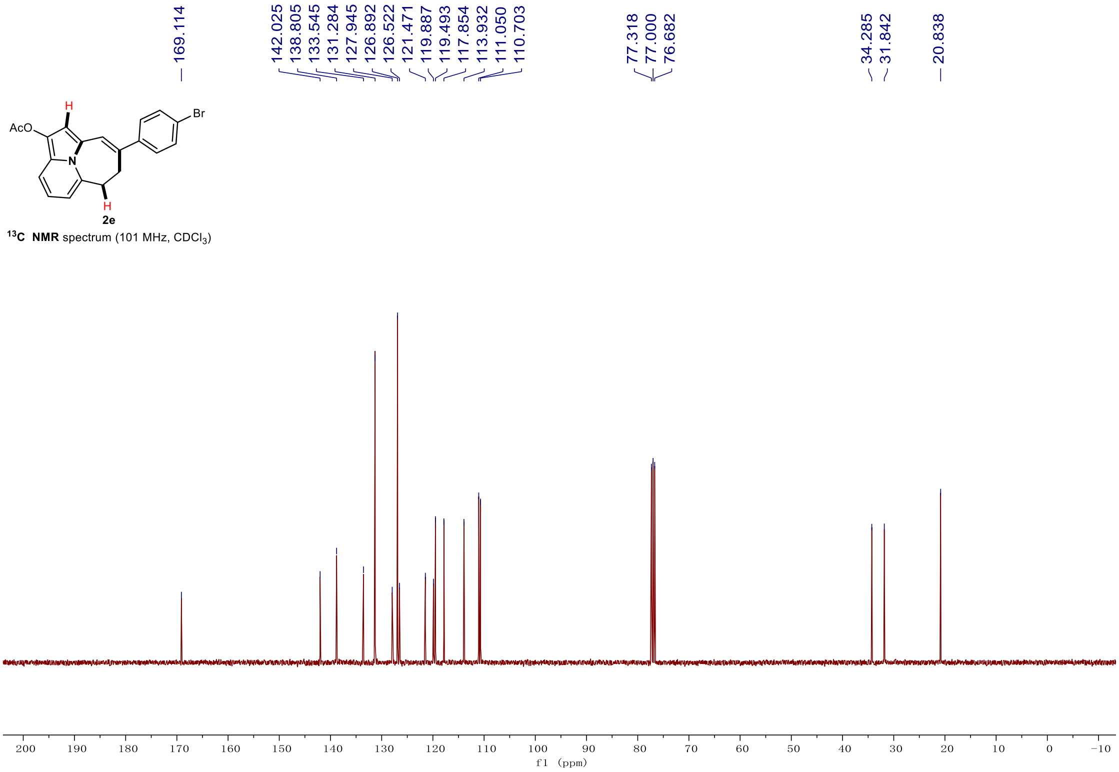

Supplementary Figure 102. ^{13}C NMR (101 MHz, $CDCl_3$) spectra for **2e**

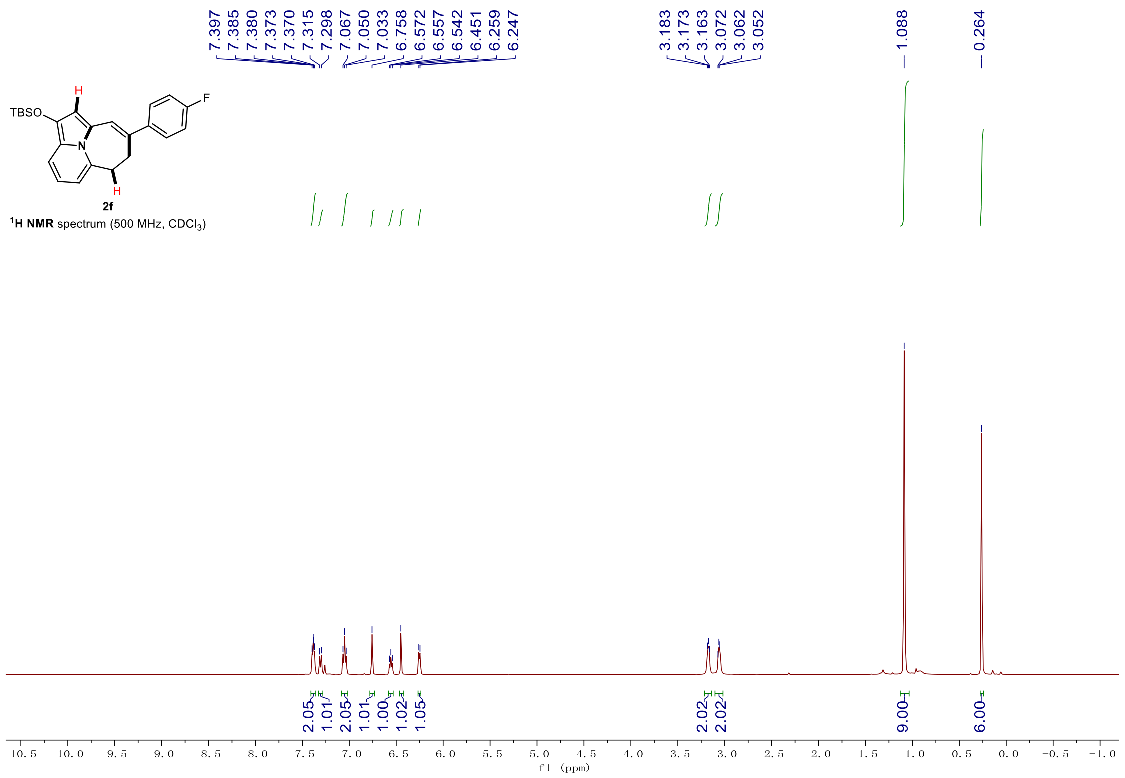

Supplementary Figure 103. ^{1}H NMR (500 MHz, $CDCl_3$) spectra for **2f**

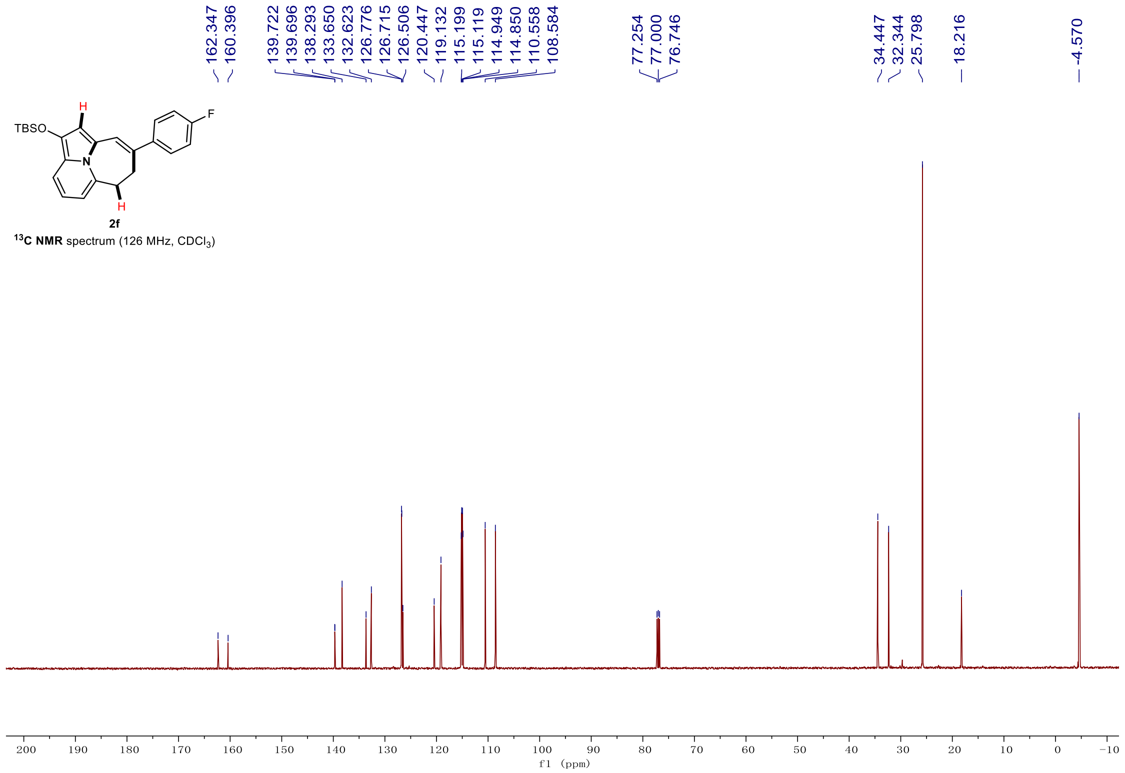

Supplementary Figure 104. ^{13}C NMR (126 MHz, $CDCl_3$) spectra for **2f**

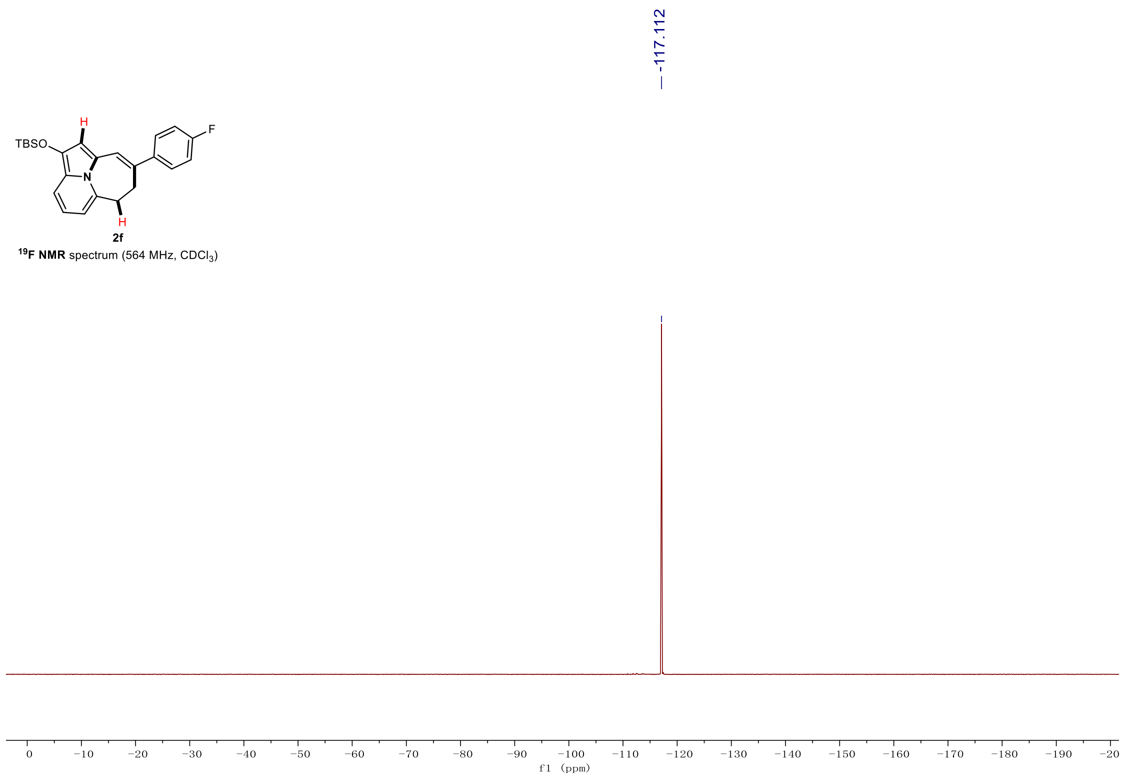

Supplementary Figure 105. ^{19}F NMR (564 MHz, $CDCl_3$) spectra for **2f**

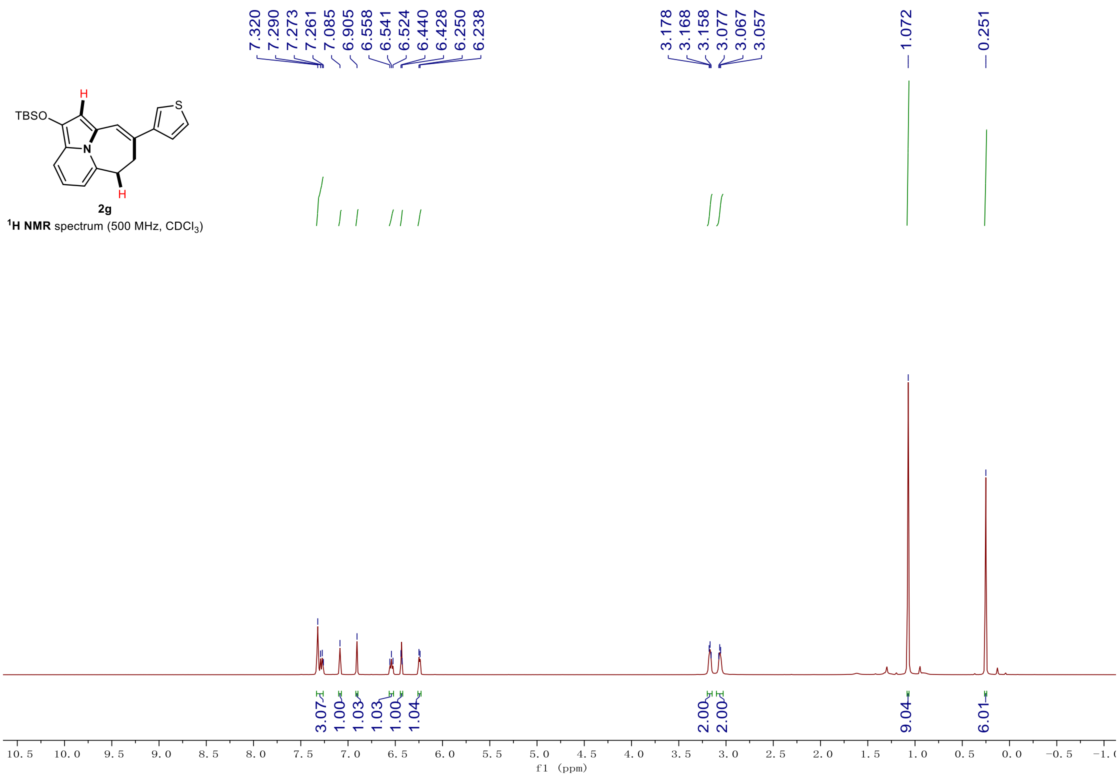

Supplementary Figure 106. ^{1}H NMR (500 MHz, $CDCl_3$) spectra for **2g**

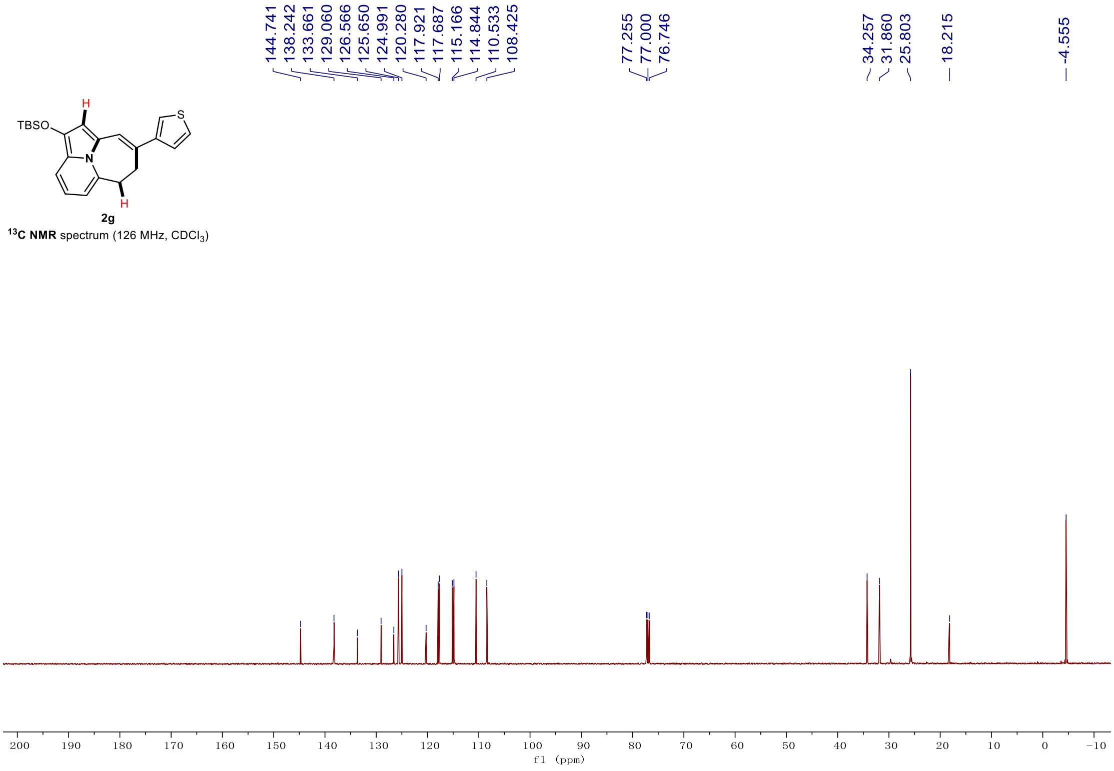

Supplementary Figure 107. ^{13}C NMR (126 MHz, $CDCl_3$) spectra for **2g**

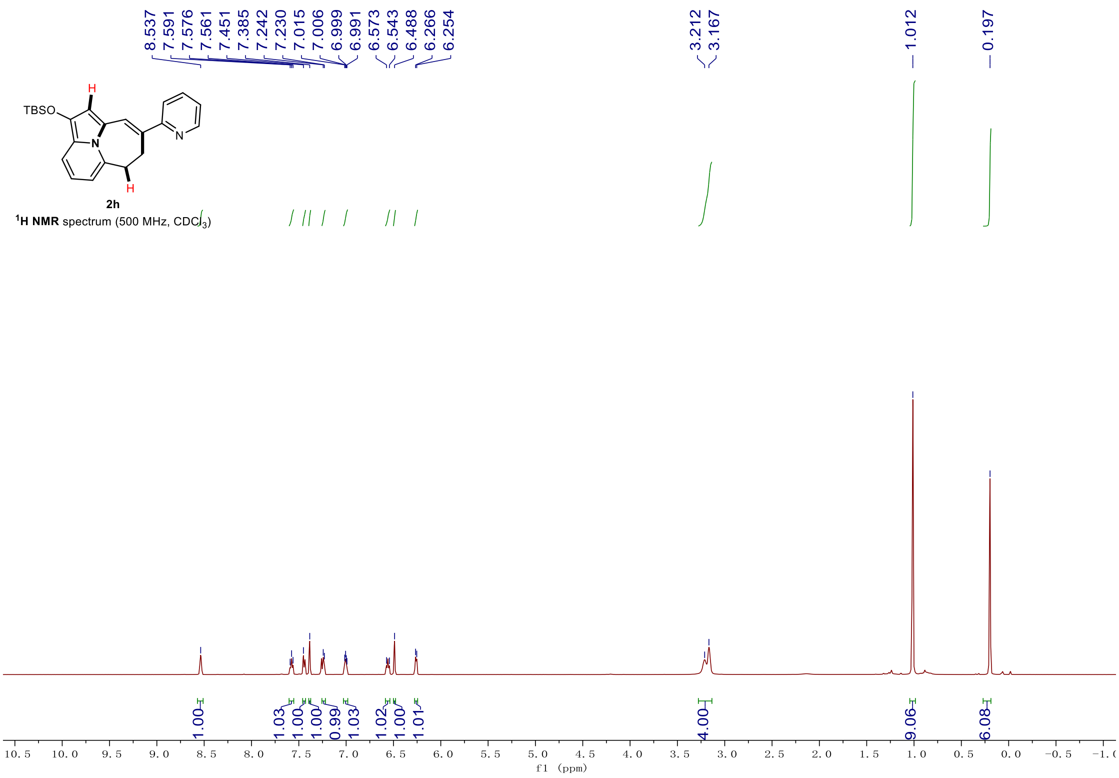

Supplementary Figure 108. ^{1}H NMR (500 MHz, $CDCl_3$) spectra for **2h**

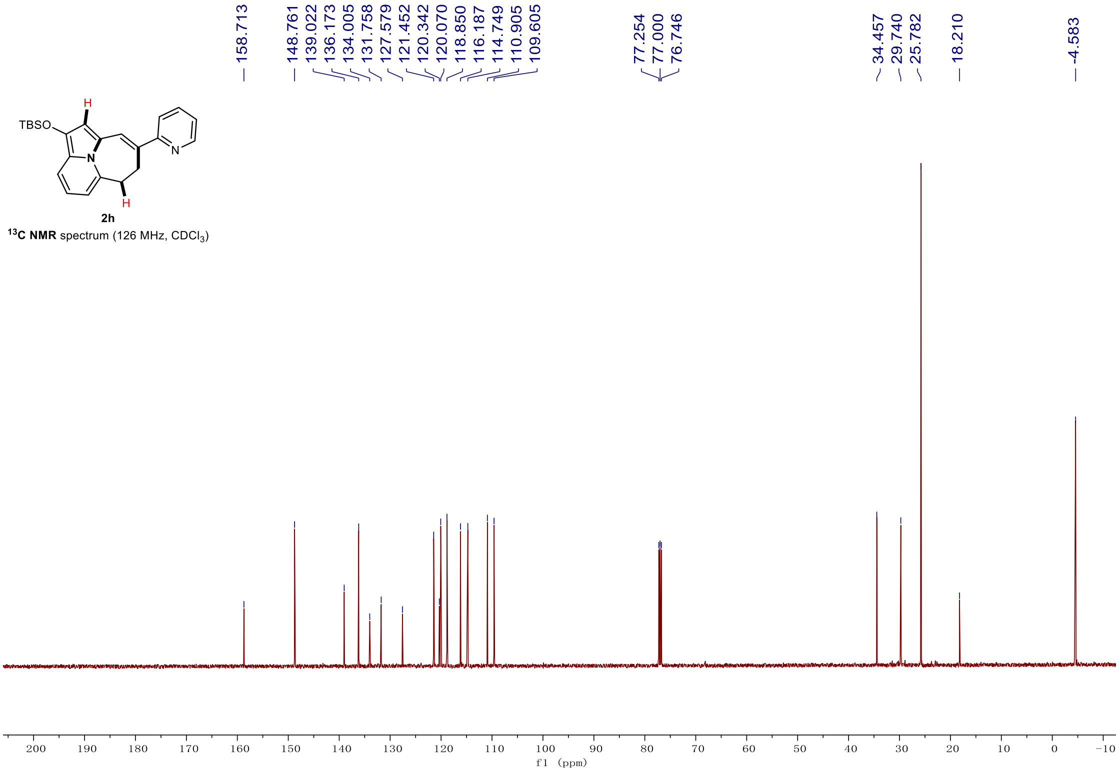

Supplementary Figure 109. ^{13}C NMR (126 MHz, $CDCl_3$) spectra for **2h**

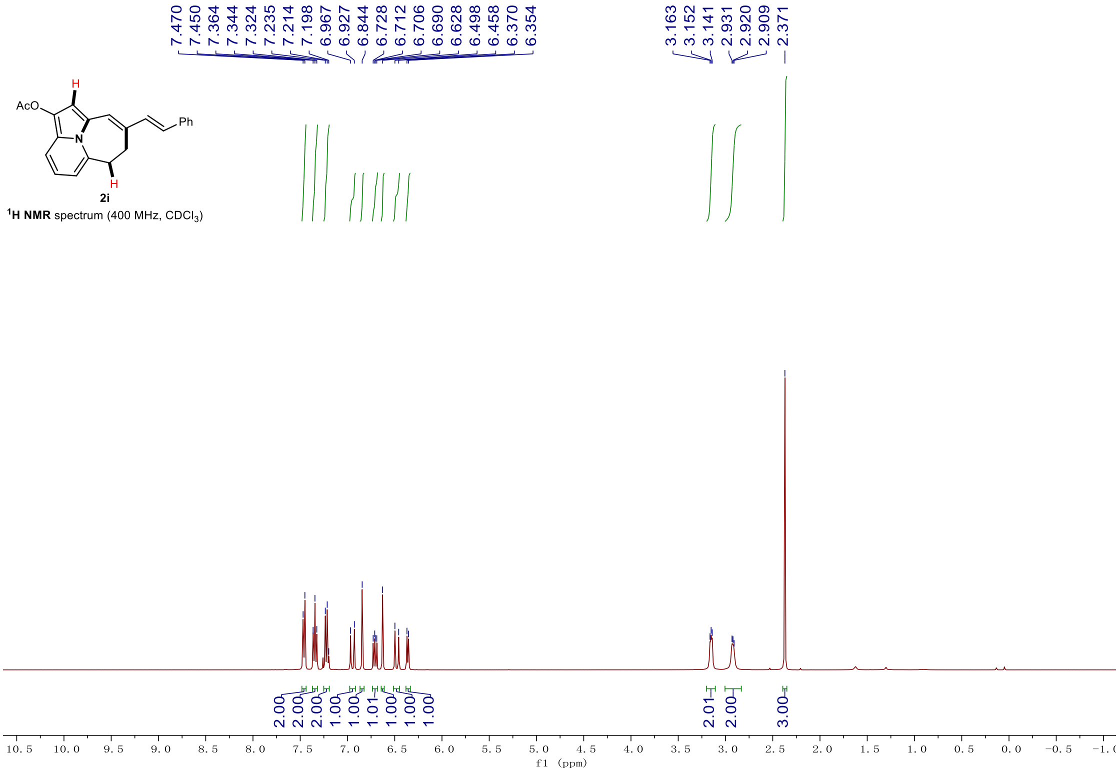

Supplementary Figure 110. ^{1}H NMR (400 MHz, $CDCl_3$) spectra for **2i**

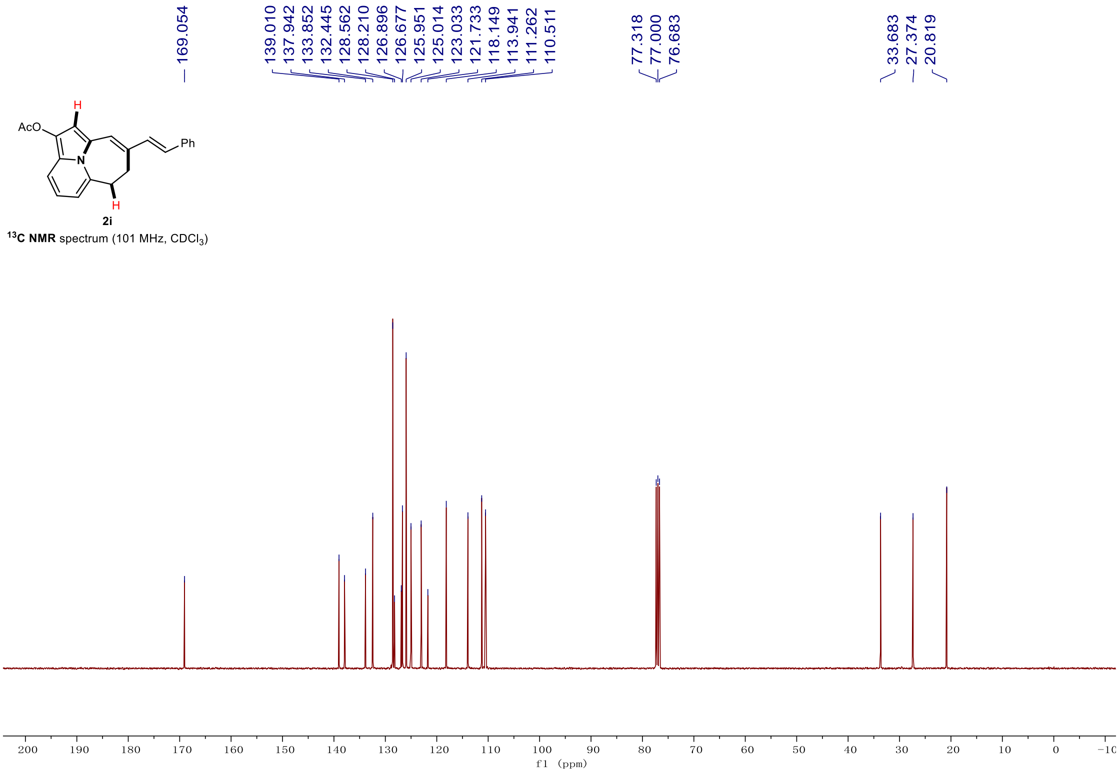

Supplementary Figure 111. ^{13}C NMR (101 MHz, $CDCl_3$) spectra for **2i**

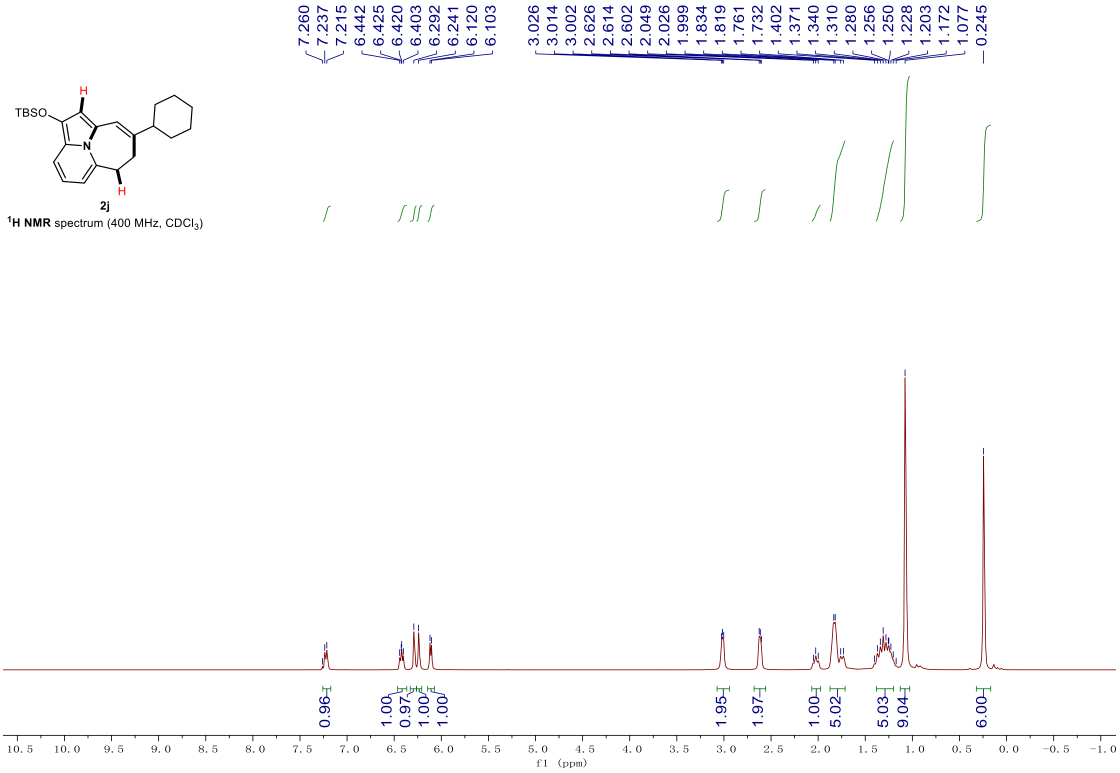

Supplementary Figure 112. ^{1}H NMR (400 MHz, $CDCl_3$) spectra for **2j**

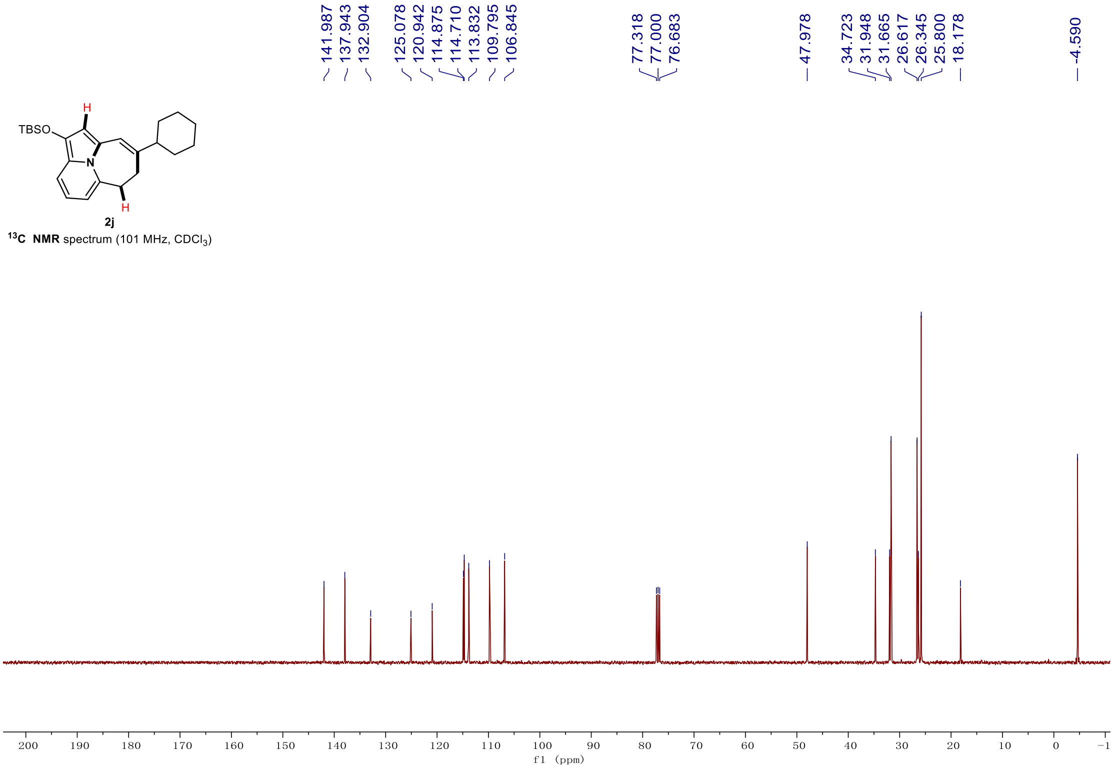

Supplementary Figure 113. ^{13}C NMR (101 MHz, $CDCl_3$) spectra for **2j**

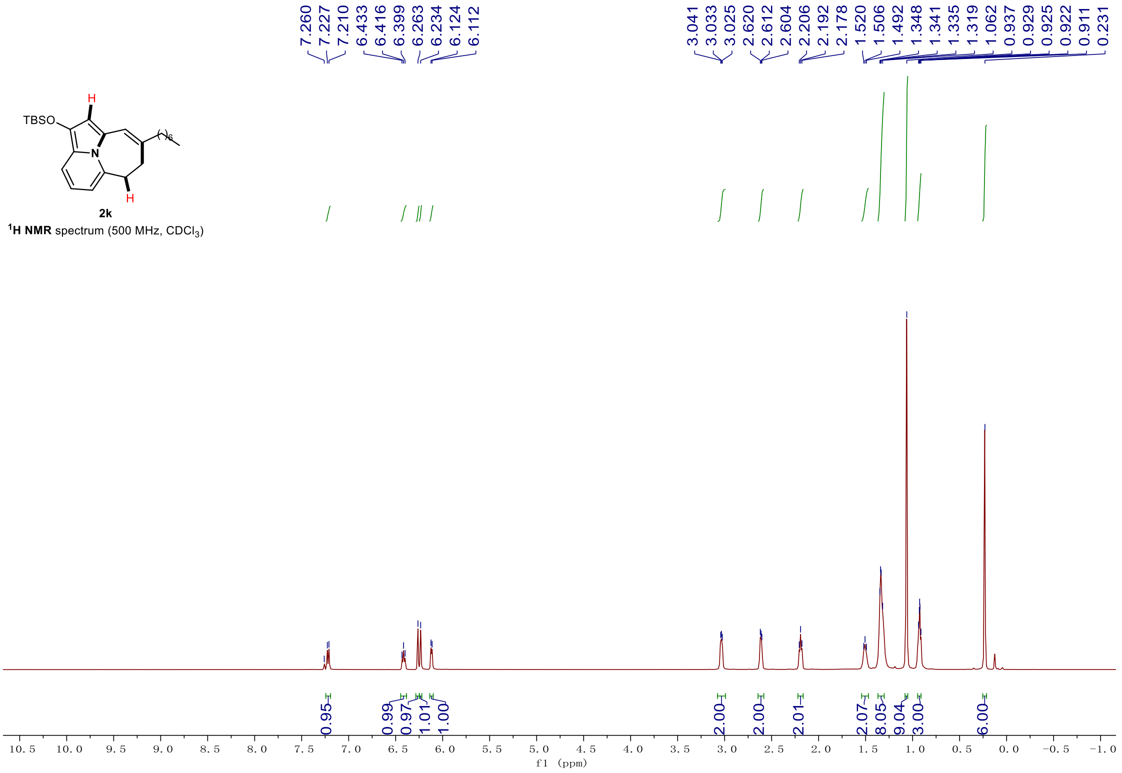

Supplementary Figure 114. ^{1}H NMR (500 MHz, $CDCl_3$) spectra for **2k**

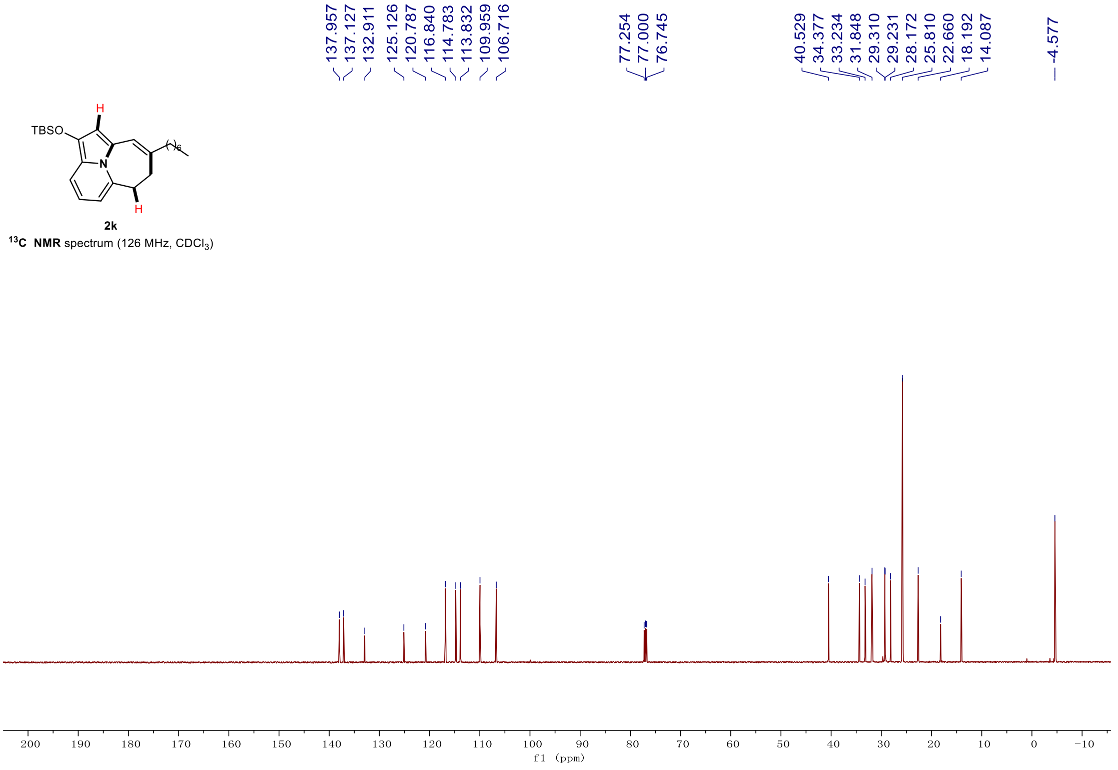

Supplementary Figure 115. ^{13}C NMR (126 MHz, $CDCl_3$) spectra for **2k**

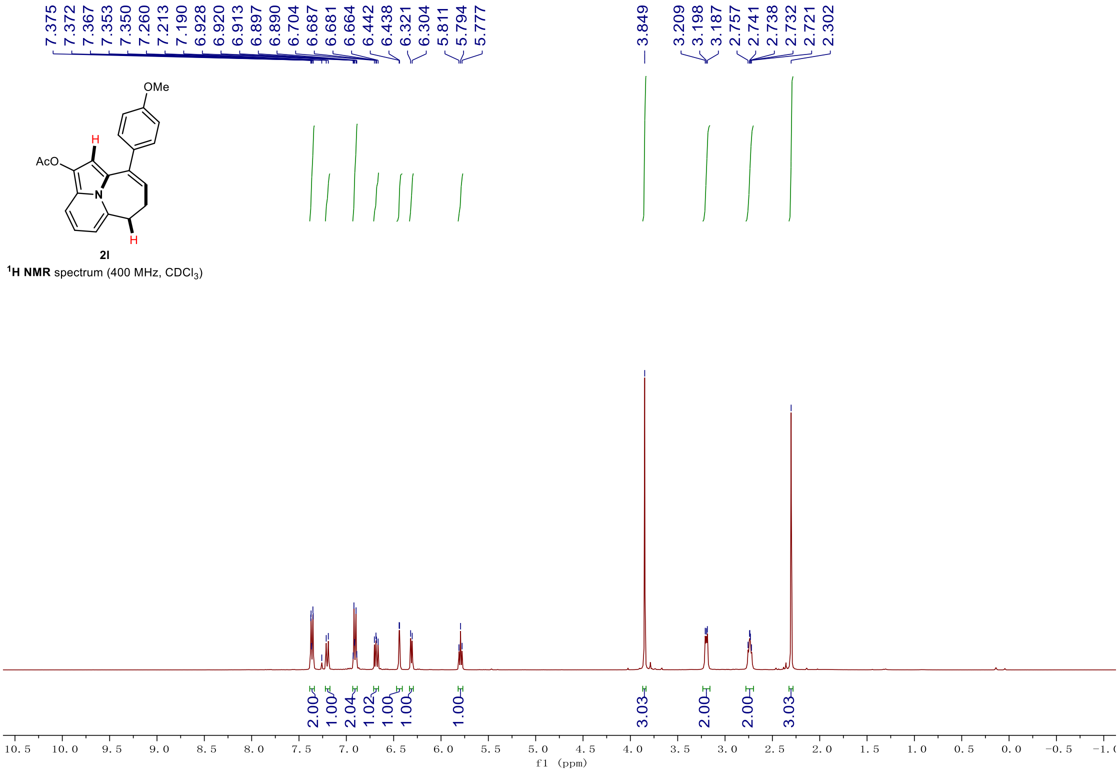

Supplementary Figure 116. ^{1}H NMR (400 MHz, CDCl$_3$) spectra for **2l**

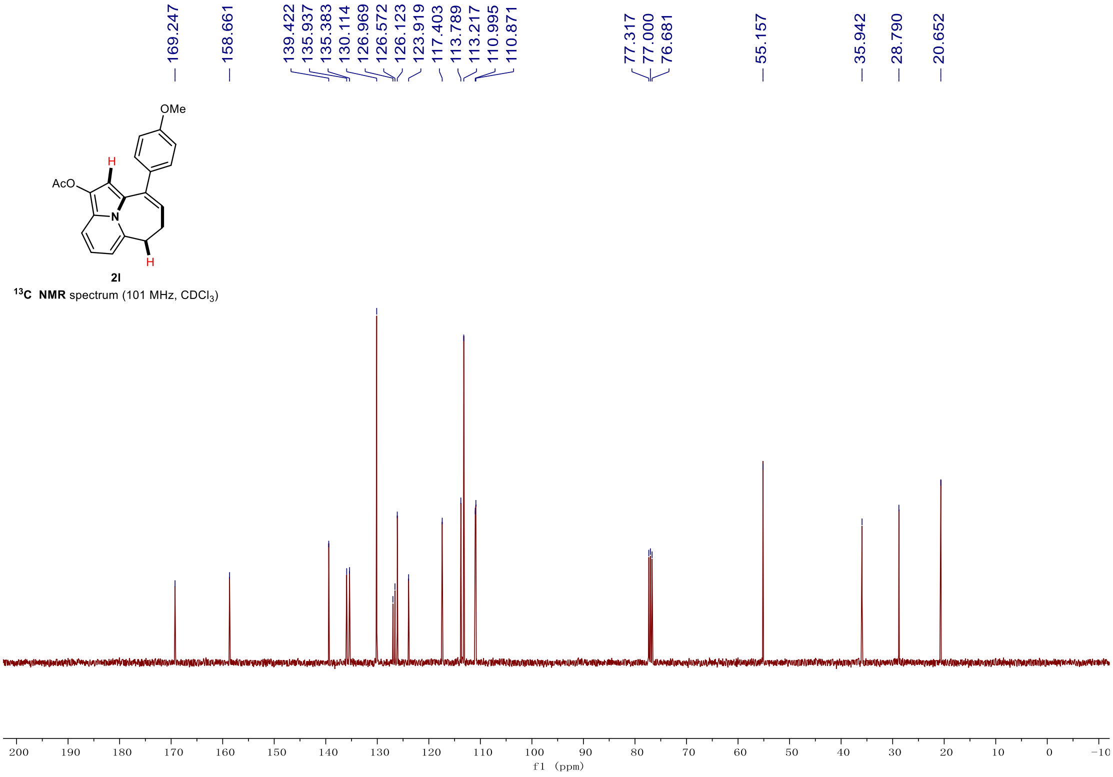

Supplementary Figure 117. ^{13}C NMR (101 MHz, CDCl$_3$) spectra for **2l**

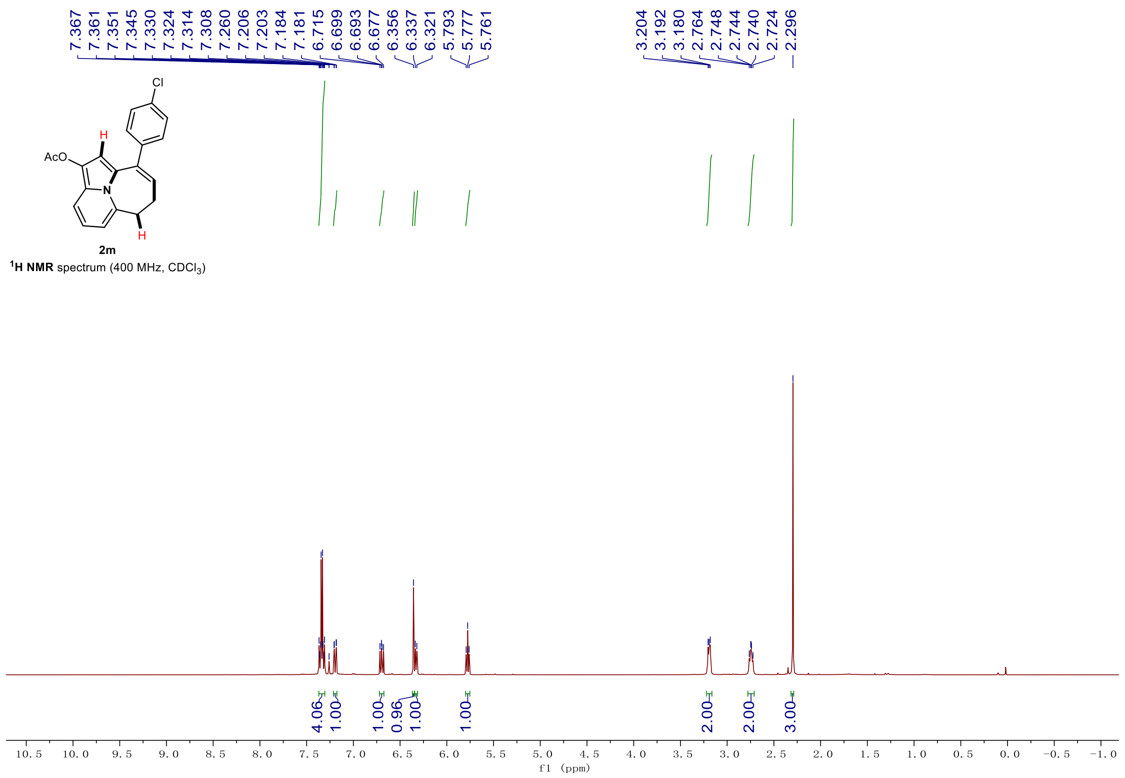

Supplementary Figure 118. ^{1}H NMR (400 MHz, $CDCl_3$) spectra for **2m**

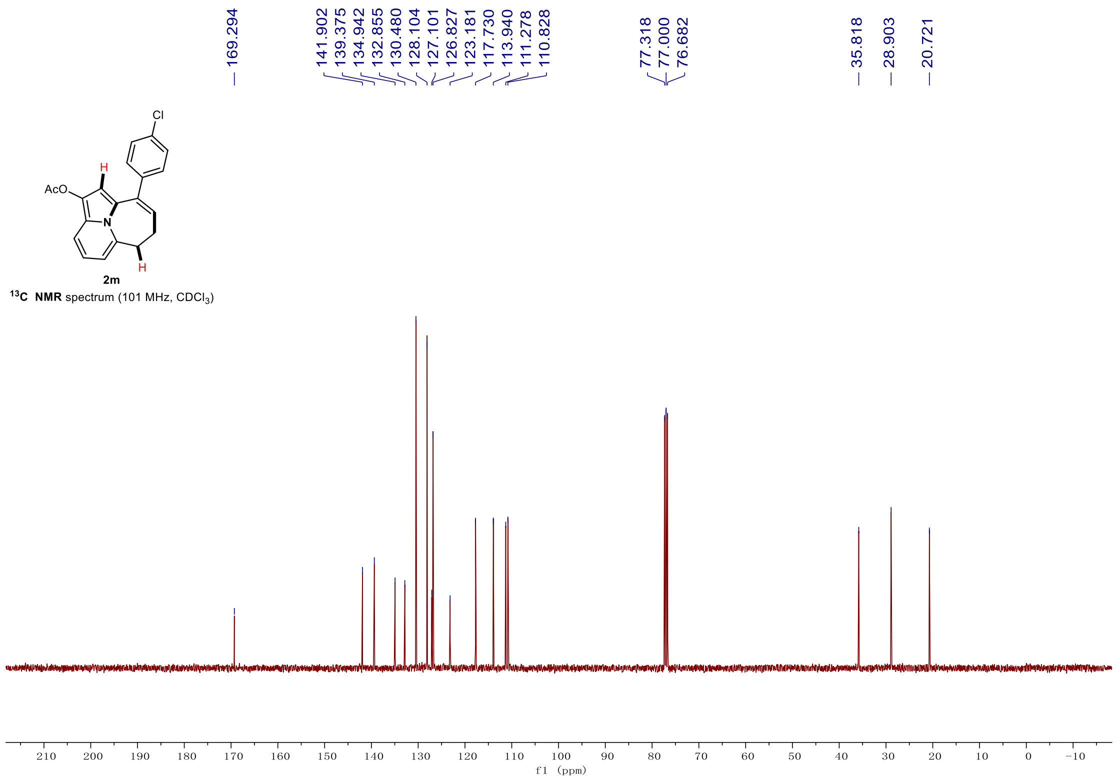

Supplementary Figure 119. ^{13}C NMR (101 MHz, $CDCl_3$) spectra for **2m**

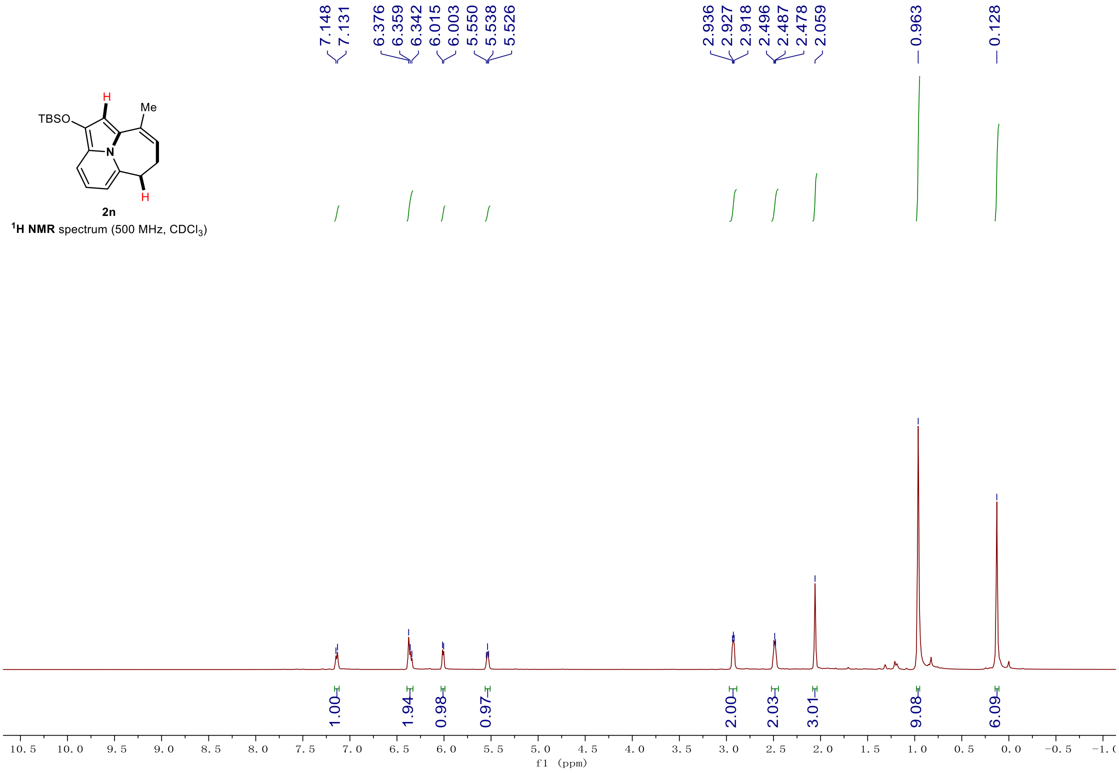

Supplementary Figure 120. ^{1}H NMR (500 MHz, $CDCl_3$) spectra for **2n**

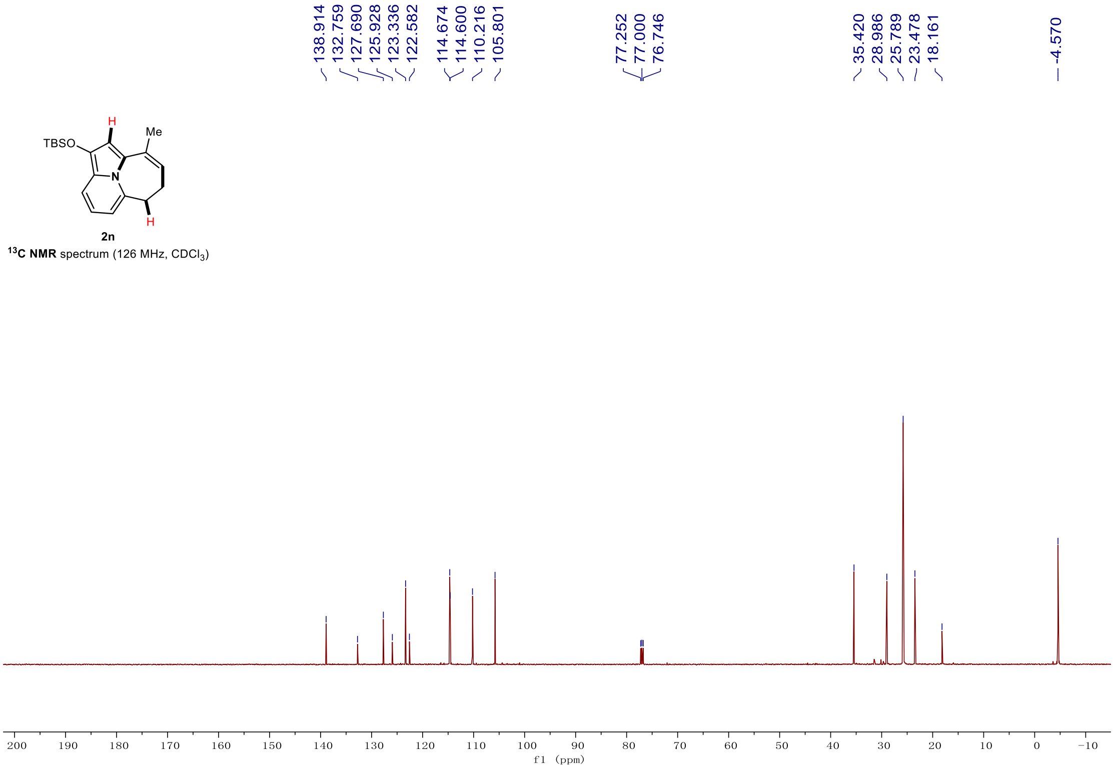

Supplementary Figure 121. ^{13}C NMR (126 MHz, $CDCl_3$) spectra for **2n**

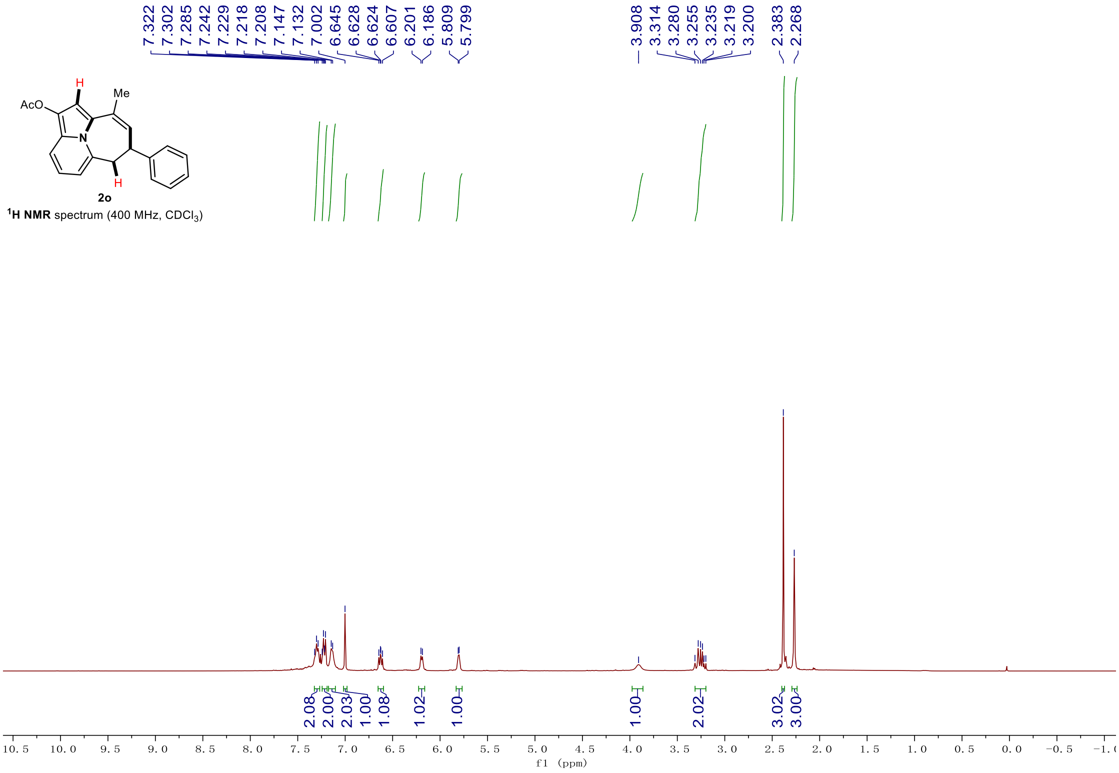

Supplementary Figure 122. ^{1}H NMR (400 MHz, $CDCl_3$) spectra for **2o**

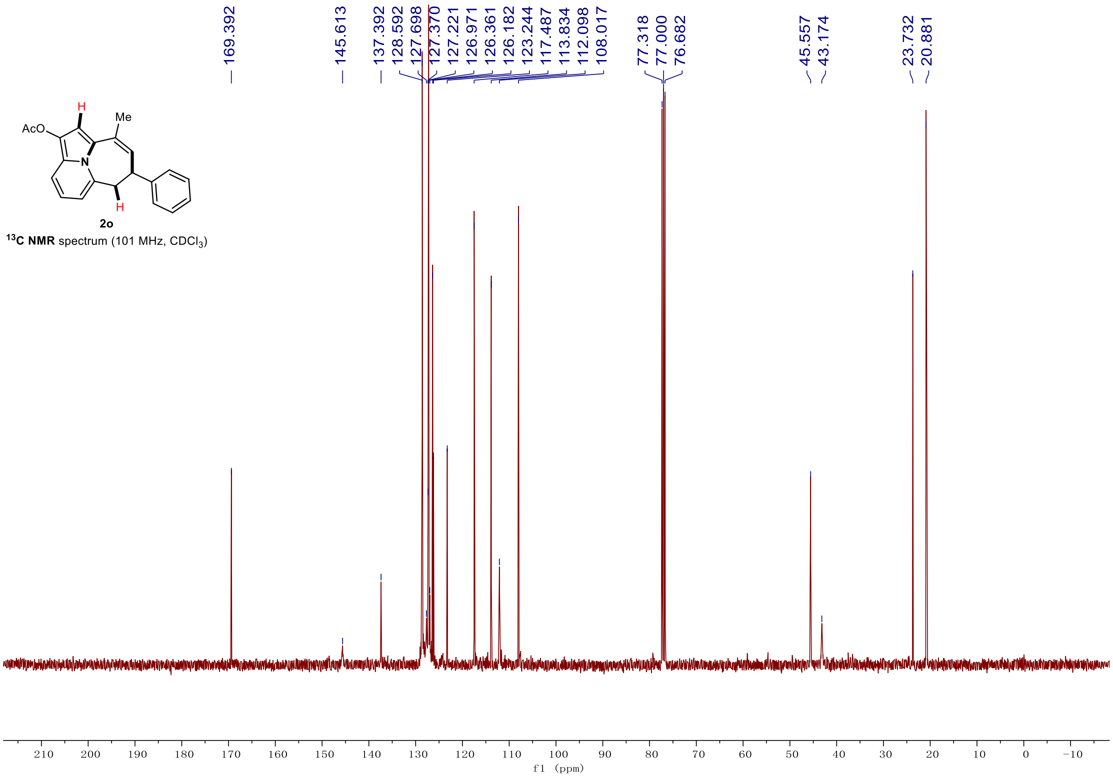

Supplementary Figure 123. ^{13}C NMR (101 MHz, $CDCl_3$) spectra for **2o**

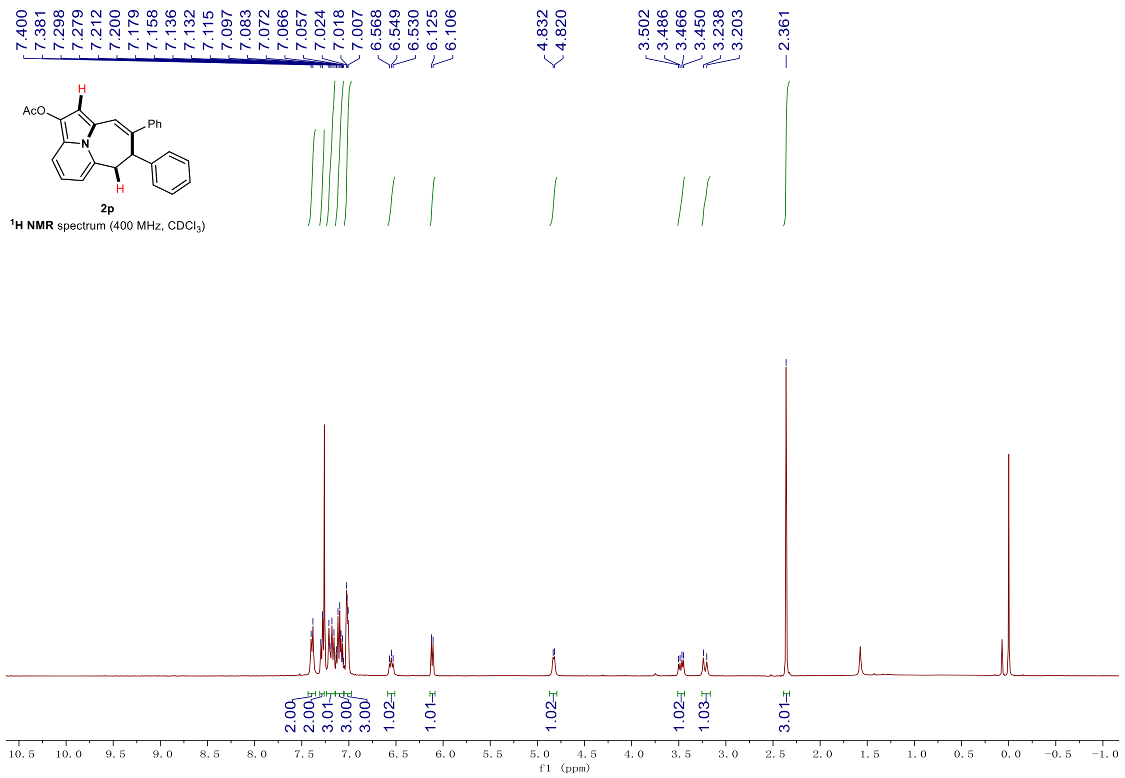

Supplementary Figure 124. ^{1}H NMR (400 MHz, $CDCl_3$) spectra for **2p**

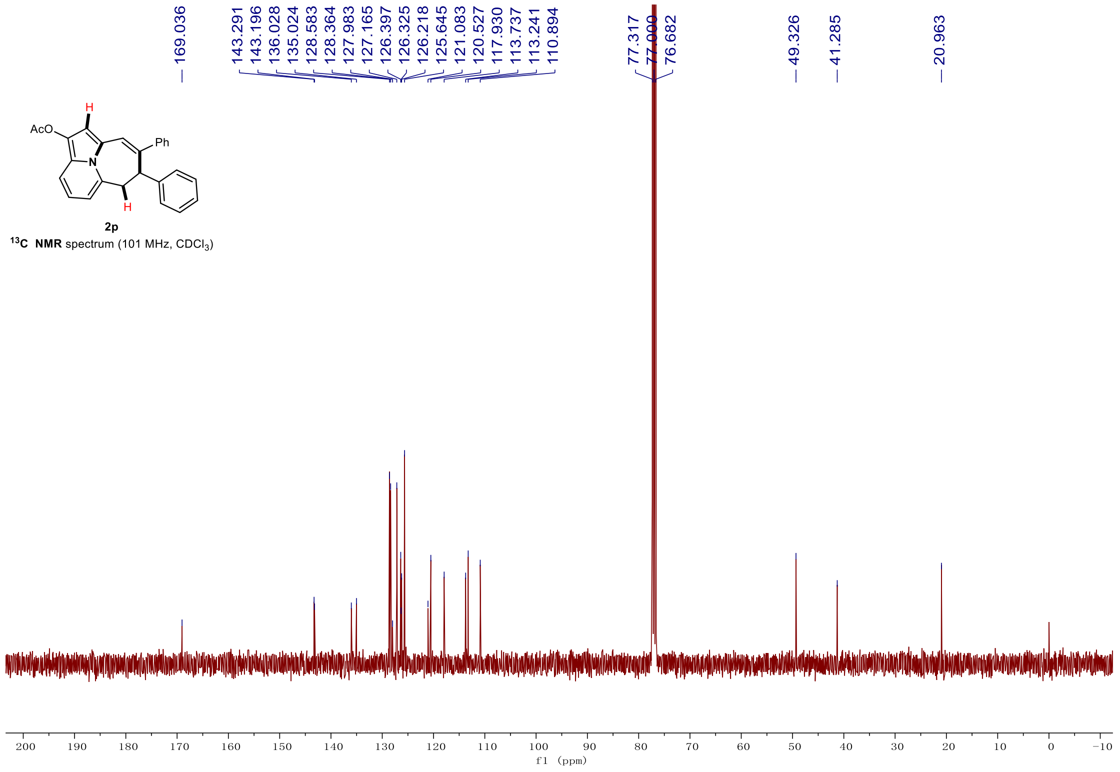

Supplementary Figure 125. ^{13}C NMR (101 MHz, $CDCl_3$) spectra for **2p**

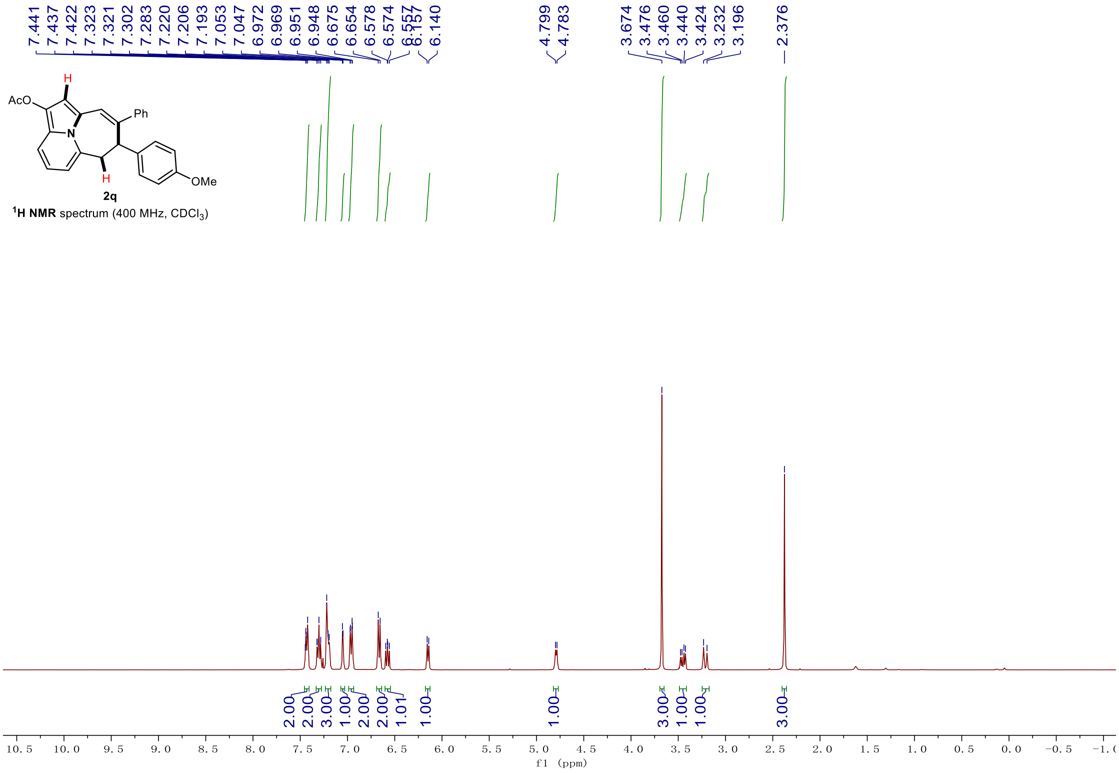

Supplementary Figure 126. ^{1}H NMR (400 MHz, $CDCl_3$) spectra for **2q**

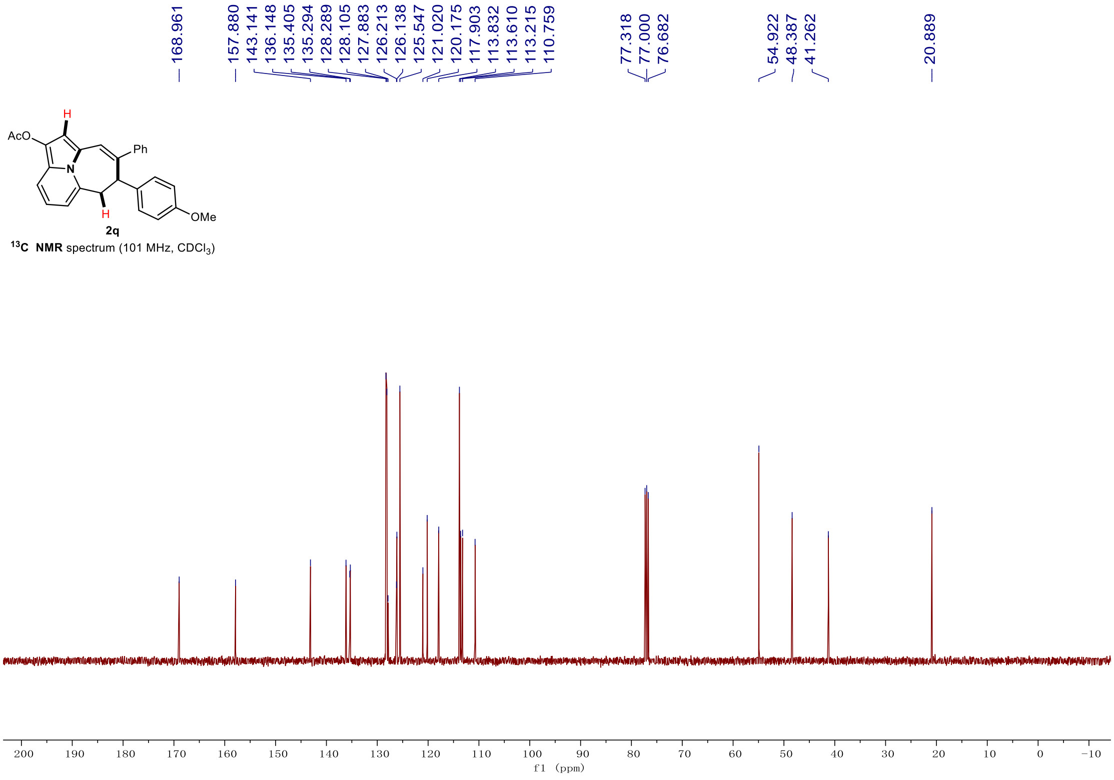

Supplementary Figure 127. ^{13}C NMR (101 MHz, $CDCl_3$) spectra for **2q**

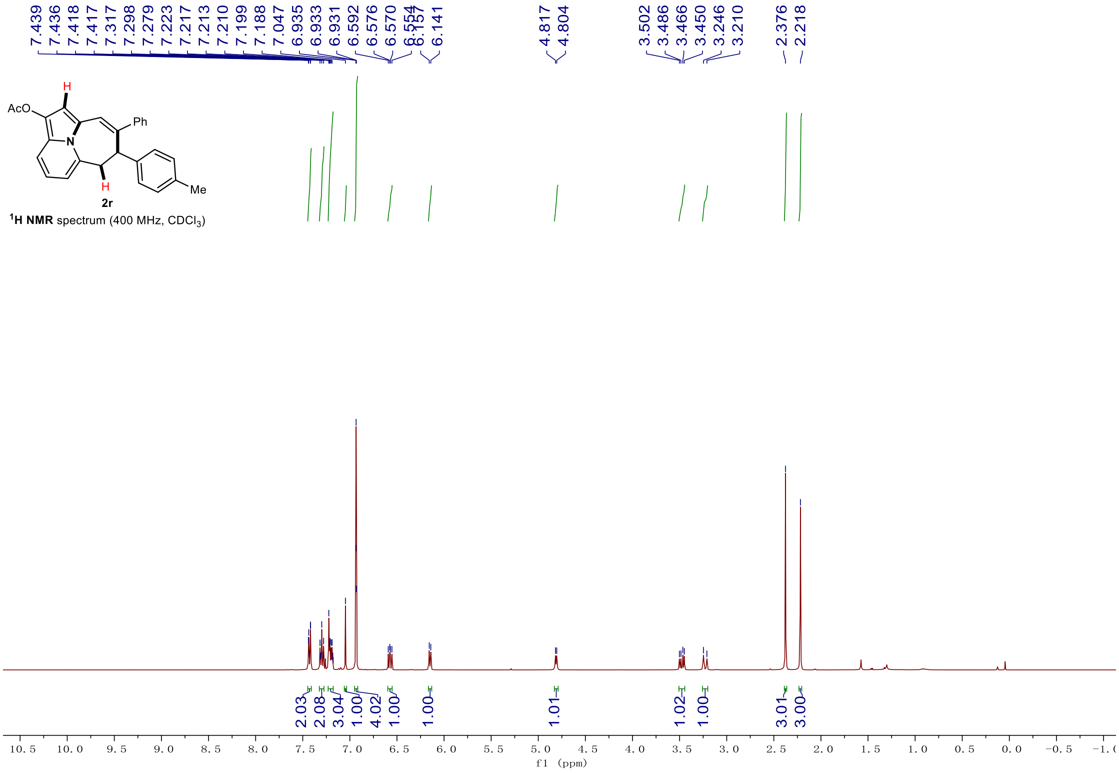

Supplementary Figure 128. ^{1}H NMR (400 MHz, $CDCl_3$) spectra for **2r**

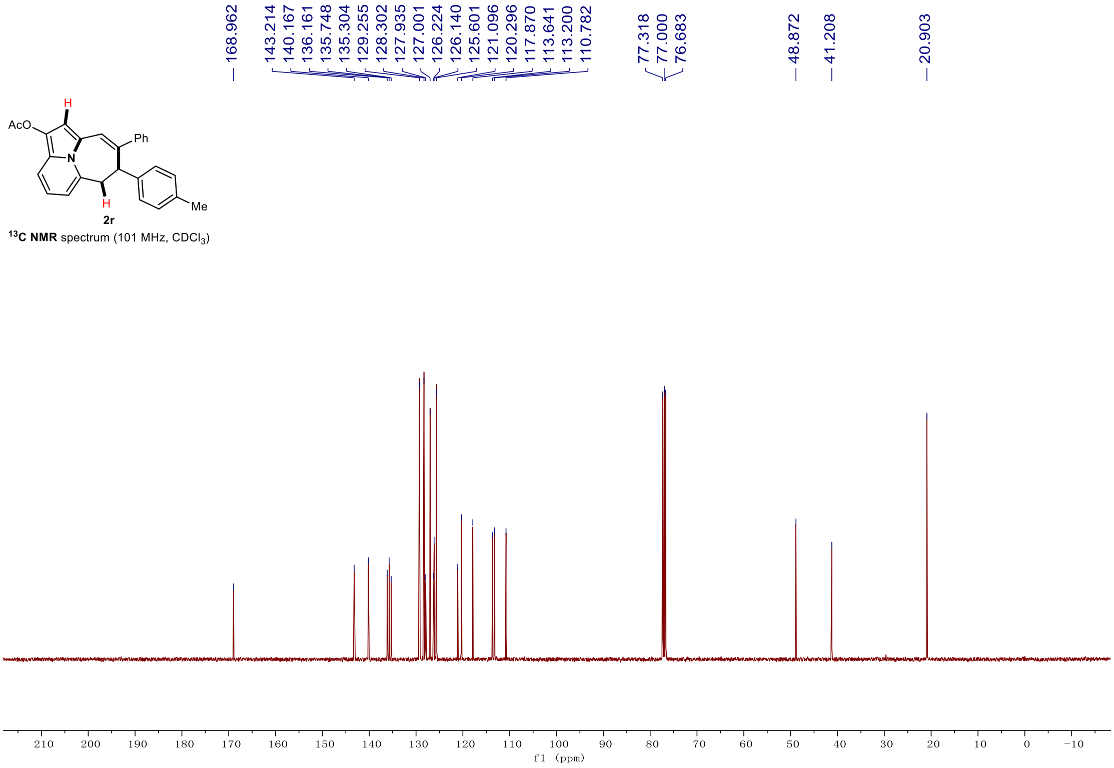

Supplementary Figure 129. ^{13}C NMR (101 MHz, $CDCl_3$) spectra for **2r**

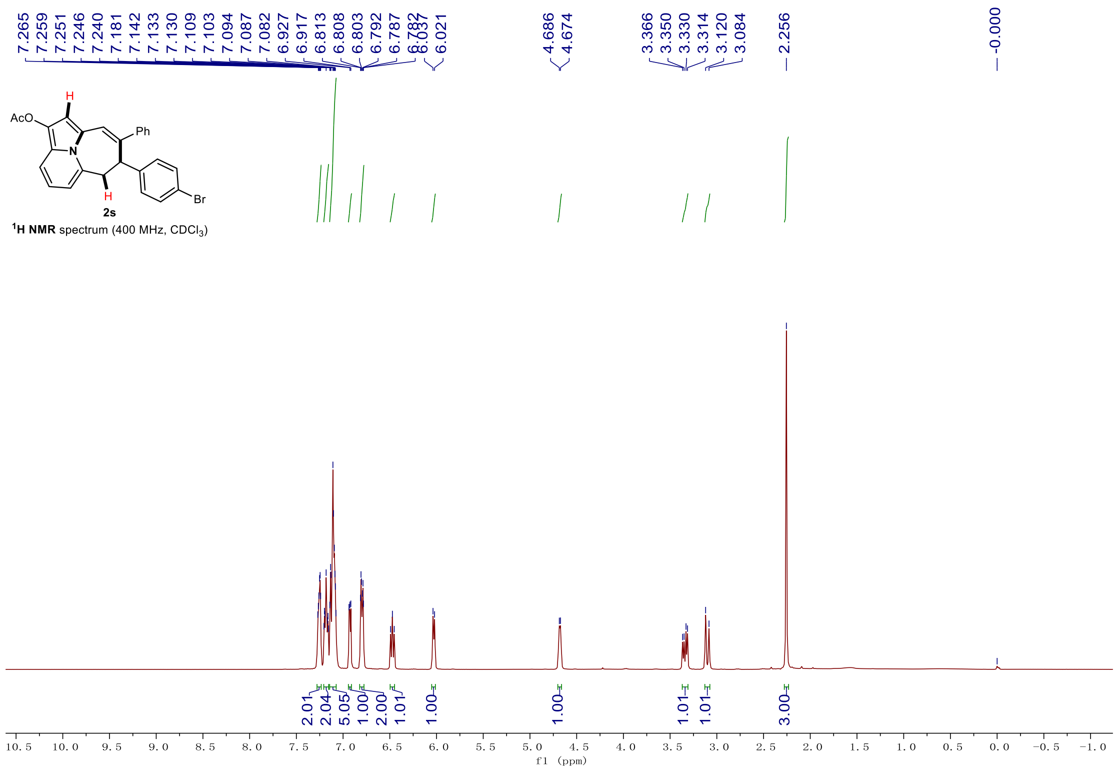

Supplementary Figure 130. ^{1}H NMR (400 MHz, $CDCl_3$) spectra for **2s**

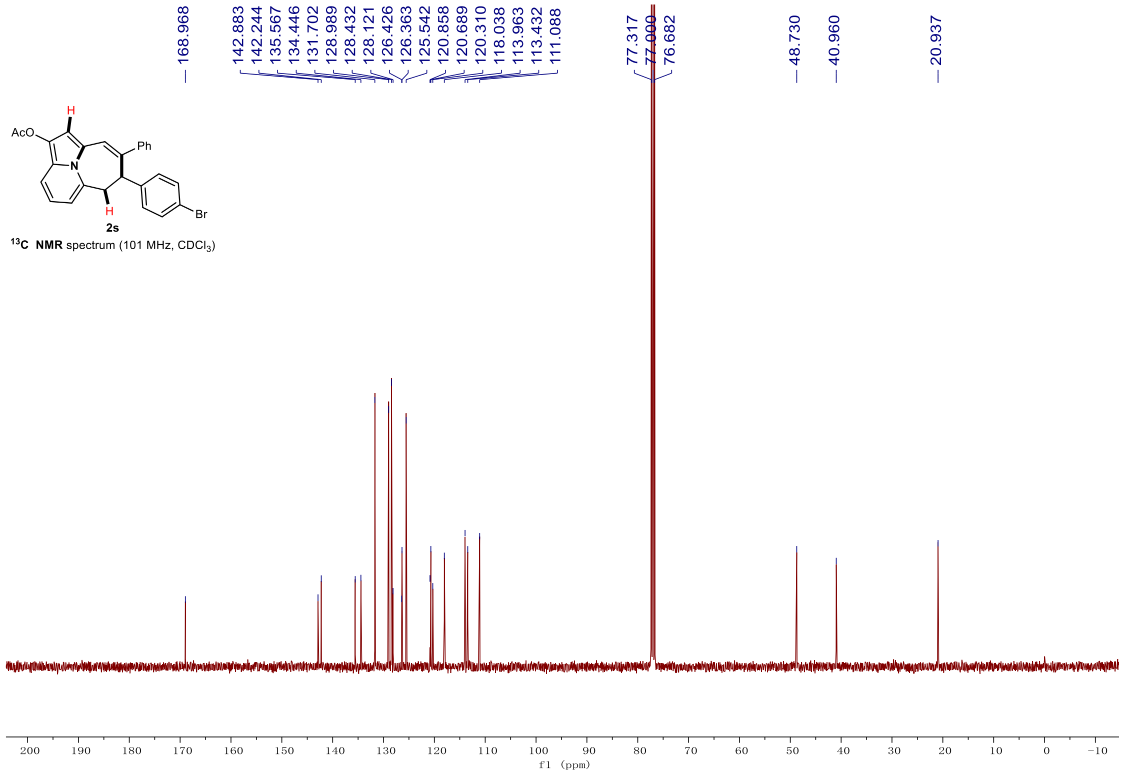

Supplementary Figure 131. ^{13}C NMR (101 MHz, $CDCl_3$) spectra for **2s**

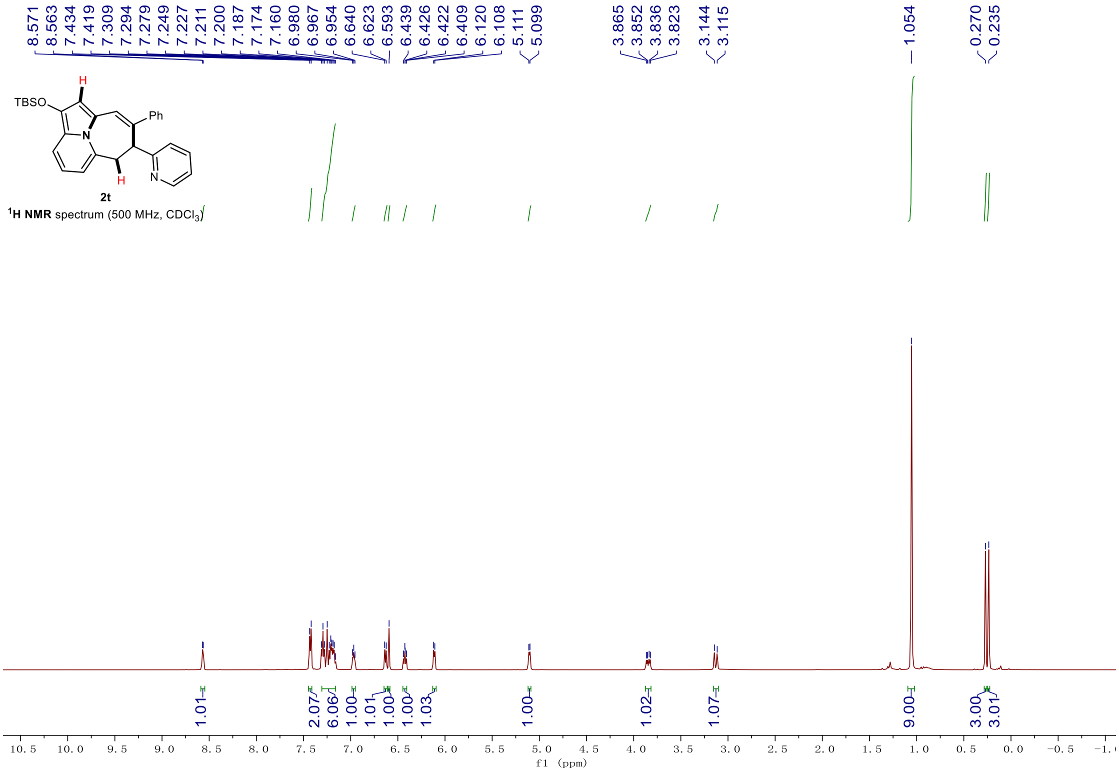

Supplementary Figure 132. ^{1}H NMR (500 MHz, $CDCl_3$) spectra for **2t**

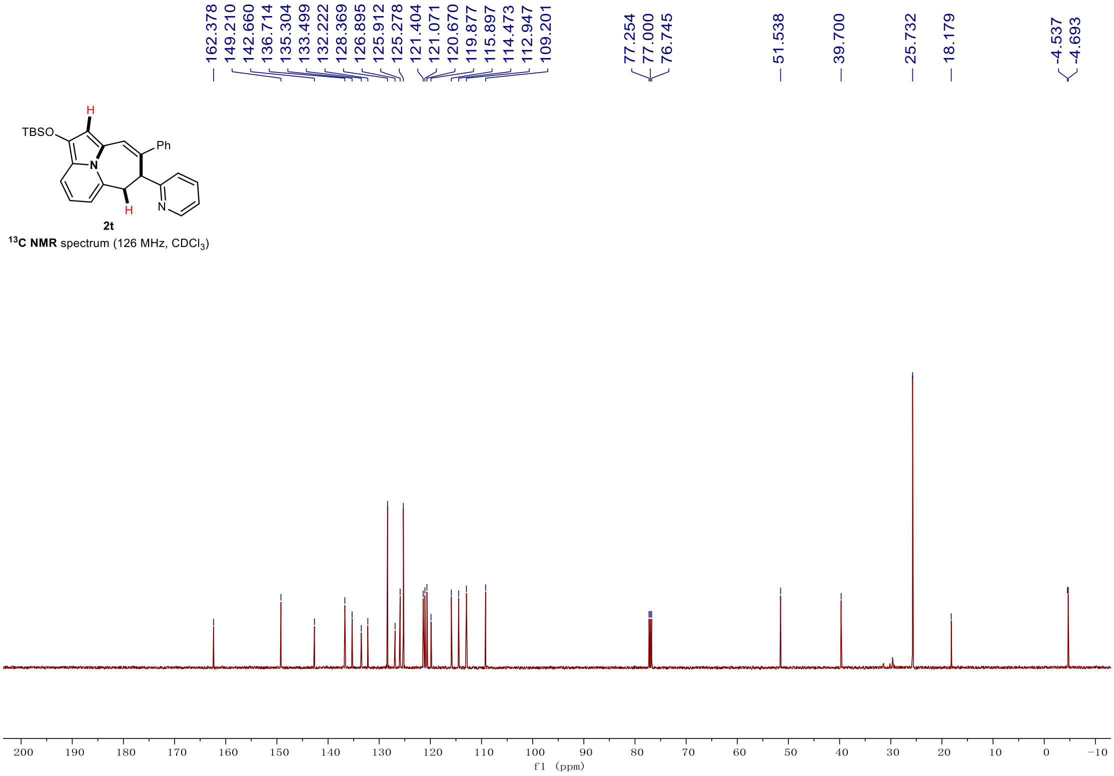

Supplementary Figure 133. ^{13}C NMR (126 MHz, $CDCl_3$) spectra for **2t**

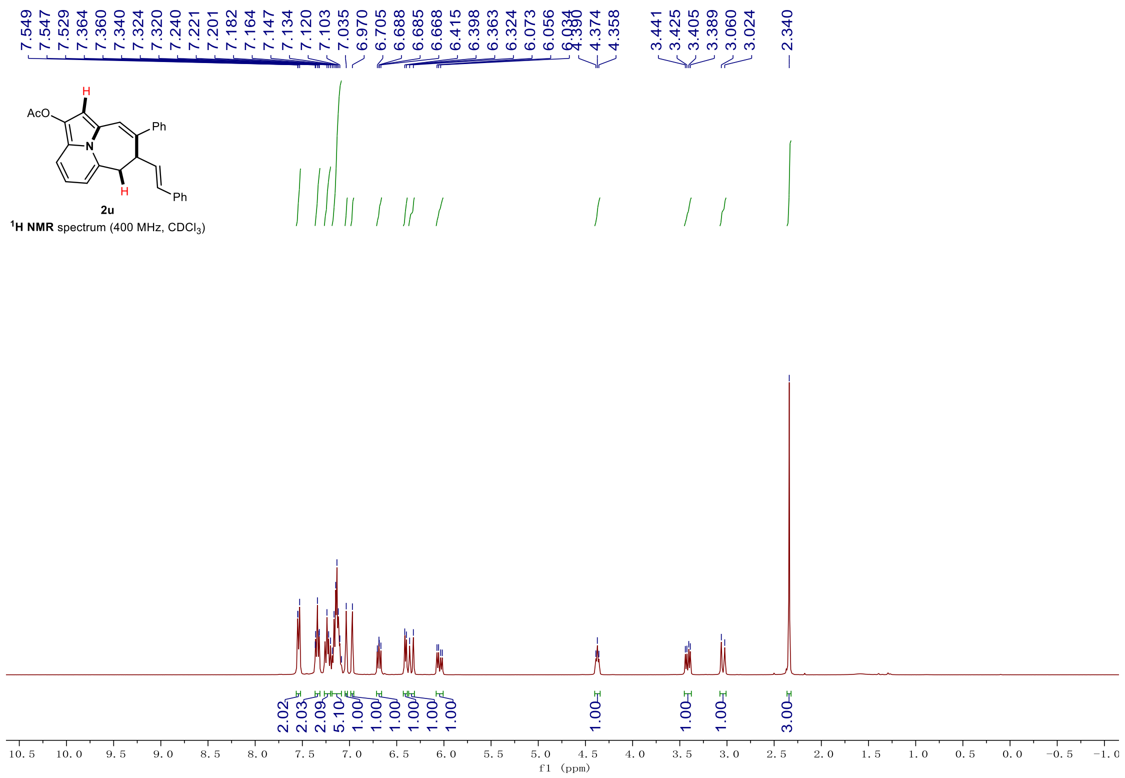

Supplementary Figure 134. ^{1}H NMR (400 MHz, $CDCl_3$) spectra for **2u**

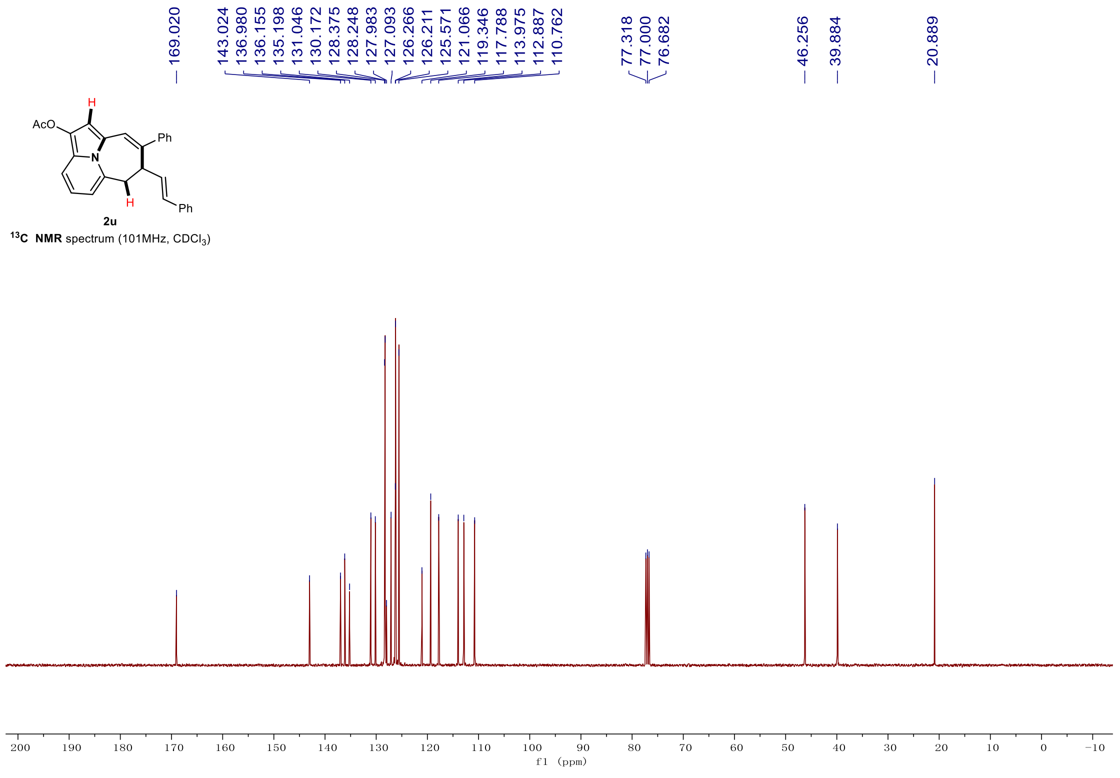

Supplementary Figure 135. ^{13}C NMR (101 MHz, $CDCl_3$) spectra for **2u**

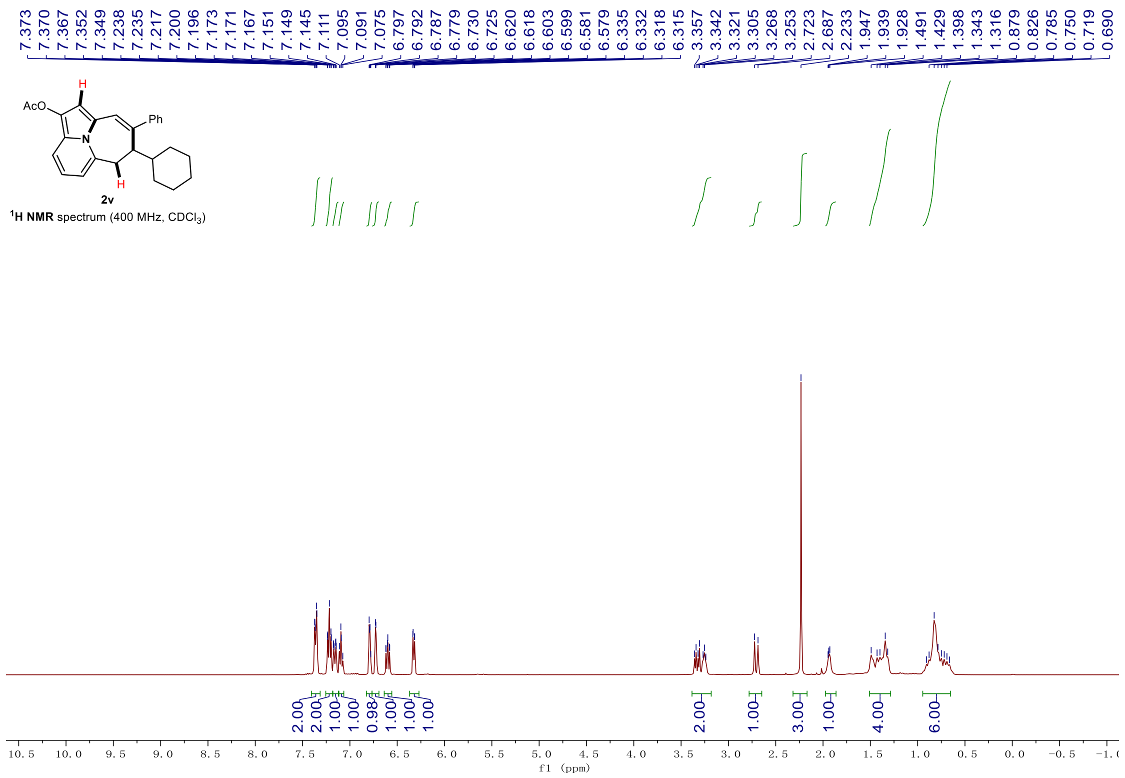

Supplementary Figure 136. ^{1}H NMR (400 MHz, $CDCl_3$) spectra for **2v**

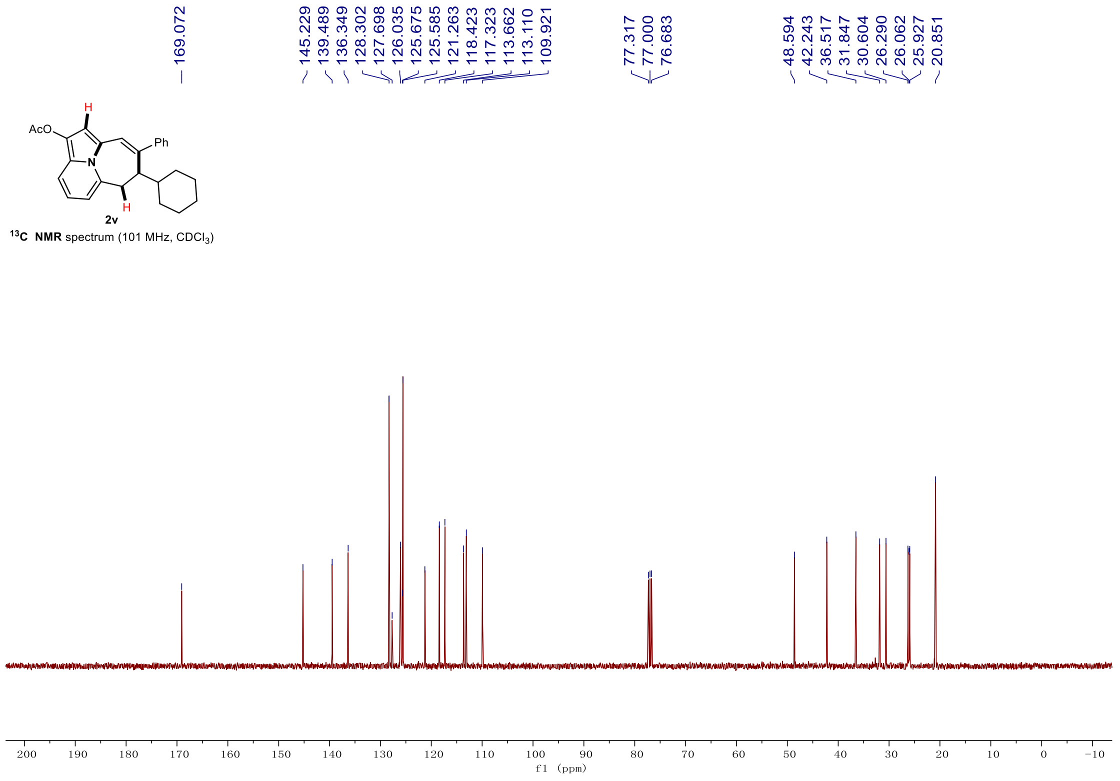

Supplementary Figure 137. ^{13}C NMR (101 MHz, $CDCl_3$) spectra for **2v**

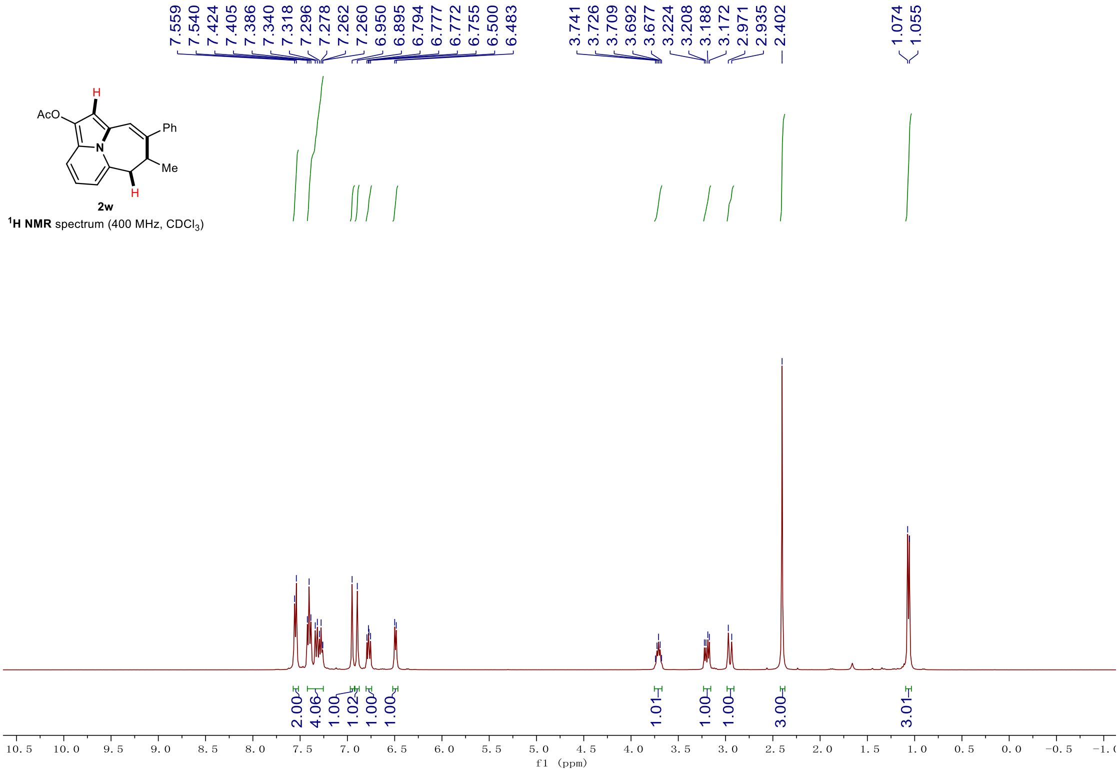

Supplementary Figure 138. ^{1}H NMR (400 MHz, $CDCl_3$) spectra for **2w**

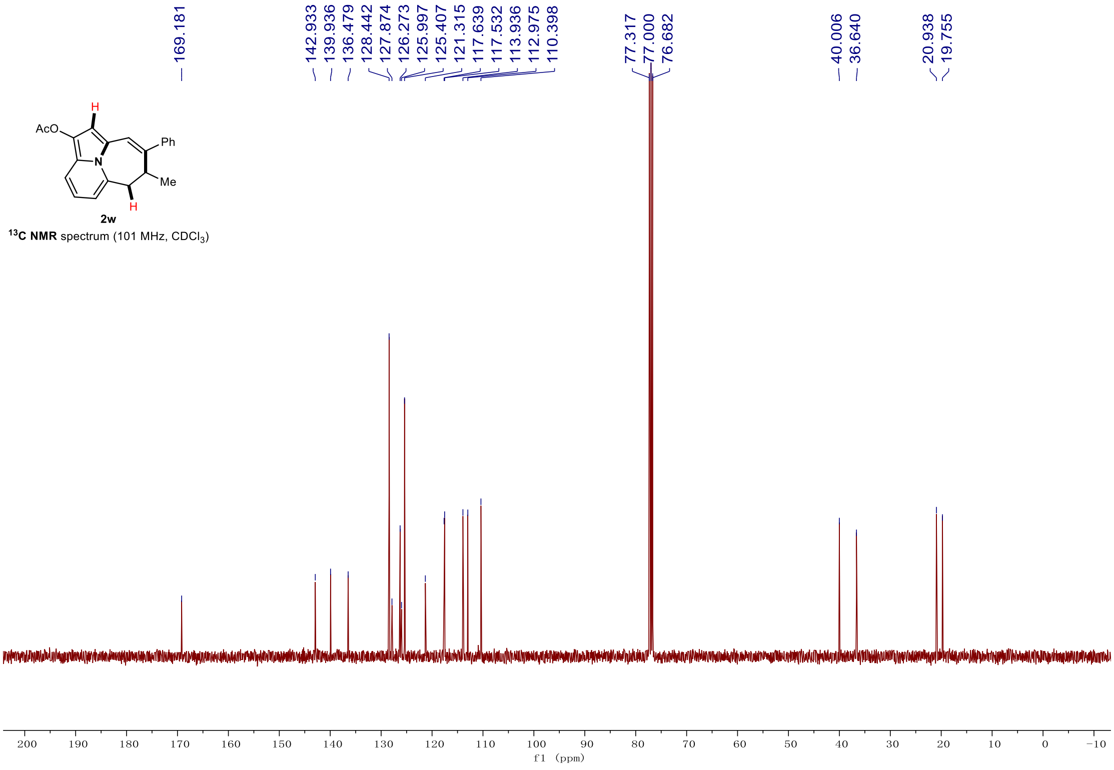

Supplementary Figure 139. ^{13}C NMR (101 MHz, $CDCl_3$) spectra for **2w**

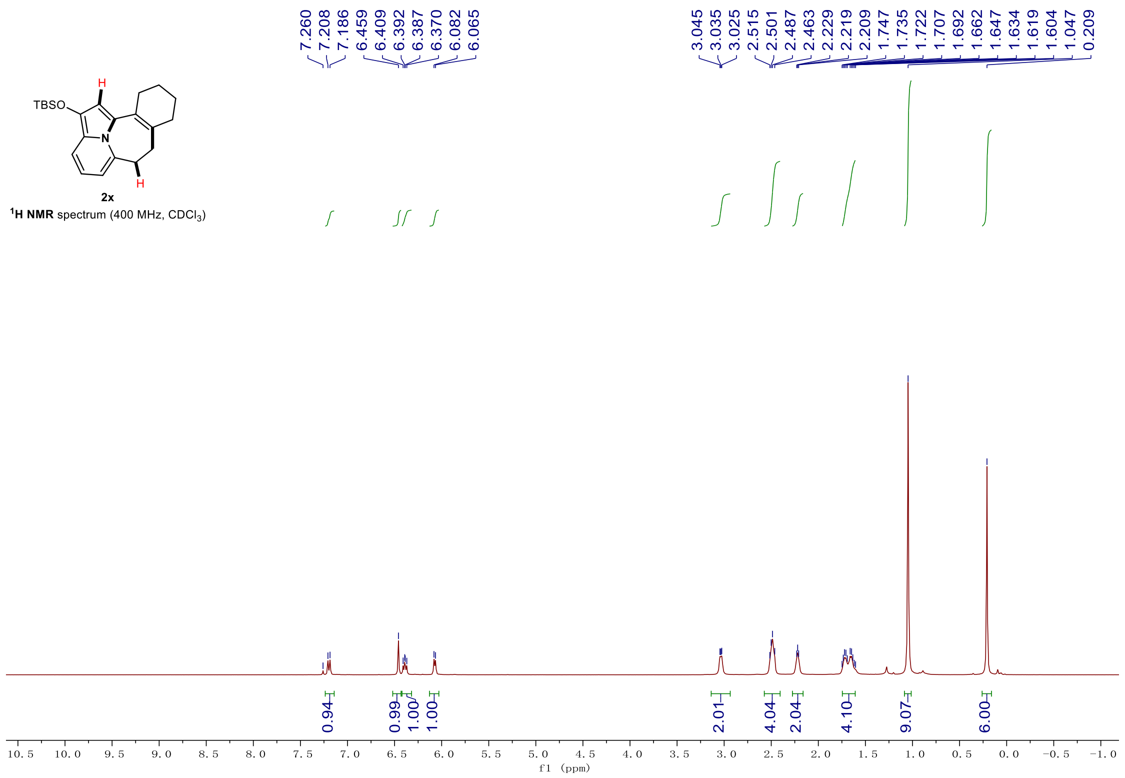

Supplementary Figure 140. ^{1}H NMR (400 MHz, $CDCl_3$) spectra for **2x**

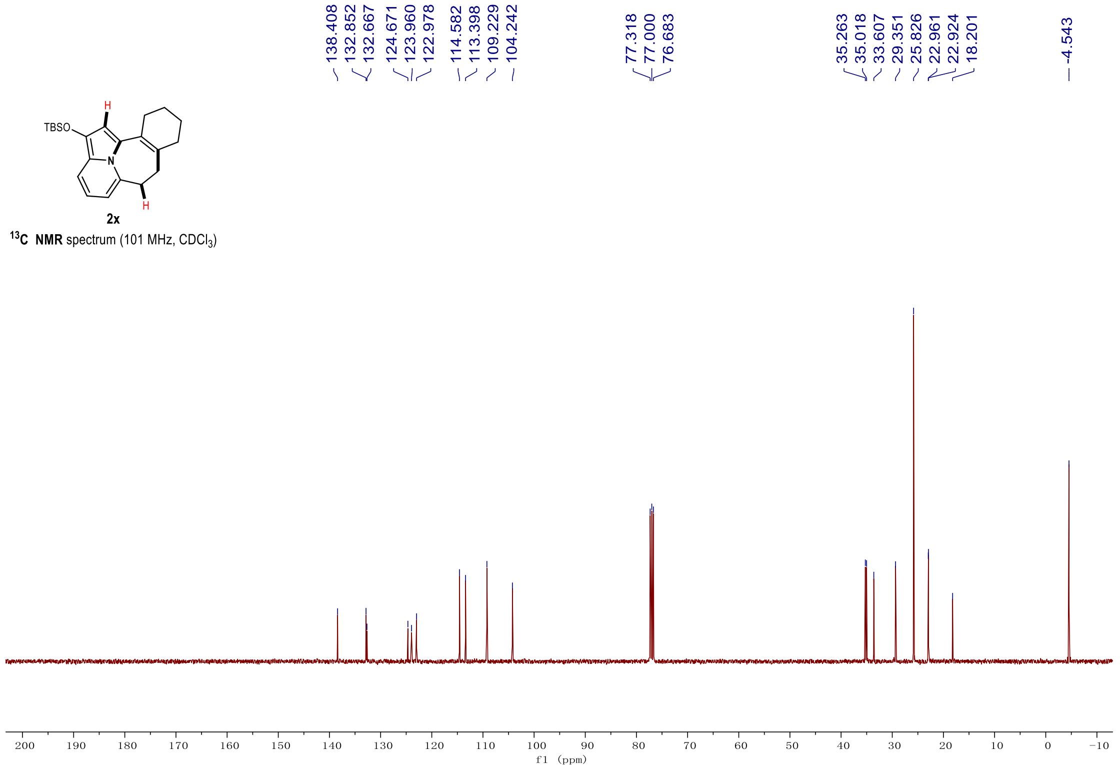

Supplementary Figure 141. ^{13}C NMR (101 MHz, $CDCl_3$) spectra for **2x**

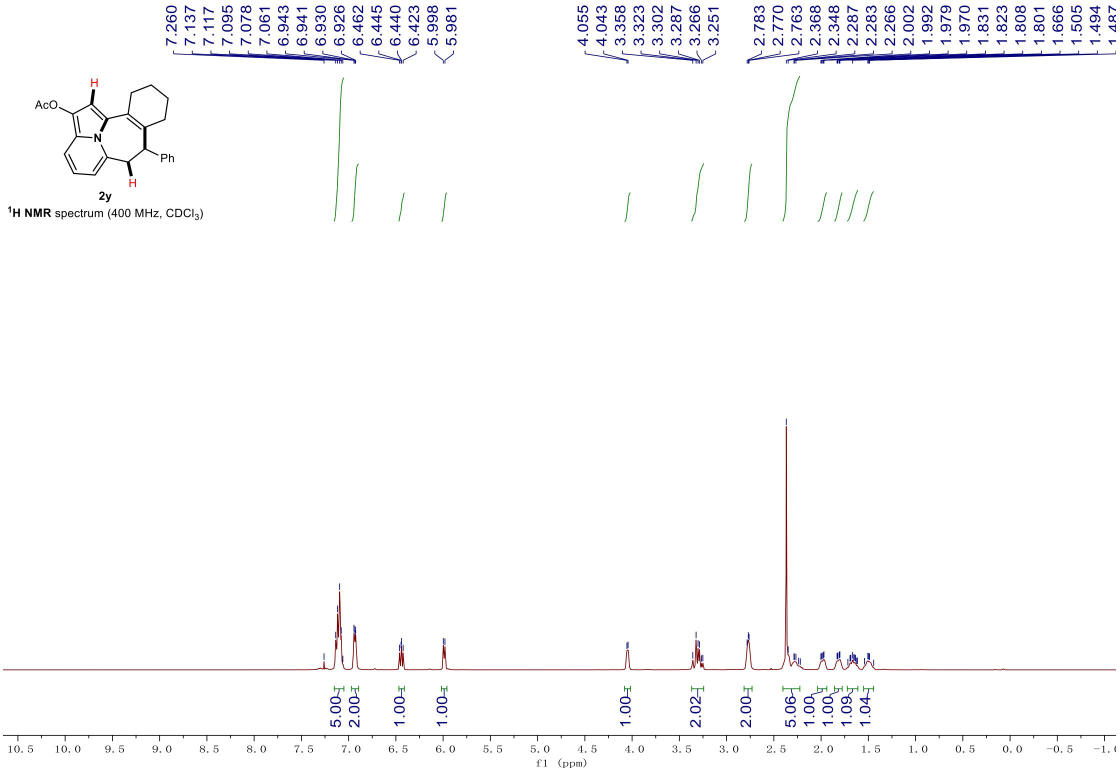

Supplementary Figure 142. ^{1}H NMR (400 MHz, $CDCl_3$) spectra for **2y**

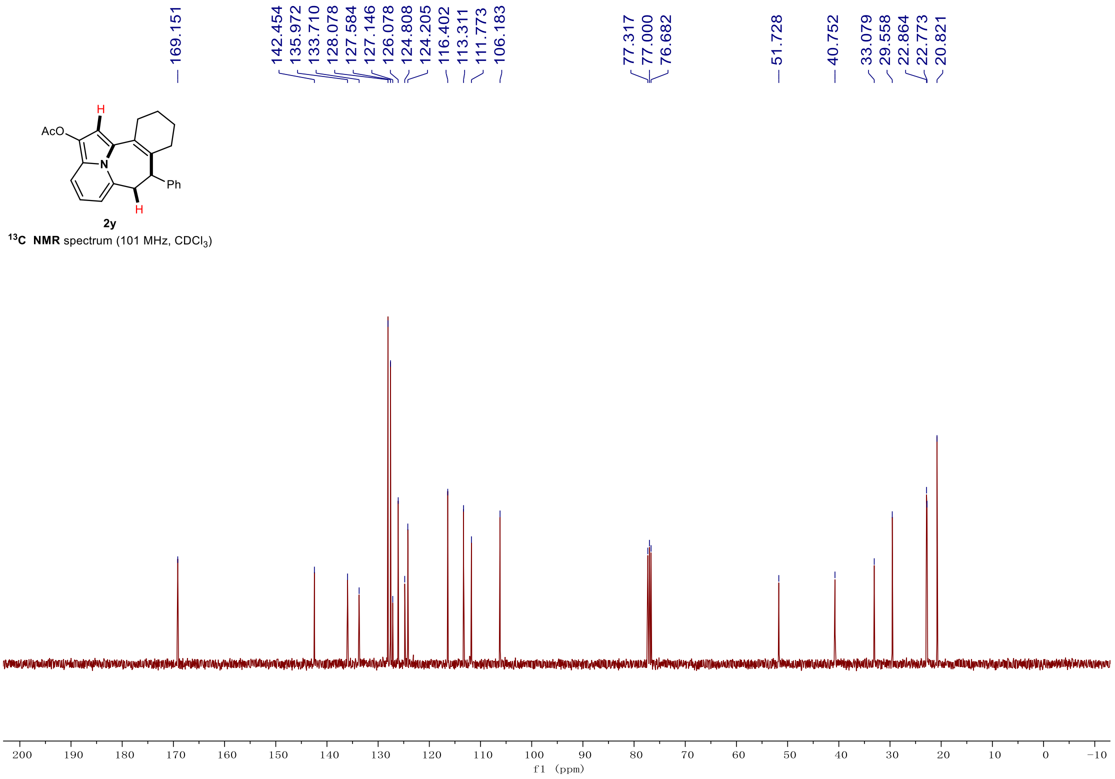

Supplementary Figure 143. ^{13}C NMR (101 MHz, $CDCl_3$) spectra for **2y**

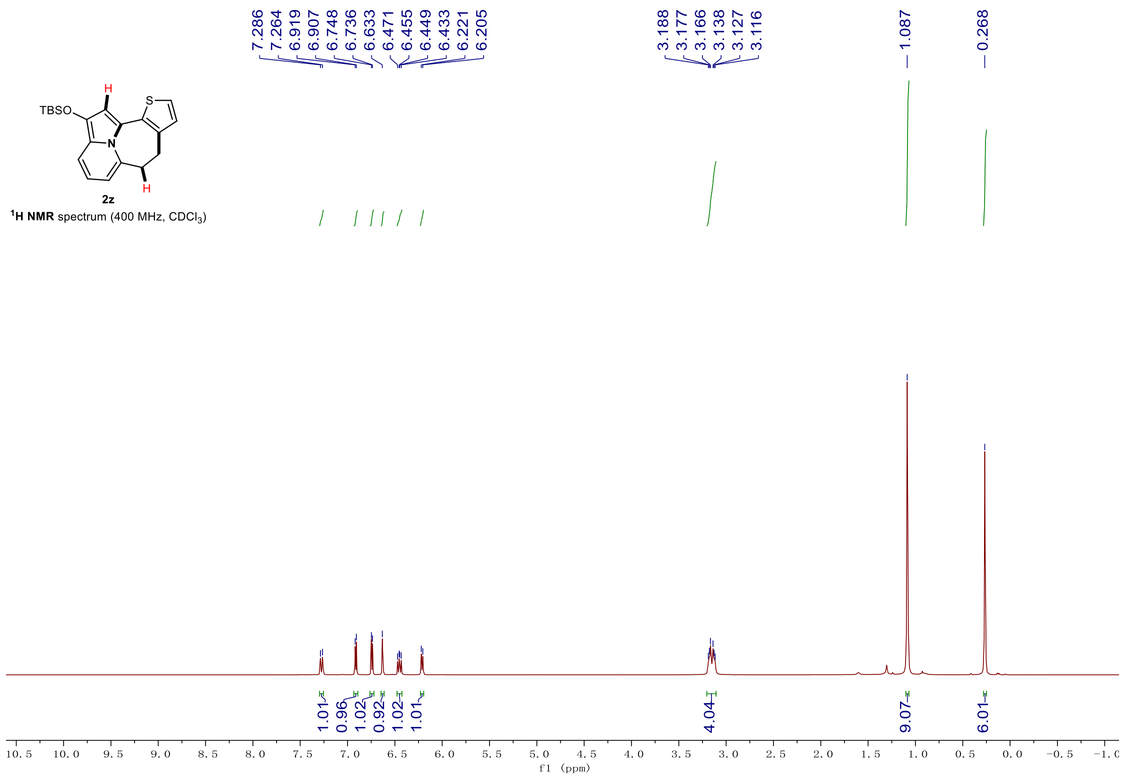

Supplementary Figure 144. ^{1}H NMR (400 MHz, $CDCl_3$) spectra for **2z**

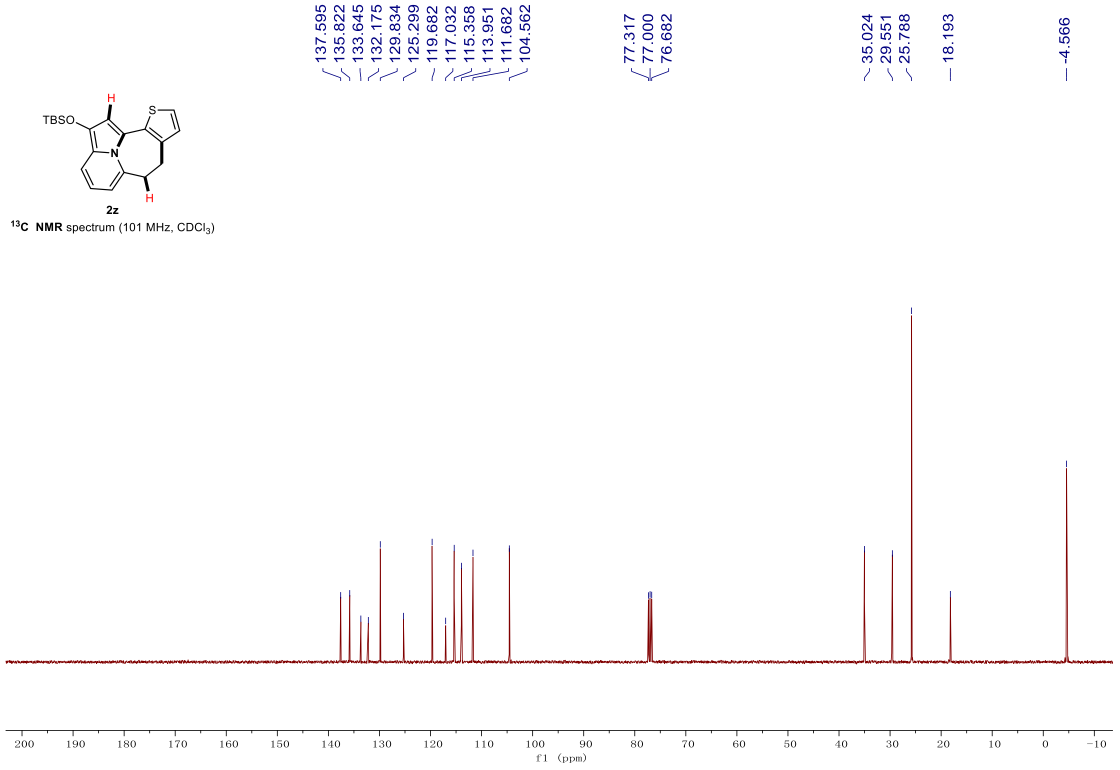

Supplementary Figure 145. ^{13}C NMR (101 MHz, $CDCl_3$) spectra for **2z**

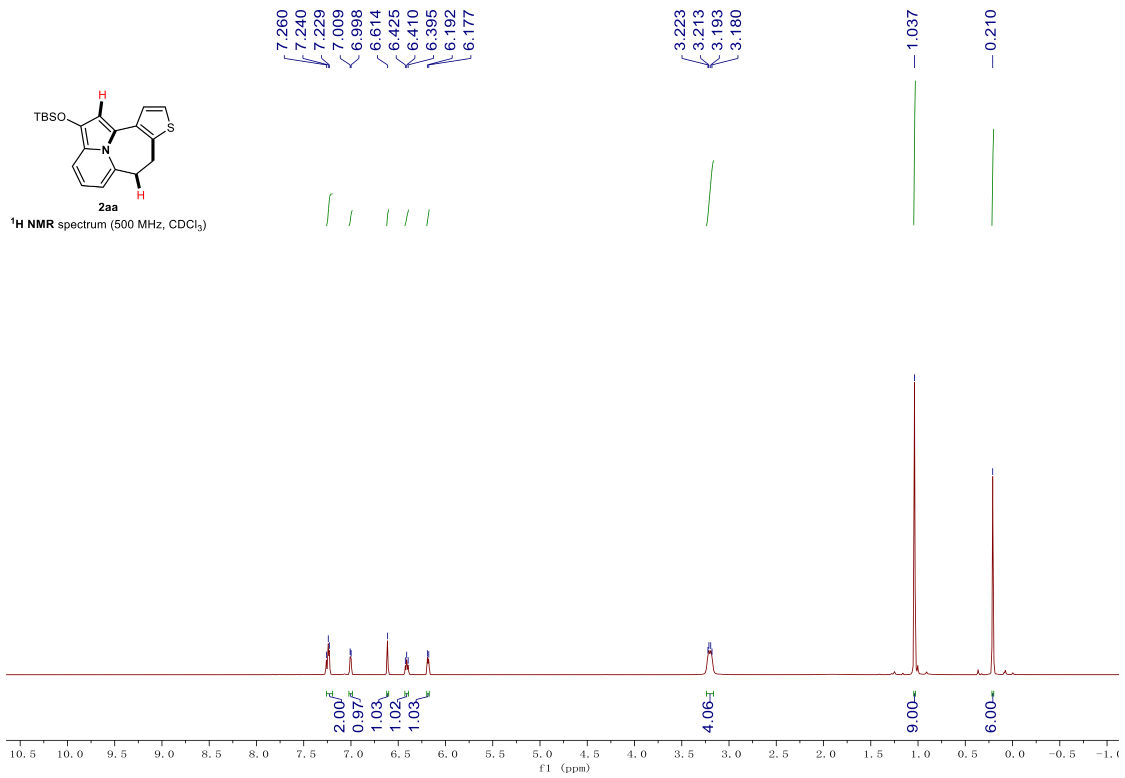

Supplementary Figure 146. ^{1}H NMR (500 MHz, $CDCl_3$) spectra for **2aa**

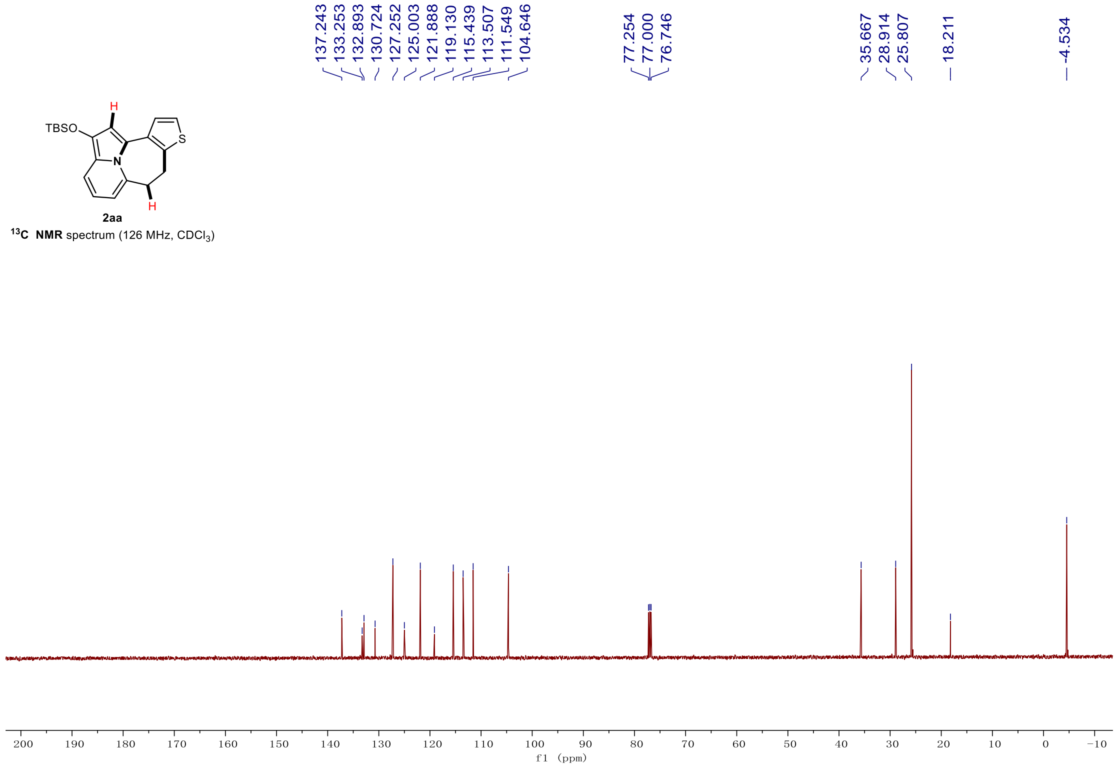

Supplementary Figure 147. ^{13}C NMR (126 MHz, $CDCl_3$) spectra for **2aa**

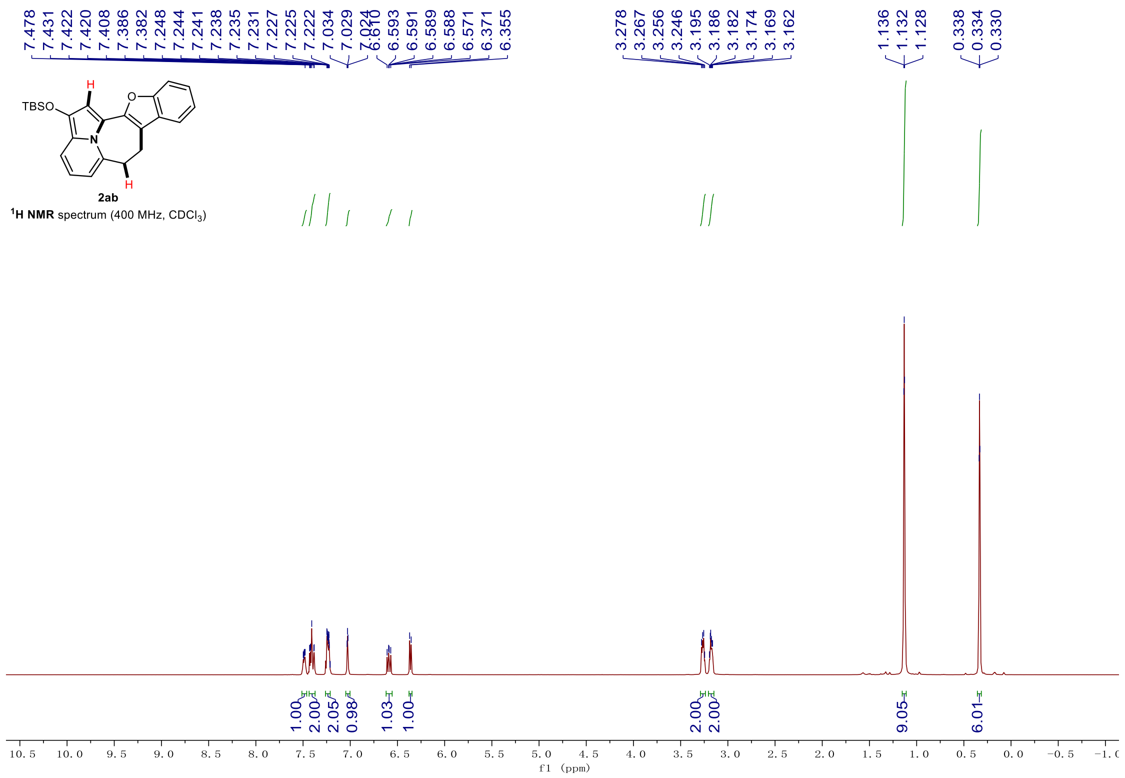

Supplementary Figure 148. ^{1}H NMR (400 MHz, $CDCl_3$) spectra for **2ab**

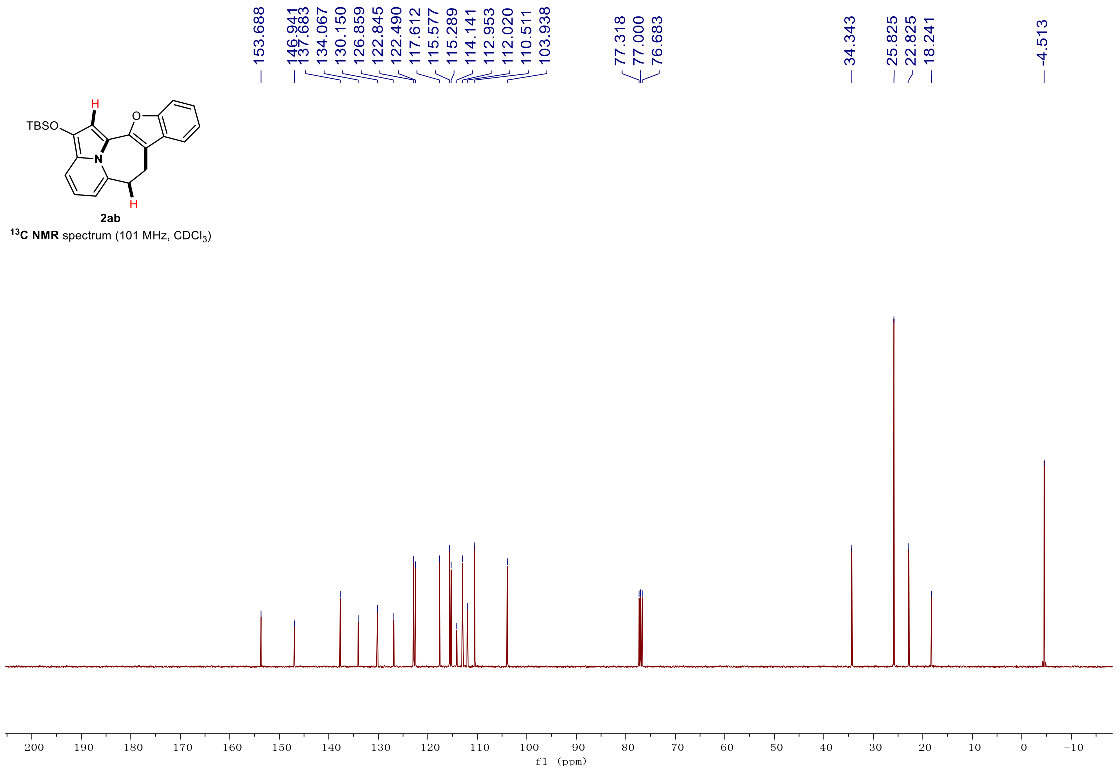

Supplementary Figure 149. ^{13}C NMR (101 MHz, $CDCl_3$) spectra for **2ab**

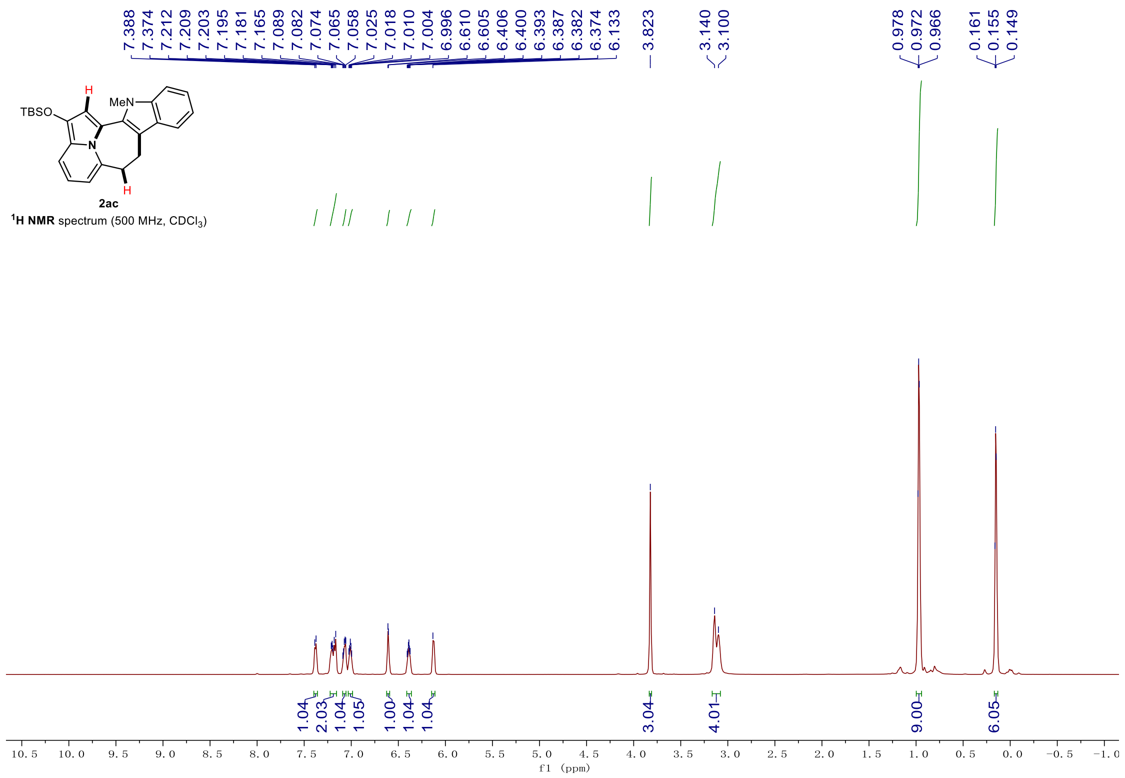

Supplementary Figure 150. ^{1}H NMR (500 MHz, $CDCl_3$) spectra for **2ac**

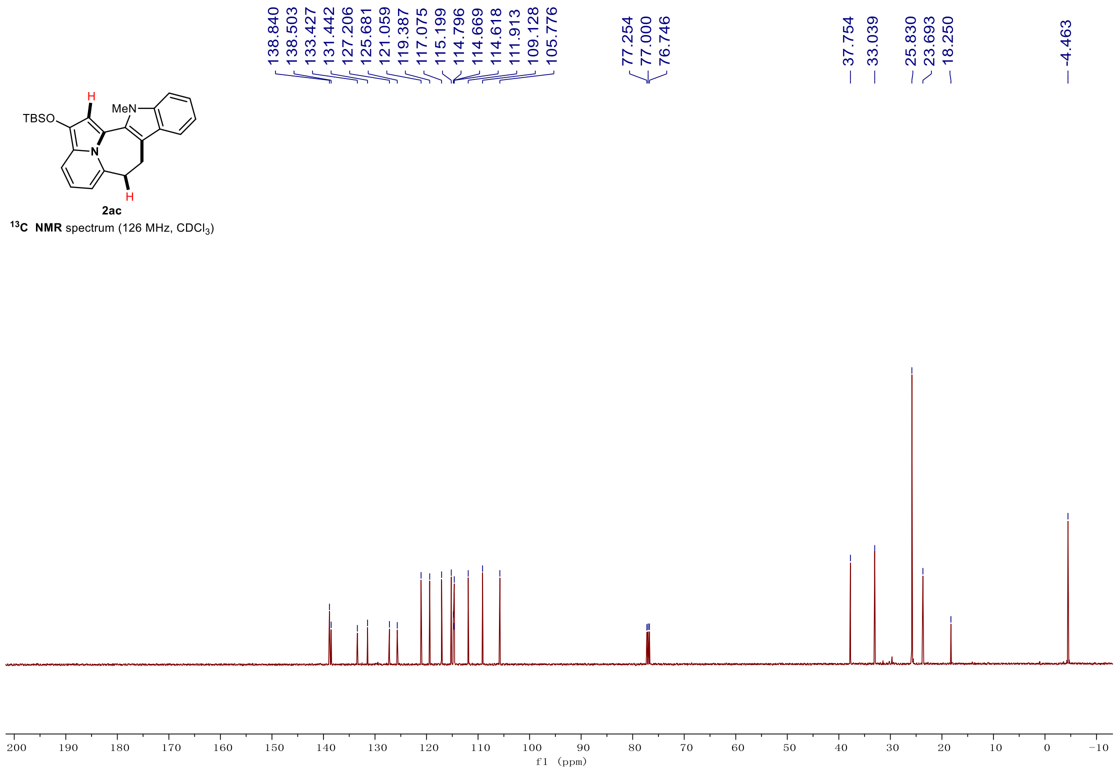

Supplementary Figure 151. ^{13}C NMR (126 MHz, $CDCl_3$) spectra for **2ac**

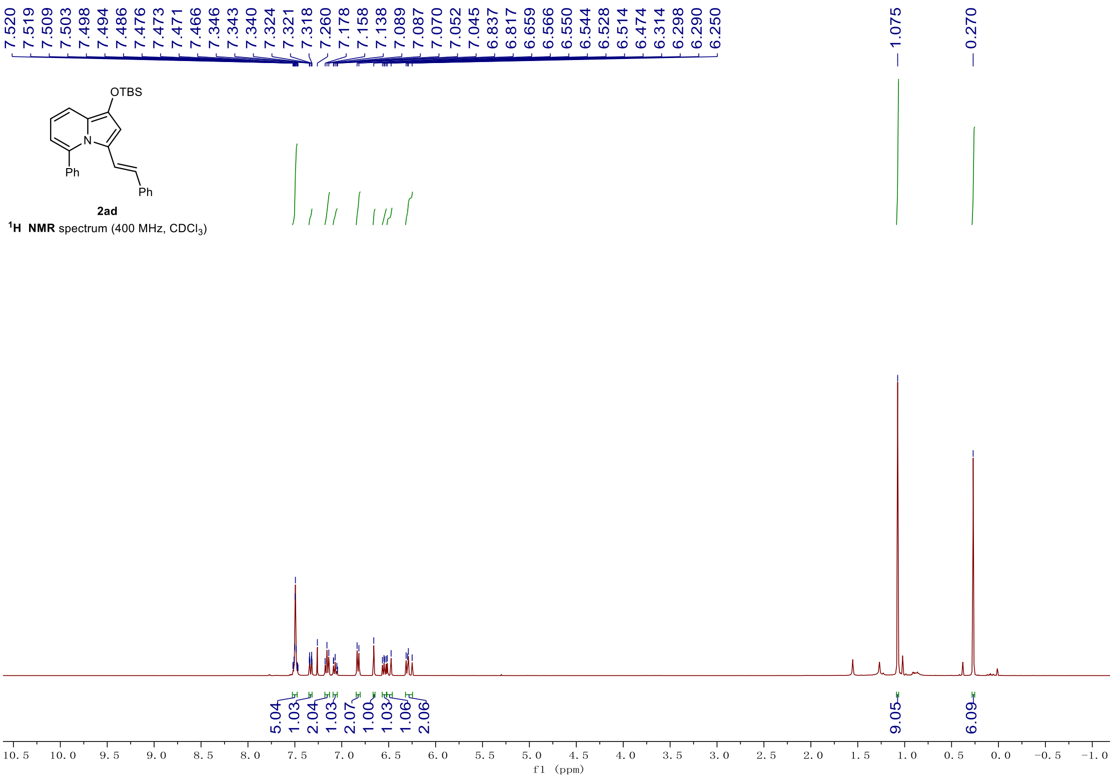

Supplementary Figure 152. ^{1}H NMR (400 MHz, $CDCl_3$) spectra for **2ad**

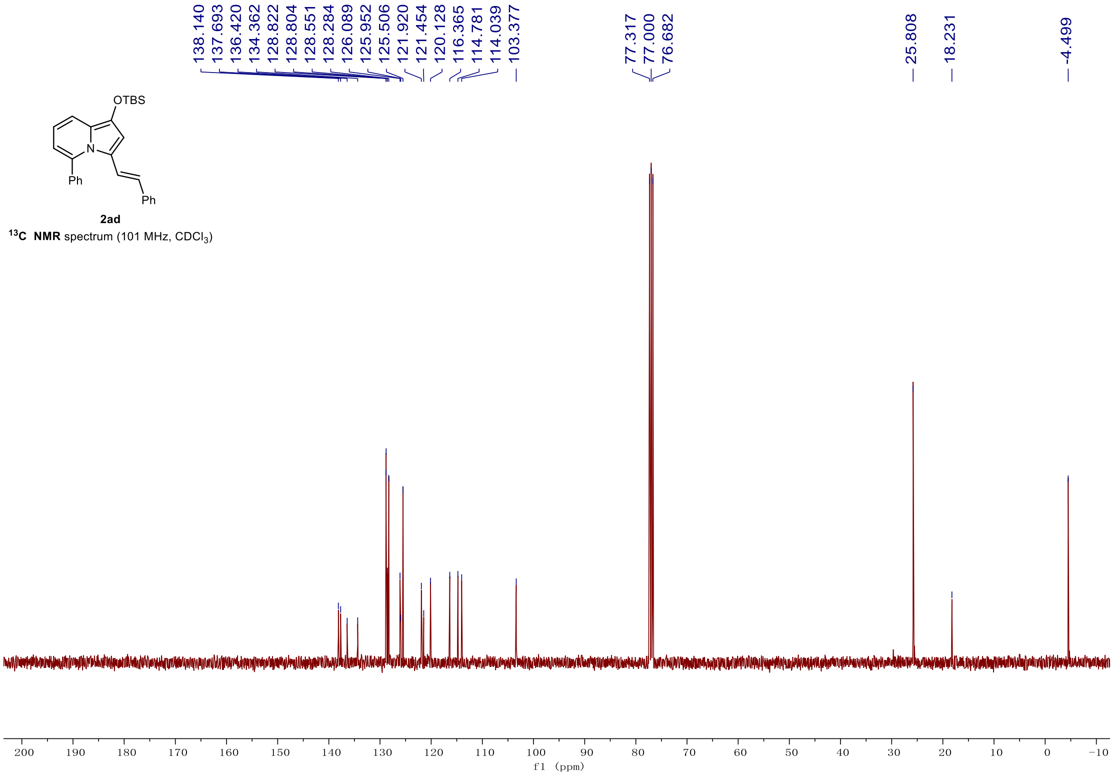

Supplementary Figure 153. ^{13}C NMR (101 MHz, $CDCl_3$) spectra for **2ad**

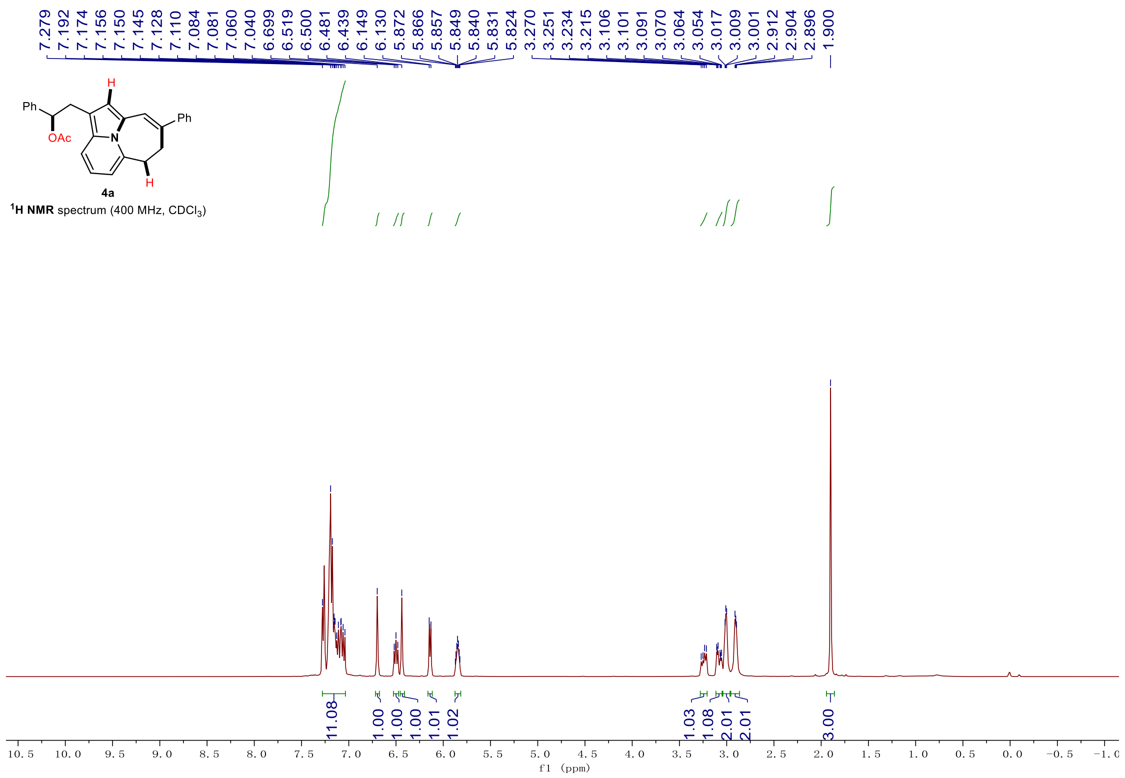

Supplementary Figure 154. ^{1}H NMR (400 MHz, $CDCl_3$) spectra for **4a**

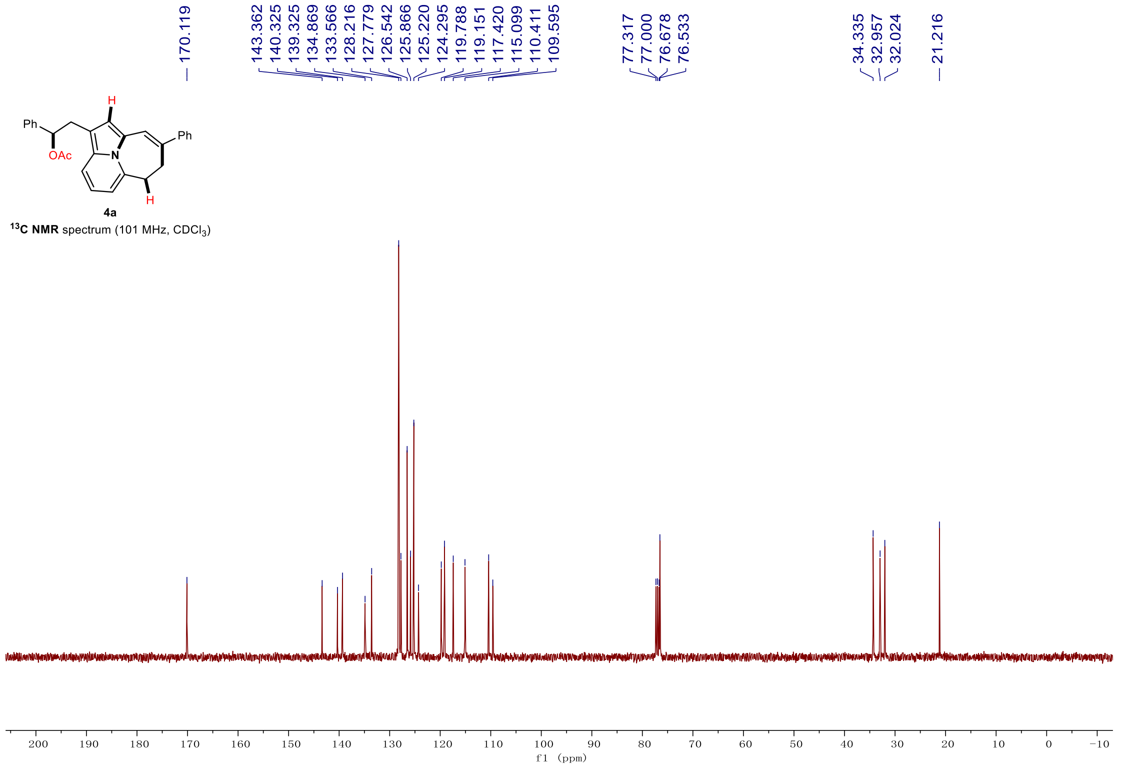

Supplementary Figure 155. ^{13}C NMR (101 MHz, $CDCl_3$) spectra for **4a**

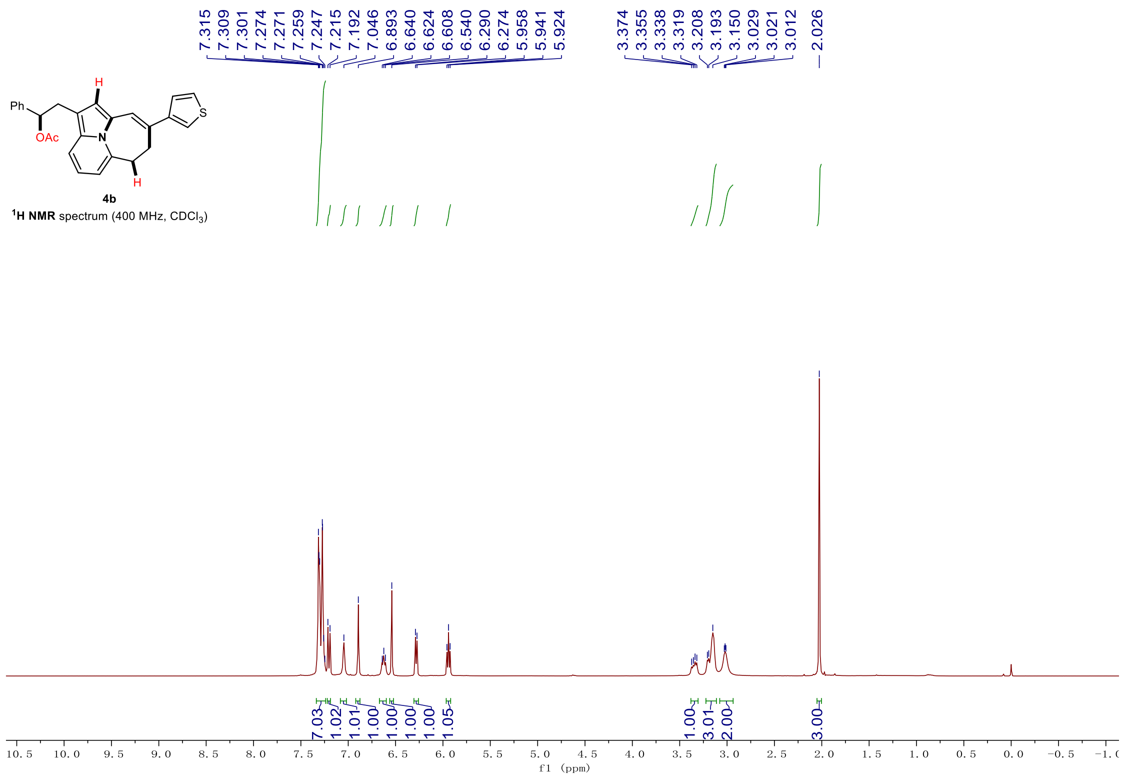

Supplementary Figure 156. ^{1}H NMR (400 MHz, $CDCl_3$) spectra for **4b**

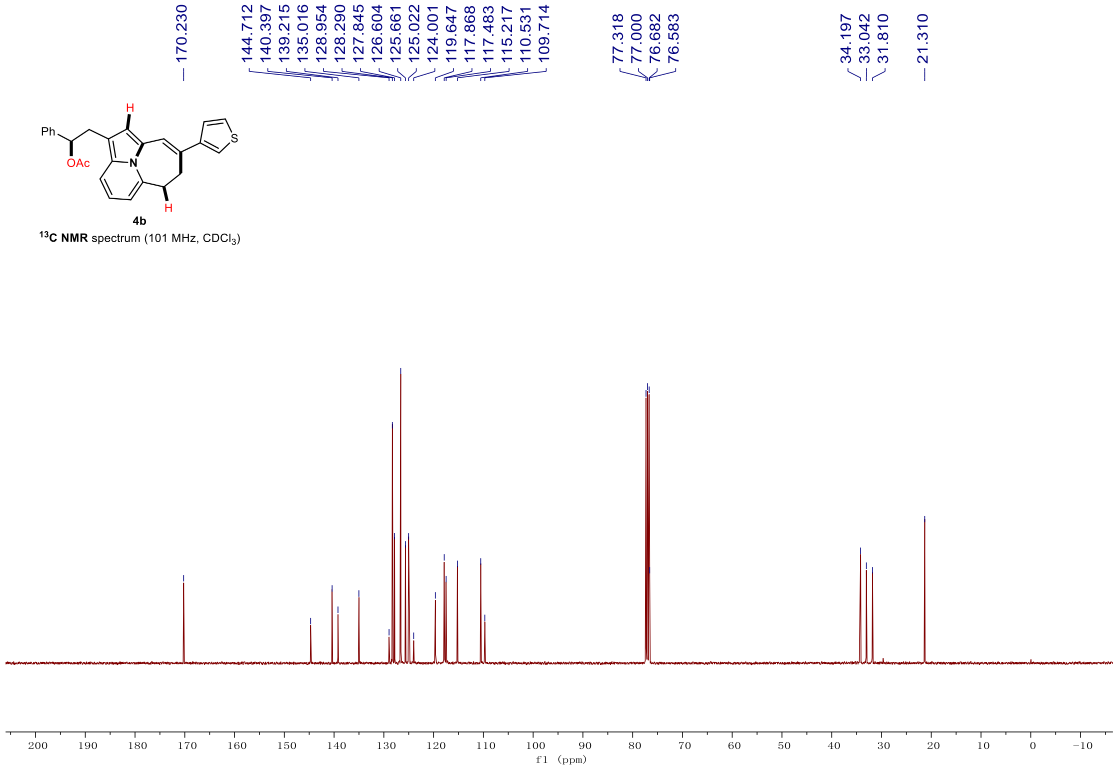

Supplementary Figure 157. ^{13}C NMR (101 MHz, $CDCl_3$) spectra for **4b**

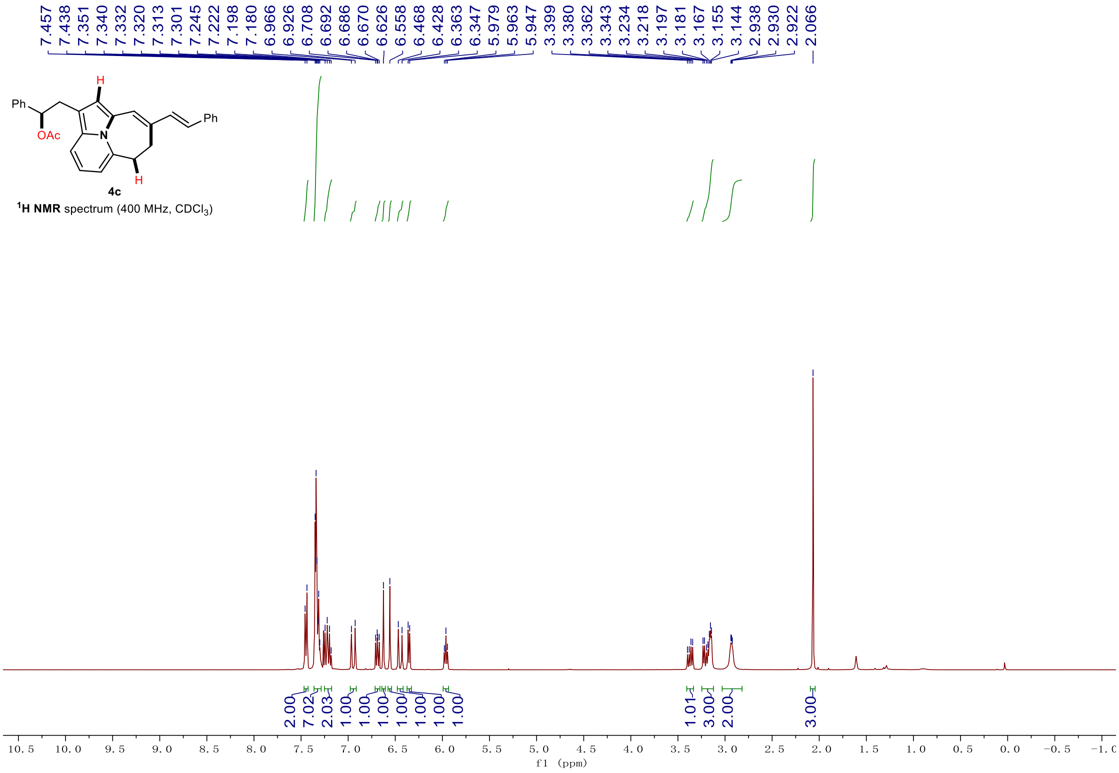

Supplementary Figure 158. ^{1}H NMR (400 MHz, $CDCl_3$) spectra for **4c**

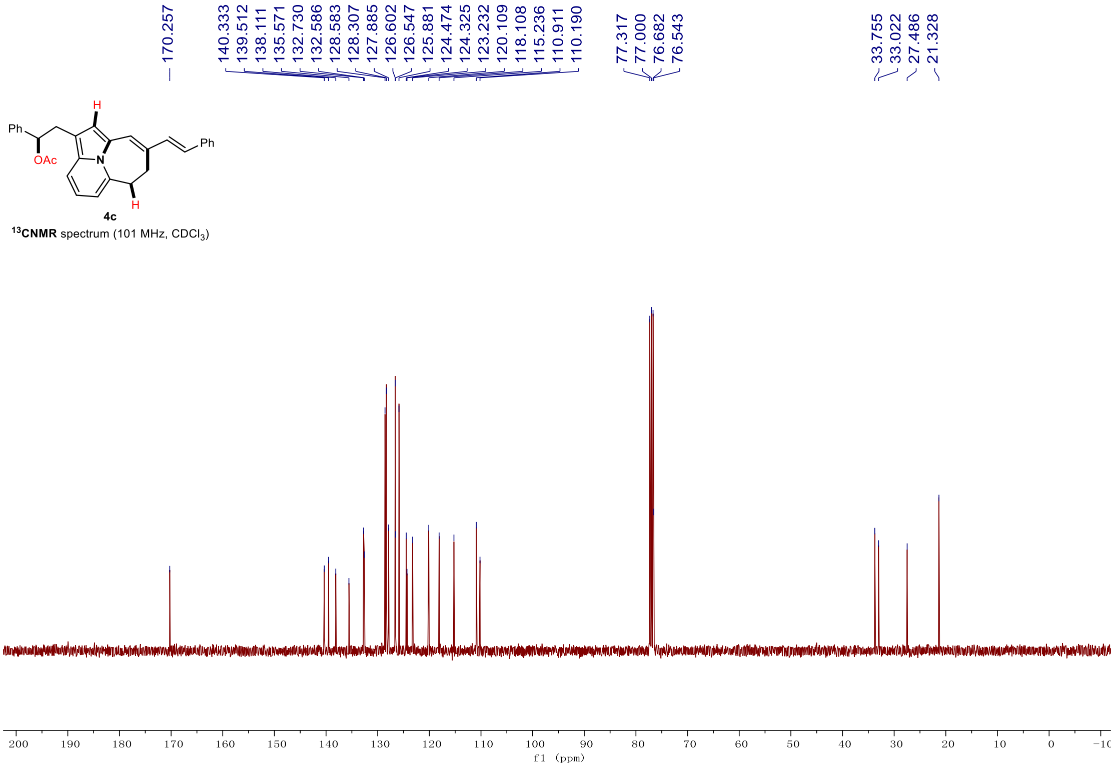

Supplementary Figure 159. ^{13}C NMR (101 MHz, $CDCl_3$) spectra for **4c**

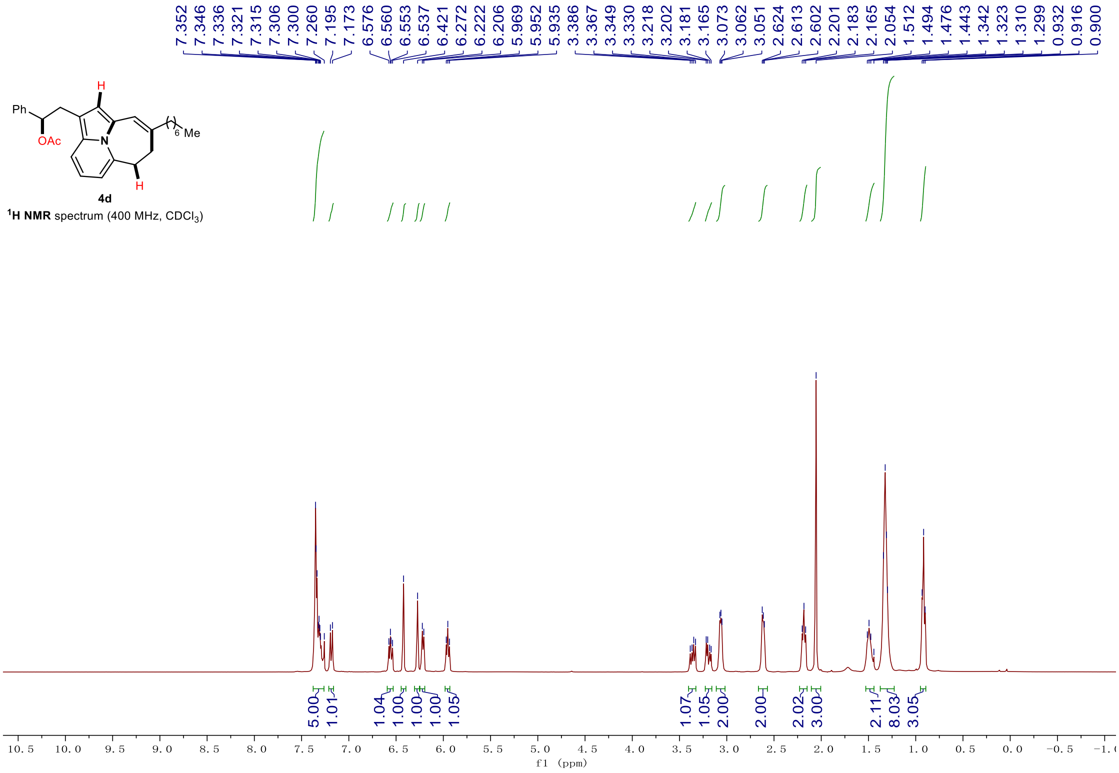

Supplementary Figure 160. ^{1}H NMR (400 MHz, $CDCl_3$) spectra for **4d**

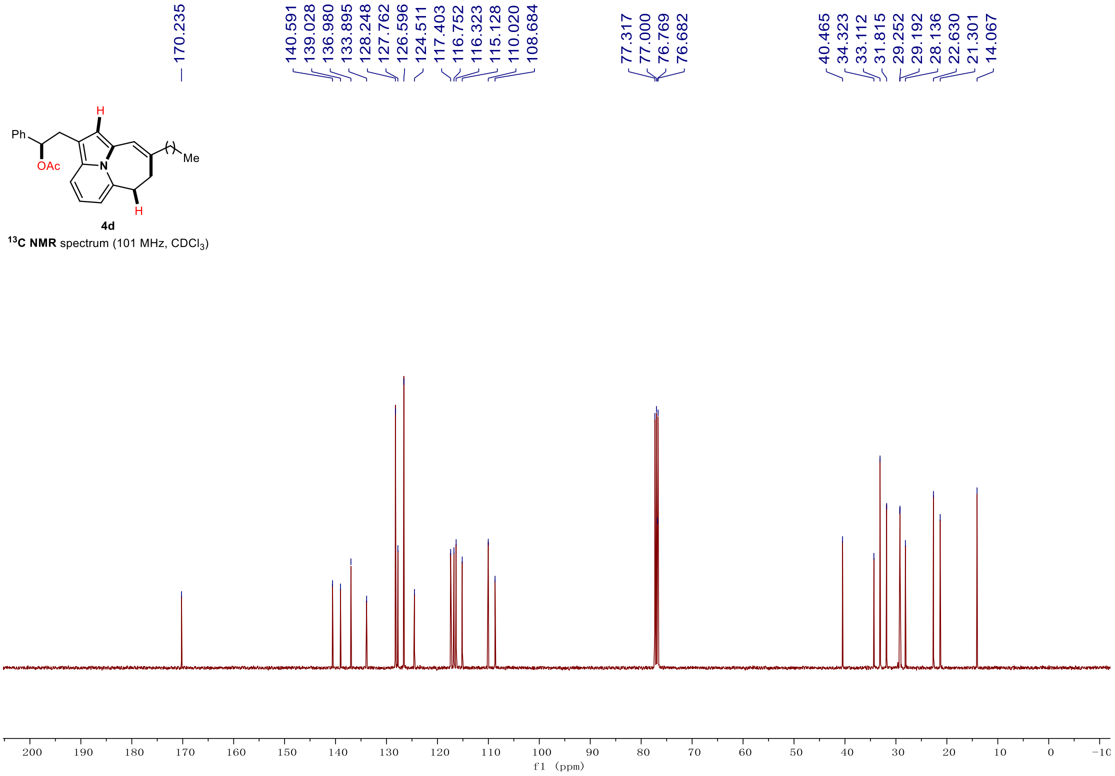

Supplementary Figure 161. ^{13}C NMR (101 MHz, $CDCl_3$) spectra for **4d**

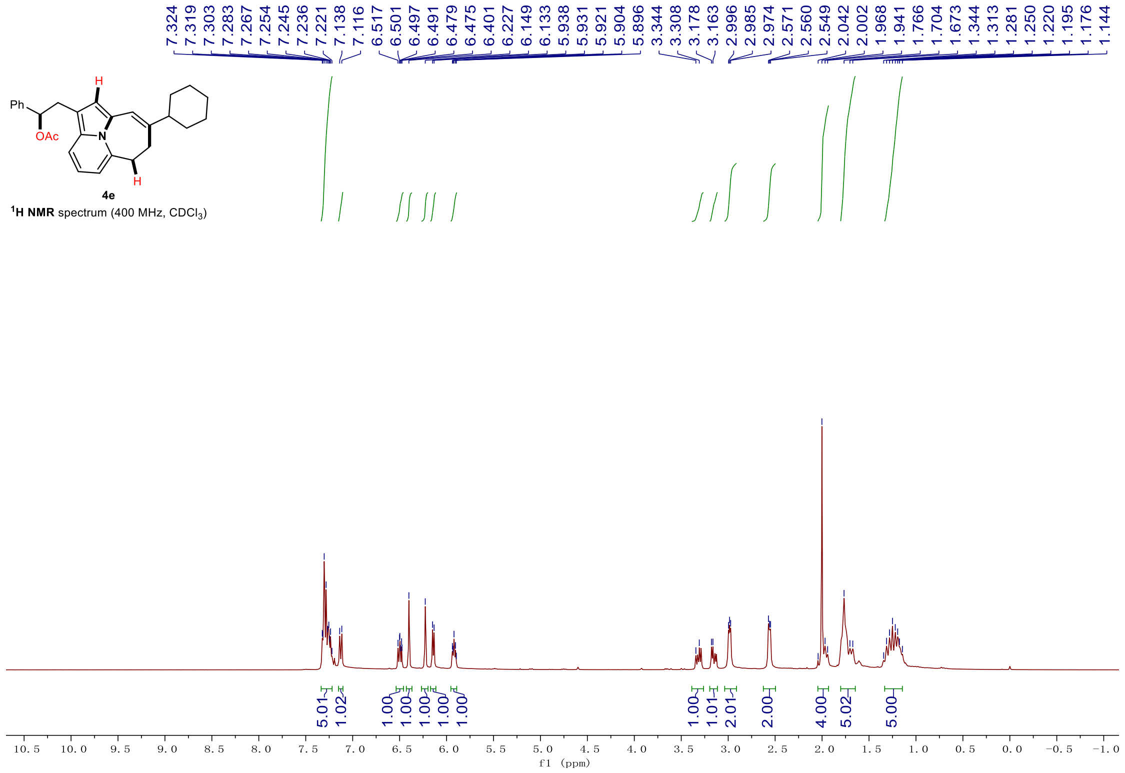

Supplementary Figure 162. ^{1}H NMR (400 MHz, $CDCl_3$) spectra for **4e**

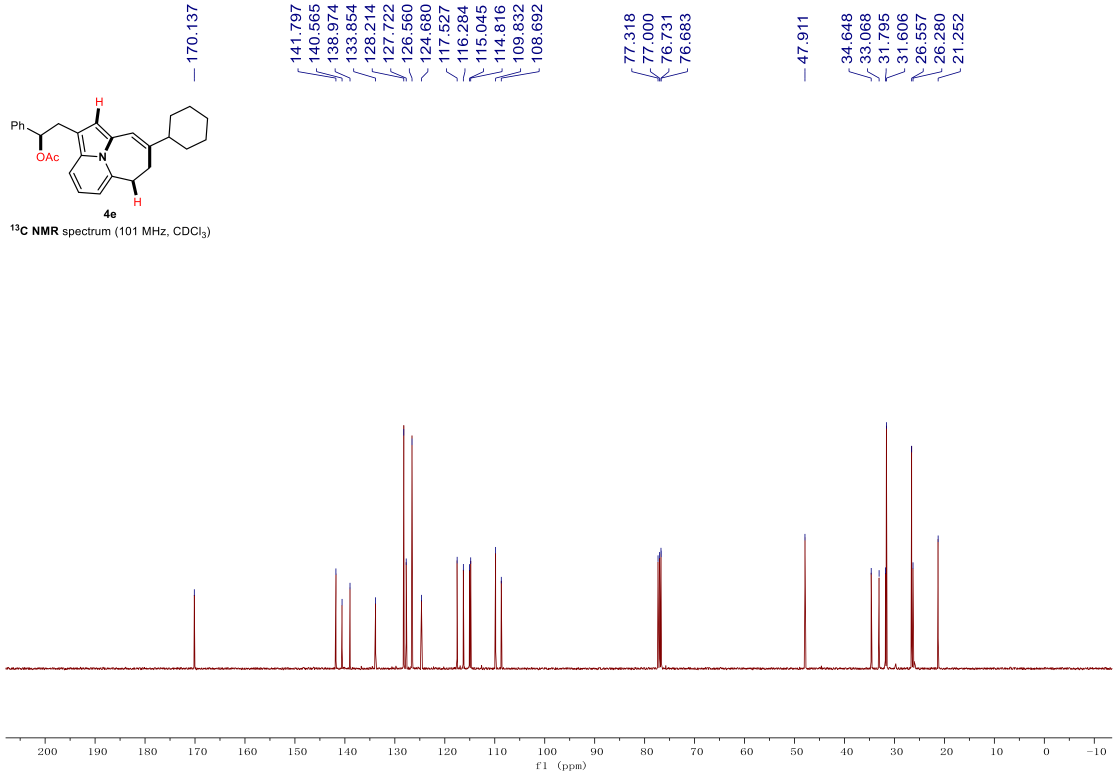

Supplementary Figure 163. ^{13}C NMR (101 MHz, $CDCl_3$) spectra for **4e**

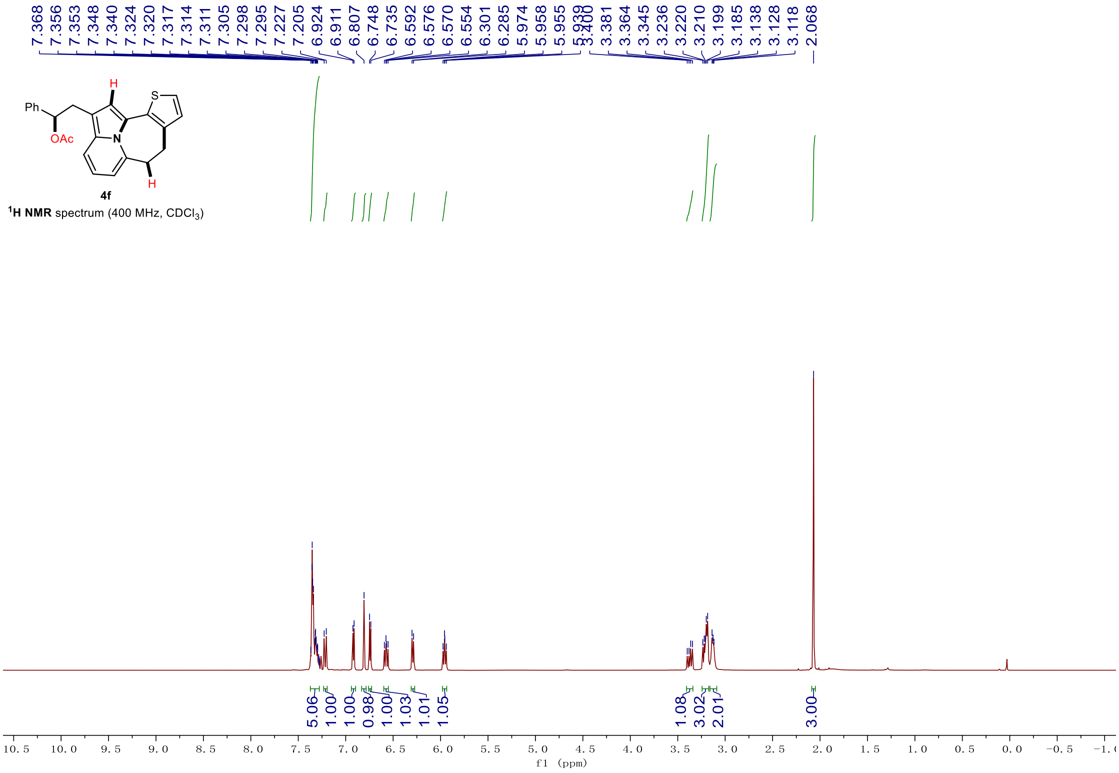

Supplementary Figure 164. ^{1}H NMR (400 MHz, $CDCl_3$) spectra for **4f**

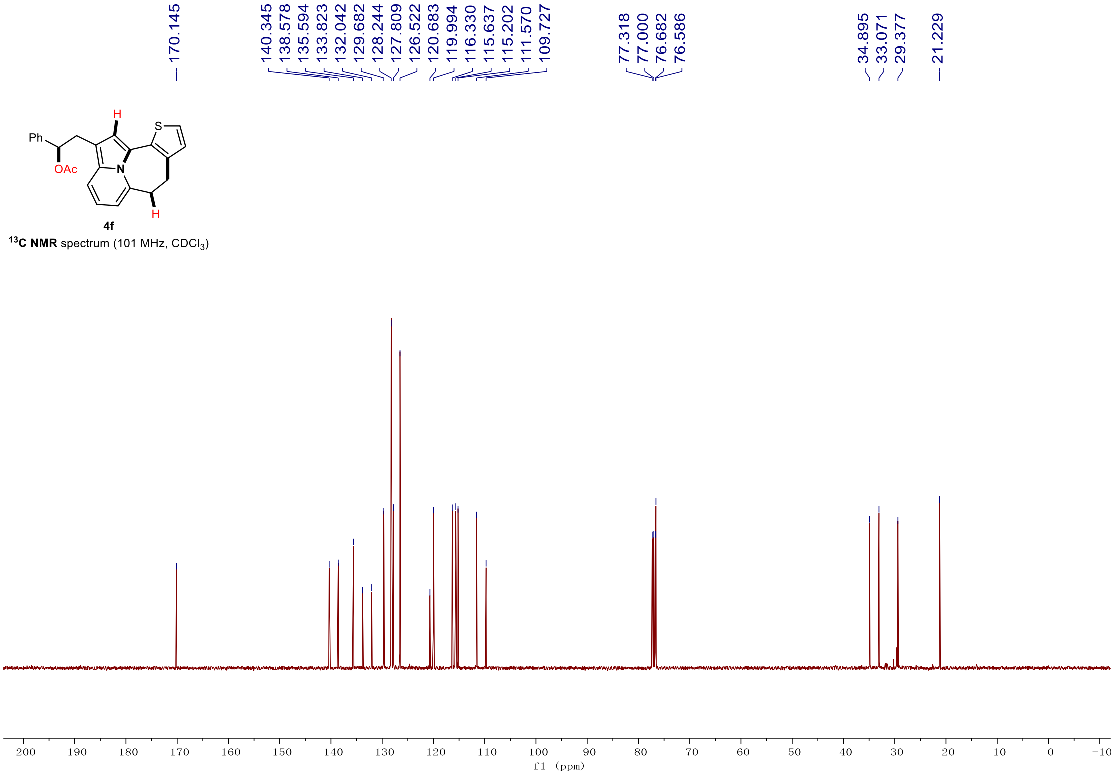

Supplementary Figure 165. ^{13}C NMR (101 MHz, $CDCl_3$) spectra for **4f**

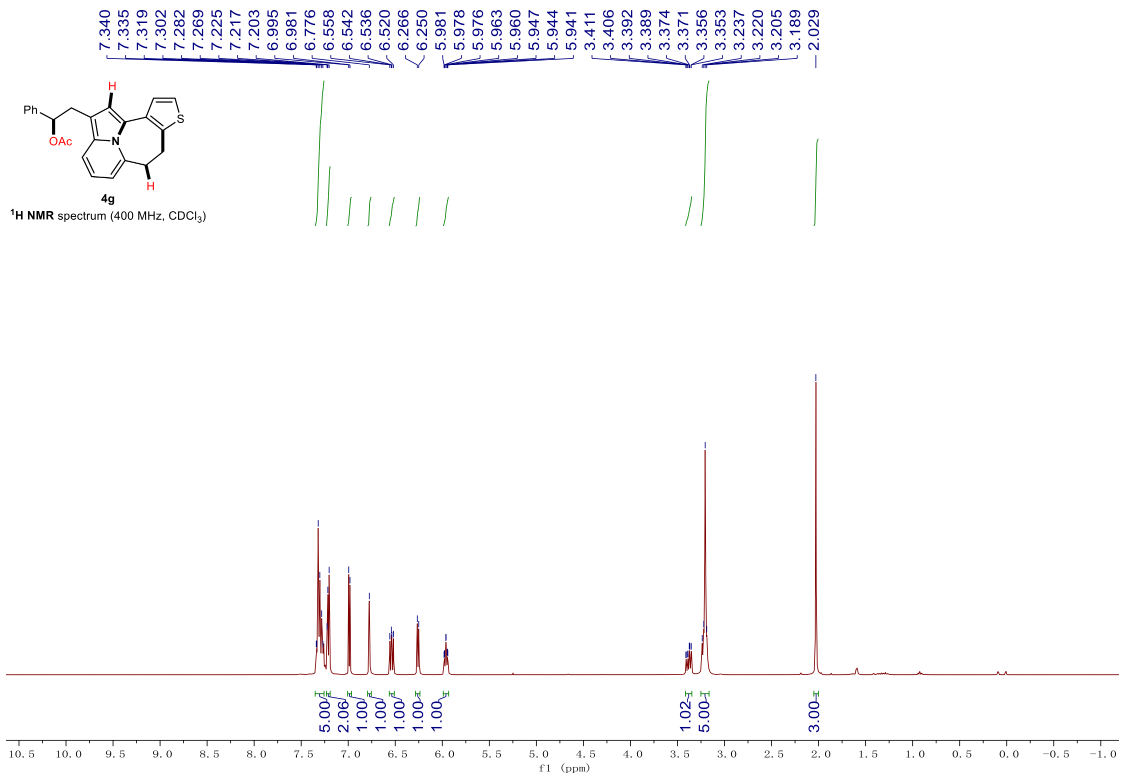

Supplementary Figure 166. ^{1}H NMR (400 MHz, $CDCl_3$) spectra for **4g**

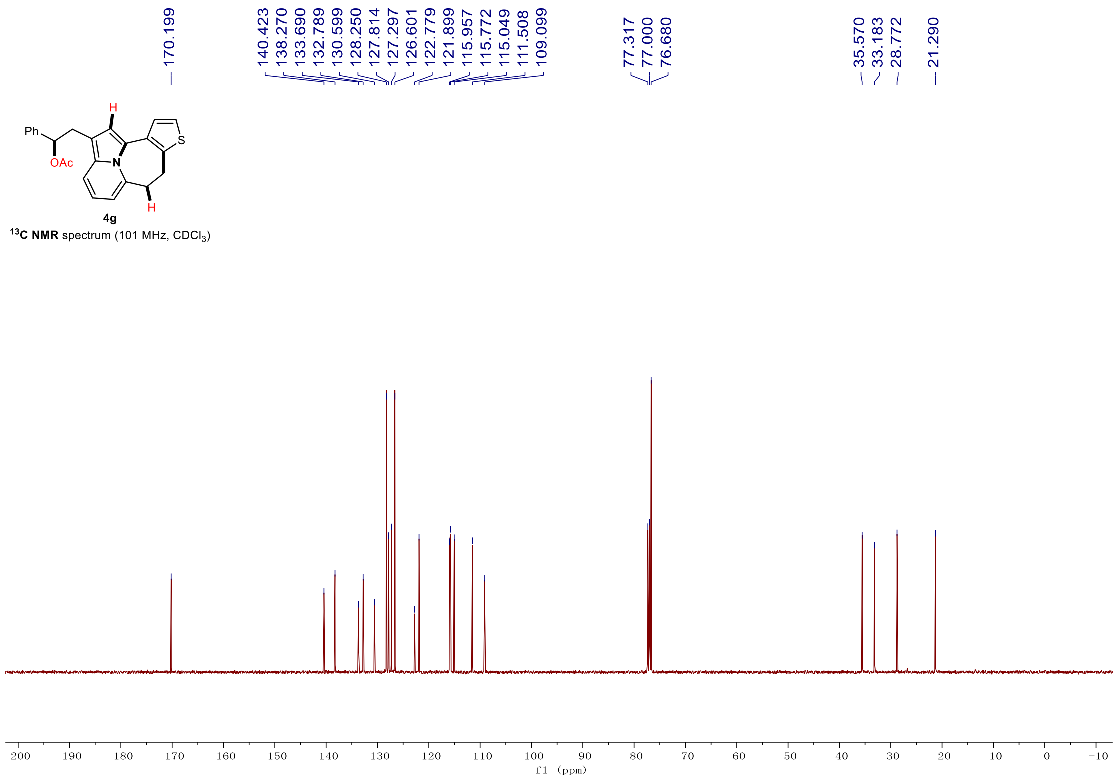

Supplementary Figure 167. ^{13}C NMR (101 MHz, $CDCl_3$) spectra for **4g**

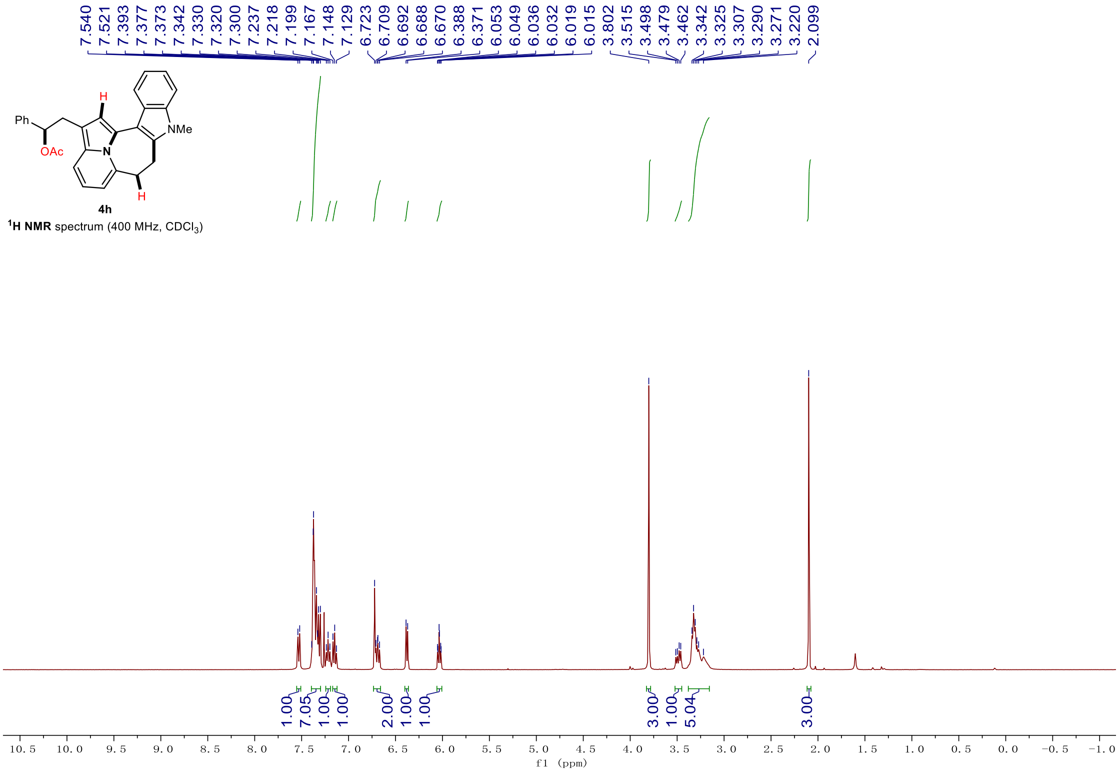

Supplementary Figure 168. ^{1}H NMR (400 MHz, $CDCl_3$) spectra for **4h**

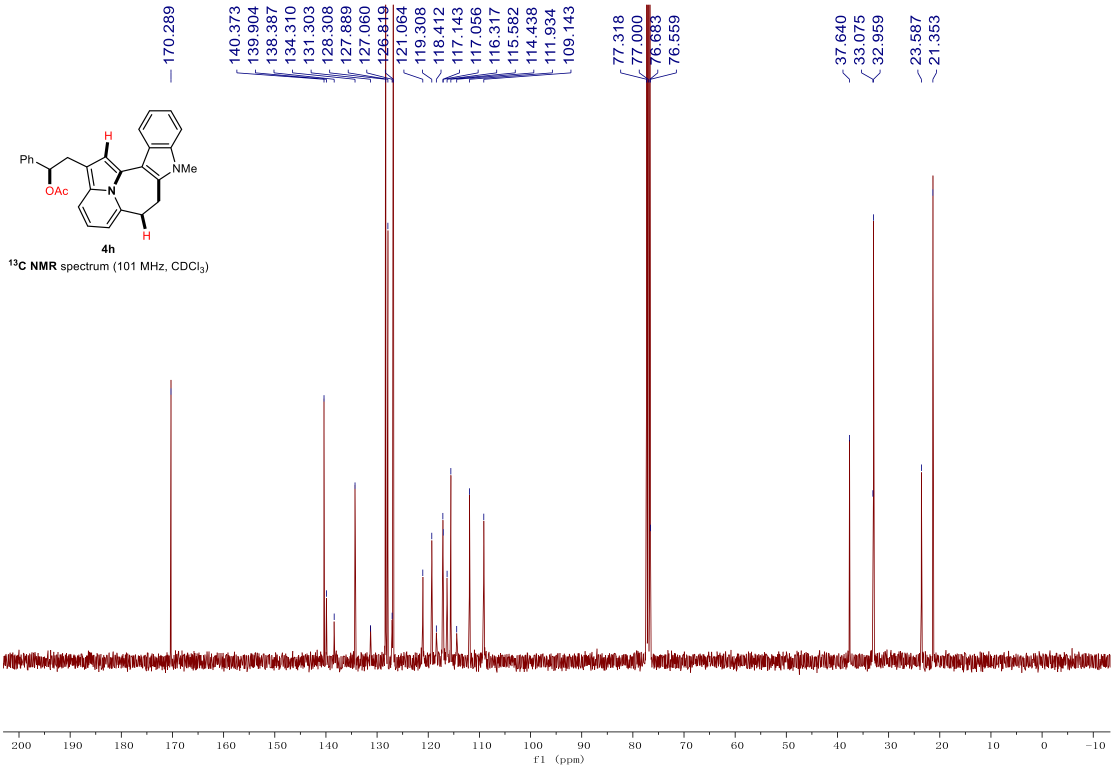

Supplementary Figure 169. ^{13}C NMR (101 MHz, $CDCl_3$) spectra for **4h**

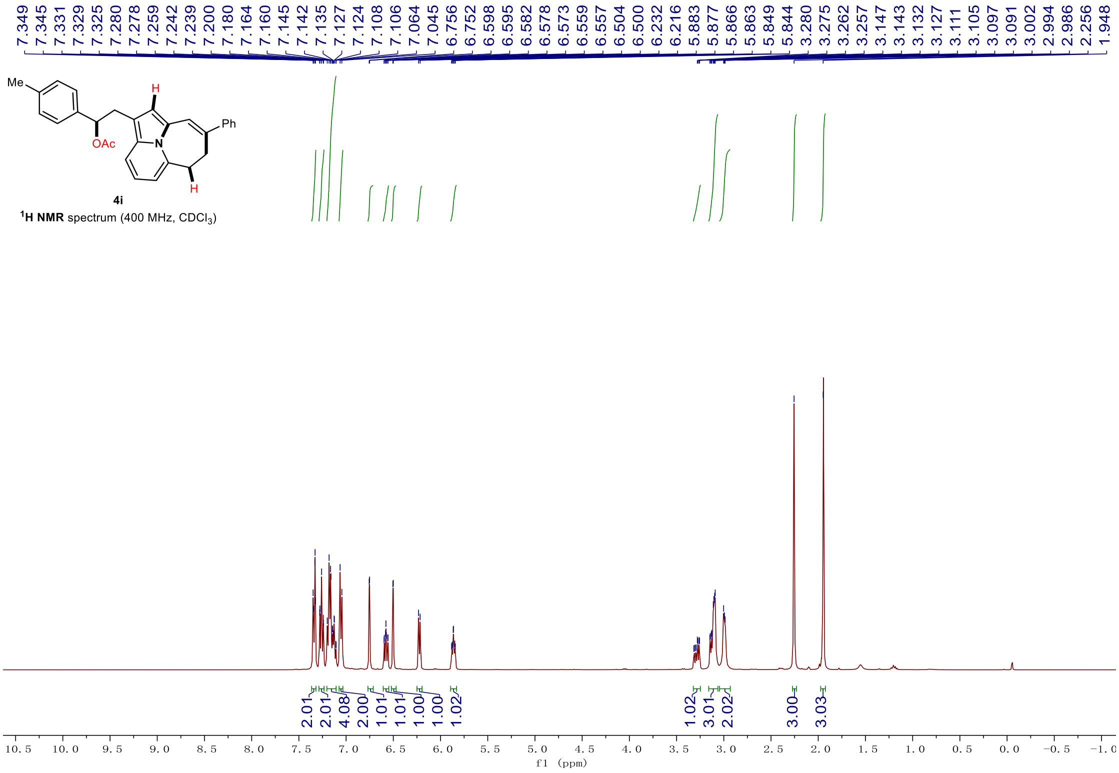

Supplementary Figure 170. ^{1}H NMR (400 MHz, $CDCl_3$) spectra for **4i**

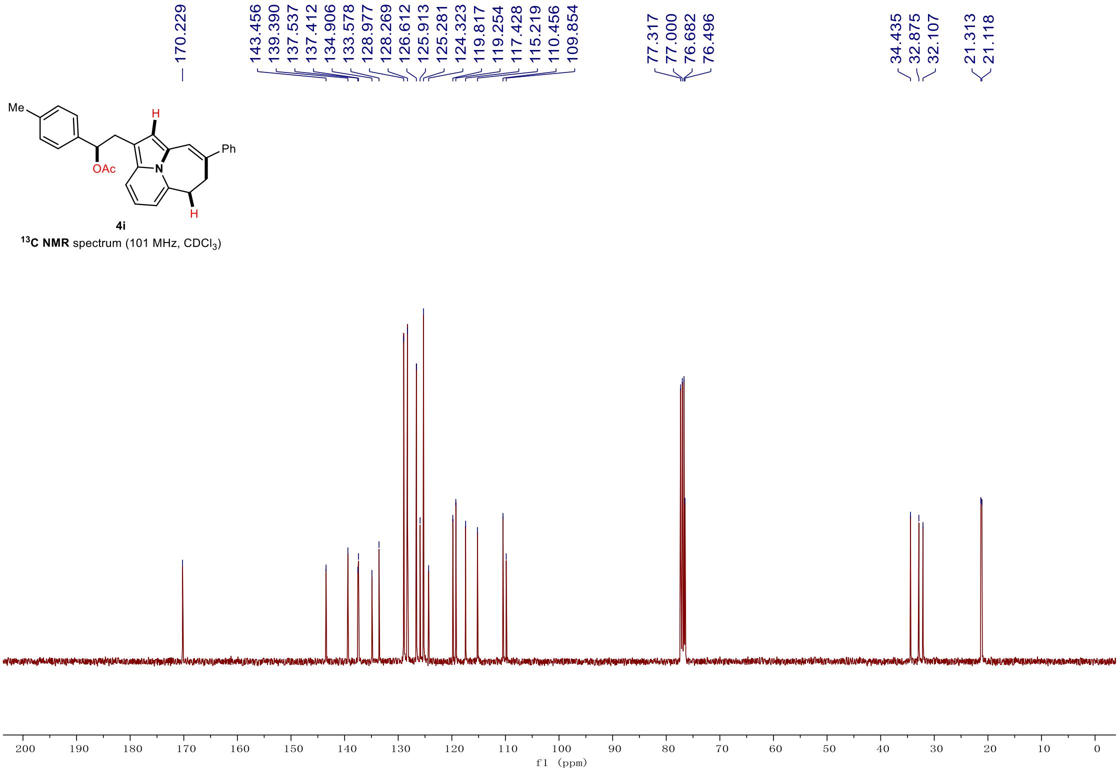

Supplementary Figure 171. ^{13}C NMR (101 MHz, $CDCl_3$) spectra for **4i**

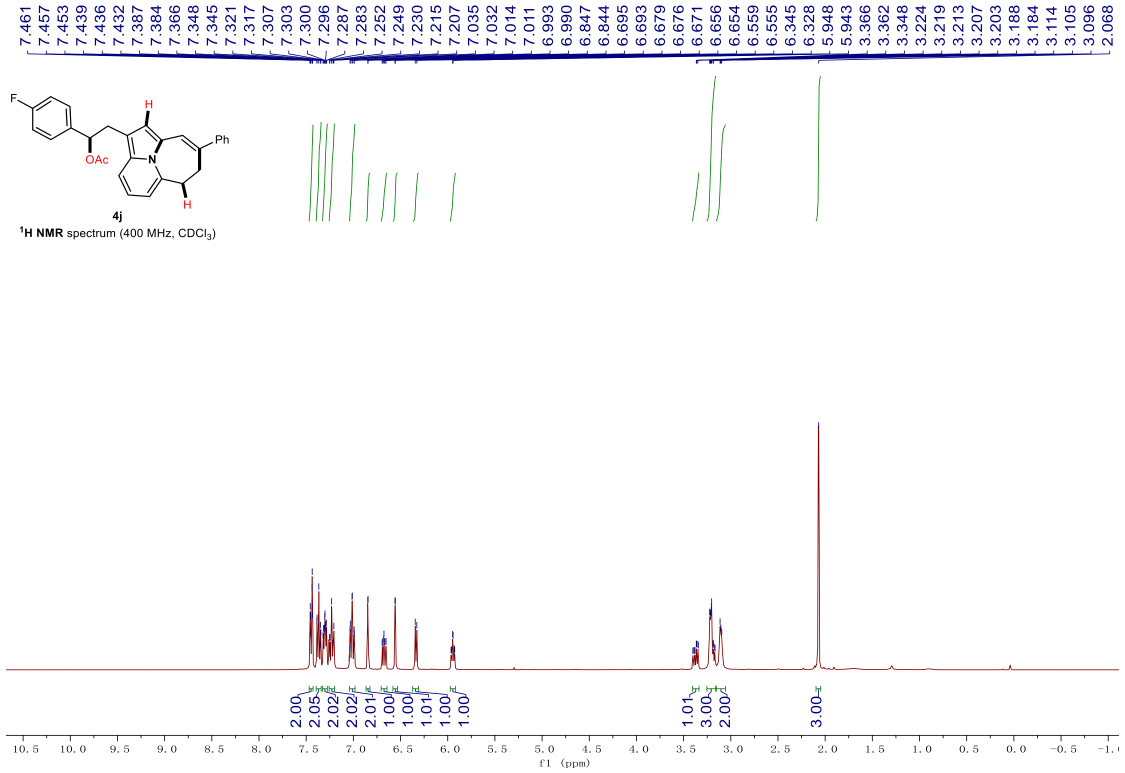

Supplementary Figure 172. ^{1}H NMR (400 MHz, $CDCl_3$) spectra for **4j**

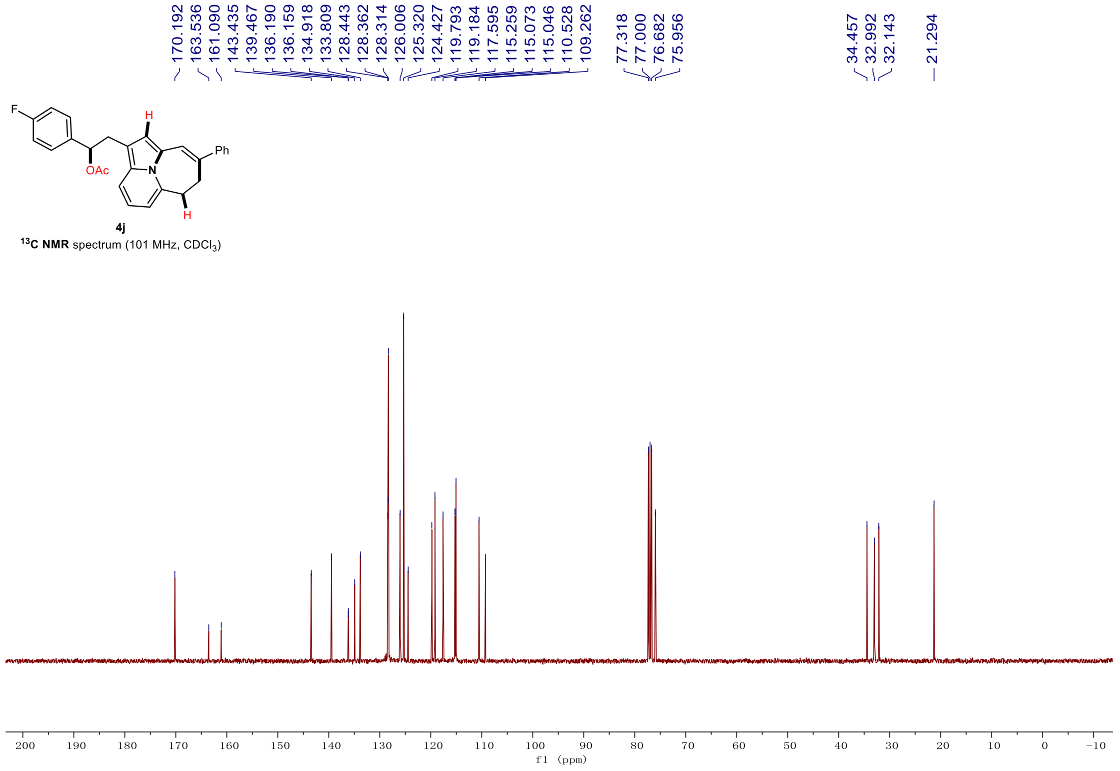

Supplementary Figure 173. ^{13}C NMR (101 MHz, $CDCl_3$) spectra for **4j**

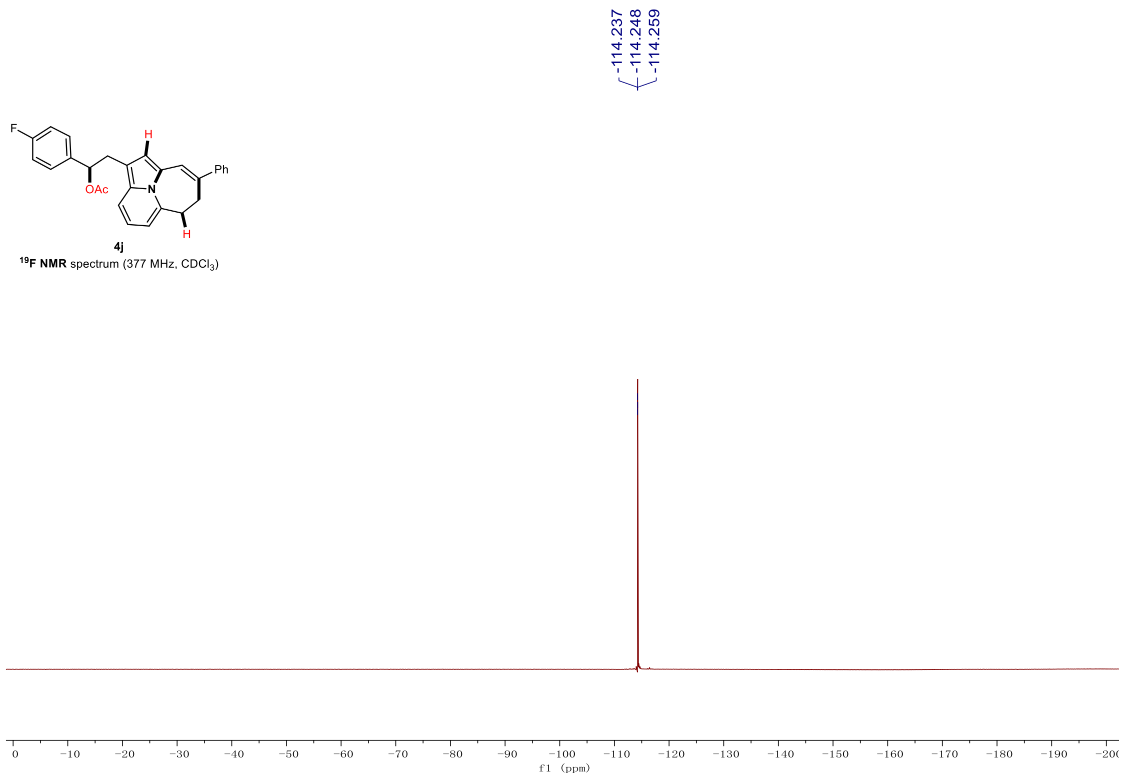

Supplementary Figure 174. ^{19}F NMR (377 MHz, $CDCl_3$) spectra for **4j**

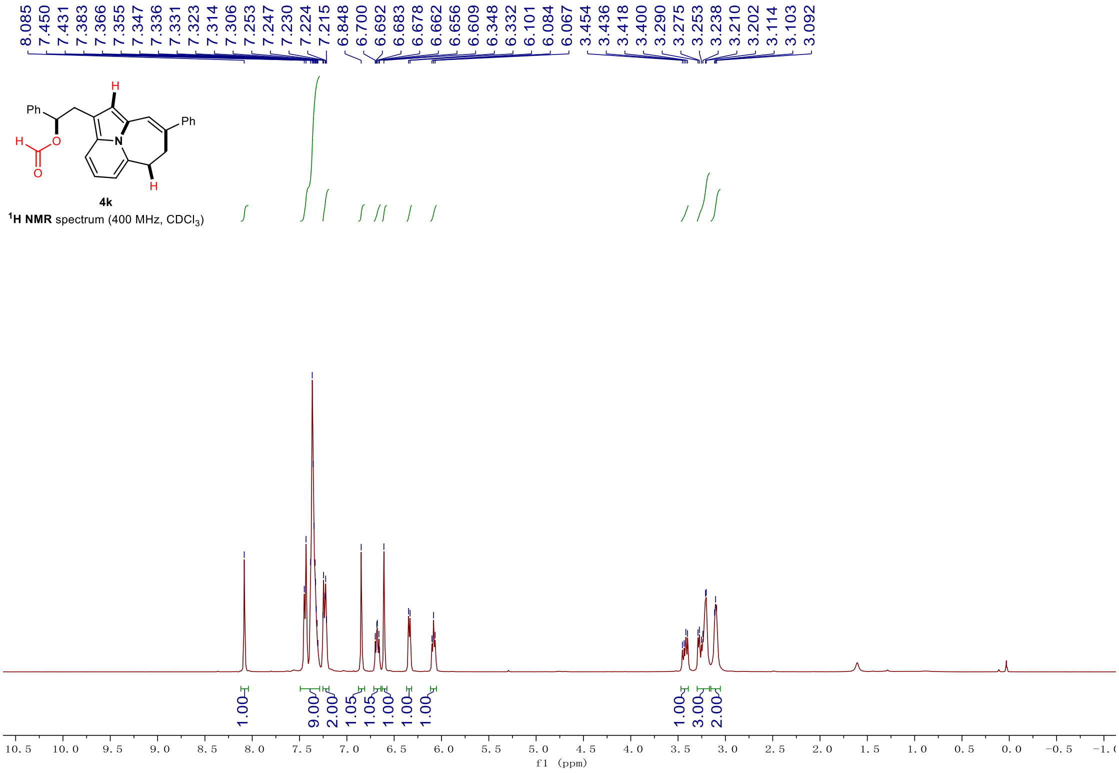

Supplementary Figure 175. ^{1}H NMR (400 MHz, $CDCl_3$) spectra for **4k**

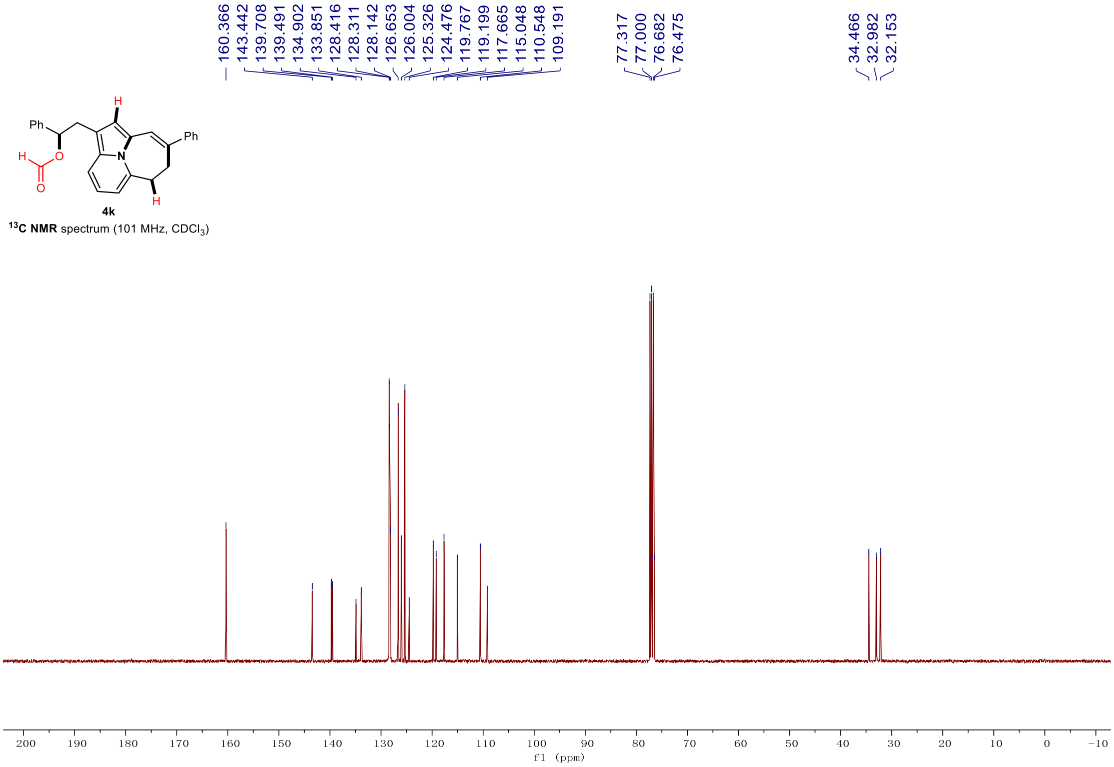

Supplementary Figure 176. ^{13}C NMR (101 MHz, $CDCl_3$) spectra for **4k**

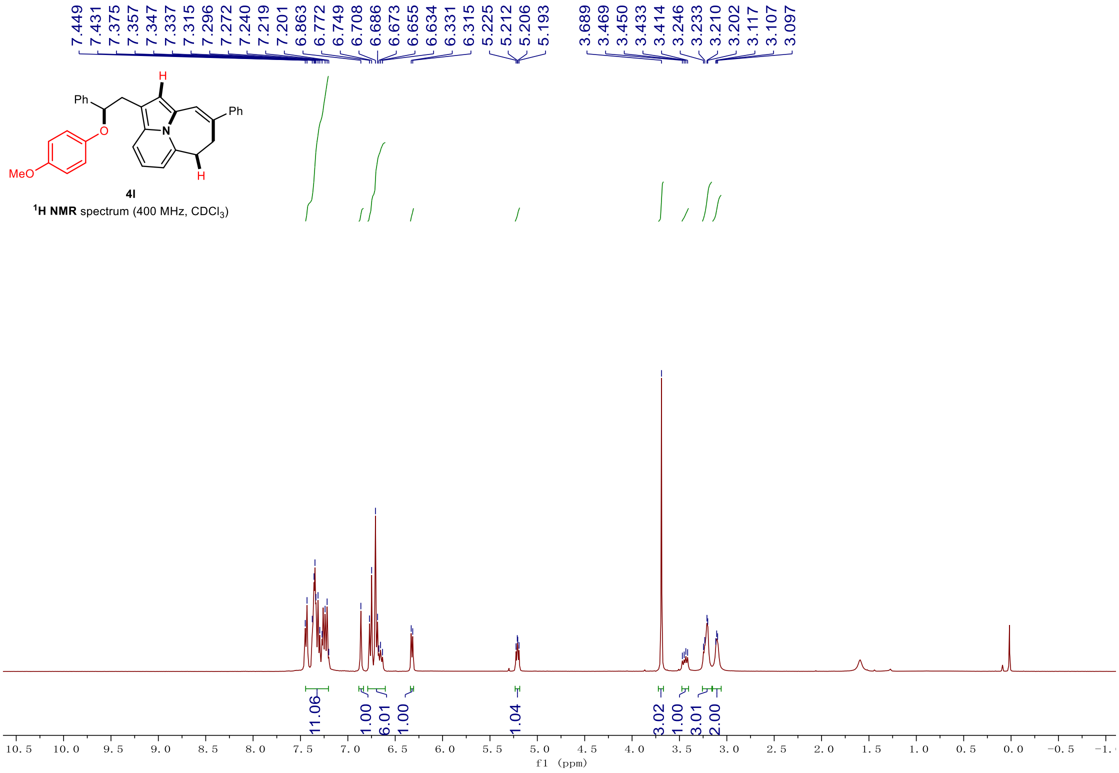

Supplementary Figure 177. ^{1}H NMR (400 MHz, CDCl$_3$) spectra for **4l**

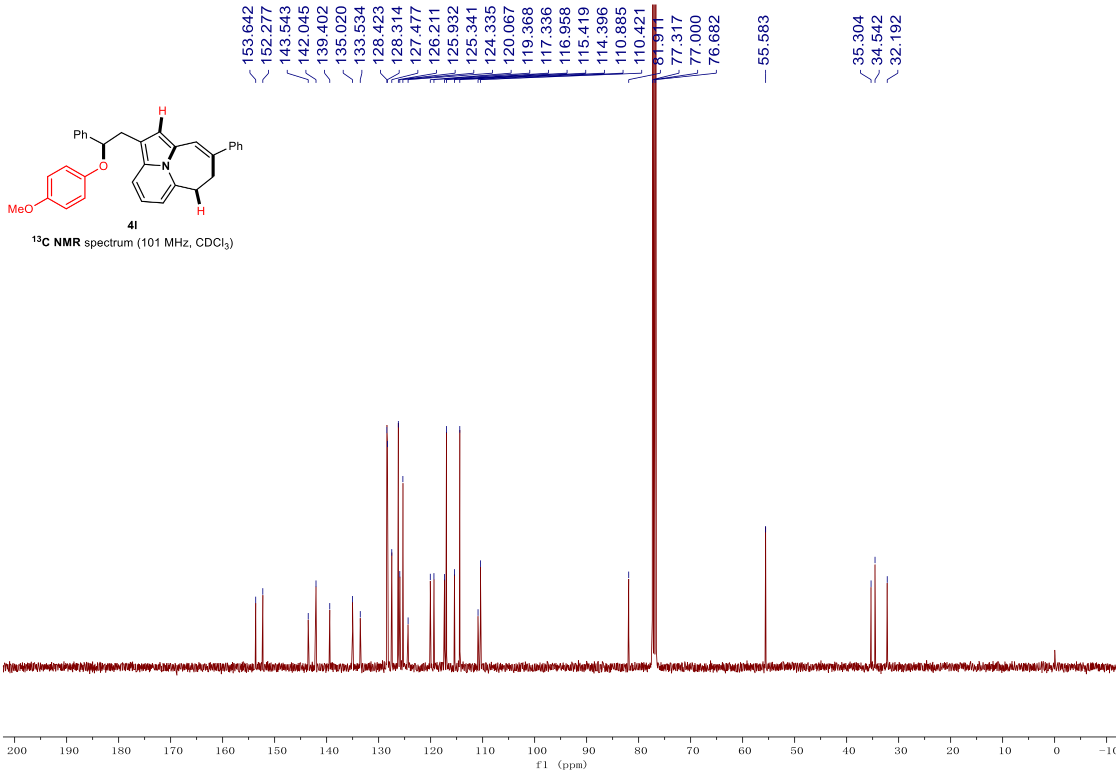

Supplementary Figure 178. ^{13}C NMR (101 MHz, CDCl$_3$) spectra for **4l**

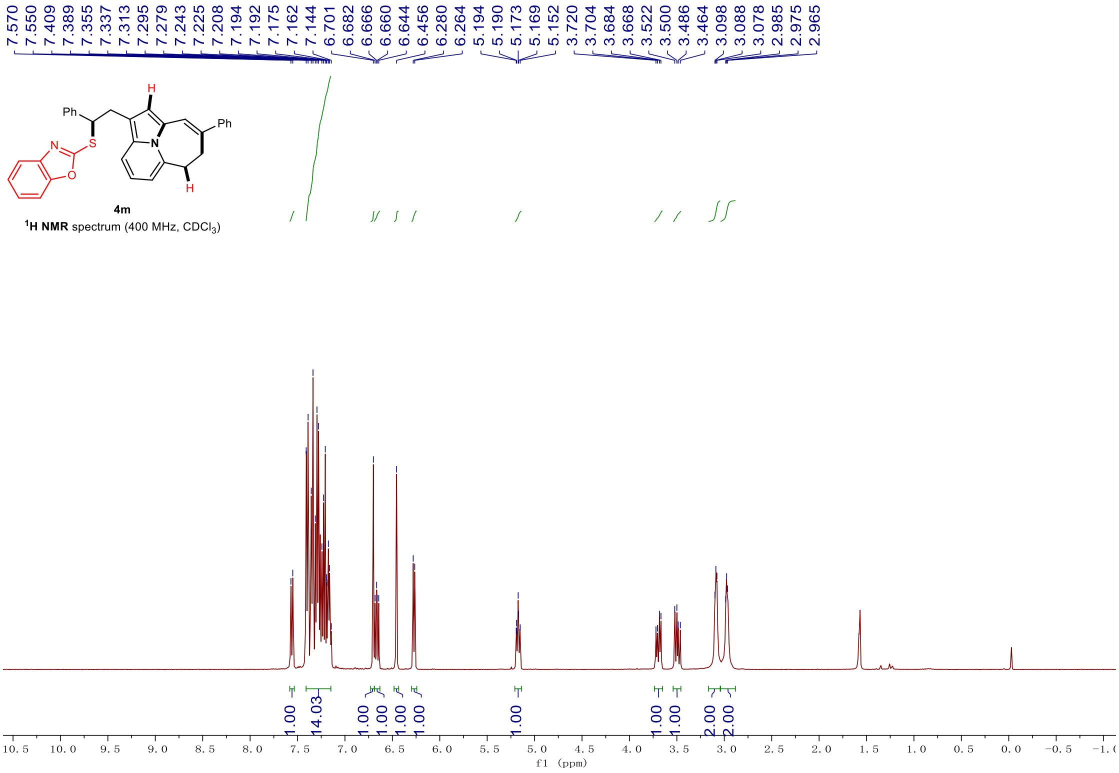

Supplementary Figure 179. ^{1}H NMR (400 MHz, $CDCl_3$) spectra for **4m**

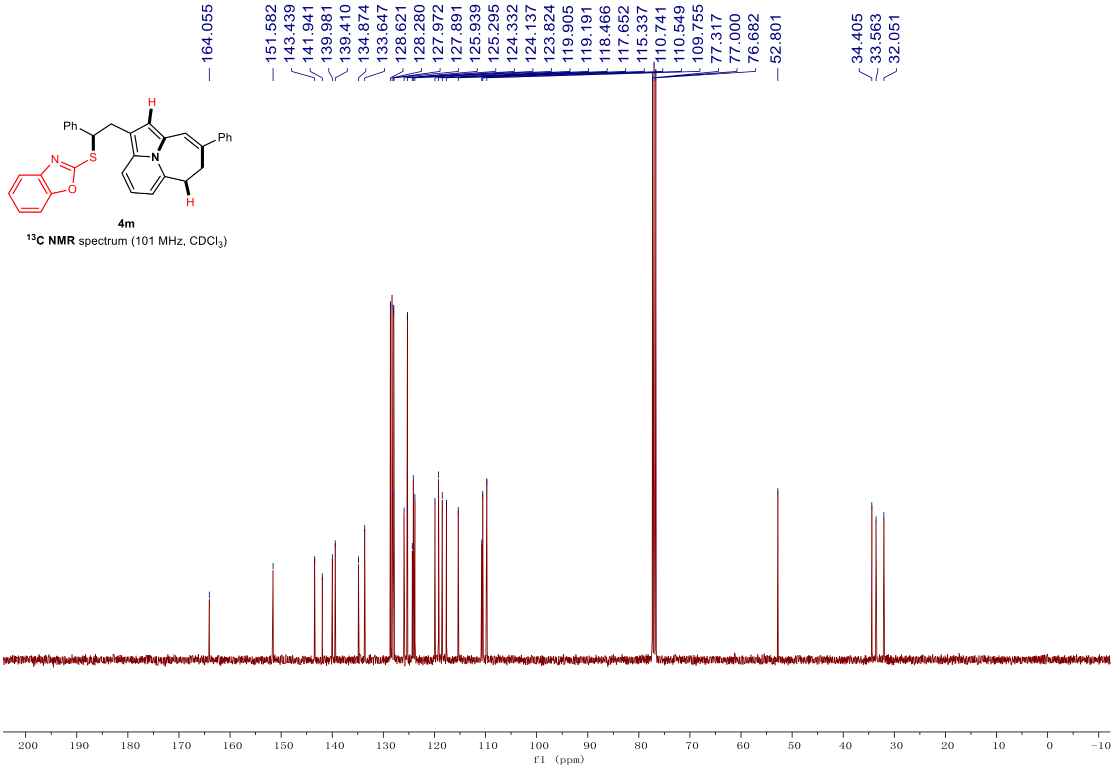

Supplementary Figure 180. ^{13}C NMR (101 MHz, $CDCl_3$) spectra for **4m**

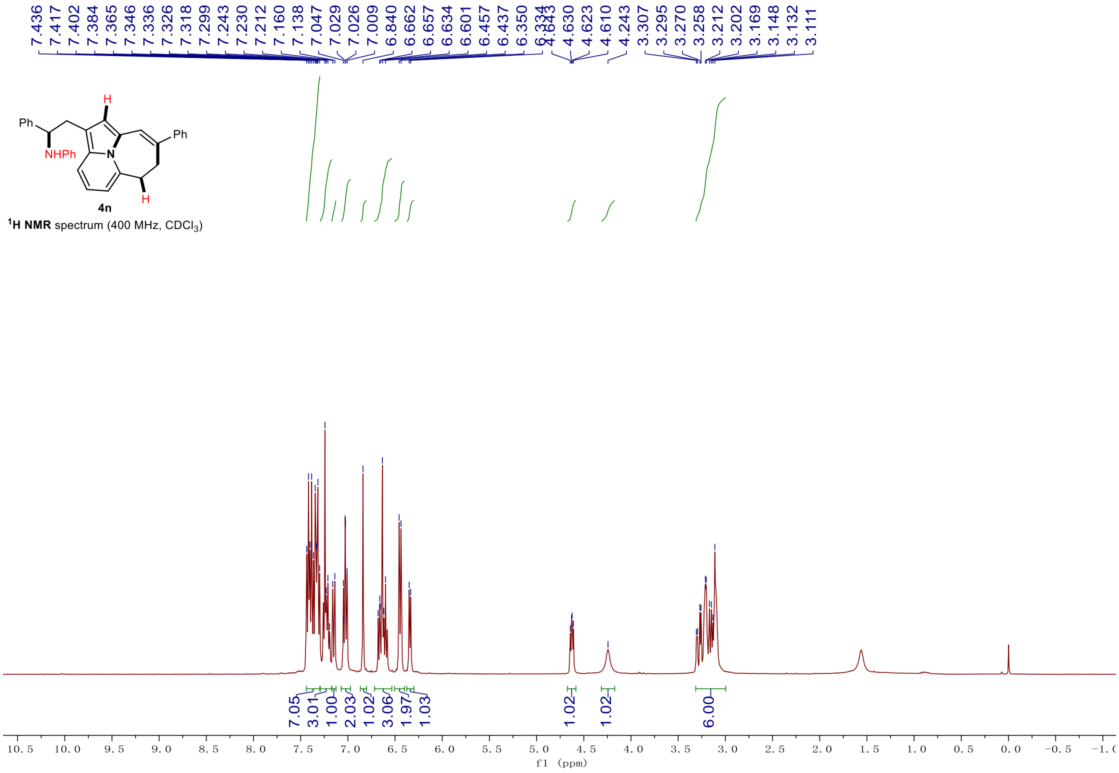

Supplementary Figure 181. ^{1}H NMR (400 MHz, $CDCl_3$) spectra for **4n**

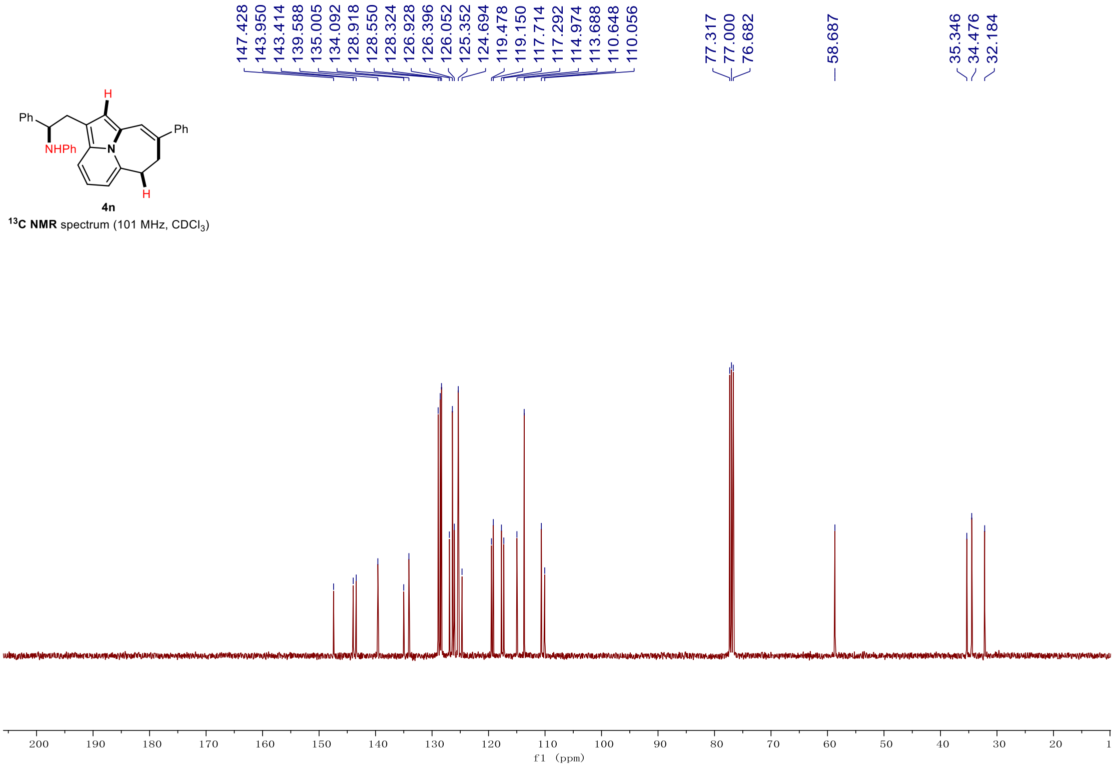

Supplementary Figure 182. ^{13}C NMR (101 MHz, $CDCl_3$) spectra for **4n**

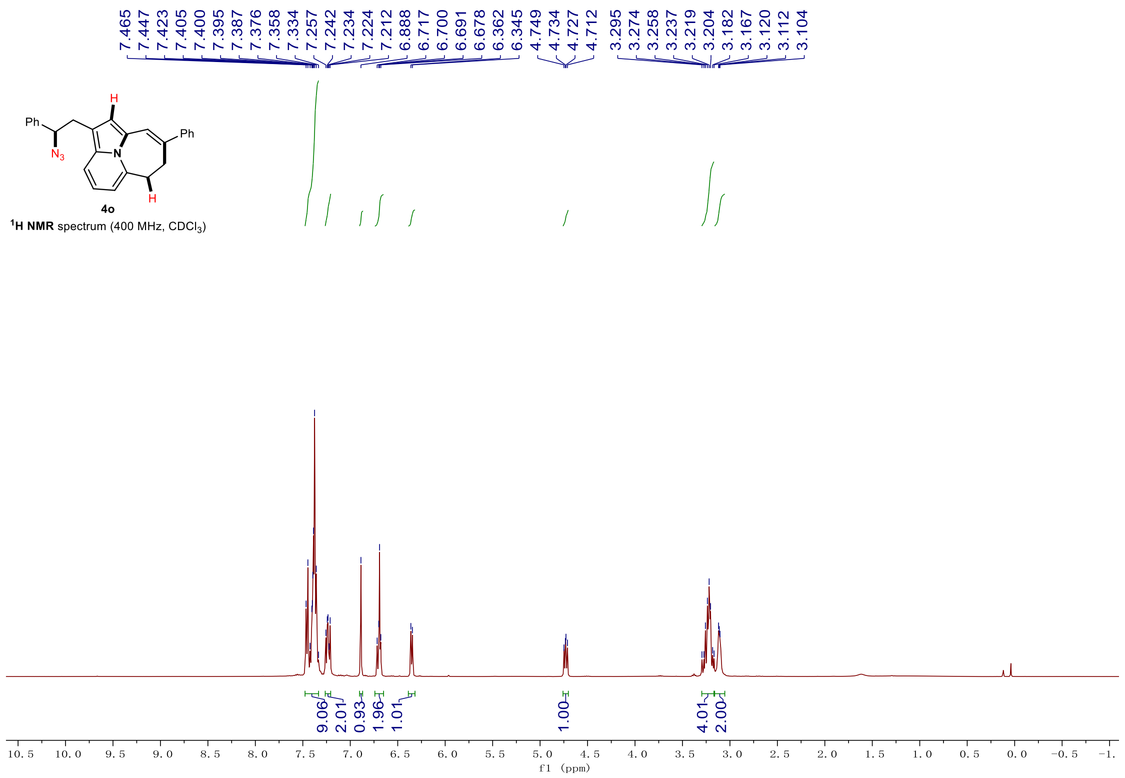

Supplementary Figure 183. ^{1}H NMR (400 MHz, $CDCl_3$) spectra for **4o**

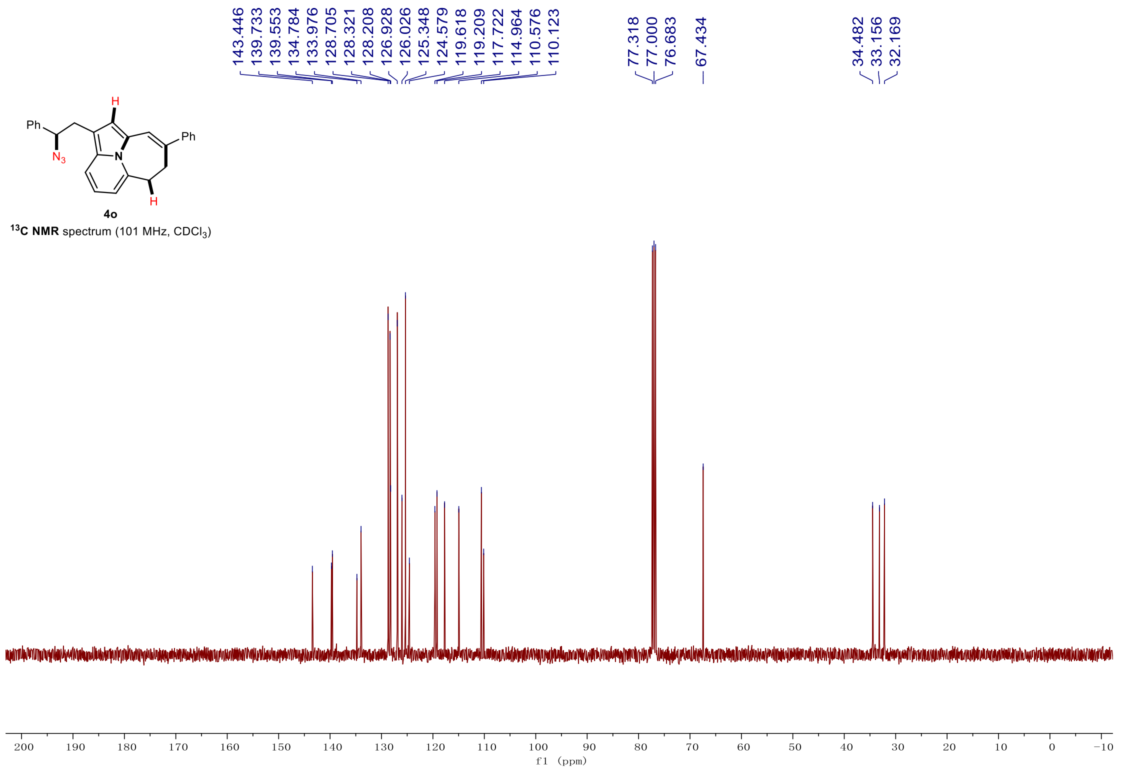

Supplementary Figure 184. ^{13}C NMR (101 MHz, $CDCl_3$) spectra for **4o**

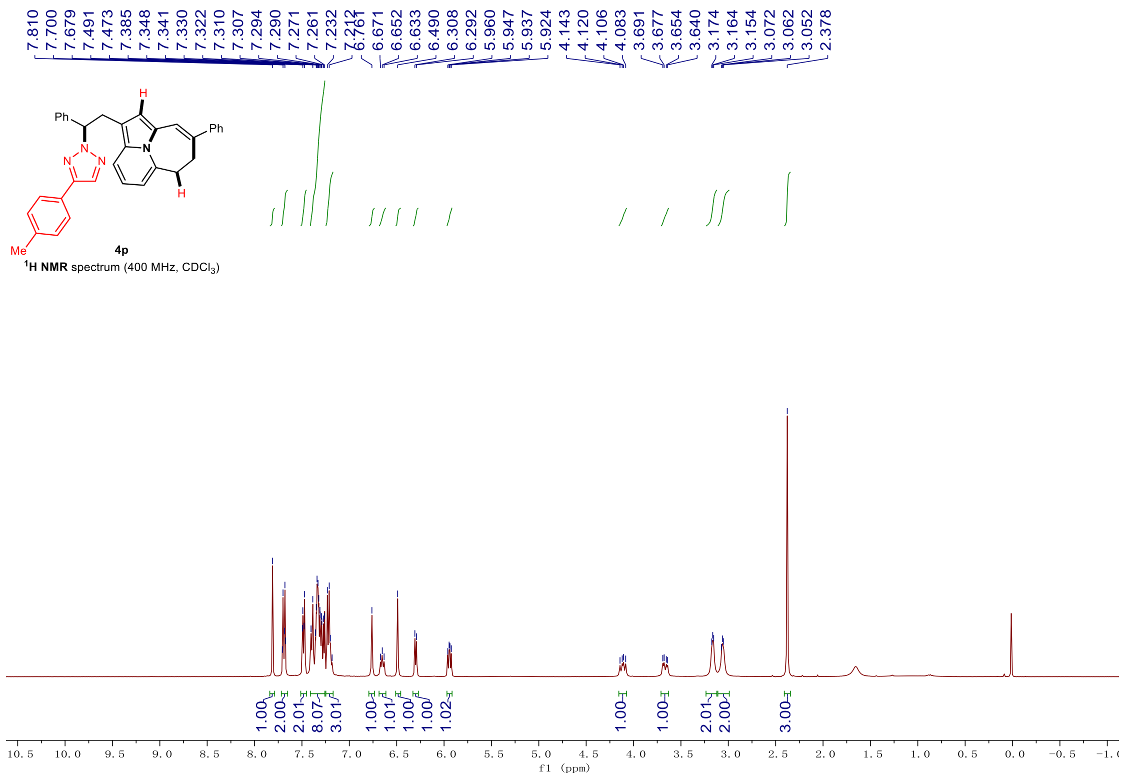

Supplementary Figure 185. ^{1}H NMR (400 MHz, $CDCl_3$) spectra for **4p**

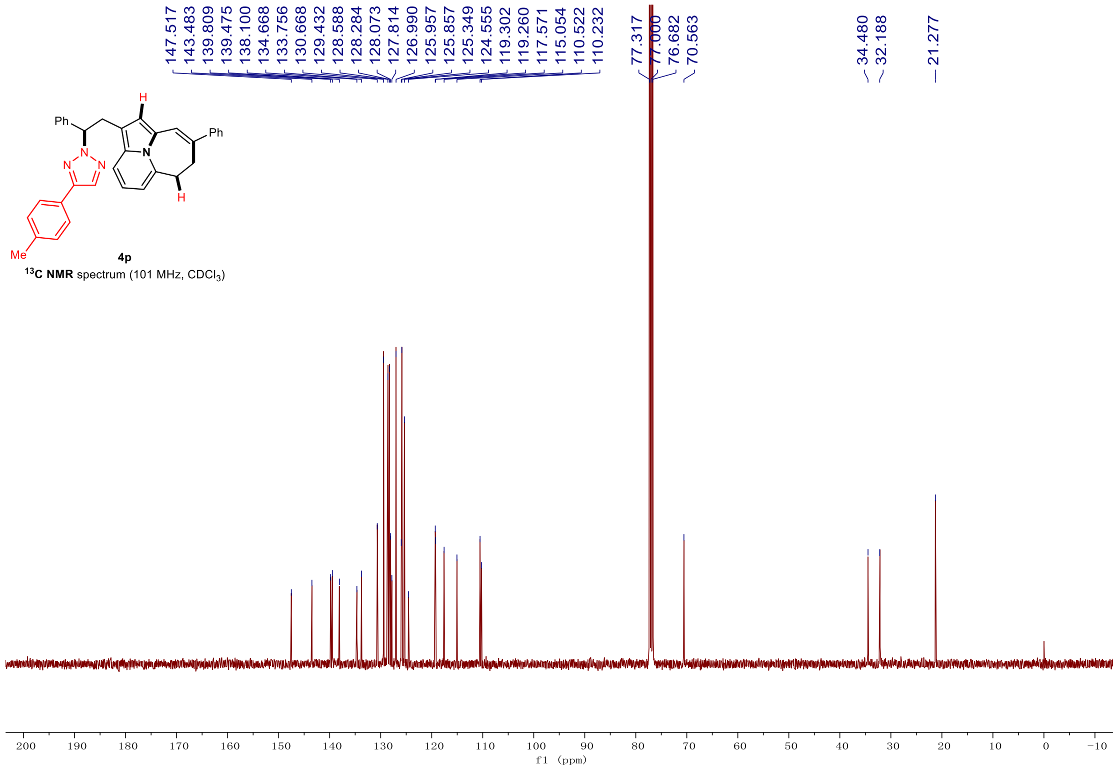

Supplementary Figure 186. ^{13}C NMR (101 MHz, $CDCl_3$) spectra for **4p**

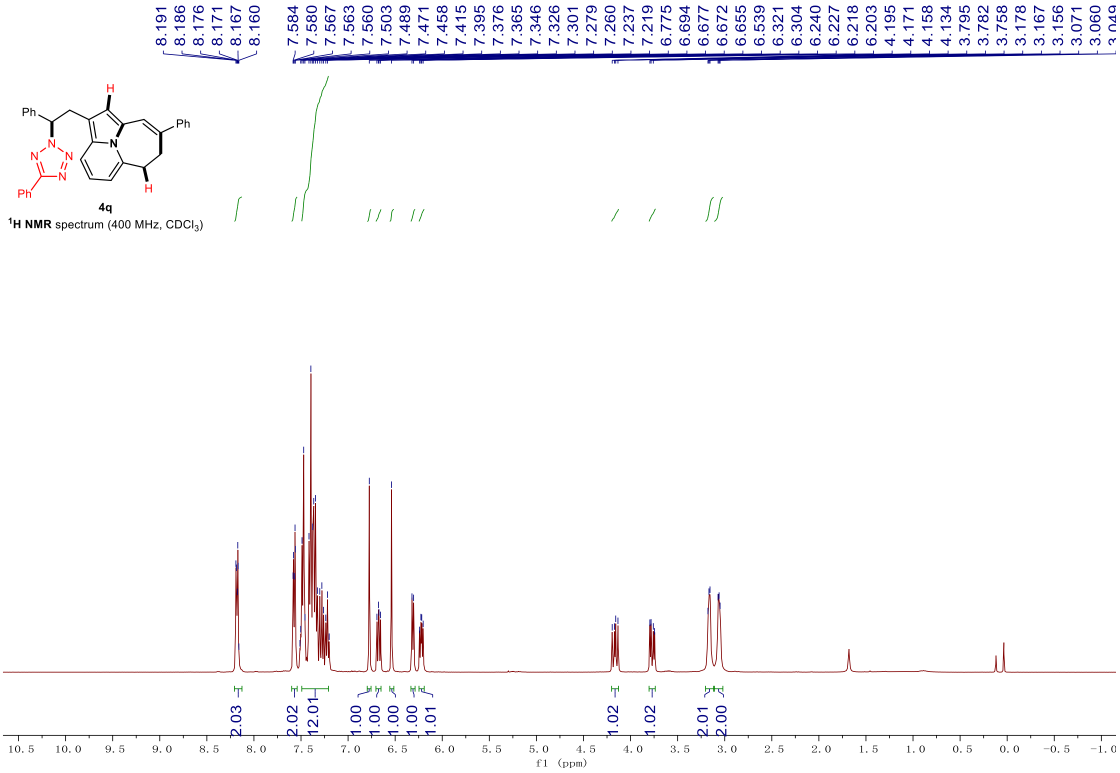

Supplementary Figure 187. ^{1}H NMR (400 MHz, CDCl$_3$) spectra for **4q**

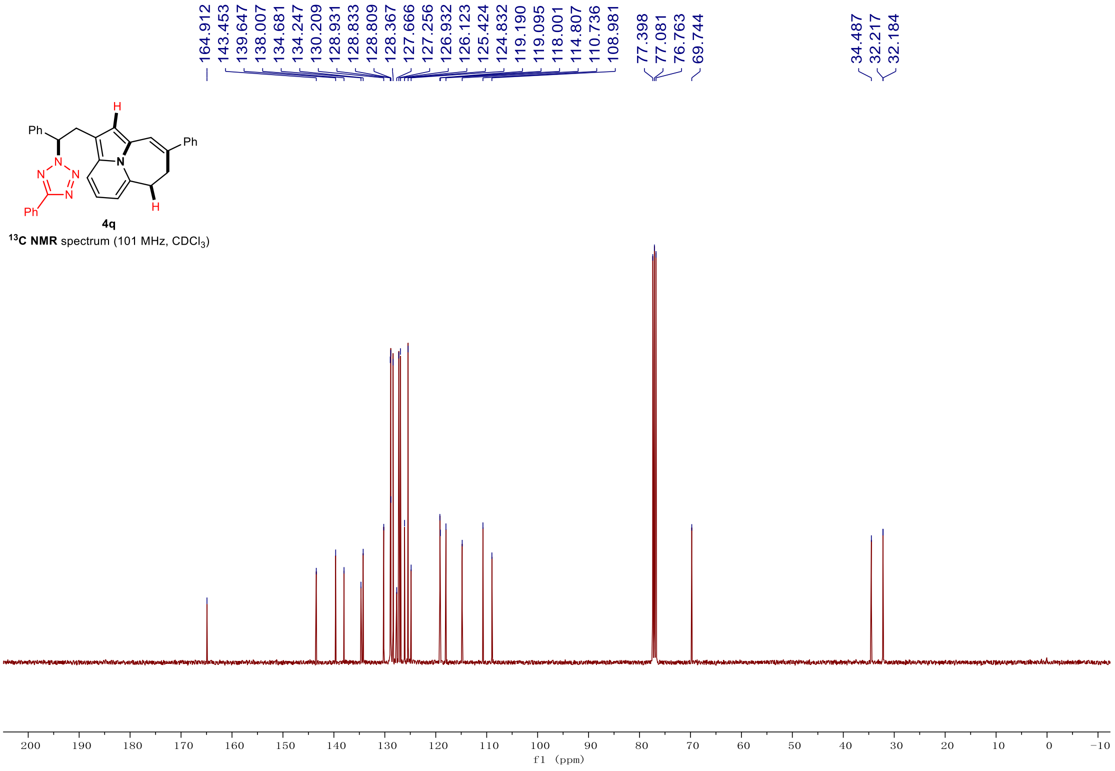

Supplementary Figure 188. ^{13}C NMR (101 MHz, CDCl$_3$) spectra for **4q**

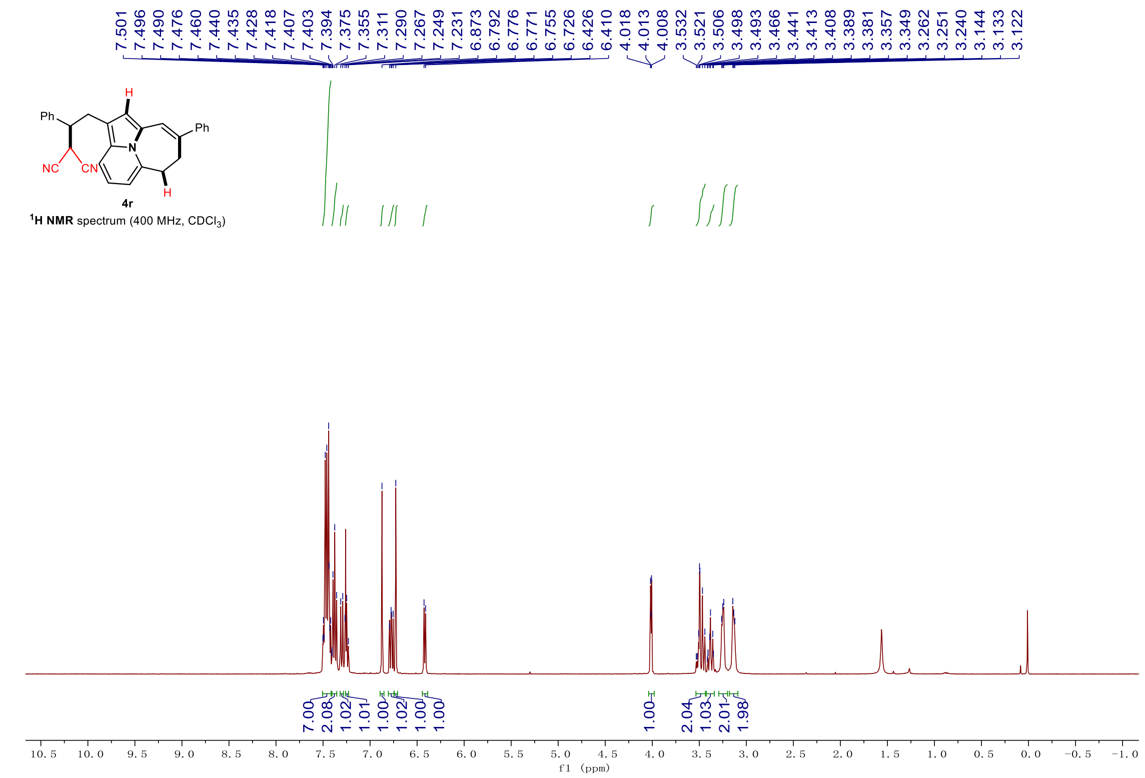

Supplementary Figure 189. ^{1}H NMR (400 MHz, $CDCl_3$) spectra for **4r**

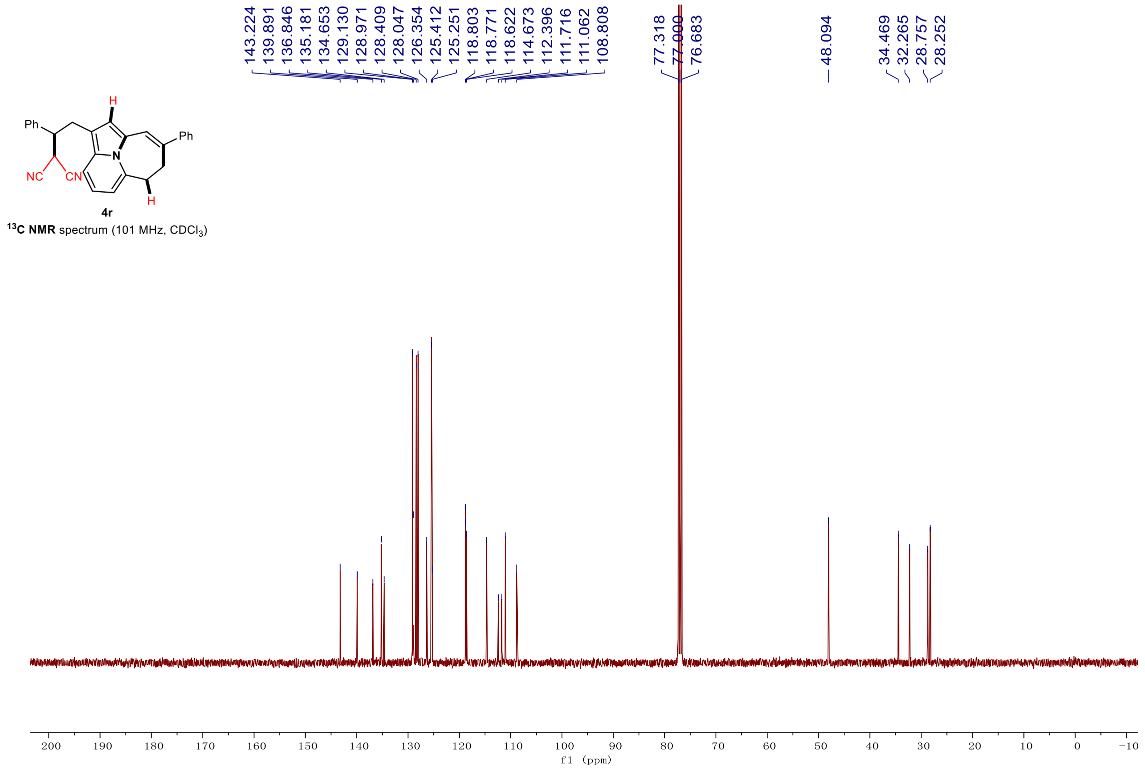

Supplementary Figure 190. ^{13}C NMR (101 MHz, $CDCl_3$) spectra for **4r**

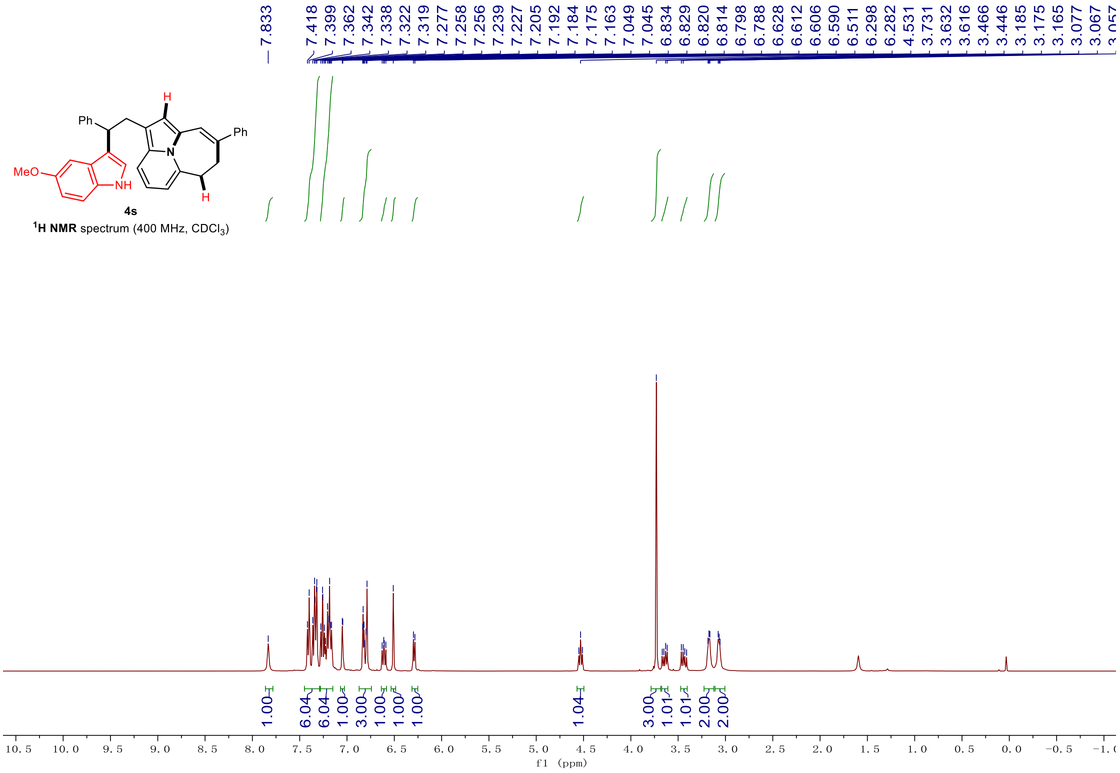

Supplementary Figure 191. ^{1}H NMR (400 MHz, $CDCl_3$) spectra for **4s**

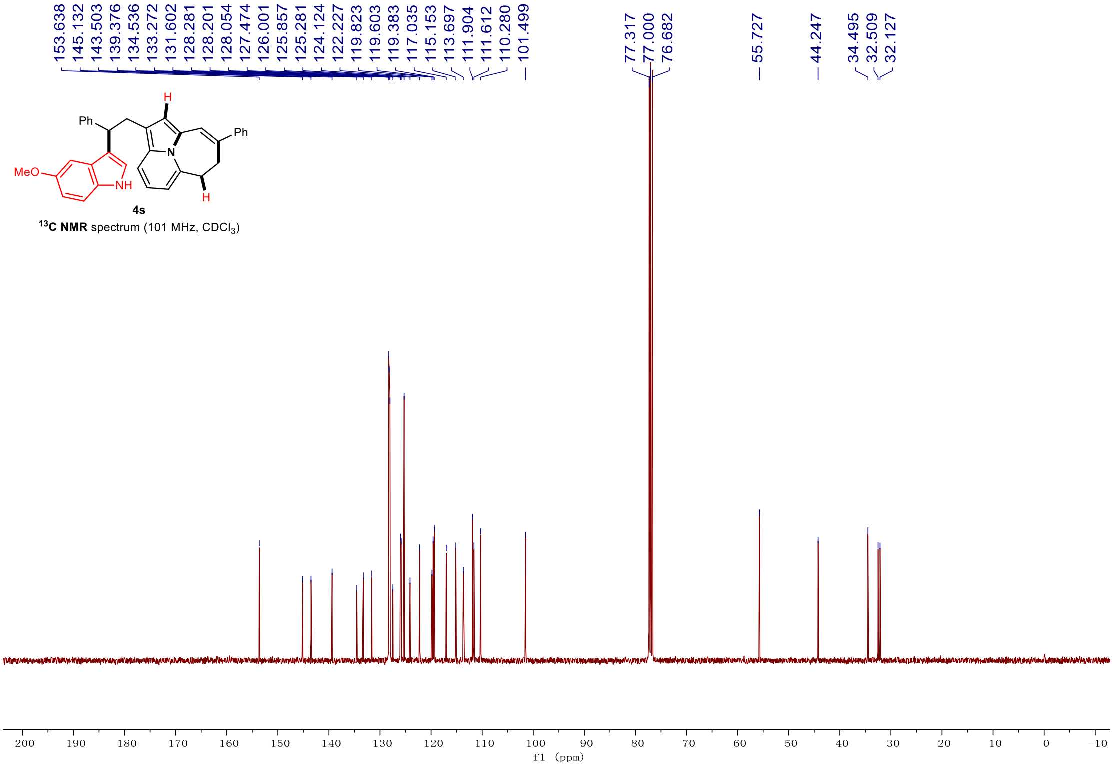

Supplementary Figure 192. ^{13}C NMR (101 MHz, $CDCl_3$) spectra for **4s**

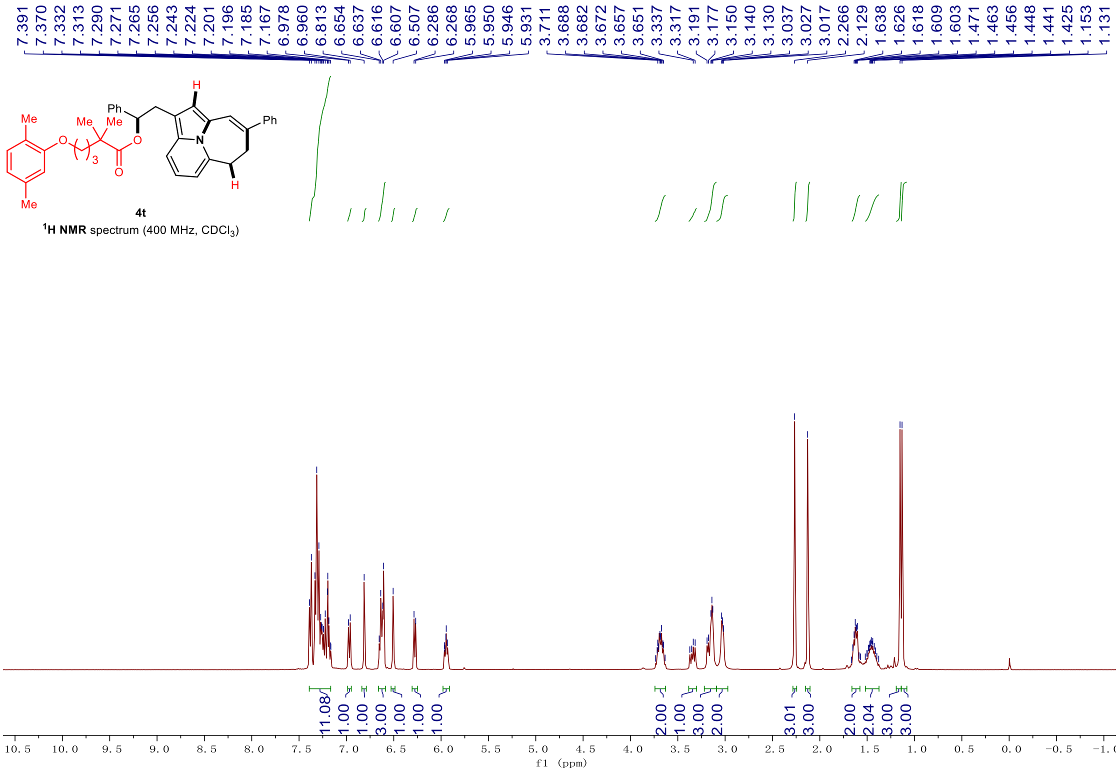

Supplementary Figure 193. ^{1}H NMR (400 MHz, CDCl$_3$) spectra for **4t**

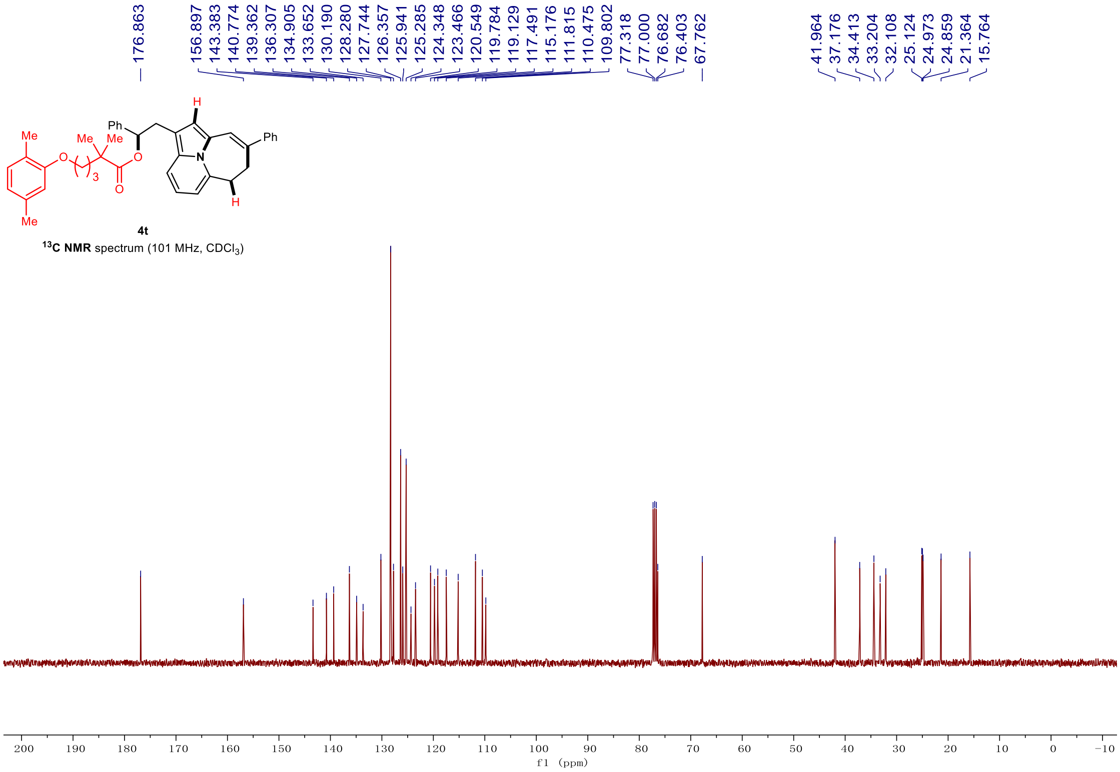

Supplementary Figure 194. ^{13}C NMR (101 MHz, CDCl$_3$) spectra for **4t**

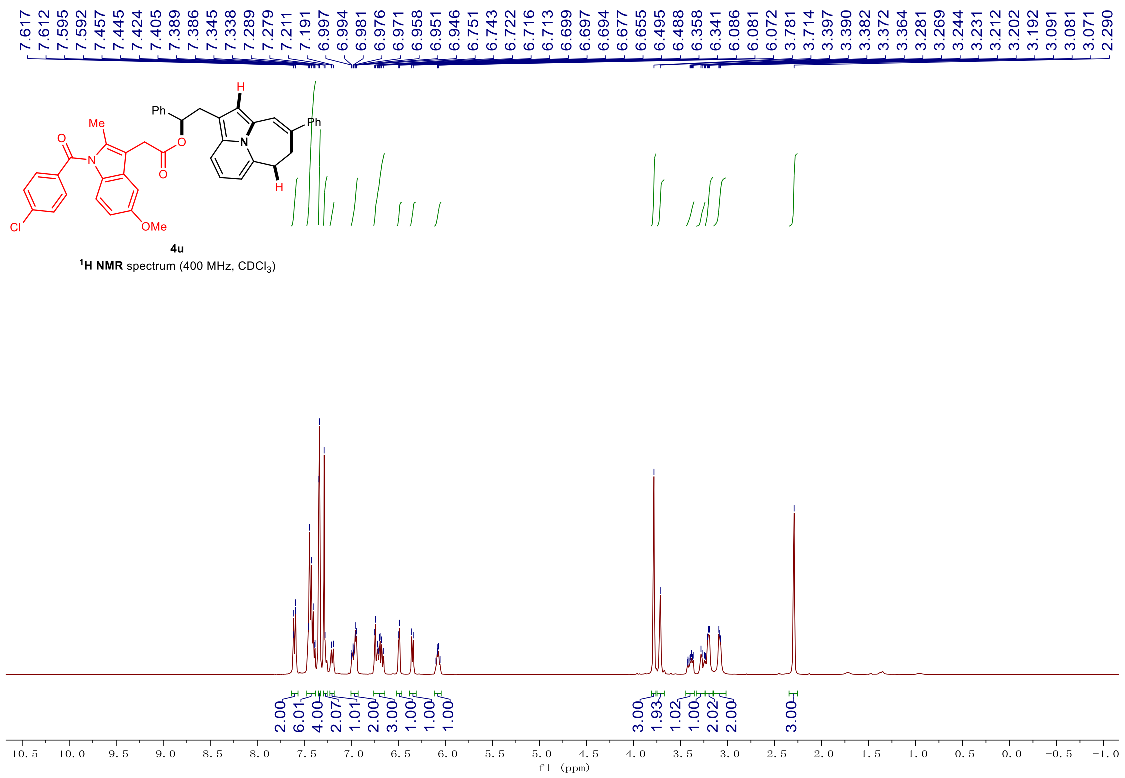

Supplementary Figure 195. ^{1}H NMR (400 MHz, $CDCl_3$) spectra for **4u**

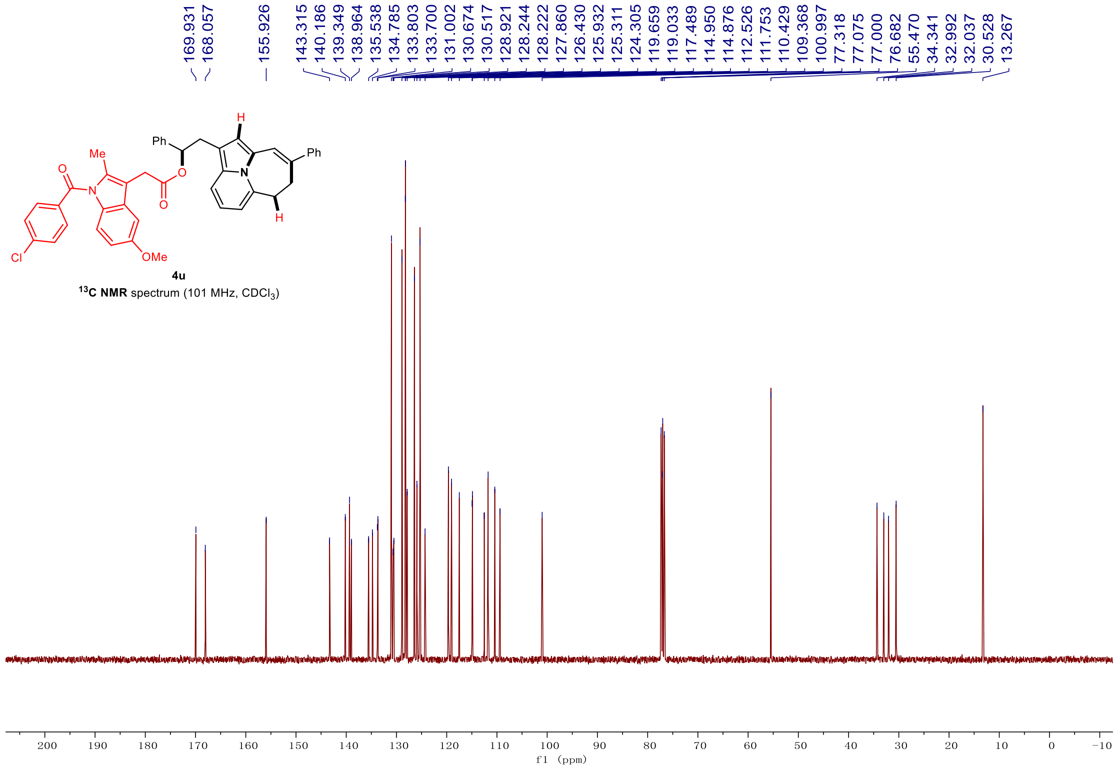

Supplementary Figure 196. ^{13}C NMR (101 MHz, $CDCl_3$) spectra for **4u**

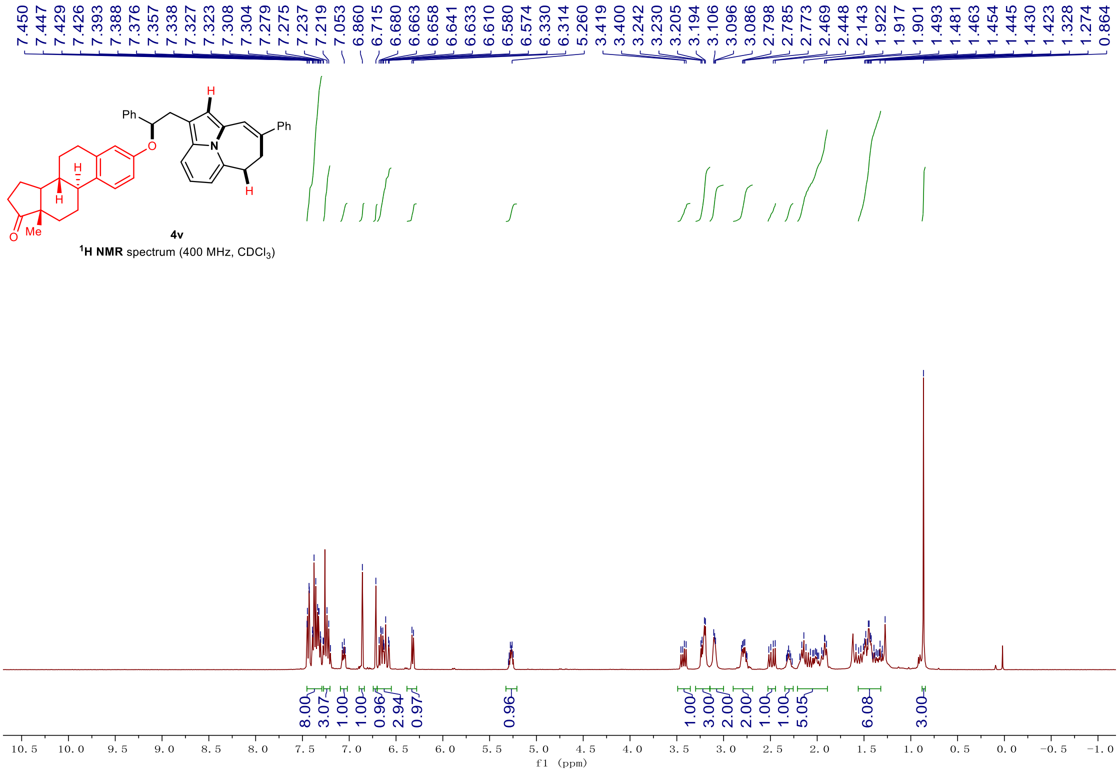

Supplementary Figure 197. ^{1}H NMR (400 MHz, $CDCl_3$) spectra for **4v**

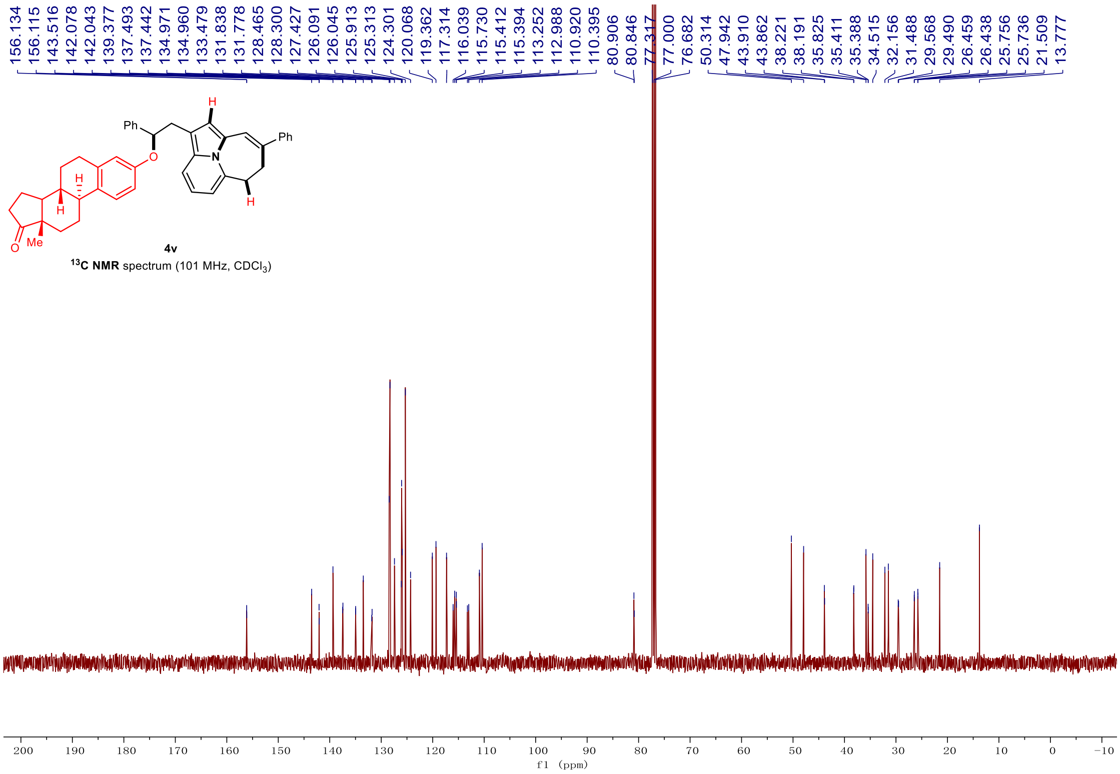

Supplementary Figure 198. ^{13}C NMR (101 MHz, $CDCl_3$) spectra for **4v**

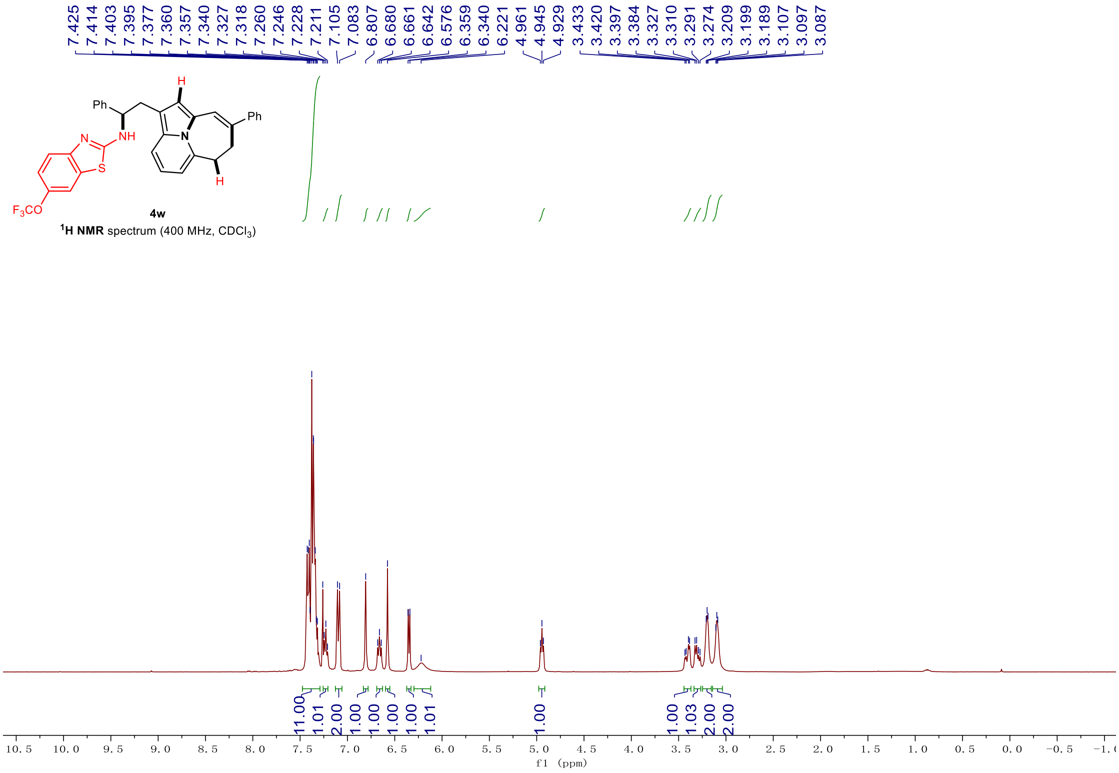

Supplementary Figure 199. ^{1}H NMR (400 MHz, $CDCl_3$) spectra for **4w**

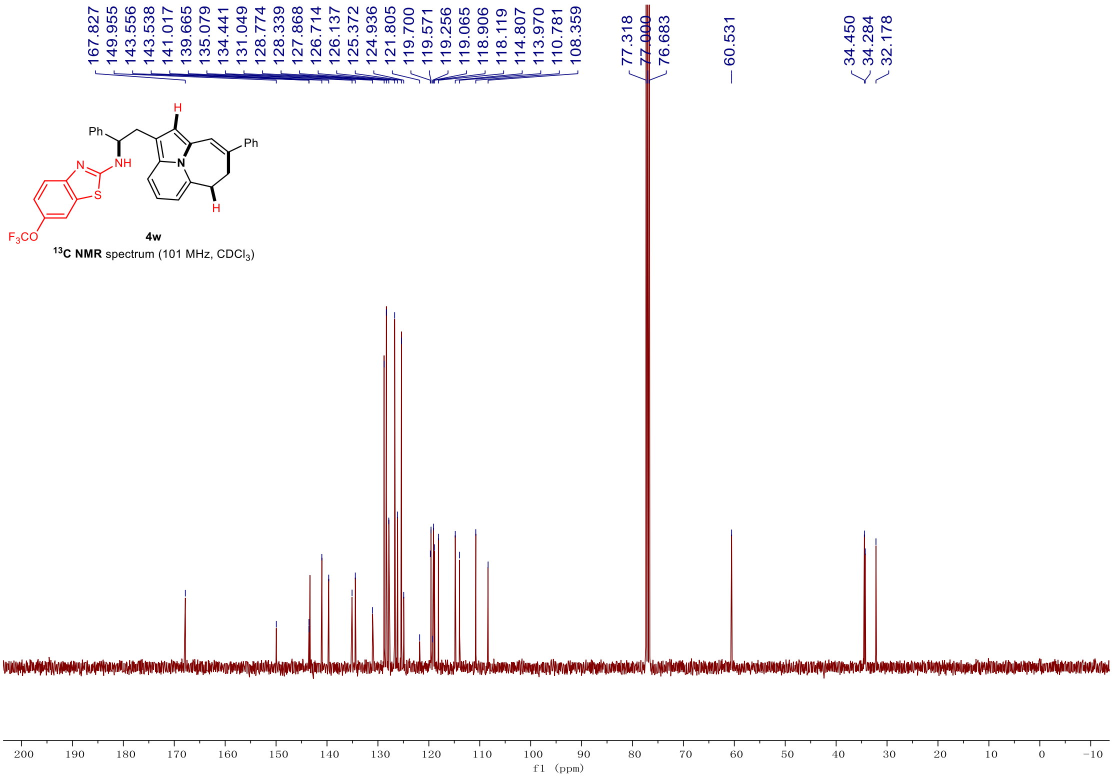

Supplementary Figure 200. ^{13}C NMR (101 MHz, $CDCl_3$) spectra for **4w**

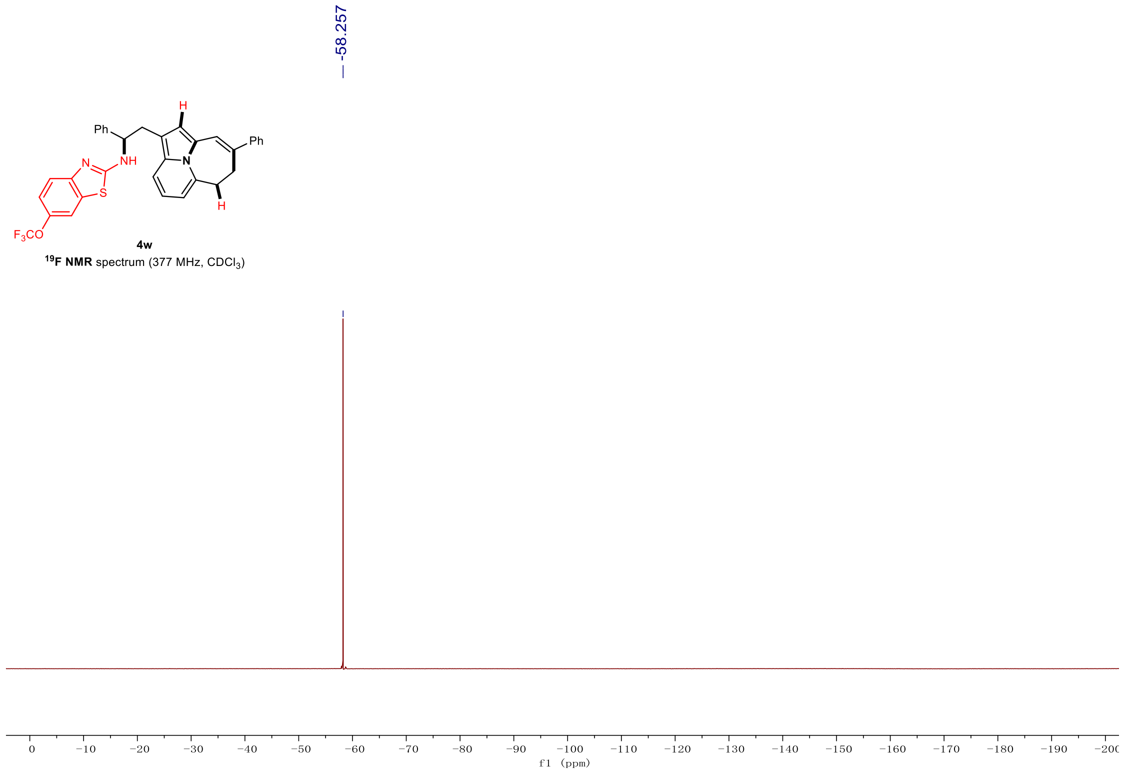

Supplementary Figure 201. ^{19}F NMR (377 MHz, $CDCl_3$) spectra for **4w**

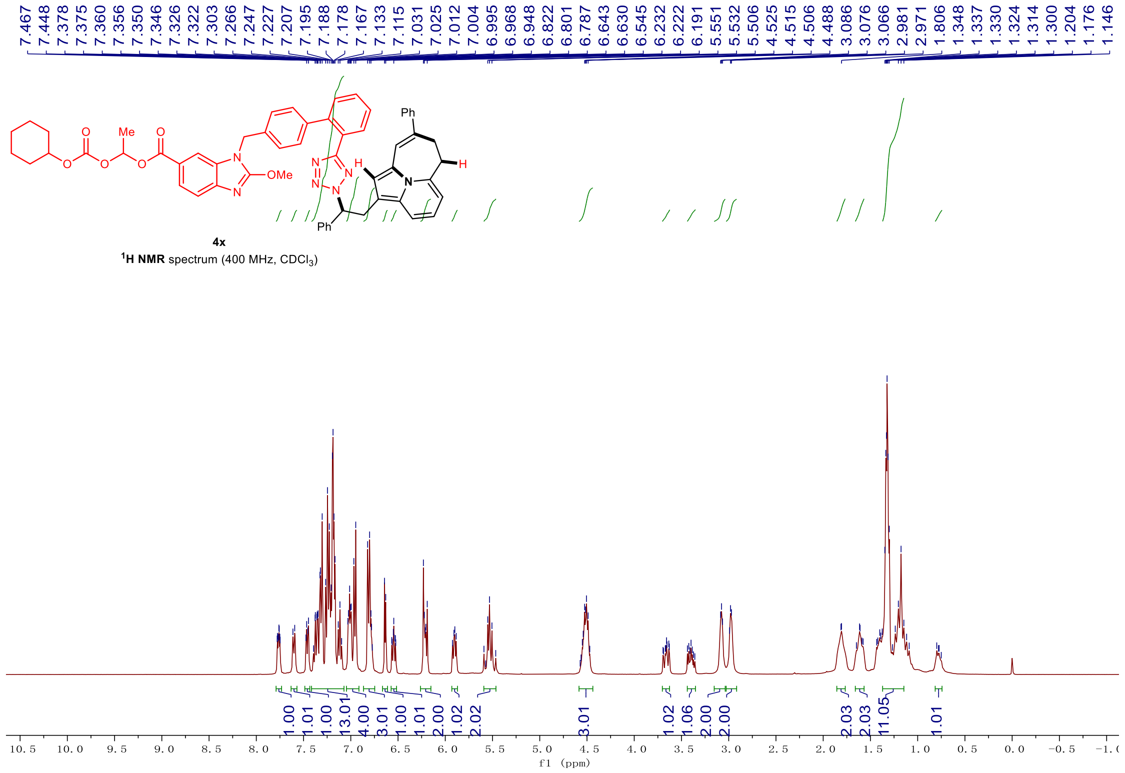

Supplementary Figure 202. ^{1}H NMR (400 MHz, $CDCl_3$) spectra for **4x**

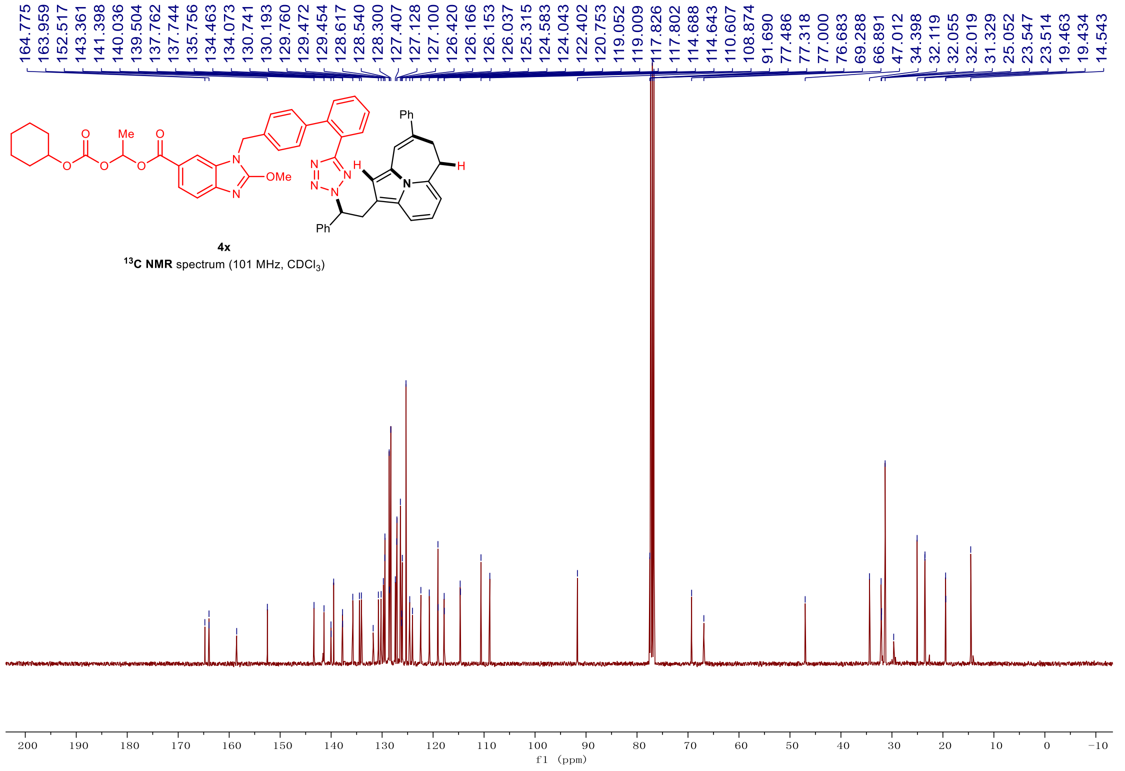

Supplementary Figure 203. ^{13}C NMR (101 MHz, $CDCl_3$) spectra for **4x**

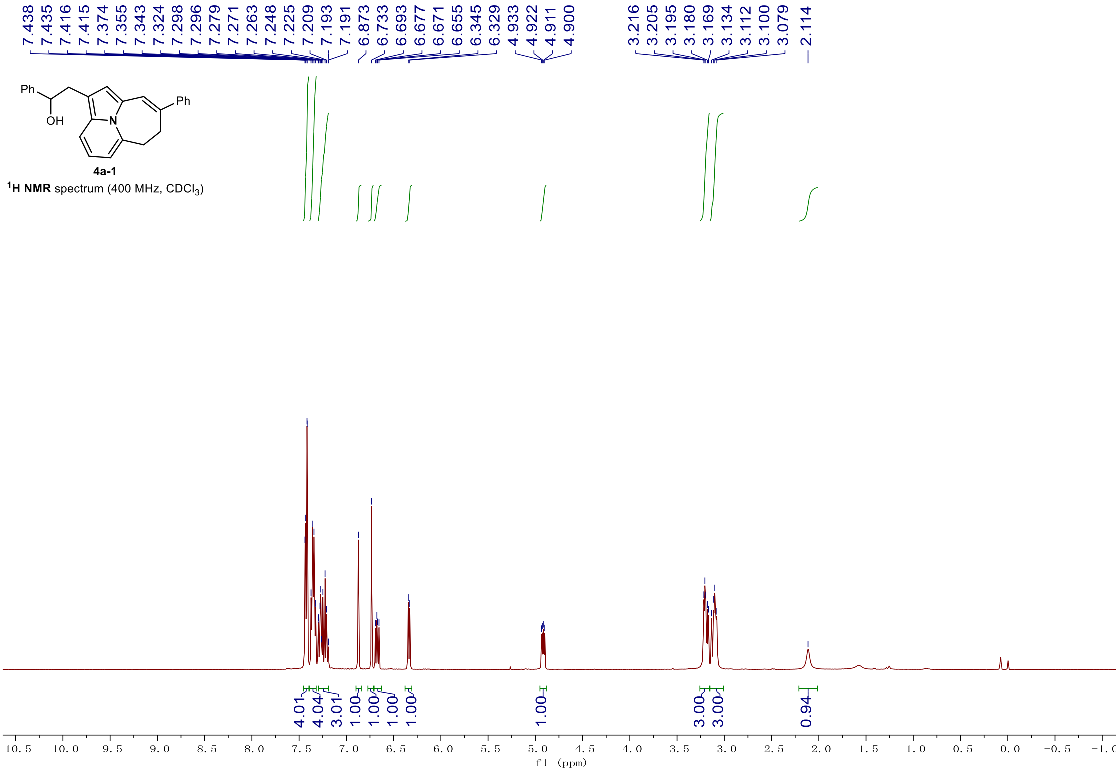

Supplementary Figure 204. ^{1}H NMR (400 MHz, $CDCl_3$) spectra for **4a-1**

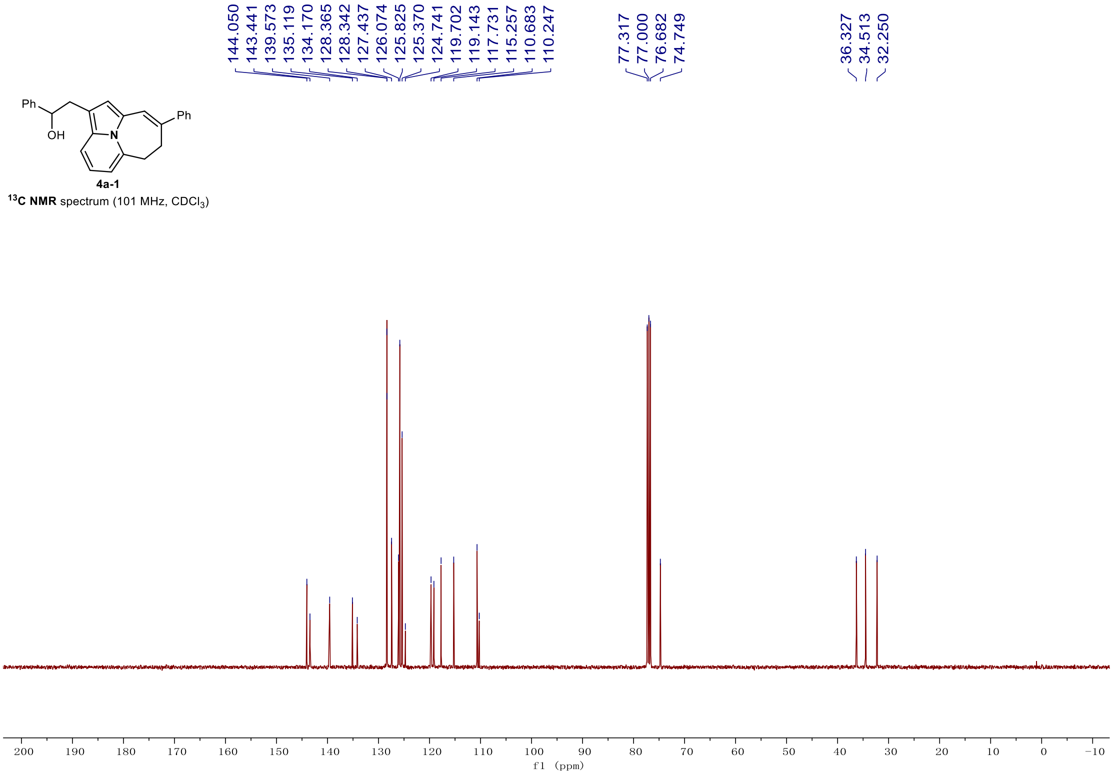

Supplementary Figure 205. ^{13}C NMR (101 MHz, $CDCl_3$) spectra for **4a-1**

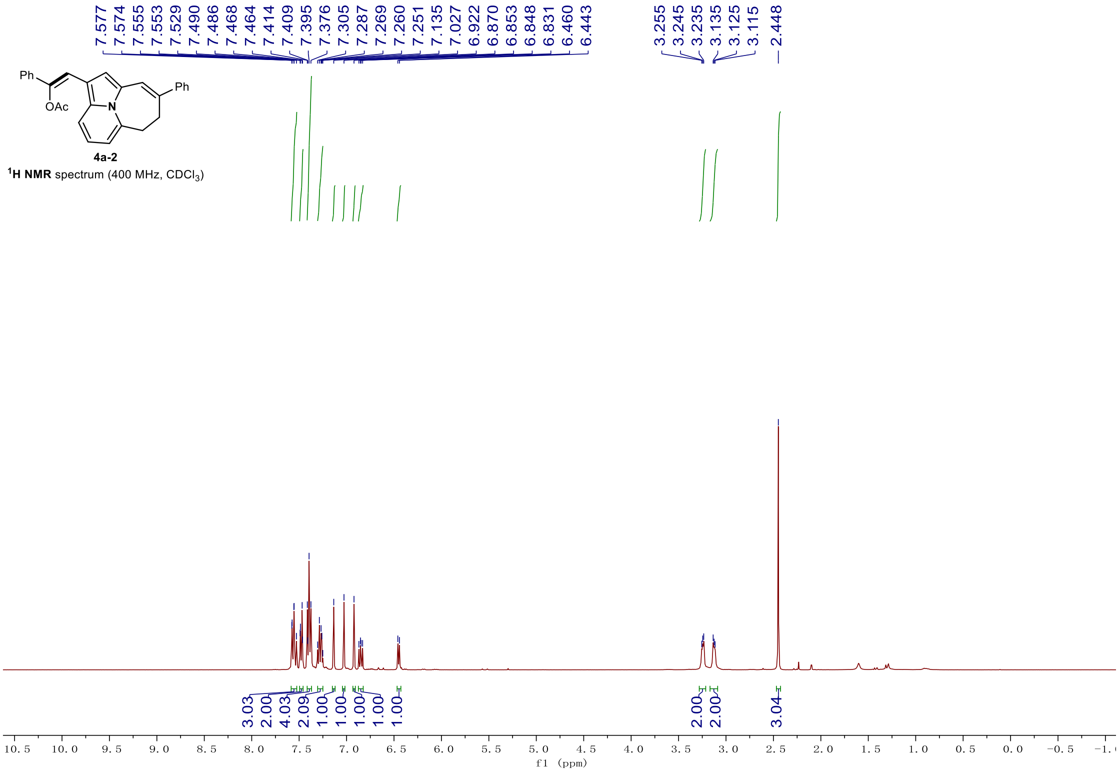

Supplementary Figure 206. ^{1}H NMR (400 MHz, CDCl$_3$) spectra for **4a-2**

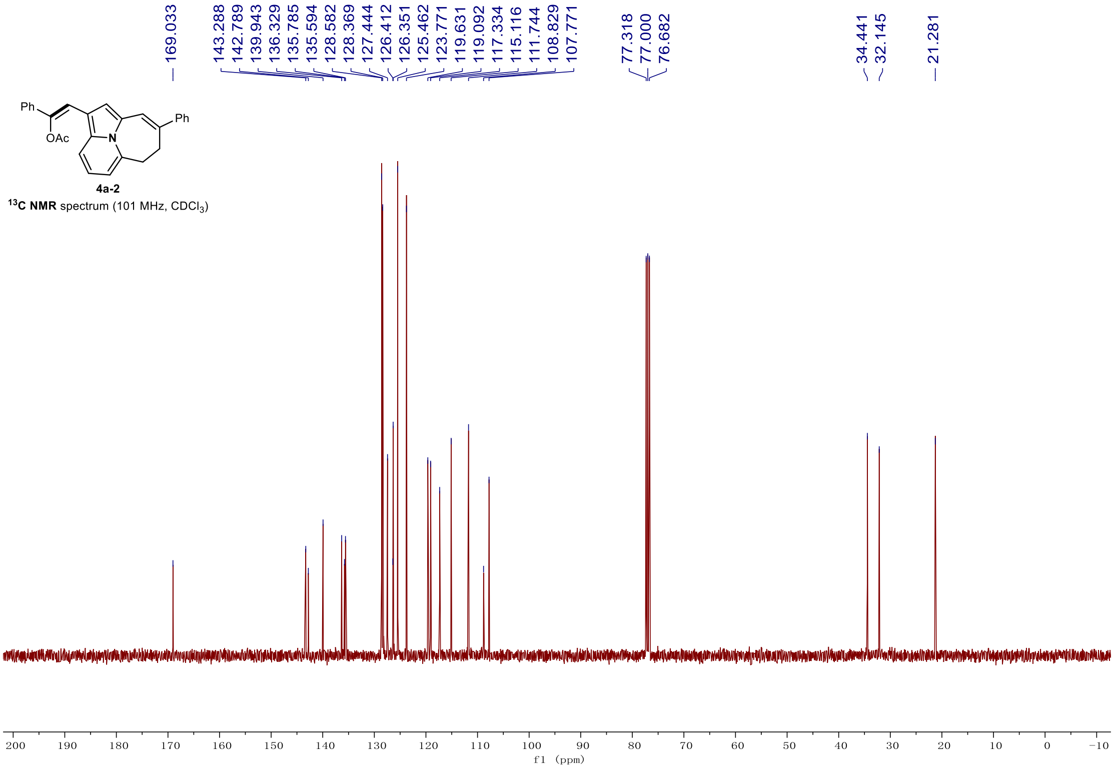

Supplementary Figure 207. ^{13}C NMR (101 MHz, CDCl$_3$) spectra for **4a-2**

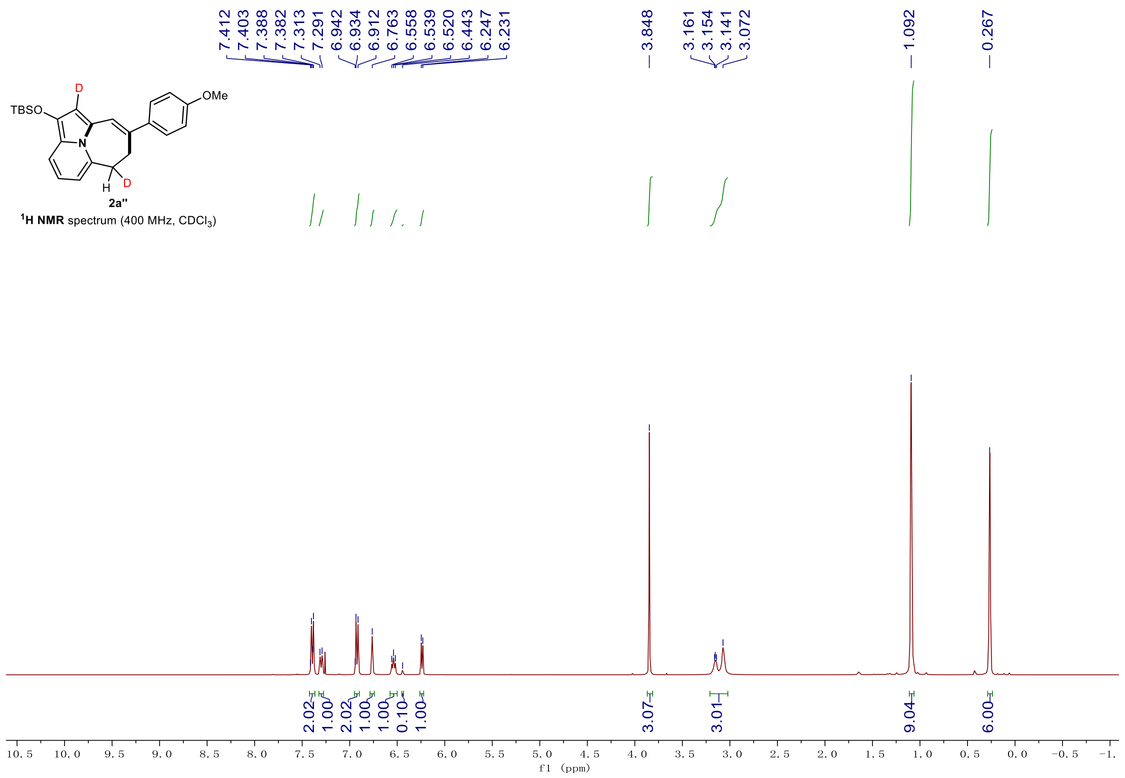

Supplementary Figure 208. ^{1}H NMR (400 MHz, $CDCl_3$) spectra for **2a''**

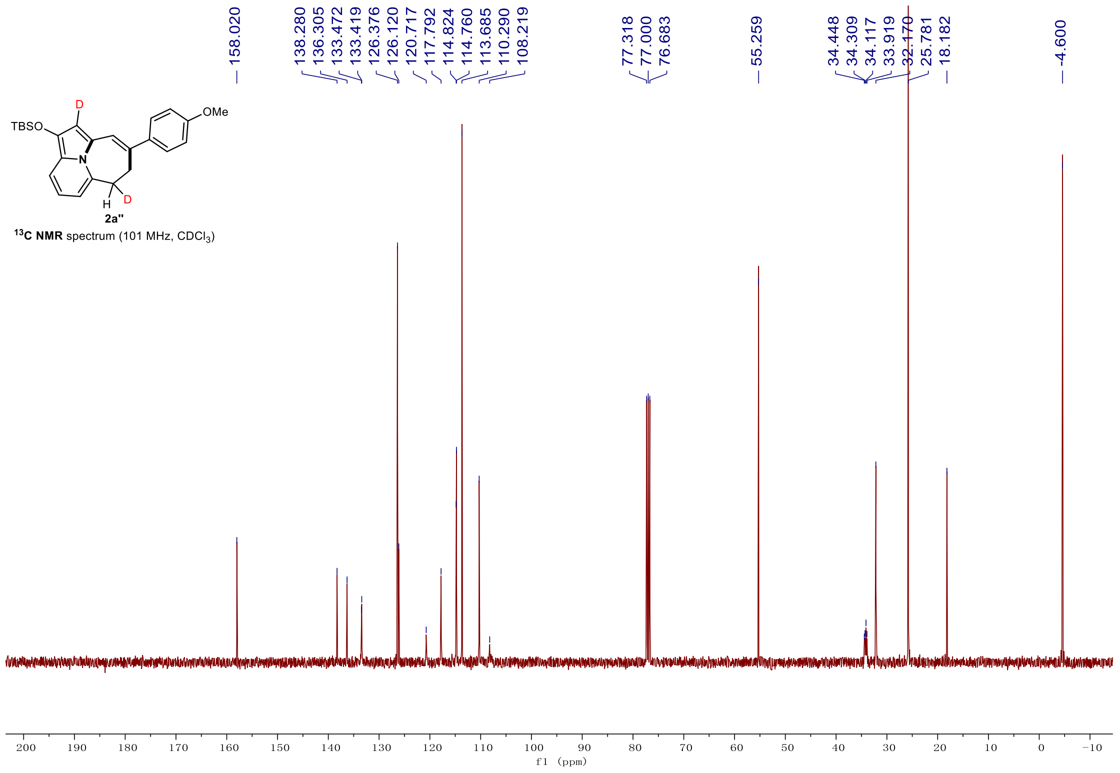

Supplementary Figure 209. ^{13}C NMR (101 MHz, $CDCl_3$) spectra for **2a''**

2 Supplementary References

1. Connors, K. A. The Karl Fischer Titration of Water. *Drug Dev. Ind. Pharm*. **14**, 1891–1903 (1988).

2. Cao, B., Wang, Y., Ding, K., Neamati, N. & Long, Y.-Q. Synthesis of the pyridinyl analogues of dibenzylideneacetone (pyr-dba) via an improved Claisen–Schmidt condensation, displaying diverse biological activities as curcumin analogues. *Org. Biomol. Chem*. **10**, 1239–1245 (2012).

3. Seregin, I. V. & Gevorgyan, V. Gold-Catalyzed 1,2-Migration of Silicon, Tin, and Germanium en Route to C-2 Substituted Fused Pyrrole-Containing Heterocycles. *J. Am. Chem. Soc.* **128**, 12050–12051 (2006).

4. Smith, C. R., Bunnelle, E. M., Rhodes, A. J. & Sarpong, R. Pt-Catalyzed Cyclization/1,2-Migration for the Synthesis of Indolizines, Pyrrolones, and Indolizinones. *Org. Lett*. **9**, 1169–1171 (2007).

5. Liu, R.-R. et al. Au-catalyzed ring-opening reactions of 2-(1-alkynyl-cyclopropyl)pyridines with nucleophiles. *Org. Biomol. Chem*. **13**, 4855–4858 (2015).
